# Deployment of Sulfinimines in Charge-Accelerated Sulfonium Rearrangement Enables a Surrogate Asymmetric Mannich Reaction

**DOI:** 10.1021/jacs.2c05368

**Published:** 2022-07-15

**Authors:** Minghao Feng, Ivan Mosiagin, Daniel Kaiser, Boris Maryasin, Nuno Maulide

**Affiliations:** † Institute of Organic Chemistry, University of Vienna, Währinger Strasse 38, 1090 Vienna, Austria; ‡ Institute of Theoretical Chemistry, University of Vienna, Währinger Strasse 17, 1090 Vienna, Austria

## Abstract

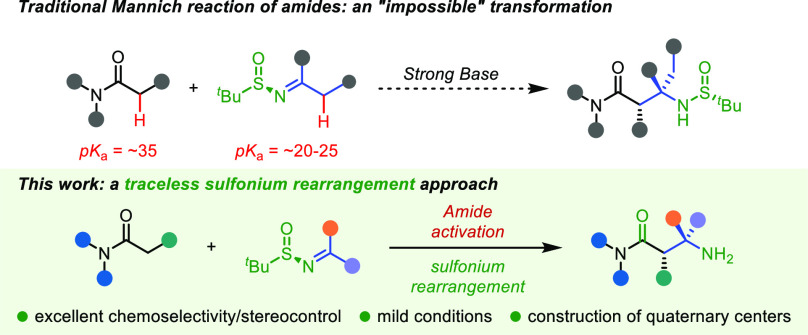

β-Amino acid derivatives are key structural elements in synthetic and biological chemistry. Despite being a hallmark method for their preparation, the direct Mannich reaction encounters significant challenges when carboxylic acid derivatives are employed. Indeed, not only is chemoselective enolate formation a pitfall (particularly with carboxamides), but most importantly the inability to reliably access α-tertiary amines through an enolate/ketimine coupling is an unsolved problem of this century-old reaction. Herein, we report a strategy enabling the first direct coupling of carboxamides with ketimines for the diastereo- and enantioselective synthesis of β-amino amides. This conceptually novel approach hinges on the innovative deployment of enantiopure sulfinimines in sulfonium rearrangements, and at once solves the problems of chemoselectivity, reactivity, and (relative and absolute) stereoselectivity of the Mannich process. In-depth computational studies explain the observed, unexpected (dia)stereoselectivity and showcase the key role of intramolecular interactions, including London dispersion, for the accurate description of the reaction mechanism.

β-Amino acids are privileged structural motifs in natural products^1^ and indispensable building blocks in medicinal chemistry and chemical biology (e.g., for the synthesis of β-peptides^2a^ or β-lactam antibiotics^2b,2c^). These highly sought-after properties have resulted in a long-standing interest in methods for the synthesis of β-amino acids and amides.^3^ Methods for the preparation of β-amino amides are numerous (Scheme 1A).^4,5^ The century-old Mannich reaction^4^ and conjugate addition^5^ are arguably the most commonly employed approaches, albeit often requiring the use of prefunctionalized, activated starting materials with limited structural flexibility.

**Scheme 1:**
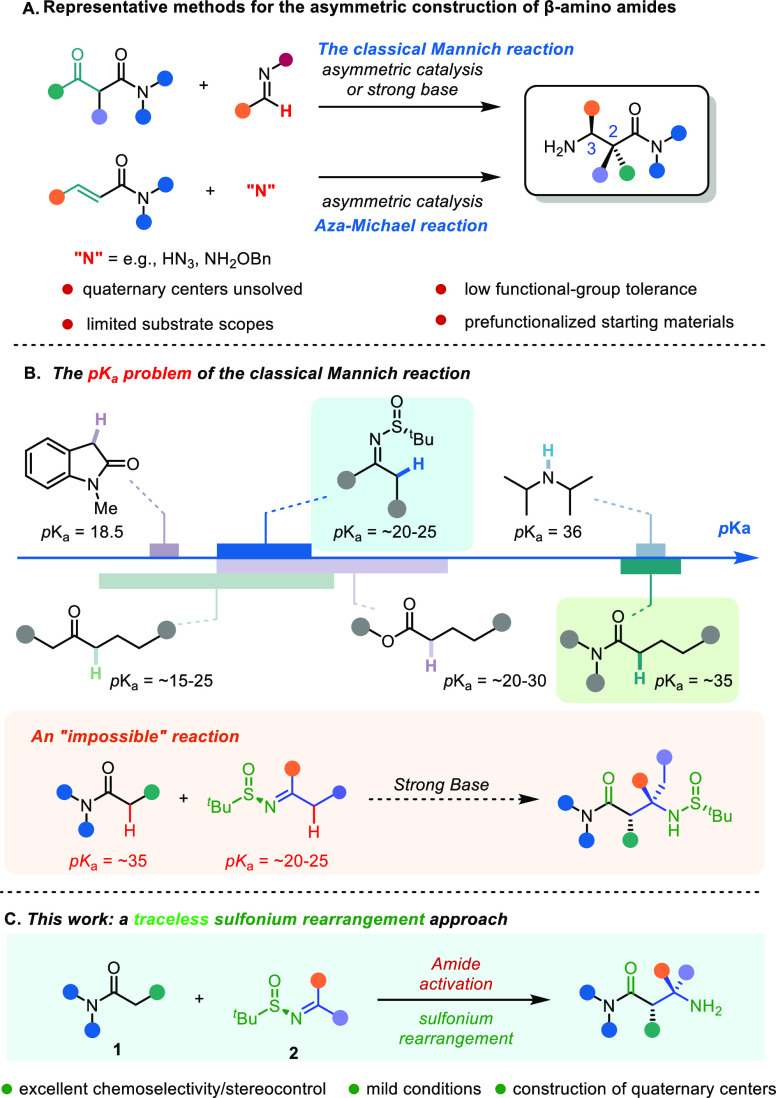
Classical Mannich Reaction and Its Intrinsic Limitations

Although asymmetric Mannich reactions with easily enolizable carbonyl compounds or the corresponding enolate equivalents have been established,^6^ owing to the high α-C–H p*K*_a_ values of amides (p*K*_a_ around 35),^7^ chemoselective enolate formation becomes an almost insurmountable barrier in these cases (Scheme 1B). This renders the classical Mannich reaction a highly challenging prospect for this critical class of donors, and, to this date, successful direct Mannich reactions of carboxamides mostly rely on designer amides bearing a 1-acyl-7-azaindole moiety.^4b,4c,4e^ Recently, the Kobayashi group developed a catalytic system enabling asymmetric Mannich reactions of aldimines with amides.^4h^ However, ketimines were shown to reside outside of the scope of the reaction, as both the steric hindrance and the α-C–H acidity thwarted a general enantioselective route to quaternary stereocenters at C(3). Despite significant advances in the field, the challenges associated with combining poorly C–H acidic carboxamides with readily enolizable ketimines appear to render their efficient direct Mannich coupling nearly impossible.

Enantioenriched organosulfur compounds have emerged as reagents of choice for stereoselective synthesis through chiral propagation.^8^ We herein show that, by synergistically combining enantioenriched sulfinimines in sulfonium rearrangements^9^ with the chemoselectivity of amide activation,^10^ a diastereo- and enantioselective strategy to access acyclic, polysubstituted β-amino amides results (Scheme 1C). This traceless and enantioselective direct coupling effectively solves the problems of chemoselective enolate formation, sluggish reactivity, and competing enolizability of ketimines in Mannich processes.

Our investigations initially focused on the coupling of enantiomerically pure (*R*)-sulfinimine **2a** (readily prepared from (*R*)-*tert*-butylsulfinamide and acetophenone), with (pyrrolidin-1-yl)pentan-1-one (**1a**). Under the optimized reaction conditions (see SI for details, S3), β-amino amide **3a** was formed in 73% yield (major diastereomer) with a d.r. of 10:1 and an e.r. of 99.9:0.1. X-ray crystallographic diffraction of **3a** (CCDC 2153021, S72), produced from an (*R*)-configured sulfinimine, allowed establishment of the (*S*,*S*)-configuration at the newly generated stereocenters of the product.

Turning our attention to the substrate scope (Table 1), we initially probed variations of the amide carbon chain and found that side chains of varying lengths were transformed with very good levels of enantioselectivity (**3b**, **3c**). The reaction displayed good functional-group tolerance: reactive handles such as halides **3d**, nitriles **3e**, ethers **3f**, imides **3g**, alkenes **3j**, and alkynes **3k** were all tolerated under the reaction conditions. Additionally, the β-amino amides **3h** and **3i**, bearing ester and ketone functionality, were successfully obtained in good yields and selectivities and showcase the exceptional chemoselectivity of electrophilic amide activation.^10^ Notably, this unusual chemoselectivity between amides and other carbonyls cannot be achieved by classical Mannich reaction protocols, as those are controlled by α-C–H p*K*_a_ values. Whereas, in the absence of external nucleophiles, amides **1k** have been previously shown to readily undergo intramolecular lactone formation/rearrangement at high temperatures,^11^ this room-temperature Mannich surrogate protocol remarkably overrides the intramolecular reaction.

**Table 1:**
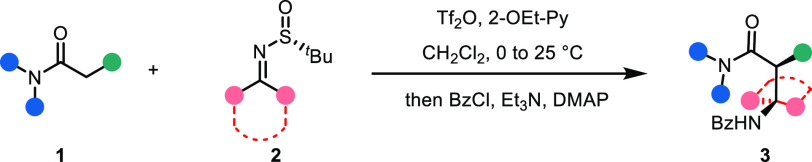

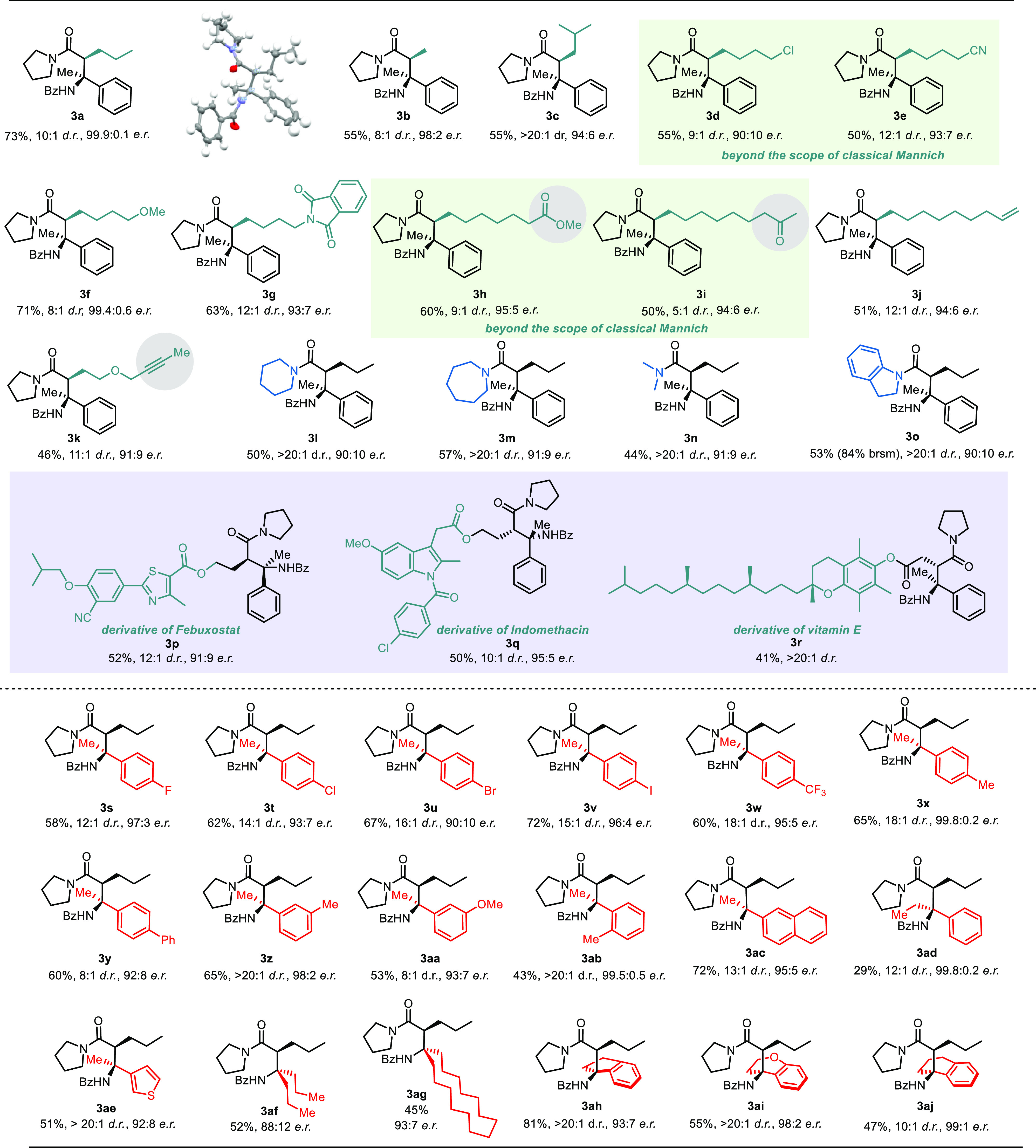
Scope of the Reaction^a^

a Reactions were performed on 0.2 mmol scale. Isolated yields of the major diastereomers are reported. Diastereomeric ratios (d.r.) were determined by ^1^H NMR analysis of the crude product. Enantiomeric ratios (e.r.) determined by HPLC.

Varying substitution at the amide nitrogen was also tolerated, and several tertiary amides were transformed to the corresponding β-amino amides, including those derived from piperidine **3l**, azepine **3m**, dimethylamine **3n**, and indoline **3o**. The established protocol also enabled formation of β-amino amides derived from Febuxostat **3p**, Indomethacin **3q**, and vitamin E **3r**.

Various sulfinimines were then prepared and employed as the reaction partners of amide **1a**, as shown in Table 1. A large degree of flexibility was found with regard to the nature of the aromatic group attached to the sulfinimine. Different halogens were well tolerated (**3s**–**3v**), as were derivatives bearing both electron-withdrawing (**3w**) and electron-donating (**3x**–**3aa**) substituents. Reaction of a sulfinimine endowed with an *ortho*-substituted aryl group also provided the desired product (**3ab**)—it is worth mentioning that the steric influence exerted by the presence of this *ortho*-substituent positively affects the stereoselectivity of the transformation. Other sterically demanding sulfinimines, prepared from 1-(naphthalen-2-yl)ethan-1-one and propiophenone, were also found to furnish the desired β-amino amides **3ac** and **3ad**. While the added steric hindrance of the sulfinimine derived from propiophenone led to outstanding enantioselectivity, a significant decrease of the yield was also observed (**3ad**). Sulfinimines derived from dialkyl ketones were also employed, giving moderate yields and good enantioselectivities (**3af**, **3ag**). The transformation also tolerates sulfinimines prepared from bicyclic ketones such as 1-indanone (**3ah**), 4-chromanone (**3ai**), 1-tetralone (**3aj**), as well as heterocyclic moieties (**3ae**).

Aiming for a direct comparison with classical Mannich protocols employing sulfinimines, we performed an experiment under reaction conditions developed by the Ellman group (Scheme 2A).^12,13^ Despite the reported success for ester-based Mannich reactions, the desired β-amino amide **4** was not detected. This further underlines the unique character and orthogonality of the coupling reaction presented herein.

**Scheme 2:**
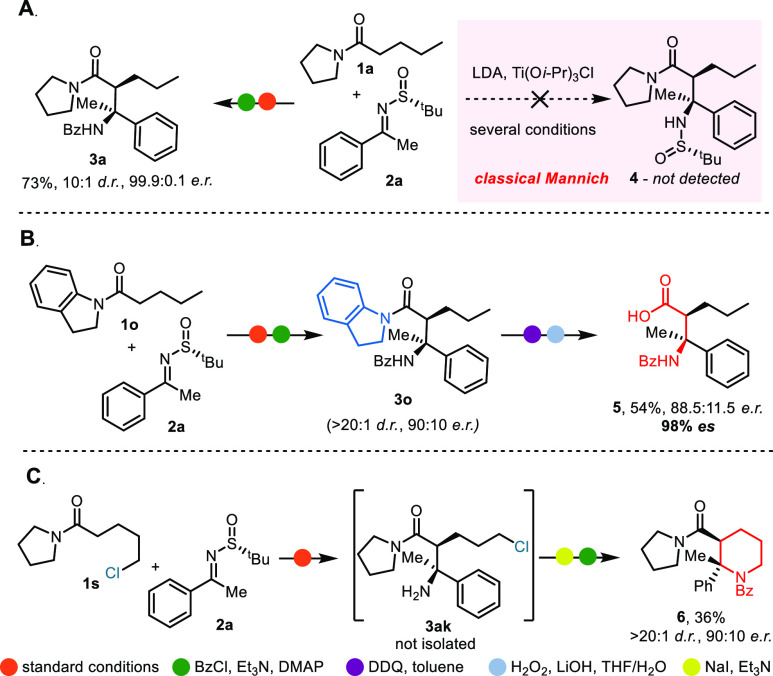
Comparison with the Classical Mannich Approach and Postreaction Functionalizations Reactions were performed on 0.2 mmol scale. Isolated yields of the major diastereomers are reported.

The products lend themselves to rapid derivatization (Scheme 2B,C). Cleavage of **3o**, bearing an indoline amide, can easily be achieved through oxidative conversion to the corresponding indole analogue using DDQ,^14,15^ and subsequent hydrolysis, affording β-amino acid **5** in 54% yield (from **3o**) with 98% enantiospecificity. The presence of two contiguous stereocenters crafted with high stereoselectivity can be harnessed for the synthesis of enantioenriched piperidines (such as **6**) with challenging substitution patterns. The sequence shown proceeded smoothly to give the desired product in 36% overall yield with excellent stereocontrol (>20:1 d.r., 90:10 e.r.).

Aiming to pinpoint the intricacies of this unprecedented process, we adopted a combined experimental/computational approach. Subjecting an ^18^O-labeled amide to the standard conditions led to exclusive formation of nonlabeled product, suggesting that the sulfinimine acts as oxygen donor (SI, S66).^16^

Our quantum chemical calculations (Scheme 3), based on precedent and the observations described above, assumed initial formation of a keteniminium intermediate **A** (Scheme 3A),^17^ which was used as the starting point. The computed Gibbs free energy profile is depicted in Scheme 3A. As shown, intermediate **B** is formed by O-addition of the sulfinimine to the keteniminium species, giving two possible double-bond isomers **B_Z** (*cis*) and **B_E** (*trans*). Both intermediates are formed reversibly (endergonic step, Δ*G*(**A** → **B_E**) = 4.7 kcal mol^–1^ and Δ*G*(**A** → **B_Z**) = 1.5 kcal mol^–1^), with a slight preference for **B_Z**. Similarly, both intermediates **B** are capable of undergoing the next step, a [3,3]-sigmatropic rearrangement: Concerted S–O bond cleavage and C–C bond formation lead to the intermediates **C_SR** and **C_SS**, ultimately determining the diastereoselectivity.

**Scheme 3:**
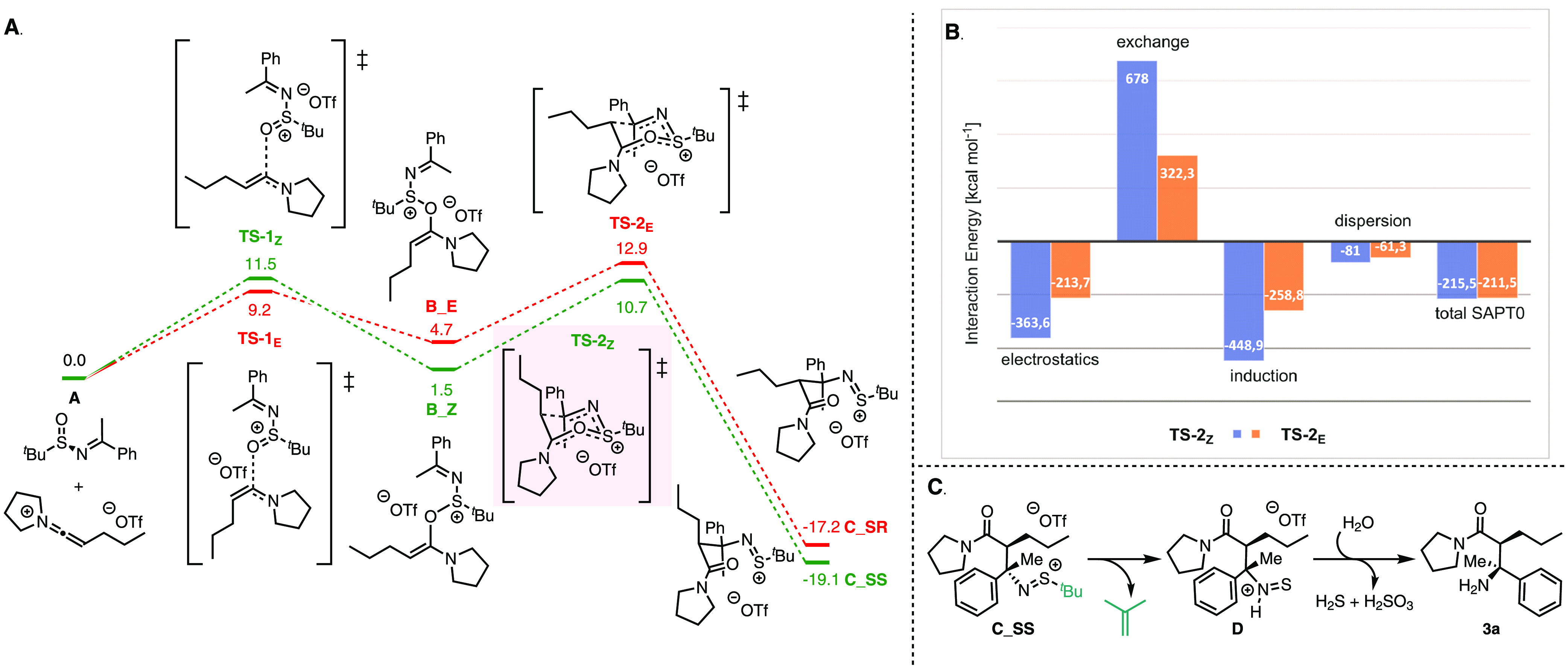
Mechanistic Insight into the Sulfonium Rearrangement (A) Computed reaction profile (DLPNO–CCSD(T)/def2-TZVP//B3LYP-D3(BJ)/def2-SVP, Δ*G*_298, DCM_) for the formation of **C_SR** and **C_SS**. The energy of **A** is taken as a reference (0.0 kcal mol^–1^). (B) SAPT0 analysis of **TS-2**_**Z**_ and **TS-2**_**E**_. Level of theory: SAPT0/jun-cc-pvdz. (C) Hypothetical path for the intramolecular C–S bond cleavage to form **3a**.

Scheme 3A shows that the [3,3]-sigmatropic rearrangement step is highly exergonic for both intermediates **B_E** and **B_Z**, while the formation of **C_SS**, relative to the reference point **A**, is thermodynamically more favorable than formation of **C_SR** (Δ*G*(**A** → **C_SS**) = −19.1 kcal mol^–1^, Δ*G*(**A** → **C_SR**) = −17.2 kcal mol^–1^).

Instead of reacting to the products **C**, intermediates **B_E** and **B_Z** can, however, also revert to the starting state **A** with different degrees of probability: While intermediate **B_E** is more likely to revert to **A** (Δ*G*^‡^(**B_E** → **A**) = 4.5 kcal mol^–1^ and Δ*G*^‡^(**B_E** → **C_SR**) = 8.2 kcal mol^–1^), **B_Z** shows a preference for undergoing the rearrangement to **C_SS** (Δ*G*^‡^(**B_Z** → **A**) = 10.0 kcal mol^–1^ and Δ*G*^‡^(**B_Z** → **C_SS**) = 9.2 kcal mol^–1^). This means that the [3,3]-sigmatropic rearrangement is computed to be kinetically more favorable for the intermediate **B_Z** than for **B_E**, for which the probability of reversion to **A** is relatively high. Ultimately, the favored formation of **C_SS** results from both thermodynamic and kinetic factors: The main reason for the observed diastereoselectivity is, thus, “hidden” in the *Z*/*E* isomerism of the transient intermediate **B**.

Apart from identifying the nature of the individual reaction steps and intermediates, our computational analysis also aided in rationalizing another counterintuitive stereochemical aspect of this transformation: As shown in Table 1, the major diastereomer formed in this process possesses *S*,*S*-configuration. However, this observation is at odds with an expected preference for an all-equatorial chairlike six-membered transition state (**TS-2**_**E**_; boat-like conformations were found to be unfavorable), which would lead to the *S*,*R*-configured product. The relative stability of the chairlike transition state **TS-2**_**Z**_, with the propyl substituent in pseudoaxial orientation, is surprising due to the expected high steric repulsion. In order to deconvolute the underlying reasons for this stability, we conducted an additional computational investigation. Encouraged by recent studies emphasizing the importance of dispersion interactions for the enantioselectivity of organocatalytic processes,^17,18^ we tested the role of these effects for geometry optimization of both transition states, **TS-2**_**E**_ and **TS-2**_**Z**_, comparing results of the B3LYP-D3(BJ) and the B3LYP (without dispersion correction) DFT approaches. Indeed, if dispersion is neglected, a substantial structural distortion is observed for **TS-2**_**Z**_, while for **TS-2**_**E**_, the effect is less significant (SI, S70, Table S3). To further clarify the role of different energetic contributions of the interactions between the sulfinimine and keteniminium fragments in the transition state structures, we performed SAPT0 (Symmetry Adapted Perturbation Theory) energy decomposition analysis (Scheme 3B),^19^ showing the following energy components: electrostatics, exchange, induction, and dispersion. The exchange term (i.e., Pauli repulsion) is large for both transition state structures, showing significant steric repulsion which is, in line with chemical intuition, substantially higher for **TS-2**_**Z**_. However, this is outweighed by the three other terms, leading to an overall preference for **TS-2**_**Z**_. The SAPT analysis therefore clearly shows that the selectivity is not determined by steric repulsion alone: importantly, three other components (induction, electrostatics, and dispersion) are essential for stabilizing **TS-2**_**Z**_, leading to the (*S*,*S*)-configured product.

Next, we sought to elucidate the mechanism of the transformation of **C_SS** into the final β-amino amide product (**3a**). Scheme 3C outlines a computationally suggested intramolecular C–S bond cleavage with the formation of isobutene as a side product (the computed free energy profile is shown in the SI, S70 (Figure S1)). It is noteworthy that this side product was also experimentally detected by in-situ NMR analysis (SI, S61). The calculations further predict a barrierless ion-pair collapse of **D**, forming the product after further N–S bond cleavage. Hypothetically other nucleophiles, e.g., water, can also attack the transient intermediate **D**, ultimately leading to the final product as proposed in Scheme 3C. Importantly, the formation of a 1,3-dicarbonyl product^16^ from the coupling of an aldimine can also be readily rationalized by this mechanistic proposal via loss of the H substituent through “imine-enamine” tautomerization in **C_SS**.

In conclusion, we have reported a conceptually novel approach to the century-old Mannich reaction. This approach hinges on the unprecedented deployment of readily available enantioenriched sulfinamides in a sulfonium rearrangement. These reagents serve as the source of both nitrogen and chiral information, and the obtained β-amino amides carry two contiguous stereogenic centers, including a fully substituted carbon, formed with high levels of diastereo- and enantioselectivity. Detailed computational studies reveal the intricacies of the process, including counterintuitive transition states, and emphasize the insufficiency of typical “chemical intuition”-based approaches that rely primarily on estimated steric repulsion. Most strikingly, this transformation constitutes an apparent direct Mannich coupling of two reaction partners that, paradoxically, cannot be coupled by a classical Mannich transform. The chemistry presented herein decisively solves the challenging problems of chemo-, diastereo-, and enantioselectivity that are intrinsic to the classical Mannich reaction and highlights the power of sulfonium rearrangements for stereoselective C–C bond formation in contemporary synthesis.

## Supporting Information Available

Experimental procedures; spectroscopic and X-ray data (PDF)
Supporting data (XYZ)

## Supplementary Material

ja2c05368_si_001.pdf

ja2c05368_si_002.xyz
