# Supplementary material for: Deployment of Sulfinimines in Charge-Accelerated Sulfonium Rearrangement Enables a Surrogate Asymmetric Mannich Reaction
Source: J Am Chem Soc. 2022 Jul 15;144(29):13044–9. doi: 10.1021/jacs.2c05368 (PMC9374180; doi:10.1021/jacs.2c05368)
Supplement: Supplementary file 1 — ja2c05368_si_001.pdf [file ja2c05368_si_001.pdf]

Supplementary Information

Deployment of Sulfinimines in Charge-Accelerated Sulfonium Rearrangement Enables a Surrogate Asymmetric Mannich Reaction

Minghao Feng, Ivan Mosiagin, Daniel Kaiser, Boris Maryasin* and Nuno Maulide*

Contents

1. **General Information** 2
2. **Optimization** 3
3. **Substrate preparation** 5
   3.1 **Preparation of amides** 5
   3.2 **Characterizations of the prepared amides** 6
   3.3 **Preparation of sulfinimines** 12
   3.4 **Characterizations of the prepared sulfinimines** 12
4. **β-Amino amide synthesis** 20
   4.1 **General procedure for the synthesis of β-amino amides** 20
   4.2 **Characterization of the prepared β-amino amides** 21
5. **Comparison to conventional Mannich reaction and synthetic applications** 57
   5.1 **Comparison to conventional Mannich reaction** 57
   5.2 **Preparation of β-amino acid 5 from indoline amide 3o** 57
   5.3 **Synthesis of the piperidine derivative 6** 58
6. **Limitations** 61
7. ***In situ* NMR studies of the reaction** 61
8. **^{18}O-labeling experiment** 66
9. **Reaction with aldimine** 68
10. **Gram-scale reaction** 68
11. **Computational Details** 70
12. **X-Ray Crystallographic data for compound 3a** 72
13. **NMR spectra** 75
14. **References** 125

1. General Information

Unless otherwise stated, all glassware was flame-dried before use and all reactions were performed under an atmosphere of argon. All solvents were distilled from appropriate drying agents prior to use or, if purchased in anhydrous form, used as received from commercial suppliers. Triflic anhydride was distilled over P_4O_{10} prior to use. All other reagents were used as received from commercial suppliers, unless otherwise stated. Reaction progress was monitored by thin layer chromatography (TLC) performed on aluminium plates coated with silica gel F_{254} with 0.2 mm thickness. Chromatograms were visualized by fluorescence quenching with UV light at 254 nm or by staining using potassium permanganate. Flash column chromatography was performed using silica gel 60 (230-400 mesh, Merck and co.). Neat infra-red spectra were recorded using a Perkin-Elmer Spectrum 100 FT-IR spectrometer. Wavenumbers (vmax) are reported in cm^{-1}. Mass spectra were obtained using a Finnigan MAT 8200 or (70 eV) or an Agilent 5973 (70 eV) spectrometer, using electrospray ionization (ESI). All ^{1}H NMR, ^{13}C NMR and ^{19}F NMR spectra were recorded using a Bruker AV-400, AV-600 or AV-700 spectrometer at 300K. Chemical shifts are given in parts per million (ppm, δ), referenced to the solvent peak of $CDCl_3$, defined at δ = 7.26 ppm (^{1}H NMR) and δ = 77.16 (^{13}C NMR). Coupling constants are quoted in Hz (*J*). ^{1}H NMR splitting patterns were designated as singlet (s), doublet (d), triplet (t), quartet (q), pentet (p). Splitting patterns that could not be interpreted or easily visualized were designated as multiplet (m) or broad (br). Selected ^{13}C NMR spectra were recorded using the attached proton test (APT) to facilitate the confirmation and assignment of the structure. Optical rotations were measured on a Unipol L 2000 polarimeter using a 100 mm path-length cell at 589 nm (c given in g/100 mL).

2. Optimization

Supplementary Table 1 | Optimization of the solvent [a]

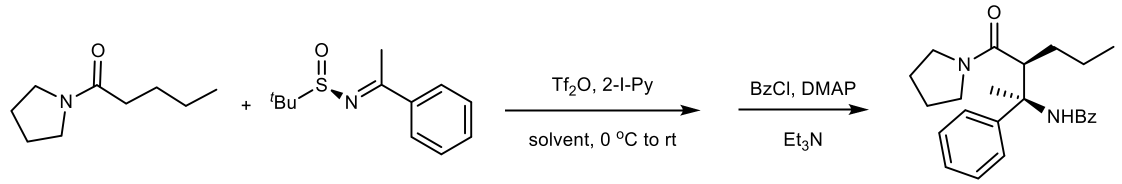

Entry	Solvent	Isolated yieds[b]	*d.r.*[c]	*ee*[d]
**1**	CH_2Cl_2	66%	10: 1	99%
**2**	MeCN	55%	9: 1	98%
**3**	THF	trace	-	-
**4**	DCE	59%	10:1	99%
**5**	dioxane	57%	5:1	88%
**6**	$MeNO_2$	trace	-	-
**7**	$CHCl_3$	55%	9:1	85%
**8**	DMF	No reaction	-	-
**9**	toluene	trace	-	-

[a] To a mixture of amide (31 mg, 0.20 mmol, 1.0 equiv.) and 2-iodopyridine (0.44 mmol) in the given solvent (1 mL) in a flame-dried Schlenk tube under argon at 0 °C, triflic anhydride (40 µL, 0.24 mmol, 1.20 equiv.) was added dropwise. After stirring for 30 min at 0 °C, sulfinimine (89 mg, 0.40 mmol, 2.00 equiv.) in 1 mL of solvent was added and the reaction stirred for a further 5 min at 0 °C. After stirring for 24 h at room temperature, triethylamine (139 µL, 1.00 mmol, 5.00 equiv.), 4-dimethylaminopyridine (5 mg, 0.04 mmol, 20 mol%) and benzoyl chloride (116 µL, 1.00 mmol, 5.00 equiv.) were added to the reaction. After stirring for another 12 h at the same temperature, the reaction was basified by the addition of a saturated aqueous solution of sodium bicarbonate (5 mL) and subsequently extracted twice with CH_2Cl_2 (5 mL). The combined organic layers were dried over anhydrous magnesium sulfate and the dried solution was filtered and subsequently concentrated under reduced pressure. The resulting crude product was purified by flash column chromatography on silica gel. [b] Isolated yield of the major diastereomer. [c] The diastereomeric ratio (*d.r.*) was determined by ^{1}HNMR analysis of the crude material. [d] Enantiomeric excess (*ee*) was determined using high-performance liquid chromatography (HPLC).

Supplementary Table 2 | Optimization of the base [a]

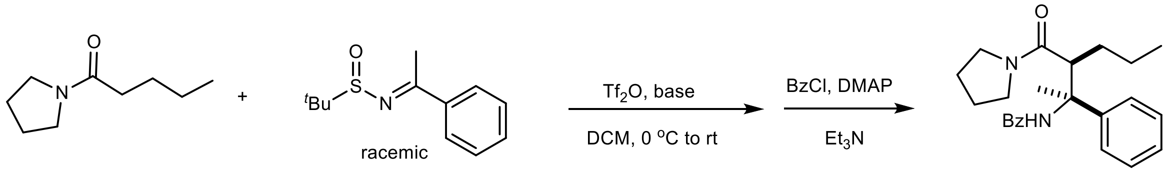

Entry	Base	Isolated yieds[b]	*d.r.*[c]
**1**	2-I-Py	66%	10:1
**2**	2,4,6-trimethylpyridine	trace	
**3**	2-F-Py	35%	10:1
**4**	2-Cl-Py	41%	10:1
**5**	2-OMe-Py	67%	10:1
**6**	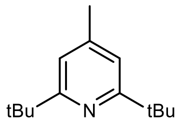	trace	
**7**	quinoline	29%	9:1
**8**	2-Ph-Py	36%	7:1
**9**	2-Me-Py	50%	10:1
10	***2-OEt-Py***	***73%***	***10:1***
**11[d]**	2-OEt-Py	55%	10:1
**12[e]**	2-OEt-Py	64%	10:1

[a] To a mixture of amide (31 mg, 0.20 mmol, 1.00 equiv.) and the given base (0.44 mmol) in CH_2Cl_2 (1 mL) in a flame-dried Schlenk tube under argon at 0 °C, triflic anhydride (40 µL, 0.24 mmol, 1.20 equiv.) was added dropwise. After stirring for 30 min at 0 °C, sulfinimine (89 mg, 0.40 mmol, 2.00 equiv.) in 1 mL of solvent was added and the reaction stirred for a further 5 min at 0 °C. After stirring for 24 h at room temperature, triethylamine (139 µL, 1.00 mmol, 5.00 equiv.), 4-dimethylaminopyridine (5 mg, 0.04 mmol, 20 mol%) and benzoyl chloride (116 µL, 1.00 mmol, 5.00 equiv.) were added to the reaction. After stirring for another 12 h at the same temperature, the reaction was basified by the addition of a saturated aqueous solution of sodium bicarbonate (5 mL) and subsequently extracted twice with CH_2Cl_2 (5 mL). The combined organic layers were dried over anhydrous magnesium sulfate and the dried solution was filtered and subsequently concentrated under reduced pressure. The resulting crude product was purified by flash column chromatography on silica gel. [b] Isolated yield of the major diastereomer isomer. [c] The diastereomeric ratio (*d.r.*) was determined by ^{1}HNMR analysis of the crude material. [d] 3.0 equivalents of sulfonimine were used. [e] the solution of sulfonimine was added in dropwise over the course of 1 h.

Note: the optimized procedure was then applied to enantioenriched sulfinimine, the enantiomeric ratio of the desired product was determined to be 99.9:0.1 by chiral HPLC analysis.

3. Substrate preparation

3.1 Preparation of amides

General Procedure A:

To a solution of the amine (1.00 equiv.) and triethylamine (2.00 equiv.) in CH_2Cl_2 (0.1 M) at 0 °C, the corresponding acyl chloride (1.20 equiv.) was added dropwise and the resulting reaction mixture was allowed to warm to room temperature while stirring overnight (14 h). After this time, a saturated aqueous solution of sodium bicarbonate was added and the biphasic system was separated. The aqueous phase was extracted with CH_2Cl_2 (three times) and the organic phases were combined and dried over anhydrous sodium sulfate. The dried solution was filtered and concentrated under reduced pressure. The resulting crude material was purified by flash column chromatography on silica gel (heptane/ethyl acetate) to afford the desired compound.

General Procedure B:

To a solution of the carboxylic acid (1.00 equiv.), triethylamine (1.00 equiv.), 4-dimethylaminopyridine (DMAP, 10 mol%) and 1-ethyl-3-(3-dimethylaminopropyl)carbodiimide hydrochloride (EDCI·HCl, 1.50 equiv.) in CH_2Cl_2 (0.1 M), the corresponding amine (1.20 equiv.) was added and the resulting solution was stirred at room temperature overnight (14 h). After this time, the organic solution was extracted sequentially with 0.5 M aqueous hydrochloric acid, saturated aqueous sodium bicarbonate and saturated aqueous sodium chloride. The washed solution was dried over anhydrous sodium sulfate, filtered and concentrated under reduced pressure. The resulting crude material was purified by flash column chromatography on silica gel (heptane/ethyl acetate) to afford the desired compound.

3.2 Characterizations of the prepared amides

1-(Pyrrolidin-1-yl)pentan-1-one (1a)

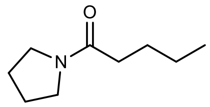

The title compound was obtained in 98% yield using General Procedure A. All analytical data were in good accordance with data reported in the literature.[1]

1-(Pyrrolidin-1-yl)propan-1-one (1b)

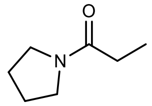

The title compound was obtained in 97% yield using General Procedure A. All analytical data were in good accordance with data reported in the literature.[2]

4-Methyl-1-(pyrrolidin-1-yl)pentan-1-one (1c)

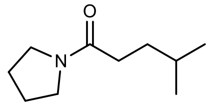

The title compound was obtained in 92% yield using General Procedure B. All analytical data were in good accordance with data reported in the literature.[3]

6-Chloro-1-(Pyrrolidin-1-yl)hexan-1-one (1d)

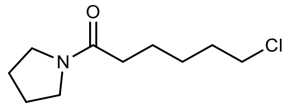

The title compound was obtained in 92% yield using General Procedure B. All analytical data were in good accordance with data reported in the literature.[4]

7-Oxo-7-(pyrrolidin-1-yl)heptanenitrile (1e)

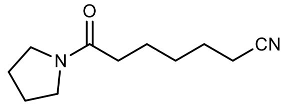

To a room-temperature solution of sodium cyanide (1.40 equiv.) in DMSO (1.5 M) was added 6-chloro-1-(pyrrolidin-1-yl)hexan-1-one (S1e, 1.00 equiv.) and the resulting mixture was heated to 120 °C for 1 h. After cooling, the solvent was evaporated under high vaccum, affording a mixture of the product and excess sodium cyanide. *Notice: sodium cyanide is a poison. All the waste of the reaction should be kept in basic media.* Direct flash column chromatography on silica gel (0 to 20% DMA/ CH_2Cl_2, DMA solution is prepared from CH_2Cl_2/methanol/ammonia = 90:9:1) afforded the pure amide (77%). All analytical data were in good accordance with data reported in the literature.[4]

6-Methoxy-1-(pyrrolidin-1-yl)hexan-1-one (1f)

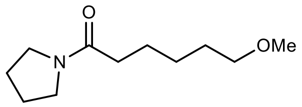

The title compound was obtained in 90% yield using General Procedure B. All analytical data were in good accordance with data reported in the literature. [5]

2-(6-Oxo-6-(pyrrolidin-1-yl)hexyl)isoindoline-1,3-dione (1g)

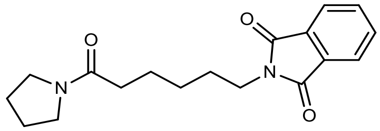

To a solution of potassium phthalimide (1.40 equiv.) in DMSO (1.5 M) was added 6-chloro-1-(pyrrolidin-1-yl)hexan-1-one (S1e, 1.00 equiv.) and the resulting mixture was heated to 120 °C for 1 h. After cooling, the solvent was evaporated under high vaccum. Direct flash column chromatography on silica gel gel (0 to 20% DMA/ CH_2Cl_2, DMA solution is prepared from CH_2Cl_2/methanol/ammonia = 90:9:1) afforded the pure amide (57%). All analytical data were in good accordance with data reported in the literature.[6]

Methyl 9-oxo-9-(pyrrolidin-1-yl)nonanoate (1h)

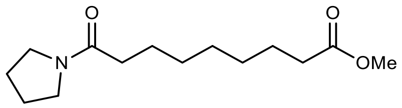

The title compound was obtained in 85% yield using General Procedure B. All analytical data were in good accordance with data reported in the literature.[4]

1-(Pyrrolidin-1-yl)undecane-1,10-dione (1i)

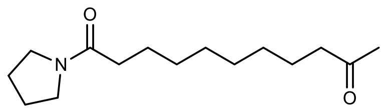

To a solution of amide (1 equiv.) and $Pd(OAc)_2$ (0.10 equiv.) in DMSO/water (0.33 M, 10/1, v/v) in a Schlenk tube under oxygen atmosphere was added TFA (1.00 equiv.) and the resulting mixture was heated to 70 °C overnight (14 h). After cooling to ambient temperature, the reaction mixture was diluted with water and extracted with ethyl acetate. The organic solution was then washed with brine, dried over Na_2SO_4, filtered and concentrated under reduced pressure. Flash column chromatography on silica gel (0 to 20% DMA/ CH_2Cl_2, DMA solution is prepared from CH_2Cl_2/methanol/ammonia = 90:9:1) afforded the pure desired amide (30%) as well as 66% unreacted starting material. All analytical data were in good accordance with data reported in the literature.[4]

1-(Pyrrolidin-1-yl)undec-10-en-1-one (1j)

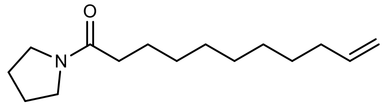

The title compound was obtained in 94% yield using General Procedure A. All analytical data were in good accordance with data reported in the literature.[7]

4-Hydroxy-1-(pyrrolidin-1-yl)butan-1-one (S1)

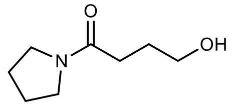

A mixture of γ-butyrolactone (2.3 mL, 30 mmol, 1.00 equiv.), pyrrolidine (60 mmol, 4.9 mL, 2.00 equiv.) and triethylamine (120 mmol, 16.8 mL, 4.00 equiv.) was heated at reflux (90 °C) overnight (14 h). The mixture was concentrated in vacuo to yield the title compound (quantitative yield). The product was deemed pure enough for further transformation without additional purification. All analytical data were in good accordance with data reported in the literature.[8]

4-Hydroxy-1-(pyrrolidin-1-yl)butan-1-one (1k)

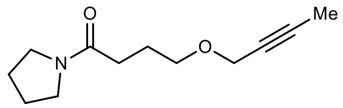

To a solution of 4-hydroxy-1-(pyrrolidin-1-yl)butan-1-one (**S1**, 1.00 equiv.) in THF (0.5 M) at 0 °C was added sodium hydride (1.20 equiv., 60% suspension in mineral oil) in small portions. After stirring for 1 h at 0 °C, 1-bromobut-2-yne (1.20 equiv.) was added dropwise and the reaction mixture was brought to room temperature and subsequently stirred overnight (14 h). Excess base was slowly quenched at 0 °C using a

saturated aqueous solution of ammonium chloride. The aqueous phase was separated and extracted with ethyl acetate. The combined organic layers were dried over anhydrous sodium sulfate, filtered, and concentrated in vacuo. Flash column chromatography on silica gel (heptane/ethyl acetate) afforded the desired product in 65% yield. All analytical data were in good accordance with data reported in the literature.[8]

1-(Piperidin-1-yl)pentan-1-one (1l)

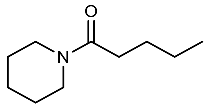

The title compound was obtained in 97% yield using General Procedure A. All analytical data were in good accordance with data reported in in the literature. [9]

1-(Piperidin-1-yl)pentan-1-one (1m)

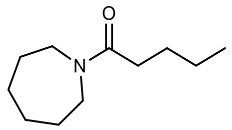

The title compound was obtained in 95% yield (174 mg, 0.95 mmol) using General Procedure A (heptane/ethyl acetate 1:2, Rf = 0.28).

^{1}H NMR (400 MHz, $CDCl_3$) δ 3.57 – 3.48 (m, 2H), 3.46 – 3.39 (m, 2H), 2.34 – 2.27 (m, 2H), 1.76 – 1.51 (m, 10H), 1.36 (dq, *J* = 14.7, 7.4 Hz, 2H), 0.93 (t, *J* = 7.3 Hz, 3H).

^{13}C NMR (100 MHz, $CDCl_3$) δ 172.8, 47.9, 45.9, 33.0, 29.2, 27.6, 27.6, 27.1, 26.8, 22.7, 13.9.

IR (neat) ν: 2927, 2857, 1638, 1423, 1376, 1194, 1169, 1101 cm^{-1}.

HRMS (ESI$^+$): exact mass calculated for $[M+H]^+$ ($C_{11}H_{22}NO$) requires m/z 184.1696, found m/z 184.1694.

***N,N*-Dimethylpentanamide (1n)**

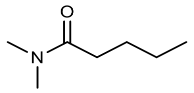

The title compound was obtained in 99% yield using General Procedure A. All analytical data were in good accordance with data reported in in the literature.[10]

1-(Indolin-1-yl)pentan-1-one (1o)

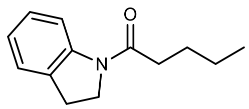

The title compound was obtained in 92% yield (187 mg, 0.92 mmol) using General Procedure A (heptane/ethyl acetate 3:1, Rf = 0.35). The obtained product was further purified by recrystallization from CH_2Cl_2/heptane.

^{1}H NMR (400 MHz, $CDCl_3$) δ 8.24 (d, *J* = 8.0 Hz, 1H), 7.16 (dd, *J* = 13.6, 6.1 Hz, 2H), 6.98 (t, *J* = 7.4 Hz, 1H), 4.01 (t, *J* = 8.3 Hz, 2H), 3.16 (t, *J* = 8.4 Hz, 2H), 2.39 (t, *J* = 7.4 Hz, 2H), 1.77 – 1.64 (m, 2H), 1.49 – 1.35 (m, 2H), 0.96 (t, *J* = 7.3 Hz, 3H).

^{13}C NMR (100 MHz, $CDCl_3$) δ 171.4, 143.1, 131.0, 127.5, 124.4, 123.4, 117.0, 47.9, 35.6, 28.0, 26.6, 22.5, 13.9.

IR (neat) ν: 2954, 2926, 2867, 1656, 1598, 1480, 1458, 1413, 1365, 1308, 1290, 1257, 1119, 761 cm^{-1}.

HRMS (ESI$^+$): exact mass calculated for $[M+H]^+$ ($C_{13}H_{18}NO$) requires m/z 204.1383, found m/z 204.1380.

4-Oxo-4-(pyrrolidin-1-yl)butyl 2-(3-cyano-4-isobutoxyphenyl)-4-methylthiazole-5-carboxylate (1p)

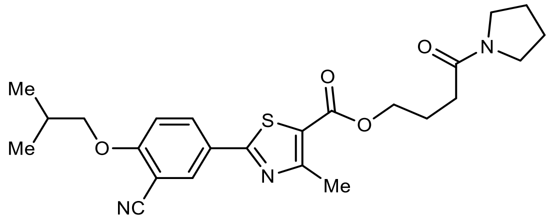

To a solution of Febuxostat (1.00 equiv.), triethylamine (1.00 equiv.), 4-dimethylaminopyridine (DMAP, 10 mol%) and 1-ethyl-3-(3-dimethylaminopropyl)carbodiimide hydrochloride (EDCI·HCl, 1.50 equiv.) in CH_2Cl_2 (0.1 M), 4-hydroxy-1-(pyrrolidin-1-yl)butan-1-one (**S1l**, 1.20 equiv.) was added and the resulting solution was stirred at room temperature overnight (14 h). After this time, the organic solution was extracted sequentially with 0.5 M aqueous hydrochloric acid, saturated aqueous sodium bicarbonate and saturated aqueous sodium chloride. The washed solution was dried over anhydrous sodium sulfate, filtered and concentrated under reduced pressure. The resulting crude material was purified by flash column chromatography on silica gel (heptane/ethyl acetate 1:2, Rf = 0.42) to afford the title compound in 80% yield (364 mg, 0.8 mmol).

^{1}H NMR (700 MHz, $CDCl_3$) δ 8.17 (d, *J* = 2.2 Hz, 1H), 8.12 (dd, *J* = 8.8, 2.2 Hz, 1H), 7.02 (d, *J* = 8.9 Hz, 1H), 4.37 (t, *J* = 6.3 Hz, 2H), 3.90 (d, *J* = 6.5 Hz, 2H), 3.45 (d, *J* = 30.1 Hz, 4H), 2.77 (s, 3H), 2.41 (t, *J* = 7.3 Hz, 2H), 2.20 (dt, *J* = 13.3, 6.7 Hz, 1H), 2.14 (t, *J* = 6.7 Hz, 2H), 1.96 (m, 2H), 1.87 (m, 2H), 1.09 (d, *J* = 6.7 Hz, 6H).

^{13}C NMR (175 MHz, $CDCl_3$) δ 170.2, 167.3, 162.6, 161.9, 161.1, 132.6, 132.2, 125.88, 121.7, 115.4, 112.7, 103.0, 75.7, 64.9, 46.6, 45.7, 30.9, 28.1, 26.1, 24.4, 24.0, 19.0 (2C), 17.4.

IR (neat) ν: 2962, 2874, 2227, 1711, 1639, 1508, 1434, 1389, 1371, 1328, 1262, 1170, 1104, 1045, 1012, 760 cm^{-1}.

HRMS (ESI$^+$): exact mass calculated for $[M+H]^+$ ($C_{24}H_{30}N_3O_4S$) requires m/z 456.1952, found m/z 456.1953.

4-Oxo-4-(pyrrolidin-1-yl)butyl 2-(1-(4-chlorobenzoyl)-5-methoxy-2-methyl-1H-indol-3-yl)acetate (1q)

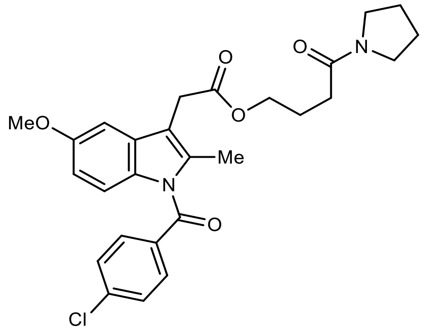

To a solution of Indometacin (1.00 equiv.), triethylamine (1.00 equiv.), 4-dimethylaminopyridine (DMAP, 10 mol%) and 1-ethyl-3-(3-dimethylaminopropyl)carbodiimide hydrochloride (EDCI·HCl, 1.50 equiv.) in CH_2Cl_2 (0.1 M), 4-hydroxy-1-(pyrrolidin-1-yl)butan-1-one (**S1l**, 1.20 equiv.) was added and the resulting solution was stirred at room temperature overnight (14 h). After this time, the organic solution was extracted sequentially with 0.5 M aqueous hydrochloric acid, saturated aqueous sodium bicarbonate and saturated aqueous sodium chloride. The washed solution was dried over anhydrous sodium sulfate, filtered and concentrated under reduced pressure. The resulting crude material was purified by flash column chromatography on silica gel (heptane/ethyl acetate 1:2, Rf = 0.52) to afford the title compound in 83% yield (412 mg, 0.83 mmol).

^{1}H NMR (700 MHz, CDCl$_3$) δ 7.64 (d, *J* = 8.5 Hz, 2H), 7.46 (d, *J* = 8.5 Hz, 2H), 6.96 (d, *J* = 2.5 Hz, 1H), 6.88 (d, *J* = 9.0 Hz, 1H), 6.65 (dd, *J* = 9.0, 2.5 Hz, 1H), 4.16 (t, *J* = 6.3 Hz, 2H), 3.81 (s, 3H), 3.65 (s, 2H), 3.40 (t, *J* = 6.9 Hz, 2H), 3.13 (t, *J* = 6.8 Hz, 2H), 2.37 (s, 3H), 2.17 (t, *J* = 7.4 Hz, 2H), 2.00 – 1.94 (m, 2H), 1.88 – 1.84 (m, 2H), 1.83 – 1.78 (m, 2H).

^{13}C NMR (175 MHz, CDCl$_3$) δ 170.7, 170.2, 168.2, 156.0, 139.2, 135.8, 133.8, 131.1 (2C), 130.7, 130.6, 129.1 (2C), 114.9, 112.6, 111.6, 101.2, 64.7, 55.6, 46.2, 45.6, 30.6, 30.4, 26.0, 24.3, 23.9, 13.3.

IR (neat) ν: 2969, 2874, 1732, 1682, 1639, 1478, 1438, 1400, 1358, 1322, 1260, 1166, 1145, 1088, 1068, 1035, 1015, 755, 530 cm^{-1}.

HRMS (ESI$^+$): exact mass calculated for $[M+H]^+$ ($C_{27}H_{30}N_2O_5Cl$) requires m/z 497.1838, found m/z 497.1839.

(*R*)-2,5,7,8-tetramethyl-2-((4*R*,8*R*)-4,8,12-trimethyltridecyl)chroman-6-yl-4-oxo-4-(pyrrolidin-1 yl)butanoate (1r)

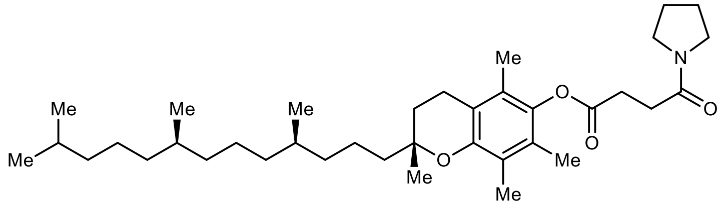

To a solution of D-α-Tocopherol succinate (1.00 equiv.), triethylamine (1.00 equiv.), 4-dimethylaminopyridine (DMAP, 10 mol%) and 1-ethyl-3-(3-dimethylaminopropyl)carbodiimide hydrochloride (EDCI·HCl, 1.50 equiv.) in CH_2Cl_2 (0.1 M), pyrrolidine (1.20 equiv.) was added and the resulting solution was stirred at room temperature overnight (14 h). After this time, the organic solution was extracted sequentially with 0.5 M aqueous hydrochloric acid, saturated aqueous sodium bicarbonate and saturated aqueous sodium chloride. The washed solution was dried over anhydrous sodium sulfate, filtered and concentrated under reduced pressure. The resulting crude material was purified by flash column chromatography on silica gel (heptane/ethyl acetate 1:1, Rf = 0.22) to afford the title compound in 88% yield (513 mg, 0.88 mmol).

^{1}H NMR (400 MHz, $CDCl_3$) δ 3.47 (td, *J* = 6.9, 2.9 Hz, 4H), 3.00 (t, *J* = 6.8 Hz, 2H), 2.68 (t, *J* = 6.8 Hz, 2H), 2.58 (t, *J* = 6.8 Hz, 2H), 2.08 (s, 3H), 2.02 (s, 3H), 2.00 – 1.92 (s+m, 5H), 1.89 – 1.81 (m, 2H), 1.76 (dt, *J* = 20.4, 6.8 Hz, 2H), 1.58 – 1.47 (m, 3H), 1.47 – 1.17 (m, 15H), 1.17 – 1.03 (m, 6H), 0.89 – 0.82 (m, 12H).

^{13}C NMR (100 MHz, $CDCl_3$) δ 172.0, 169.5, 149.3,140.6, 126.8, 125.1, 122.9, 117.3, 75.0, 46.5, 45.8, 39.4 (2C), 37.5 (3C), 37.3, 32.8 (2C), 32.7, 31.1, 29.3, 28.9, 28.0, 26.1, 24.8, 24.5, 24.4, 22.7, 22.6, 21.0, 20.6, 19.8, 19.7, 13.0, 12.1, 11.8.

IR (neat) ν: 2924, 2868, 1753, 1649, 1439, 1365, 1224, 1201, 1143, 1109, 1080, 995, 913, 759, 735 cm^{-1}.

HRMS (ESI$^+$): exact mass calculated for $[M+H]^+$ ($C_{37}H_{62}NO_4$) requires m/z 584.4673, found m/z 584.4671.

3.3 Preparation of sulfinimines

General Procedure C:

A 100 ml two-necked, round-bottomed flask, equipped with an inlet adapter with three-way stopcock, a rubber septum, and a magnetic stirring bar, was charged with (*R*)-*tert*-butanesulfinamide (500 mg, 4.20 mmol, 1.00 equiv.). The flask was evacuated and backfilled with nitrogen. THF (8 mL), $Ti(OEt)_4$ (2.40 ml, 10.4 mmol, 2.70 equiv.), and the corresponding ketone (4.20 mmol, 1.00 equiv.) were sequentially added to the flask, and the resulting solution was stirred at 55 °C. After 14-48 h (monitored by TLC), the reaction mixture was allowed to cool to room temperature, ethyl acetate was added, and the reaction was terminated by the addition of brine. The resulting mixture was stirred for 30 min at room temperature and the insoluble materials were removed by filtration through a pad of Celite. The resulting solution was concentrated under reduced pressure to afford the crude product, which was purified through flash column chromatography on silica gel.

3.4 Characterizations of the prepared sulfinimines

***(R,E)*-2-Methyl-*N*-(1-phenylethylidene)propane-2-sulfinamide (2a)**

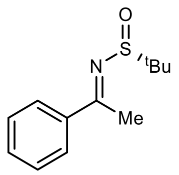

The title compound was obtained in 91% yield using General Procedure C. All analytical data were in good accordance with data reported in in the literature.[13]

***(R,E)-N*-(1-(4-Fluorophenyl)ethylidene)-2-methylpropane-2-sulfinamide (2b)**

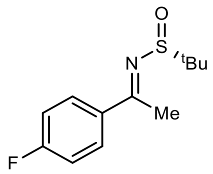

The title compound was obtained in 77% yield using General Procedure C. All analytical data were in good accordance with data reported in in the literature.[14]

***(R,E)-N*-(1-(4-Chlorophenyl)ethylidene)-2-methylpropane-2-sulfinamide (2c)**

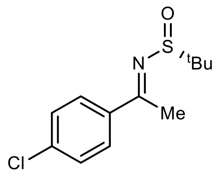

The title compound was obtained in 82% yield using General Procedure C. All analytical data were in good accordance with data reported in in the literature.[15]

***(R,E)-N*-(1-(4-Bromophenyl)ethylidene)-2-methylpropane-2-sulfinamide (2d)**

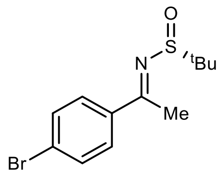

The title compound was obtained in 85% yield using General Procedure C. All analytical data were in good accordance with data reported in in the literature.[16]

***(R,E)-N*-(1-(4-Iodophenyl)ethylidene)-2-methylpropane-2-sulfinamide (2e)**

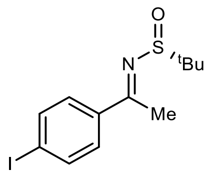

The title compound was obtained in 81% yield (1.19 g, 3.40 mmol) using General Procedure C (heptane/ethyl acetate 1:1, Rf = 0.33).

^{1}H NMR (400 MHz, $CDCl_3$) δ 7.77 (d, *J* = 8.7 Hz, 2H), 7.59 (d, *J* = 8.5 Hz, 2H), 2.73 (s, 3H), 1.31 (s, 9H).

^{13}C NMR (100 MHz, $CDCl_3$) δ 175.4, 138.2, 137.7 (2C), 128.7 (2C), 98.9, 57.6, 22.5 (3C), 19.5.

IR (neat) ν: 2958, 2925, 1716, 1602, 1579, 1554, 1479, 1454, 1389, 1362, 1274, 1223, 1183, 1087, 1069, 1004, 977, 822, 758, 734, 678, 650, 586 cm^{-1}.

HRMS (ESI$^+$): exact mass calculated for $[M+H]^+$ ($C_{12}H_{17}INOS$) requires m/z 350.0070, found m/z 350.0071.

$[\alpha]_D^{20}$ = 1.03 (c = 2.0, $CHCl_3$).

Enantiomeric ratio > 99:1 was determined by chiral HPLC analysis: Chiralpak IC, *n*-heptane+0.1%IPA/IPA 9:1, 1 mL/min, 25 °C, detection at 230 nm, retention time (min): 5.3 (major) and 6.0 (minor).

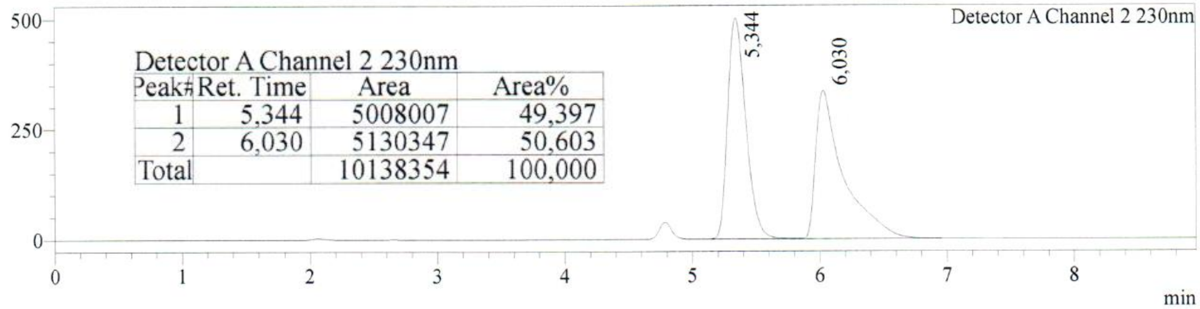

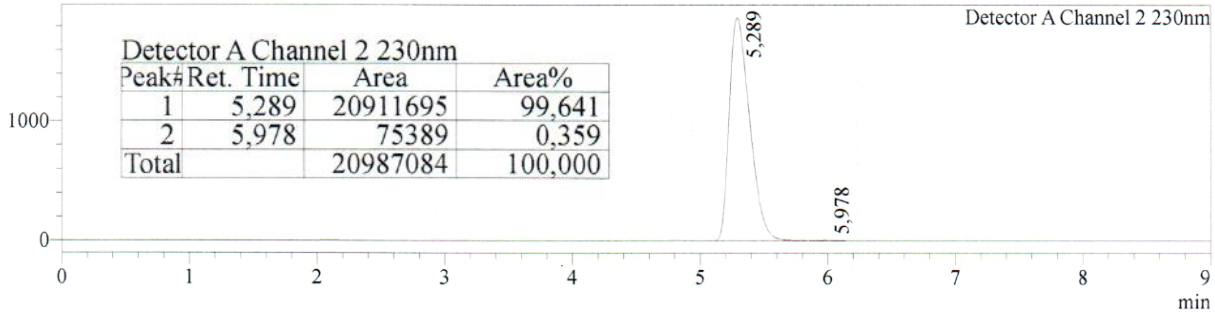

***(R,E)*-2-Methyl-*N*-(1-(4-(trifluoromethyl)phenyl)ethylidene)propane-2-sulfinamide (2f)**

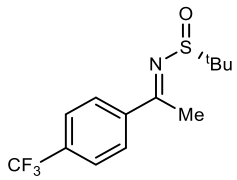

The title compound was obtained in 66% yield using General Procedure C. All analytical data were in good accordance with data reported in in the literature.[17]

(R,E)-2-Methyl-N-(1-(p-tolyl)ethylidene)propane-2-sulfinamide (2g)

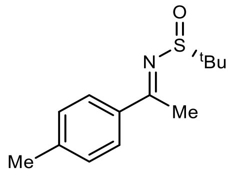

The title compound was obtained in 88% yield using General Procedure C. All analytical data were in good accordance with data reported in in the literature.[18]

(R,E)-N-(1-([1,1'-biphenyl]-4-yl)ethylidene)-2-methylpropane-2-sulfinamide (2h)

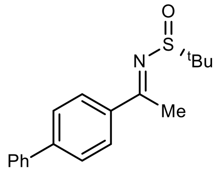

The title compound was obtained in 79% yield using General Procedure C. All analytical data were in good accordance with data reported in in the literature.[19]

(R,E)-2-methyl-N-(1-(m-tolyl)ethylidene)propane-2-sulfinamide (2i)

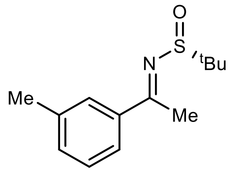

The title compound was obtained in 68% yield using General Procedure C. All analytical data were in good accordance with data reported in in the literature.[18]

(R,E)-N-(1-(3-methoxyphenyl)ethylidene)-2-methylpropane-2-sulfinamide (2j)

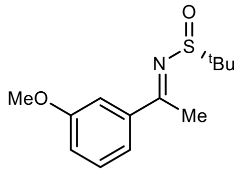

The title compound was obtained in 59% yield using General Procedure C. All analytical data were in good accordance with data reported in in the literature.[20]

(R,E)-2-methyl-N-(1-(m-tolyl)ethylidene)propane-2-sulfinamide (2k)

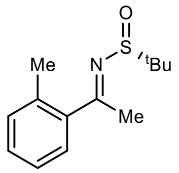

The title compound was obtained in 49% yield using General Procedure C. All analytical data were in good accordance with data reported in in the literature.[21]

(R,E)-2-methyl-N-(1-(naphthalen-2-yl)ethylidene)propane-2-sulfinamide (2l)

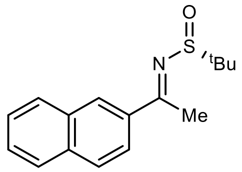

The title compound was obtained in 83% yield using General Procedure C. All analytical data were in good accordance with data reported in in the literature.[18]

(R,E)-2-Methyl-N-(1-phenylpropylidene)propane-2-sulfinamide (2m)

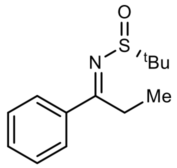

The title compound was obtained in 44% yield using General Procedure C. All analytical data were in good accordance with data reported in in the literature.[13]

(R,E)-2-methyl-N-(1-(thiophen-3-yl)ethylidene)propane-2-sulfinamide (2n)

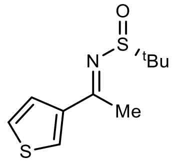

The title compound was obtained in 82% yield (790 mg, 3.44 mmol) using General Procedure C (heptane/ethyl acetate 1:1, Rf = 0.22).

^{1}H NMR (400 MHz, CDCl$_3$) δ 7.83 (dd, *J* = 2.9, 1.2 Hz, 1H), 7.57 (dd, *J* = 5.1, 1.1 Hz, 1H), 7.30 (dd, *J* = 5.1, 2.9 Hz, 1H), 2.72 (s, 3H), 1.30 (s, 9H).

^{13}C NMR (100 MHz, CDCl$_3$) δ 171.4, 143.0, 128.8, 126.7, 126.2, 57.2, 22.5 (3C), 20.4.

IR (neat) ν: 3082, 2958, 2926, 2867, 1715, 1584, 1515, 1475, 1456, 1423, 1388, 1362, 1265, 1064, 909, 875, 793, 758, 735, 644 cm^{-1}.

HRMS (ESI$^+$): exact mass calculated for $[M+H]^+$ ($C_{10}H_{16}NOS_2$) requires m/z 230.0668, found m/z 230.0668.

$[\alpha]_D^{20}$ = -0.47 (c = 1.0, $CHCl_3$).

Enantiomeric ratio > 99:1 was determined by chiral HPLC analysis: Chiralpak IH-3, *n*-heptane+0.1%IPA/IPA 9:1, 1 mL/min, 25 °C, detection at 230 nm, retention time (min): 5.9 (major) and 10.5 (minor).

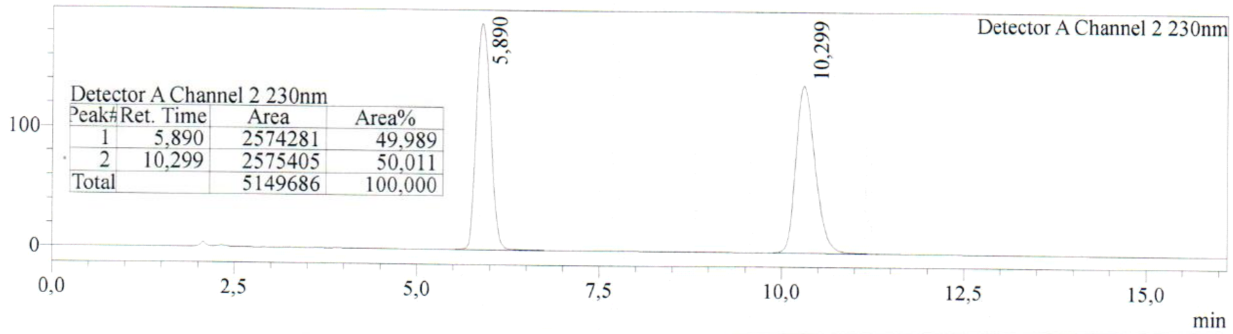

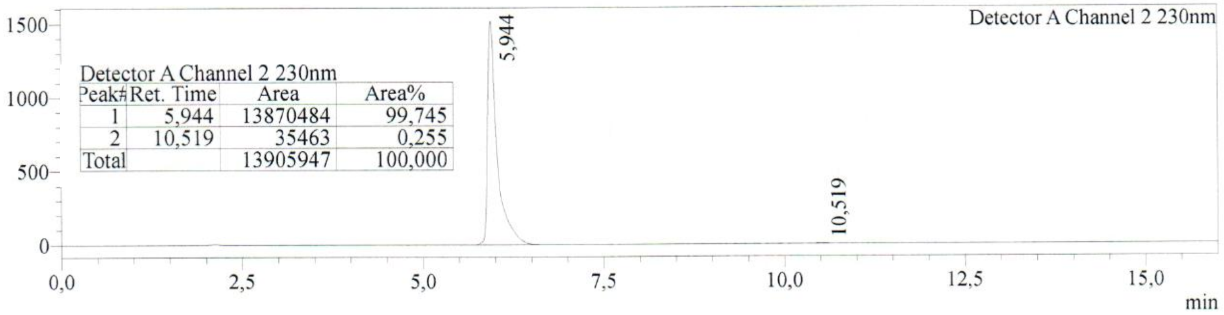

***(R)*-*N*-(Heptan-4-ylidene)-2-methylpropane-2-sulfinamide (2o)**

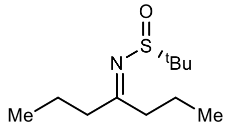

The title compound was obtained in 63% yield using General Procedure C. All analytical data were in good accordance with data reported in in the literature.[23]

***(R)-N*-cyclopentadecylidene-2-methylpropane-2-sulfinamide (2p)**

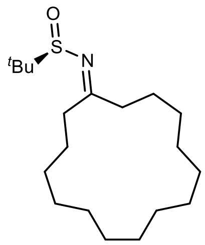

The title compound was obtained in 85% yield (1.17 g, 3.57 mmol) using General Procedure C (heptane/ethyl acetate 1:1, Rf = 0.5).

^{1}H NMR (600 MHz, $CDCl_3$) δ 2.82 – 2.61 (m, 1H), 2.40 (t, *J* = 7.3 Hz, 1H), 1.73 – 1.61 (m, 22H), 1.42 – 1.29 (m, 9H), 1.23 (s, 4H).

^{13}C NMR (100 MHz, $CDCl_3$) δ 189.4, 40.3, 35.8, 27.7, 27.2, 26.7, 26.5 (4C), 26.4, 26.3(2C), 26.2, 26.1, 24.6, 22.2 (3C).

IR (neat) ν: 2974, 2877, 1716, 1627, 1459, 1419, 1361, 1222, 1183, 1075, 759, 586 cm^{-1}.

HRMS (ESI$^+$): exact mass calculated for $[M+H]^+$ ($C_{19}H_{38}NOS$) requires m/z 328.2669, found m/z 328.2668.

$[\alpha]_D^{20}$ = -4.05 (c = 4.0, $CHCl_3$).

Enantiomeric ratio > 99:1 was determined by chiral HPLC analysis: Chiralpak IH-3, *n*-heptane+0.1%IPA/IPA 95:5, 1 mL/min, 25 °C, detection at 230 nm, retention time (min): 3.3 (major) and 2.9 (minor).

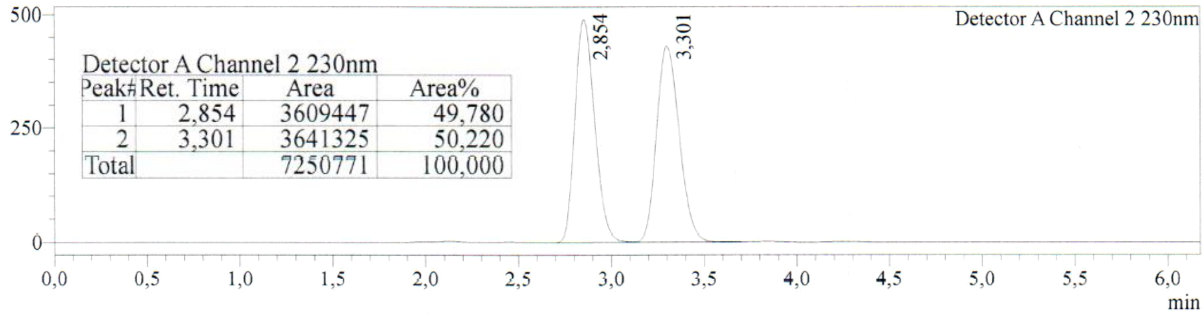

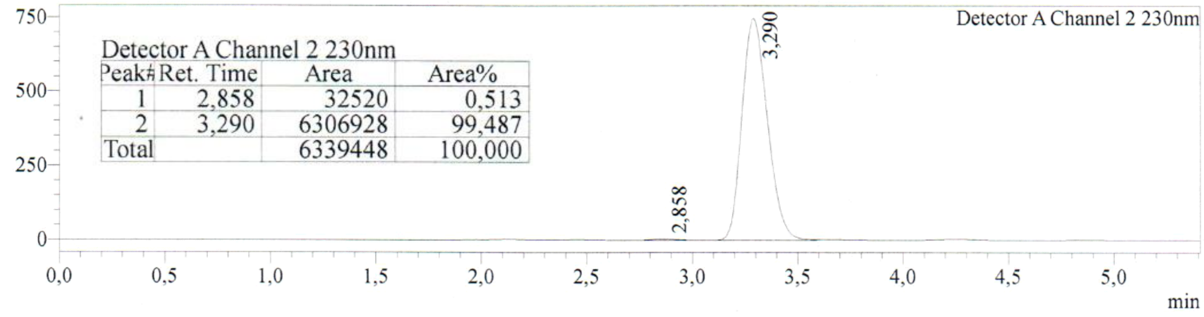

(R,E)-N-(2,3-dihydro-1*H*-inden-1-ylidene)-2-methylpropane-2-sulfinamide (2q)

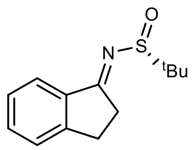

The title compound was obtained in 51% yield using General Procedure C. All analytical data were in good accordance with data reported in in the literature.[20]

(R,E)-N-(chroman-4-ylidene)-2-methylpropane-2-sulfinamide (2r)

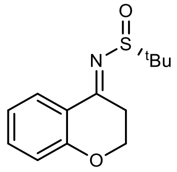

The title compound was obtained in 48% yield using General Procedure C. All analytical data were in good accordance with data reported in in the literature.[22]

(R,E)-N-(3,4-dihydronaphthalen-1(2*H*)-ylidene)-2-methylpropane-2-sulfinamide (2s)

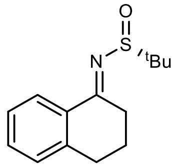

The title compound was obtained in 58% yield using General Procedure C. All analytical data were in good accordance with data reported in in the literature.[20]

4. β-Amino amide synthesis

4.1 General procedure for the synthesis of β-amino amides

General Procedure D:

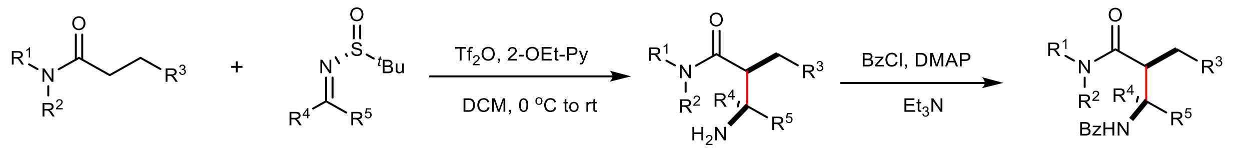

To a cooled (0 °C) mixture of amide (0.2 mmol, 1.00 equiv.), 2-OEt-pyridine (54 µL, 0.44 mmol, 2.20 equiv.) in CH_2Cl_2 (1 mL) under argon in flame-dried Schlenk tube triflic anhydride (40 µL, 0.24 mmol, 1.20 equiv.) was added dropwise. After stirring for 30 min at 0 °C, sulfonimine (0.40 mmol, 2.00 equiv.) in CH_2Cl_2 (1 mL) was added and the reaction mixture was stirred for a further 5 min at 0 °C. After 24 h of stirring at room temperature, triethylamine (139 µL, 1.00 mmol, 5.00 equiv.), 4-dimethylaminopyridine (DMAP, 5.0 mg, 0.04 mmol, 0.20 equiv.) and benzoyl chloride (116 µL, 1.00 mmol, 5.00 equiv.) were added. After full consumption of the unprotected β-amino amide (monitored by LC/MS), excess benzoyl chloride was quenched by the addition of a saturated aqueous solution of sodium bicarbonate. The resulting biphasic mixture was extracted with CH_2Cl_2 (twice). The combined organic layers were dried over anhydrous magnesium sulfate, the dried solution was filtered and the filtrate was concentrated under reduced pressure to afford the crude product. At this point, the diastereomeric ratio was determined ^{1}H-NMR analysis. The product (major diastereomer) was obtained after purification by flash column chromatography on silica gel.

4.2 Characterization of the prepared β-amino amides

N-((2*S*,3*S*)-2-Phenyl-3-(pyrrolidine-1-carbonyl)hexan-2-yl)benzamide (3a)

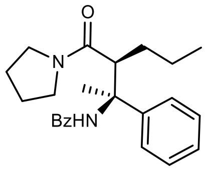

Prepared according to the general procedure in 73% (55 mg, 0.146 mmol) yield as a white foam (heptane/ethyl acetate 1:1, Rf = 0.38). The *d.r.* was determined by crude NMR as 10:1.

^{1}H NMR (600 MHz, $CDCl_3$) δ 9.10 (s, 1H), 7.99 (d, *J* = 7.1 Hz, 2H), 7.51 – 7.42 (m, 3H), 7.33 (d, *J* = 4.3 Hz, 4H), 7.23 (dd, *J* = 8.5, 4.3 Hz, 1H), 3.70 (dt, *J* = 9.7, 6.8 Hz, 1H), 3.58 (qd, *J* = 12.0, 6.1 Hz, 3H), 2.69 (dd, *J* = 11.6, 3.3 Hz, 1H), 2.08 – 1.99 (m, 2H), 1.95 (ddd, *J* = 10.2, 6.7, 2.7 Hz, 2H), 1.91 (d, *J* = 6.6 Hz, 3H), 1.80 (ddd, *J* = 24.1, 11.3, 4.5 Hz, 1H), 1.23 – 1.12 (m, 1H), 1.08 – 1.00 (m, 1H), 0.97 – 0.90 (m, 1H), 0.74 (t, *J* = 7.2 Hz, 3H).

^{13}C NMR (150 MHz, $CDCl_3$) δ 173.9, 166.5, 142.4, 135.3, 131.16, 128.5 (2C), 128.0 (2C), 127.2 (2C), 126.5, 126.0 (2C), 60.8, 54.4, 47.8, 45.8, 30.5, 26.1, 24.4, 24.38, 20.8, 14.1.

IR (neat) ν: 3300, 2957, 2928, 1718, 1601, 1523, 1452, 1310, 1384, 1253, 1107, 1028, 779, 756 cm^{-1}.

$[\alpha]_D^{20}$ = -0.35 (c = 1.0, $CHCl_3$).

HRMS (ESI$^+$): exact mass calculated for $[M+Na]^+$ ($C_{24}H_{30}N_2O_2Na$) requires m/z 401.2199, found m/z 401.2196.

Enantiomeric ratio = 99.9:0.1 was determined by chiral HPLC analysis: Chiralpak IC, *n*-heptane+0.1%IPA/IPA 75:25, 1 mL/min, 25 °C, detection at 230 nm, retention time (min): 42.1 (major) and 21.4 (minor).

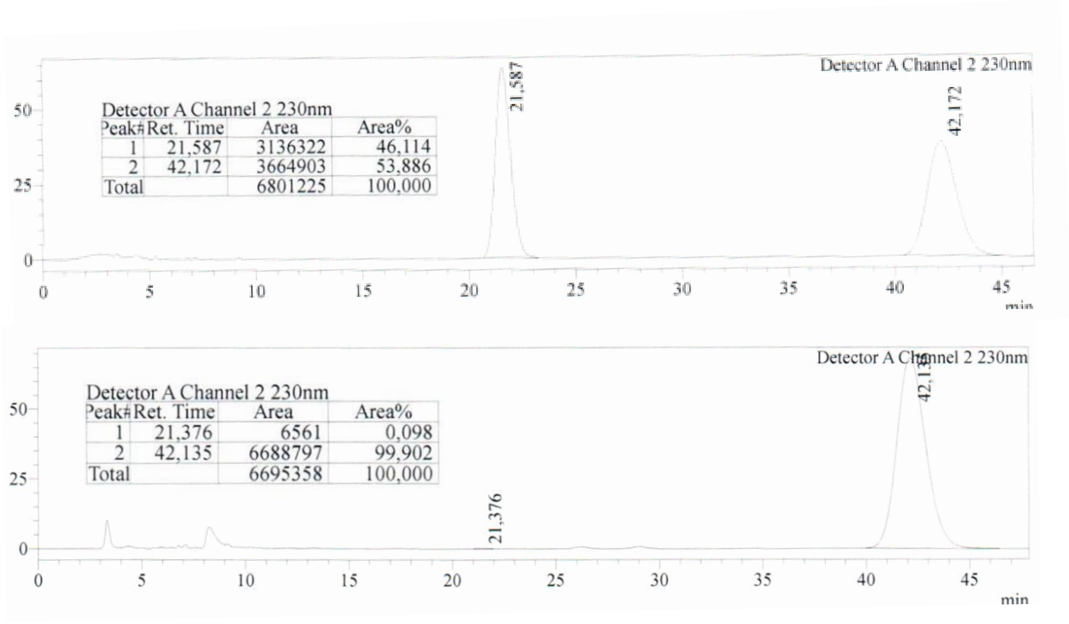

***N*-((2*S*,3*S*)-3-Methyl-4-oxo-2-phenyl-4-(pyrrolidin-1-yl)butan-2-yl)benzamide (3b)**

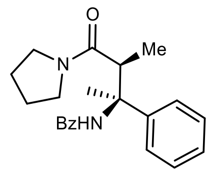

Prepared according to the general procedure in 55% yield (39 mg, 0.11 mmol) as a white foam (heptane/ethyl acetate 1:1, Rf = 0.35). The *d.r.* was determined by crude NMR as 8:1.

^{1}H NMR (600 MHz, $CDCl_3$) δ 9.20 (s, 1H), 7.99 (dd, *J* = 8.2, 1.3 Hz, 2H), 7.51 – 7.43 (m, 3H), 7.37 – 7.29 (m, 4H), 7.23 (ddd, *J* = 8.5, 6.3, 4.3 Hz, 1H), 3.67 (dt, *J* = 9.7, 7.0 Hz, 1H), 3.55 (tdd, *J* = 13.2, 11.0, 6.5 Hz, 3H), 2.74 (q, *J* = 7.0 Hz, 1H), 2.03 (pd, *J* = 12.7, 6.5 Hz, 2H), 1.98 – 1.89 (m, 5H), 0.97 (d, *J* = 7.0 Hz, 3H).

^{13}C NMR (150 MHz, $CDCl_3$) δ 174.6, 166.6, 142.2, 135.3, 131.2, 128.5 (2C), 127.9 (2C), 127.0 (2C), 126.4, 126.0 (2C), 60.5, 48.1, 47.6, 45.9, 26.1, 24.4, 24.1, 13.4.

IR (neat) ν: 3293, 3059, 2972, 2931, 1716, 1670, 1620, 1580, 1523, 1486, 1455, 1372, 1310, 1227, 1189, 1074, 756, 701 cm^{-1}.

$[\alpha]_D^{20}$ = -0.09 (c = 1.0, $CHCl_3$).

HRMS (ESI$^+$): exact mass calculated for $[M+Na]^+$ ($C_{22}H_{26}N_2O_2Na$) requires m/z 373.1886, found m/z 373.1884.

Enantiomeric ratio = 98:2 was determined by chiral HPLC analysis: Chiralpak IC, *n*-heptane+0.1%IPA/IPA 70:30, 1 mL/min, 25 °C, detection at 254 nm, retention time (min): 42.1 (major) and 25.5 (minor).

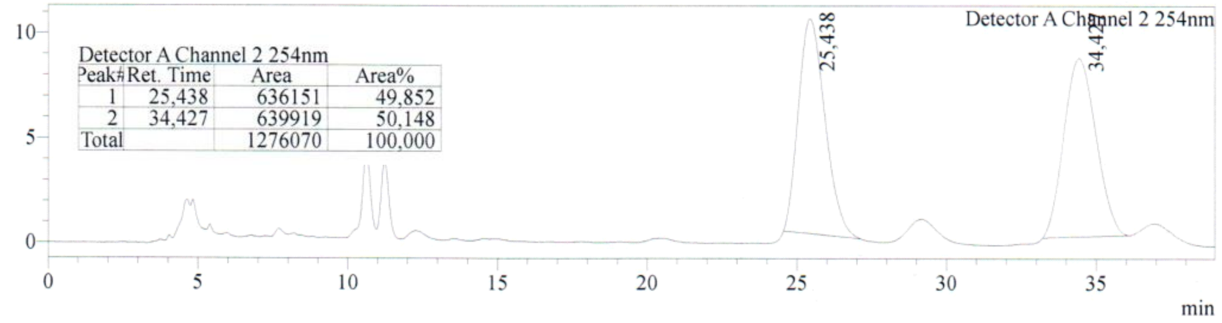

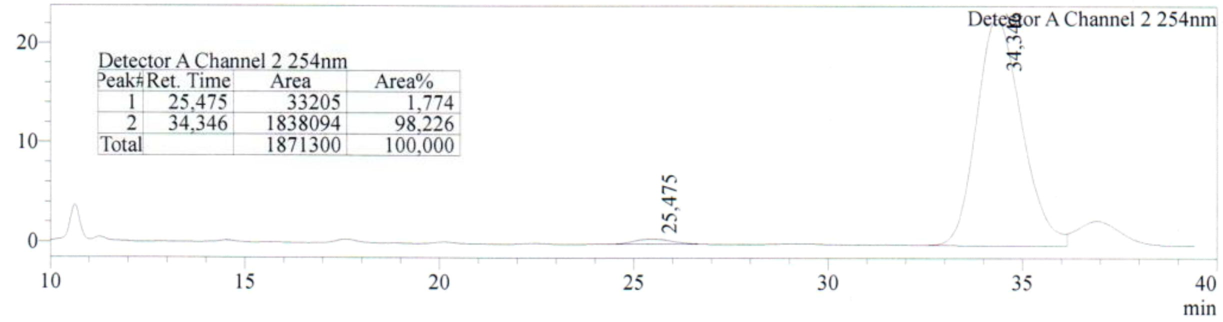

N-((2S,3S)-5-Methyl-2-phenyl-3-(pyrrolidine-1-carbonyl)hexan-2-yl)benzamide (3c)

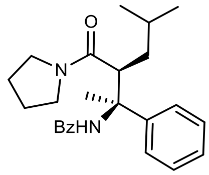

Prepared according to the general procedure in 55% yield (43 mg, 0.11 mmol) as a white foam (heptane/ethyl acetate 1:1, Rf = 0.38). The *d.r.* was determined by crude NMR to be >20:1.

^{1}H NMR (600 MHz, $CDCl_3$) δ 8.93 (s, 1H), 7.99 (d, *J* = 7.2 Hz, 2H), 7.51 – 7.43 (m, 3H), 7.34 – 7.29 (m, 4H), 7.22 (ddd, *J* = 8.4, 5.9, 2.7 Hz, 1H), 3.79 – 3.72 (m, 1H), 3.64 – 3.52 (m, 3H), 2.78 (dd, *J* = 11.1, 2.4 Hz, 1H), 2.07 – 2.00 (m, 2H), 1.97 – 1.90 (m, 5H), 1.90 – 1.83 (m, 1H), 1.20 (dtd, *J* = 13.2, 6.5, 3.2 Hz, 1H), 0.91 – 0.85 (m, 1H), 0.76 (d, *J* = 6.6 Hz, 3H), 0.56 (d, *J* = 6.5 Hz, 3H).

^{13}C NMR (150 MHz, $CDCl_3$) δ 173.9, 166.5, 142.3, 135.3, 131.1, 128.4 (2C), 127.9 (2C), 127.1 (2C), 126.4, 126.0 (2C), 61.2, 52.3, 47.8, 45.9, 37.2, 26.1, 26.0, 24.3, 24.2, 23.7, 21.6.

IR (neat) ν: 3311, 3060, 2956, 2872, 1716, 1671, 1619, 1580, 1525, 1487, 1448, 1368, 1341, 1309, 1224, 758, 701 cm^{-1}.

$[\alpha]_D^{20}$ = -0.42 (c = 1.0, $CHCl_3$).

HRMS (ESI$^+$): exact mass calculated for $[M+Na]^+$ ($C_{25}H_{32}N_2O_2Na$) requires m/z 415.2356, found m/z 415.2349.

Enantiomeric ratio = 94:6 was determined by chiral HPLC analysis: Chiralpak IC, *n*-heptane+0.1%IPA/EtOH 80:20, 1 mL/min, 25 °C, detection at 230 nm, retention time (min): 12.7 (major) and 10.3 (minor).

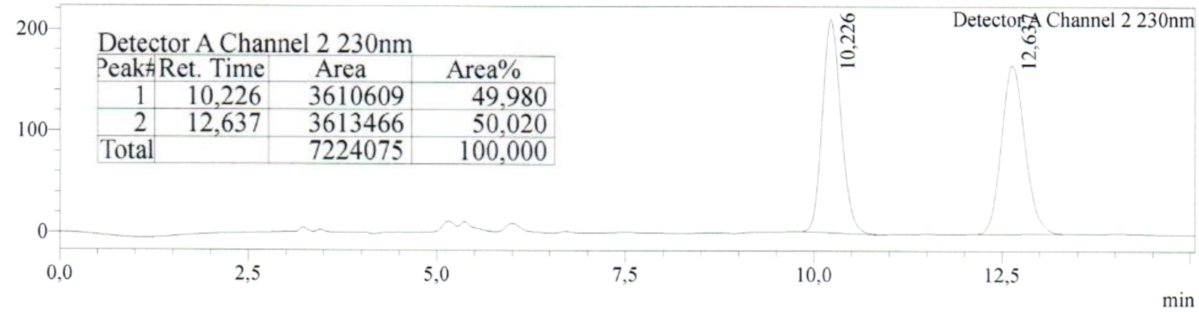

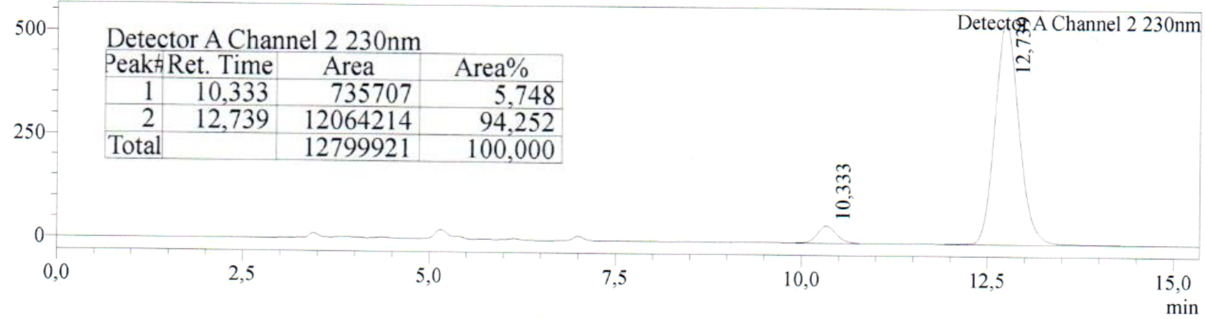

***N*-((2*S*,3*S*)-7-Chloro-2-phenyl-3-(pyrrolidine-1-carbonyl)heptan-2-yl)benzamide (3d)**

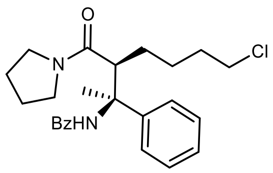

Prepared according to the general procedure in 55% yield (47 mg, 0.11 mmol) as a white foam (heptane/ethyl acetate 1:1, Rf = 0.32). The *d.r.* was determined by crude NMR as 9:1.

^{1}H NMR (600 MHz, $CDCl_3$) δ 9.06 (s, 1H), 8.03 – 7.95 (m, 2H), 7.53 – 7.41 (m, 3H), 7.32 (d, *J* = 4.3 Hz, 4H), 7.25 – 7.20 (m, 1H), 3.70 (dt, *J* = 9.6, 6.9 Hz, 1H), 3.64 – 3.55 (m, 3H), 3.41 – 3.30 (m, 2H), 2.70 (dd, *J* = 11.5, 2.8 Hz, 1H), 2.09 – 2.00 (m, 2H), 2.00 – 1.89 (m, 5H), 1.89 – 1.79 (m, 1H), 1.66 – 1.49 (m, 2H), 1.26 (dd, *J* = 12.9, 5.9 Hz, 1H), 1.14 – 1.03 (m, 2H).

^{13}C NMR (150 MHz, $CDCl_3$) δ 173.5, 166.5, 142.2, 135.2, 131.2, 128.5 (2C), 128.0 (2C), 127.1 (2C), 126.6, 125.9 (2C), 60.8, 54.2, 47.8, 45.9, 44.6, 32.3, 27.6, 26.1, 24.7, 24.4, 24.2.

IR (neat) ν: 3304, 2971, 2876, 1716, 1670, 1619, 1580, 1524, 1487, 1450, 1371, 1340, 1310, 1225, 757, 701 cm^{-1}.

$[\alpha]_D^{20}$ = -0.64 (c = 2.0, $CHCl_3$).

HRMS (ESI$^+$): exact mass calculated for $[M+Na]^+$ ($C_{25}H_{31}N_2O_2{}^{35}ClNa$) requires m/z 449.1966, found m/z 449.1961.

Enantiomeric ratio = 90:10 was determined by chiral HPLC analysis: Chiralpak IC, *n*-heptane+0.1%IPA/EtOH 80:20, 1 mL/min, 25 °C, detection at 230 nm, retention time (min): 15.0 (major) and 13.4 (minor).

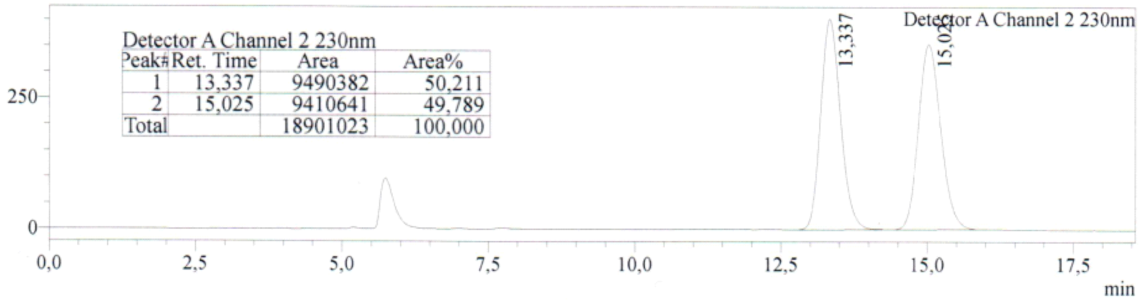

Detector A Channel 2 230nm

Peak#	Ret. Time	Area	Area%
1	13,337	9490382	50,211
2	15,025	9410641	49,789
Total		18901023	100,000

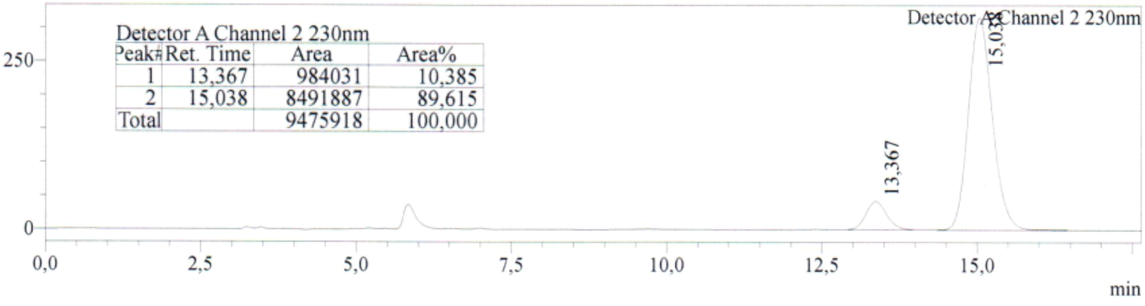

Detector A Channel 2 230nm

Peak#	Ret. Time	Area	Area%
1	13,367	984031	10,385
2	15,038	8491887	89,615
Total		9475918	100,000

N-((2S,3S)-7-Cyano-2-phenyl-3-(pyrrolidine-1-carbonyl)heptan-2-yl)benzamide (3e)

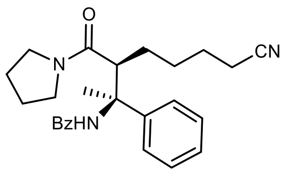

Prepared according to the general procedure in 50% yield (42 mg, 0.10 mmol) as a white foam (heptane/ethyl acetate 1:1, Rf = 0.15). The *d.r.* was determined by crude NMR as 12:1.

^{1}H NMR (600 MHz, $CDCl_3$) δ 9.03 (s, 1H), 7.97 (d, *J* = 7.0 Hz, 2H), 7.52 – 7.43 (m, 3H), 7.36 – 7.29 (m, 4H), 7.25 – 7.21 (m, 1H), 3.70 (dt, *J* = 9.5, 6.8 Hz, 1H), 3.64 – 3.54 (m, 3H), 2.70 (dd, *J* = 11.4, 2.9 Hz, 1H), 2.19 – 2.12 (m, 2H), 2.09 – 2.01 (m, 2H), 1.99 – 1.93 (m, 2H), 1.91 (s, 3H), 1.89 – 1.80 (m, 1H), 1.53 – 1.41 (m, 2H), 1.28 – 1.23 (m, 1H), 1.09 (tdd, *J* = 12.5, 5.3, 3.3 Hz, 2H).

^{13}C NMR (150 MHz, $CDCl_3$) δ 173.3, 166.5, 142.1, 135.2, 131.2, 128.5 (2C), 128.0 (2C), 127.1 (2C), 126.7, 125.9 (2C), 119.3, 60.8, 54.0, 47.9, 45.9, 27.5, 26.4, 26.0, 25.1, 24.3, 24.2, 16.8.

IR (neat) ν: 3311, 2971, 2871, 1716, 1669, 1619, 1579, 1524, 1487, 1451, 1369, 1340, 1310, 1225, 757, 702 cm^{-1}.

$[\alpha]_D^{20}$ = -0.44 (c = 2.0, $CHCl_3$).

HRMS (ESI$^+$): exact mass calculated for $[M+Na]^+$ ($C_{26}H_{31}N_3O_2Na$) requires m/z 440.2308, found m/z 440.2307.

Enantiomeric ratio = 93:7 was determined by chiral HPLC analysis: Chiralpak IC, *n*-heptane+0.1%IPA/EtOH 85:15, 1 mL/min, 25 °C, detection at 230 nm, retention time (min): 6.7 (major) and 7.6 (minor).

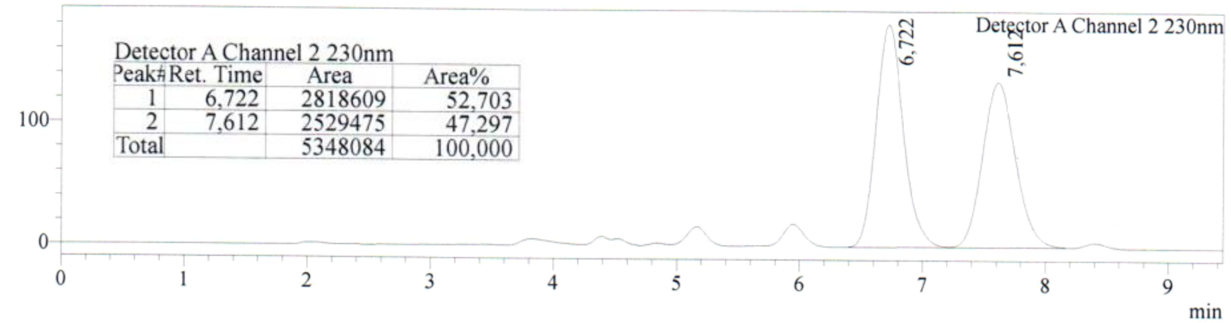

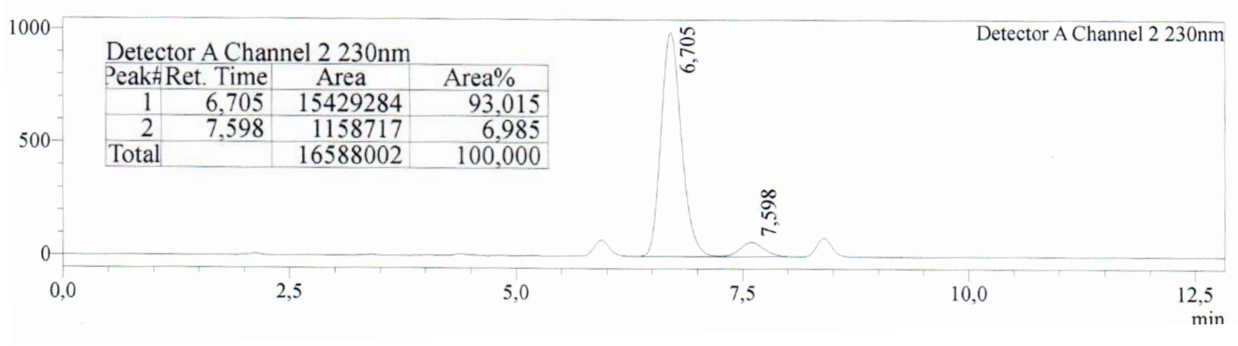

***N*-((2*S*,3*S*)-7-Methoxy-2-phenyl-3-(pyrrolidine-1-carbonyl)heptan-2-yl)benzamide (3f)**

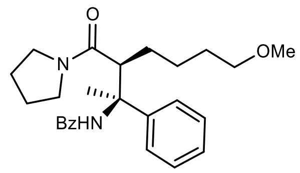

Prepared according to the general procedure in 71% yield (60 mg, 0.14 mmol) as a yellow foam (heptane/ethyl acetate 1:1, Rf = 0.25). The *d.r.* was determined by crude NMR as 8:1.

^{1}H NMR (600 MHz, CDCl$_3$) δ 9.07 (s, 1H), 7.98 (d, *J* = 7.2 Hz, 2H), 7.51 – 7.42 (m, 3H), 7.31 (d, *J* = 4.2 Hz, 4H), 7.22 (dq, *J* = 8.6, 4.4 Hz, 1H), 3.71 – 3.65 (m, 1H), 3.63 – 3.54 (m, 3H), 3.20 (s, 3H), 3.20 – 3.13 (m, 2H), 2.69 (dd, *J* = 11.5, 3.1 Hz, 1H), 2.02 (td, *J* = 12.9, 6.4 Hz, 2H), 1.94 (dd, *J* = 13.6, 6.8 Hz, 2H), 1.90 (s, 3H), 1.82 (ddd, *J* = 23.9, 11.5, 4.3 Hz, 1H), 1.41 – 1.34 (m, 2H), 1.22 – 1.14 (m, 1H), 1.14 – 1.05 (m, 1H), 0.98 (ddd, *J* = 19.4, 11.0, 5.7 Hz, 1H).

^{13}C NMR (150 MHz, CDCl$_3$) δ 173.7, 166.5, 142.3, 135.3, 131.1, 128.5 (2C), 127.9 (2C), 127.2 (2C), 126.5, 126.0 (2C), 72.1, 60.8, 58.4, 54.3, 47.8, 45.9, 29.4, 28.1, 26.1, 24.4, 24.2, 24.0.

IR (neat) ν: 3019, 2971, 2942, 1715, 1671, 1620, 1524, 1448, 1365, 1223, 759 cm^{-1}.

$[\alpha]_D^{20}$ = -0.65 (c = 2.0, CHCl$_3$).

HRMS (ESI$^+$): exact mass calculated for $[M+Na]^+$ ($C_{26}H_{34}N_2O_3Na$) requires m/z 445.2462, found m/z 445.2463.

Enantiomeric ratio = 99:1 was determined by chiral HPLC analysis: Chiralpak IC, *n*-heptane+0.1%IPA/EtOH 60:40, 1 mL/min, 25 °C, detection at 230 nm, retention time (min): 13.8 (major) and 12.3 (minor).

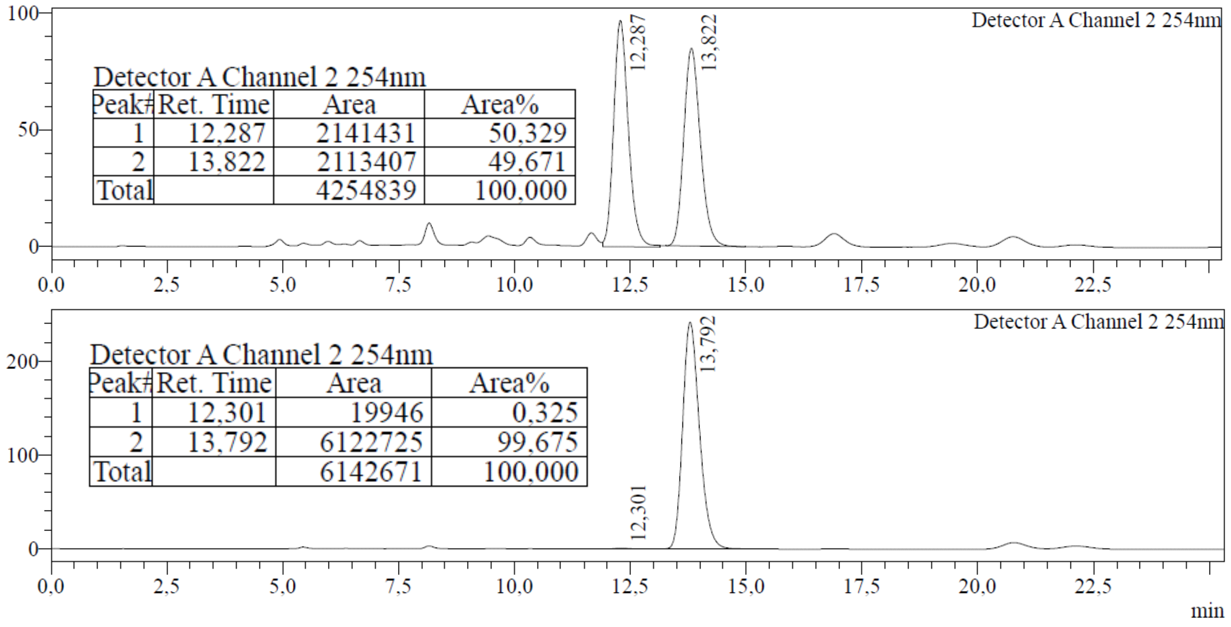

N-((2S,3S)-7-(1,3-Dioxoisoindolin-2-yl)-2-phenyl-3-(pyrrolidine-1-carbonyl)heptan-2-yl)benzamide (3g)

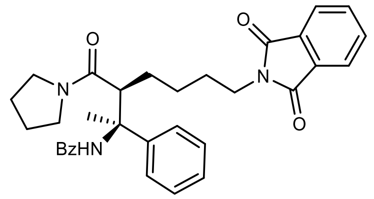

Prepared according to the general procedure in 63% yield (68 mg, 0.126 mmol) as a white solid (heptane/ethyl acetate 1:1, Rf = 0.16). The *d.r.* was determined by crude NMR as 12:1.

^{1}H NMR (700 MHz, CDCl$_3$) δ 9.09 (s, 1H), 7.99 – 7.94 (m, 2H), 7.79 (dd, *J* = 5.4, 3.0 Hz, 2H), 7.69 (dd, *J* = 5.4, 3.0 Hz, 2H), 7.50 – 7.46 (m, 1H), 7.44 (t, *J* = 7.3 Hz, 2H), 7.32 – 7.27 (m, 4H), 7.20 – 7.15 (m, 1H), 3.71 – 3.64 (m, 1H), 3.58 – 3.47 (m, 5H), 2.67 (dd, *J* = 11.4, 3.4 Hz, 1H), 2.08 – 2.00 (m, 2H), 1.94 (dd, *J* = 13.7, 6.9 Hz, 2H), 1.89 (s, 3H), 1.86 – 1.78 (m, 1H), 1.52 – 1.43 (m, 2H), 1.22 – 1.15 (m, 1H), 1.08 (tdd, *J* = 11.0, 7.0, 4.4 Hz, 1H), 0.99 – 0.92 (m, 1H).

^{13}C NMR (175 MHz, CDCl$_3$) δ 173.6, 168.2, 166.5, 142.2, 135.2 (2C), 133.9, 132.0 (2C), 131.1, 128.4 (2C), 128.0 (2C), 127.1 (2C), 126.5 (2C), 125.9, 123.0 (2C), 60.6, 54.2, 47.8, 45.8, 37.7, 28.5, 27.9, 26.0, 25.1, 24.3, 24.2.

IR (neat) ν: 2971, 2877, 17771, 1711, 1670, 1618, 1579, 1523, 1487, 1450, 1396, 1366, 1310, 1224, 757, 719, 530 cm^{-1}.

$[\alpha]_D^{20}$ = -0.48 (c = 2.0, CHCl$_3$).

HRMS (ESI$^+$): exact mass calculated for [M+Na]$^+$ ($C_{33}H_{35}N_3O_4Na$) requires m/z 560.2520, found m/z 560.2521.

Enantiomeric ratio = 93:7 was determined by chiral HPLC analysis: Chiralpak IC, *n*-heptane+0.1%IPA/EtOH 7:3, 1 mL/min, 25 °C, detection at 210 nm, retention time (min): 25.8 (major) and 22.1 (minor).

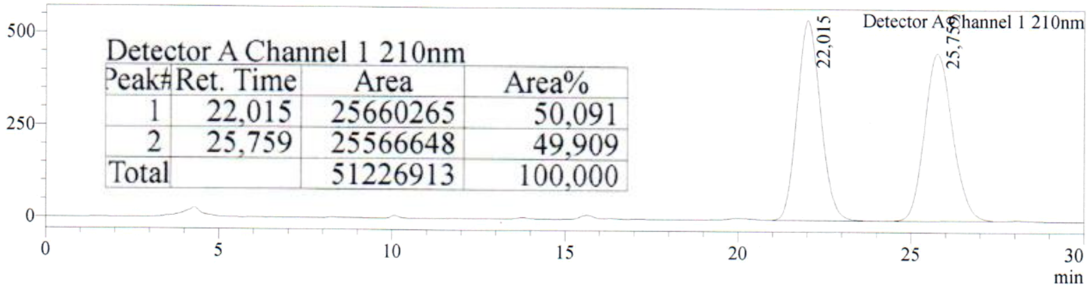

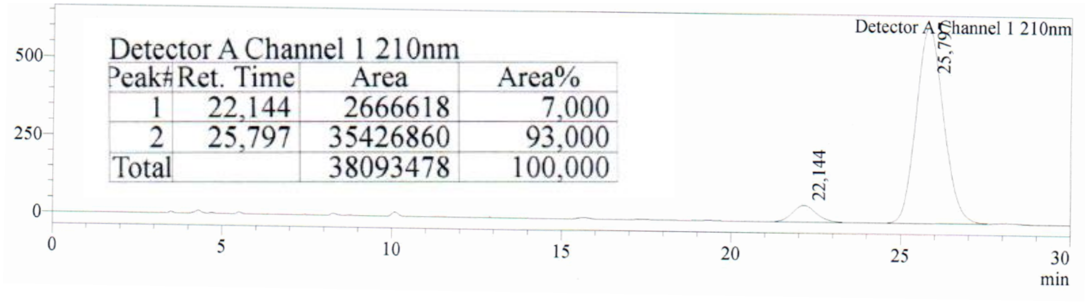

Methyl (8*S*,9*S*)-9-benzamido-9-phenyl-8-(pyrrolidine-1-carbonyl)decanoate (3h)

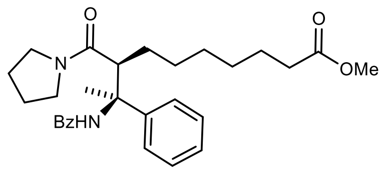

Prepared according to the general procedure in 60% yield (57 mg, 0.12 mmol) as a yellow oil (heptane/ethyl acetate 1:1, Rf = 0.25). The *d.r.* was determined by crude NMR as 9: 1.

^{1}H NMR (600 MHz, $CDCl_3$) δ 9.07 (s, 1H), 8.04 – 7.91 (m, 2H), 7.51 – 7.41 (m, 3H), 7.32 (d, *J* = 4.3 Hz, 4H), 7.26 – 7.18 (m, 1H), 3.69 (dt, *J* = 9.7, 6.9 Hz, 1H), 3.63 (s, 3H), 3.61 – 3.52 (m, 3H), 2.67 (dd, *J* = 11.5, 3.2 Hz, 1H), 2.21 (t, *J* = 7.5 Hz, 2H), 2.09 – 1.99 (m, 2H), 1.95 (dd, *J* = 13.7, 6.9 Hz, 2H), 1.92 – 1.88 (m, 3H), 1.79 (dt, *J* = 13.0, 7.6 Hz, 1H), 1.55 – 1.43 (m, 2H), 1.12 (m, 5H), 1.08 – 1.01 (m, 1H), 0.89 (dt, *J* = 14.1, 7.0 Hz, 1H).

^{13}C NMR (150 MHz, $CDCl_3$) δ 174.1, 173.8, 166.5, 142.4, 135.3, 131.1, 128.5 (2C), 128.0 (2C), 127.2 (2C), 126.5, 126.0 (2C), 60.8, 54.3, 51.4, 47.8, 45.8, 33.9, 29.2, 28.8, 28.2, 27.3, 26.1, 24.7, 24.4, 24.3.

IR (neat) ν: 3300, 2928, 2872, 1736, 1671, 1619, 1524, 1449, 1255, 1189, 1074, 1029, 701, 597 cm^{-1}.

$[\alpha]_D^{20}$ = -0.58 (c = 2.0, $CHCl_3$).

HRMS (ESI^+): exact mass calculated for $[M+Na]^+$ ($C_{29}H_{38}N_2O_4Na$) requires m/z 501.2724, found m/z 501.2722.

Enantiomeric ratio = 95:5 was determined by chiral HPLC analysis: Chiralpak IC, *n*-heptane+0.1%IPA/IPA 7:3, 1 mL/min, 25 °C, detection at 254 nm, retention time (min): 18.3 (major) and 15.4 (minor).

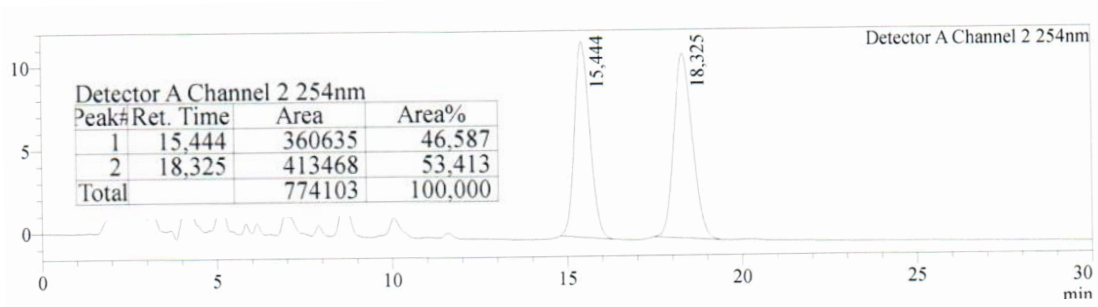

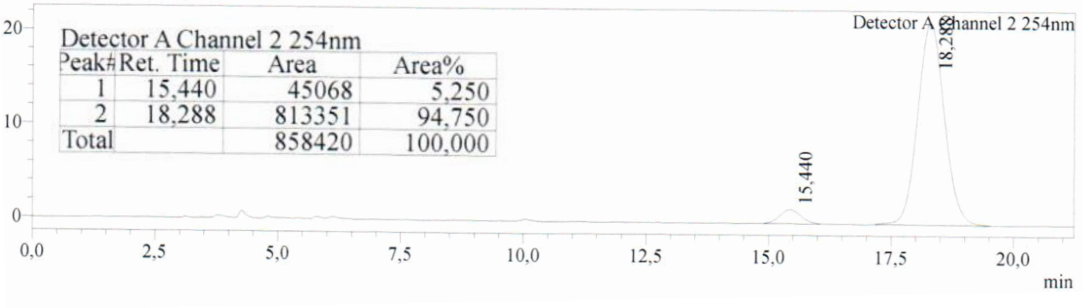

***N*-((2*S*,3*S*)-11-oxo-2-phenyl-3-(pyrrolidine-1-carbonyl)dodecan-2-yl)benzamide (3i)**

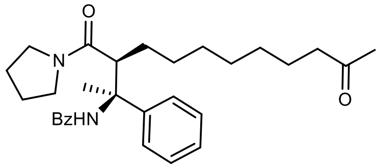

Prepared according to the general procedure in 50% yield (48 mg, 0.1 mmol) as a yellow oil (heptane/ethyl acetate 1:1, Rf = 0.22). The *d.r.* was determined by crude NMR as 5: 1.

^{1}H NMR (600 MHz, $CDCl_3$) δ 9.08 (s, 1H), 8.01 – 7.96 (m, 2H), 7.51 – 7.42 (m, 3H), 7.32 (d, *J* = 4.3 Hz, 4H), 7.22 (dt, *J* = 8.5, 4.1 Hz, 1H), 3.69 (dt, *J* = 9.8, 6.9 Hz, 1H), 3.65 – 3.52 (m, 3H), 2.67 (dd, *J* = 11.5, 3.1 Hz, 1H), 2.34 (t, *J* = 7.4 Hz, 2H), 2.10 (s, 3H), 2.03 (app dq, *J* = 12.7, 6.4 Hz, 2H), 1.99 – 1.92 (m, 2H), 1.90 (s, 3H), 1.78 (dt, *J* = 12.0, 7.8 Hz, 1H), 1.50 – 1.43 (m, 2H), 1.17 – 1.02 (m, 8H), 0.87 (dd, *J* = 13.8, 6.6 Hz, 1H).

^{13}C NMR (150 MHz, $CDCl_3$) δ 209.2, 173.9, 166.5, 142.4, 135.3, 131.1, 128.5 (2C), 127.9 (2C), 127.2 (2C), 126.5, 126.0 (2C), 60.8, 54.4, 47.8, 45.8, 43.6, 29.8, 29.3, 29.0, 28.9, 28.3, 27.4, 26.1, 24.4, 24.3, 23.6.

IR (neat) ν: 3300, 2971, 2929, 2855, 1713, 1670, 1618, 1524, 1487, 1309, 1226, 1187, 701, 597 cm^{-1}.

$[\alpha]_D^{20}$ = -0.54 (c = 2.0, $CHCl_3$).

HRMS (ESI^+): exact mass calculated for $[M+Na]^+$ ($C_{30}H_{40}N_2O_3Na$) requires m/z 499.2931, found m/z 499.2936.

Enantiomeric ratio = 95:5 was determined by chiral HPLC analysis: Chiralpak IC, *n*-heptane+0.1%IPA/EtOH 70:30, 1 mL/min, 25 °C, detection at 230 nm, retention time (min): 24.7 (major) and 20.1 (minor).

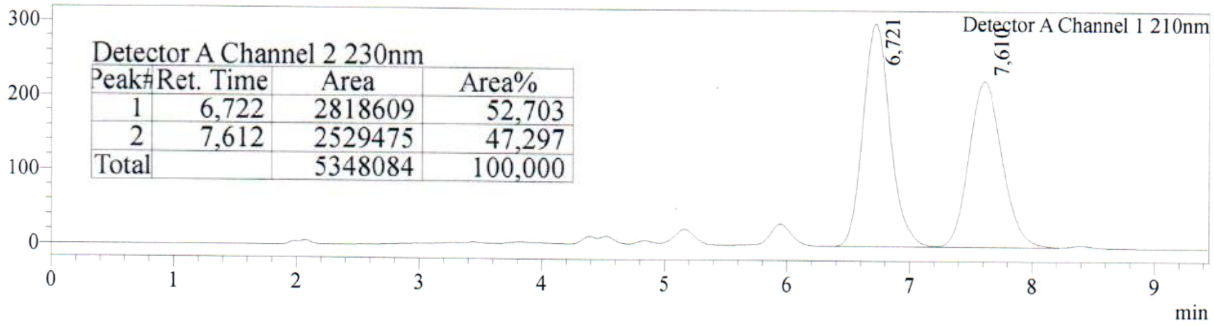

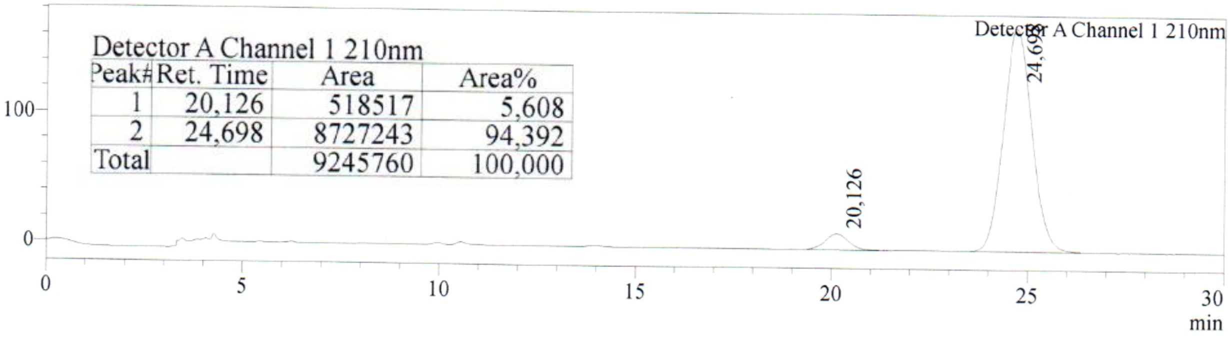

N-((2S,3S)-2-phenyl-3-(pyrrolidine-1-carbonyl)dodec-11-en-2-yl)benzamide (3j)

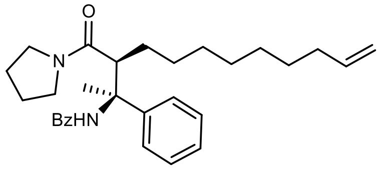

Prepared according to the general procedure in 51% yield (47 mg, 0.102 mmol) as a yellow foam (heptane/ethyl acetate 1:1, Rf = 0.38). The *d.r.* was determined by crude NMR as 12:1.

^{1}H NMR (600 MHz, $CDCl_3$) δ 9.09 (s, 1H), 8.01 – 7.96 (m, 2H), 7.51 – 7.43 (m, 3H), 7.32 (d, *J* = 4.1 Hz, 4H), 7.25 – 7.21 (m, 1H), 5.76 (ddt, *J* = 16.9, 10.2, 6.7 Hz, 1H), 4.95 (dd, *J* = 17.1, 1.8 Hz, 1H), 4.91 (dd, *J* = 10.2, 1.0 Hz, 1H), 3.69 (dd, *J* = 7.0, 2.7 Hz, 1H), 3.63 – 3.54 (m, 3H), 2.68 (dd, *J* = 11.5, 3.0 Hz, 1H), 2.03 (dq, *J* = 13.1, 6.5 Hz, 2H), 1.95 (ddd, *J* = 11.0, 8.1, 4.3 Hz, 4H), 1.92 – 1.88 (m, 3H), 1.79 (dt, *J* = 12.3, 7.7 Hz, 1H), 1.28 (dt, *J* = 14.9, 7.3 Hz, 2H), 1.20 – 1.03 (m, 8H), 0.89 (dd, *J* = 13.6, 8.1 Hz, 1H).

^{13}C NMR (150 MHz, $CDCl_3$) δ 173.9, 166.5, 142.5, 139.1, 135.4, 131.2, 128.5 (2C), 128.0 (2C), 127.2 (2C), 126.5, 126.0 (2C), 114.1, 60.8, 54.4, 47.8, 45.9, 33.70, 29.5, 29.2, 28.9, 28.7, 28.3, 27.5, 26.1, 24.4, 24.3.

IR (neat) ν: 3298, 3025, 2973, 1716, 1671, 1620, 1580, 1524, 1487, 1449, 1369, 1339, 1309, 1225, 912, 759, 700 cm^{-1}.

$[\alpha]_D^{20}$ = -0.60 (c = 2.0, $CHCl_3$).

HRMS (ESI+): exact mass calculated for $[M+H]^+$ ($C_{30}H_{41}N_2O_2$) requires m/z 461.3163, found m/z 461.3163.

Enantiomeric ratio = 94:6 was determined by chiral HPLC analysis: Chiralpak IC, *n*-heptane+0.1%IPA/EtOH 80:20, 1 mL/min, 25 °C, detection at 210 nm, retention time (min): 11.6 (major) and 9.7 (minor).

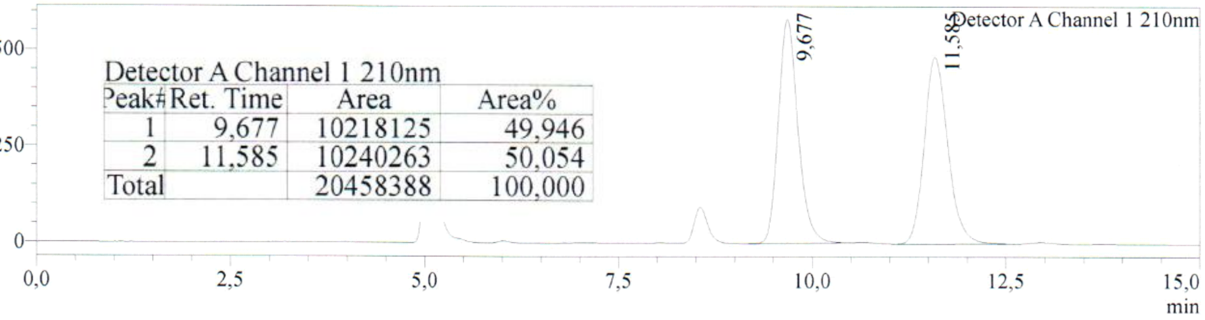

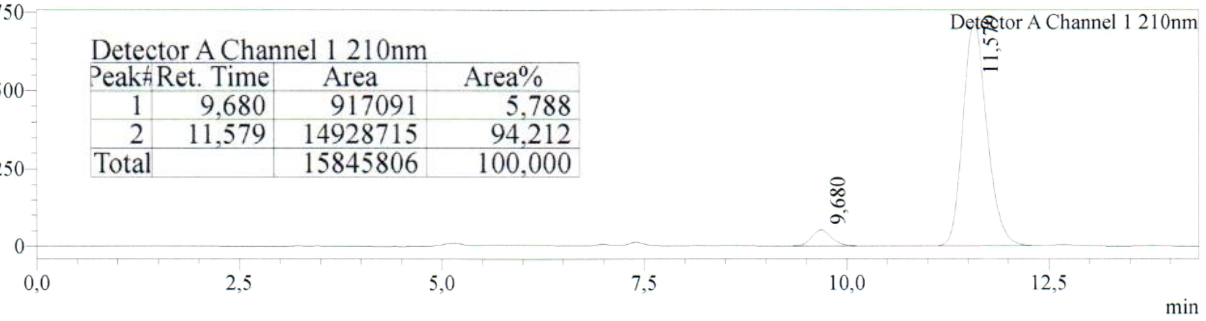

***N*-((2*S*,3*S*)-5-(But-2-yn-1-yloxy)-2-phenyl-3-(pyrrolidine-1-carbonyl)pentan-2-yl)benzamide (3k)**

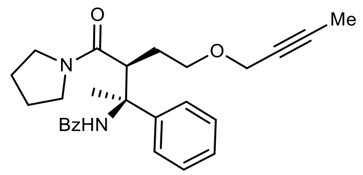

Prepared according to the general procedure in 46% yield (40 mg, 0.092 mmol) as a yellow oil (heptane/ethyl acetate 1:1, Rf = 0.32). The *d.r.* was determined by crude NMR as 11:1.

^{1}H NMR (600 MHz, $CDCl_3$) δ 9.17 (s, 1H), 8.04 – 7.94 (m, 2H), 7.52 – 7.41 (m, 3H), 7.35 – 7.28 (m, 4H), 7.23 – 7.19 (m, 1H), 3.91 (dq, *J* = 15.3, 2.2 Hz, 1H), 3.88 – 3.78 (m, 2H), 3.65 – 3.52 (m, 3H), 3.29 (td, *J* = 9.8, 3.5 Hz, 1H), 3.24 (dt, *J* = 9.4, 4.6 Hz, 1H), 2.95 (dd, *J* = 11.3, 3.7 Hz, 1H), 2.06 – 1.96 (m, 3H), 1.96 – 1.90 (m, 5H), 1.80 (t, *J* = 2.3 Hz, 3H), 1.46 – 1.38 (m, 1H).

^{13}C NMR (150 MHz, $CDCl_3$) δ 173.3, 166.5, 142.1, 135.6, 131.1, 128.4 (2C), 128.0 (2C), 127.2 (2C), 126.5, 126.0 (2C), 82.3, 74.8, 67.3, 60.6, 58.2, 50.9, 47.7, 45.9, 28.0, 26.0, 24.5, 24.2, 3.5.

IR (neat) ν: 3292, 3060, 2971, 2876, 1716, 1671, 1619, 1579, 1524, 1486, 1451, 1362, 1340, 1309, 1225, 1080, 757, 701, 668, 530 cm^{-1}.

$[\alpha]_D^{20}$ = -0.71 (c = 2.0, $CHCl_3$).

HRMS (ESI$^+$): exact mass calculated for $[M+Na]^+$ ($C_{27}H_{32}N_2O_3Na$) requires m/z 455.2305, found m/z 455.2296.

Enantiomeric ratio = 91:9 was determined by chiral HPLC analysis: Chiralpak IC, *n*-heptane+0.1%IPA/EtOH 60:40, 1 mL/min, 25 °C, detection at 254 nm, retention time (min): 15.3 (major) and 12.5 (minor).

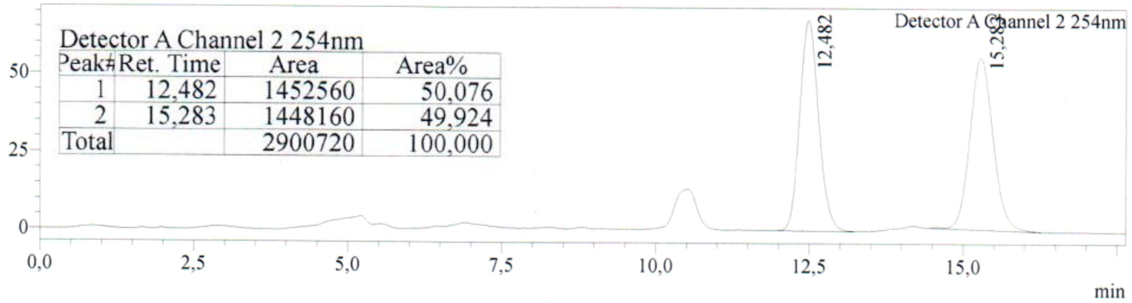

Detector A Channel 2 254nm

Peak#	Ret. Time	Area	Area%
1	12,482	1452560	50,076
2	15,283	1448160	49,924
Total		2900720	100,000

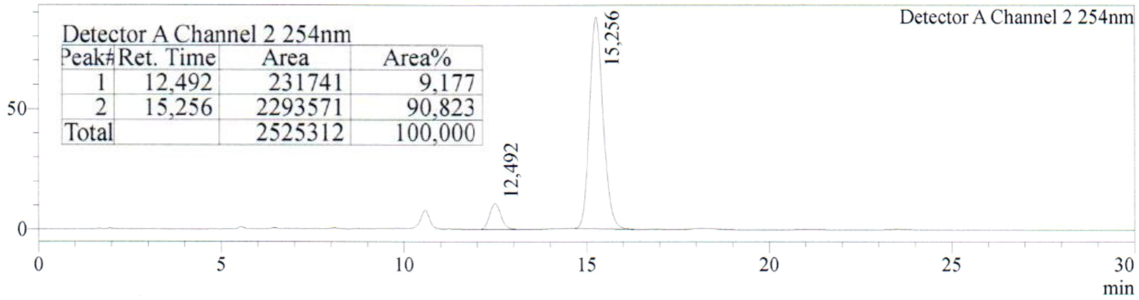

Detector A Channel 2 254nm

Peak#	Ret. Time	Area	Area%
1	12,492	231741	9,177
2	15,256	2293571	90,823
Total		2525312	100,000

***N*-((2*S*,3*S*)-2-Phenyl-3-(piperidine-1-carbonyl)hexan-2-yl)benzamide (3l)**

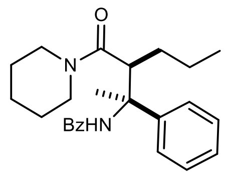

Prepared according to the general procedure in 50% yield (39 mg, 0.1 mmol) as a white foam (heptane/ethyl acetate 1:1, Rf = 0.50). The *d.r.* was determined by crude NMR to be >20: 1.

^{1}H NMR (600 MHz, $CDCl_3$) δ 9.14 (s, 1H), 7.99 (d, *J* = 7.5 Hz, 2H), 7.49 (t, *J* = 7.2 Hz, 1H), 7.45 (t, *J* = 7.4 Hz, 2H), 7.36 – 7.30 (m, 4H), 7.23 (dt, *J* = 8.2, 2.8 Hz, 1H), 3.90 – 3.83 (m, 1H), 3.71 (dt, *J* = 12.7, 4.8 Hz, 1H), 3.65 – 3.54 (m, 2H), 2.93 (dd, *J* = 11.4, 3.2 Hz, 1H), 1.90 – 1.80 (m, 4H), 1.72 (d, *J* = 5.0 Hz, 2H), 1.64 – 1.54 (m, 3H), 1.23 – 1.14 (m, 1H), 1.05 – 0.99 (m, 1H), 0.96 – 0.86 (m, 1H), 0.73 (t, *J* = 7.3 Hz, 3H).

^{13}C NMR (150 MHz, $CDCl_3$) δ 173.4, 166.4, 142.6, 135.4, 131.2, 128.5 (2C), 128.0 (2C), 127.2 (2C), 126.5, 126.0 (2C), 60.7, 50.7, 47.8, 43.1, 30.6, 26.7, 25.9, 24.7, 24.5, 21.0, 14.0.

IR (neat) ν: 3022, 2859, 1716, 1670, 1614, 1580, 1521, 1487, 1446, 1369, 1307, 1248, 1220, 758, 712, 700, 668, 592 cm^{-1}.

$[\alpha]_D^{20}$ = -0.35 (c = 2.0, $CHCl_3$).

HRMS (ESI^+): exact mass calculated for $[M+Na]^+$ ($C_{25}H_{32}N_2O_2Na$) requires m/z 415.2356, found m/z 415.2355.

Enantiomeric ratio = 90:10 was determined by chiral HPLC analysis: Chiralpak IC, *n*-heptane+0.1%IPA/IPA 70:30, 1 mL/min, 25 °C, detection at 254 nm, retention time (min): 20.8 (major) and 16.3 (minor).

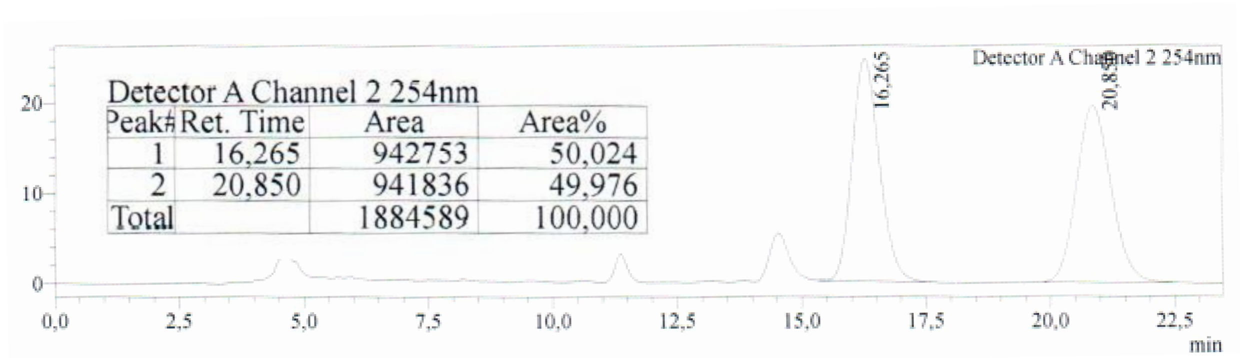

Detector A Channel 2 254nm

Peak#	Ret. Time	Area	Area%
1	16,265	942753	50,024
2	20,850	941836	49,976
Total		1884589	100,000

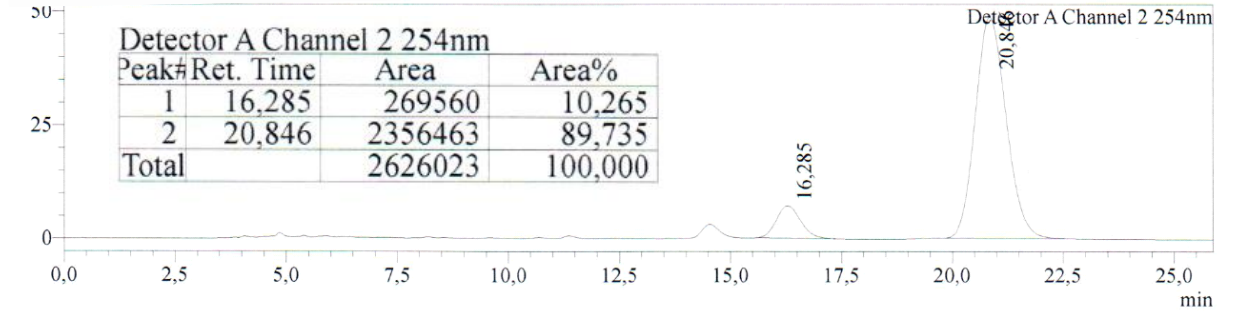

Detector A Channel 2 254nm

Peak#	Ret. Time	Area	Area%
1	16,285	269560	10,265
2	20,846	2356463	89,735
Total		2626023	100,000

***N*-((2*S*,3*S*)-3-(Azepane-1-carbonyl)-2-phenylhexan-2-yl)benzamide (3m)**

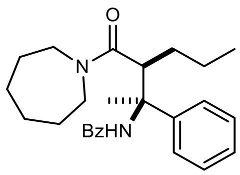

Prepared according to the general procedure in 57% yield (46 mg, 0.114 mmol) as a yellow foam (heptane/ethyl acetate 1:1, Rf = 0.50). The *d.r.* was determined by crude NMR to be >20: 1.

^{1}H NMR (600 MHz, $CDCl_3$) δ 9.15 (s, 1H), 7.99 (dd, *J* = 8.1, 1.3 Hz, 2H), 7.56 – 7.41 (m, 3H), 7.37 – 7.29 (m, 4H), 7.23 (ddd, *J* = 8.5, 5.8, 2.8 Hz, 1H), 3.88 – 3.79 (m, 1H), 3.74 (ddd, *J* = 13.8, 6.8, 5.2 Hz, 1H), 3.65 – 3.57 (m, 1H), 3.48 (ddd, *J* = 13.7, 6.8, 5.4 Hz, 1H), 2.89 (dd, *J* = 11.3, 3.4 Hz, 1H), 1.90 (s, 3H), 1.89 – 1.85 (m, 1H), 1.85 – 1.79 (m, 2H), 1.79 – 1.74 (m, 2H), 1.64 (dddd, *J* = 17.6, 12.2, 6.0, 3.4 Hz, 4H), 1.19 (app dqd, *J* = 14.4, 7.3, 4.5 Hz, 1H), 1.10 – 1.02 (m, 1H), 0.97 – 0.87 (m, 1H), 0.74 (t, *J* = 7.2 Hz, 3H).

^{13}C NMR (150 MHz, $CDCl_3$) δ 175.1, 166.4, 142.6, 135.4, 131.1, 128.5 (2C), 127.9 (2C), 127.2 (2C), 126.4, 126.0 (2C), 60.9, 51.3, 49.0, 45.8, 30.9, 29.0, 28.0, 27.3, 26.5, 24.5, 21.0, 14.2.

IR (neat) ν: 3294, 3061, 2930, 2860, 1716, 1670, 1615, 1580, 1521, 1486, 1455, 1370, 1306, 1263, 1223, 1199, 804, 758, 700, 668 cm^{-1}.

$[\alpha]_D^{20}$ = -0.41 (c = 2.0, $CHCl_3$).

HRMS (ESI$^+$): exact mass calculated for $[M+Na]^+$ ($C_{26}H_{34}N_2O_2Na$) requires m/z 429.2512, found m/z 429.2509.

Enantiomeric ratio = 91:9 was determined by chiral HPLC analysis: Chiralpak IC, *n*-heptane+0.1%IPA/IPA 80:20, 1 mL/min, 25 °C, detection at 230 nm, retention time (min): 32.9 (major) and 25.53 (minor).

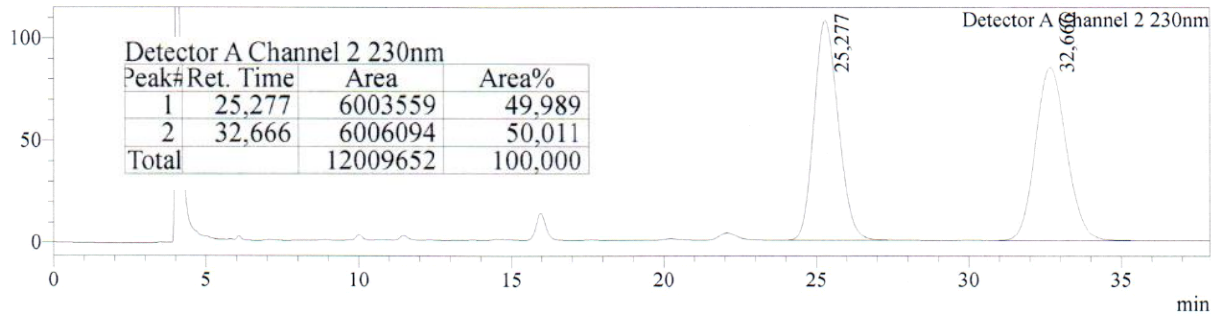

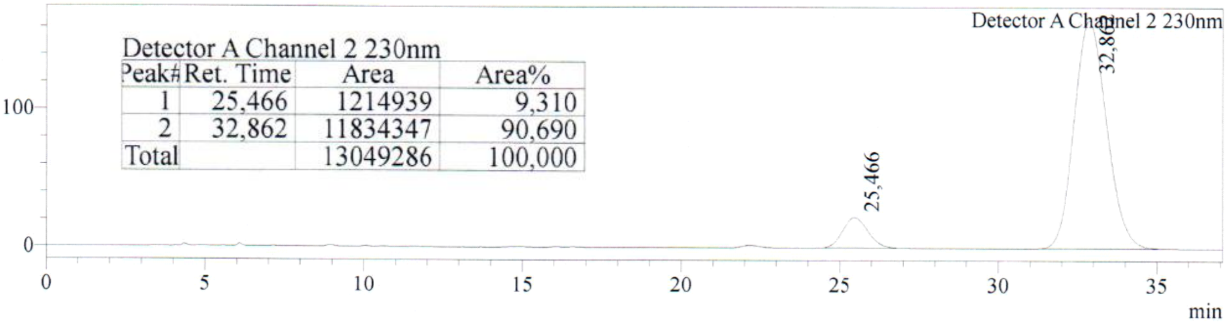

***N*-((2*S*,3*S*)-3-(Dimethylcarbamoyl)-2-phenylhexan-2-yl)benzamide (3n)**

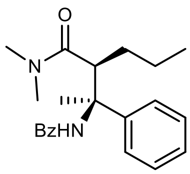

Prepared according to the general procedure in 44% yield (31 mg, 0.088 mmol) as a white foam (heptane/ethyl acetate 1:1, Rf = 0.32). The *d.r.* was determined by crude NMR to be >20: 1.

^{1}H NMR (600 MHz, $CDCl_3$) δ 9.07 (s, 1H), 7.99 (d, *J* = 7.2 Hz, 2H), 7.57 – 7.43 (m, 3H), 7.39 – 7.30 (m, 4H), 7.23 (ddd, *J* = 8.3, 5.6, 3.0 Hz, 1H), 3.22 (s, 3H), 3.08 (s, 3H), 2.93 (dd, *J* = 11.5, 3.2 Hz, 1H), 1.88 (s, 3H), 1.80 (ddd, *J* = 23.9, 11.3, 4.4 Hz, 1H), 1.19 – 1.11 (m, 1H), 1.09 – 1.00 (m, 1H), 0.97 – 0.89 (m, 1H), 0.73 (t, *J* = 7.2 Hz, 3H).

^{13}C NMR (150 MHz, $CDCl_3$) δ 175.6, 166.4, 142.5, 135.3, 131.1, 128.5 (2C), 127.9 (2C), 127.1 (2C), 126.5, 126.0 (2C), 60.8, 51.3, 38.4, 35.9, 30.7, 24.3, 20.7, 14.0.

IR (neat) ν: 3304, 3023, 2957, 2872, 1716, 1670, 1624, 1579, 1522, 1487, 1448, 1418, 1399, 1366, 1308, 1223, 759, 713, 699, 668, 530 cm^{-1}.

$[\alpha]_D^{20}$ = -0.52 (c = 2.0, $CHCl_3$).

HRMS (ESI$^+$): exact mass calculated for $[M+Na]^+$ ($C_{22}H_{28}N_2O_2Na$) requires m/z 375.2043, found m/z 375.2043.

Enantiomeric ratio = 91:9 was determined by chiral HPLC analysis: Chiralpak IC, *n*-heptane+0.1%IPA/EtOH 80:20, 1 mL/min, 25 °C, detection at 230 nm, retention time (min): 13.2 (major) and 9.6 (minor).

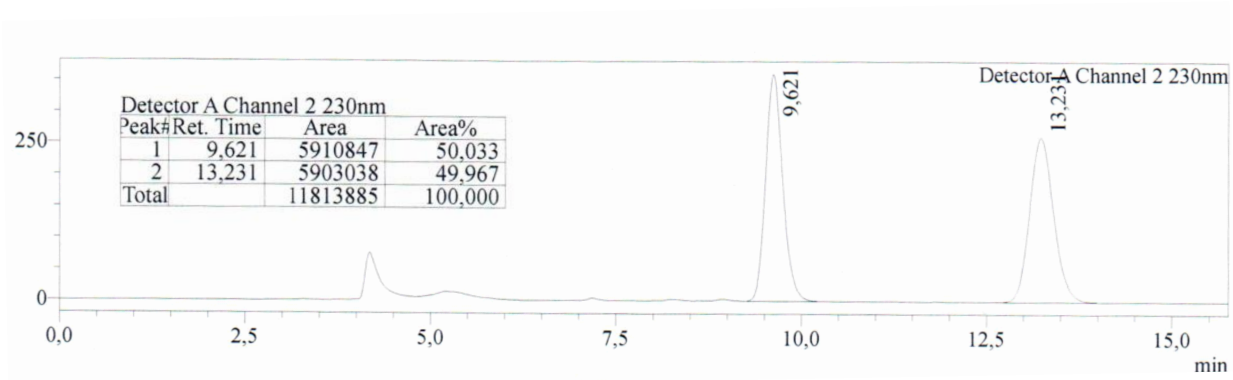

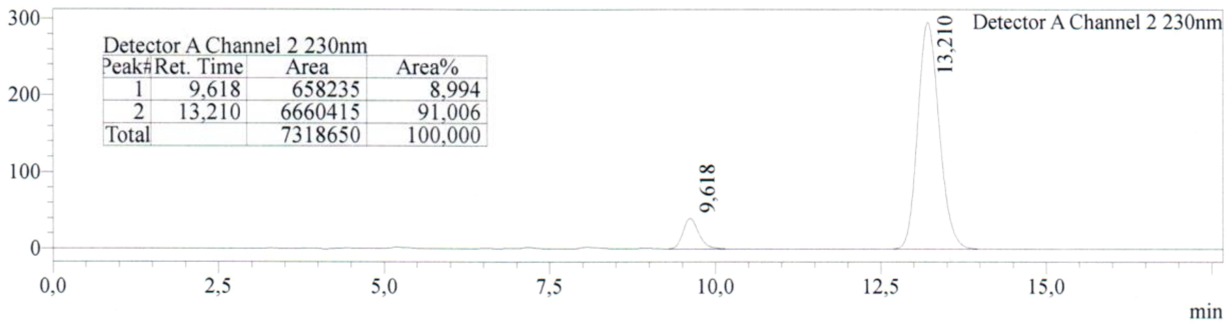

***N*-((2*S*,3*S*)-3-(Indoline-1-carbonyl)-2-phenylhexan-2-yl)benzamide (3o)**

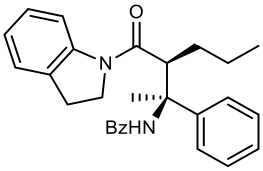

Prepared according to the general procedure in 53% yield (45 mg, 0.106 mmol, 84% brsm) as a yellow foam (heptane/ethyl acetate 1:1, Rf = 0.53). The *d.r.* was determined by crude NMR to be >20: 1.

^{1}H NMR (600 MHz, $CDCl_3$) δ 8.95 (s, 1H), 8.39 (d, *J* = 8.0 Hz, 1H), 8.11 – 7.98 (m, 2H), 7.55 – 7.47 (m, 3H), 7.42 – 7.33 (m, 4H), 7.25 (ddd, *J* = 9.4, 4.5, 3.0 Hz, 3H), 7.10 (td, *J* = 7.4, 1.0 Hz, 1H), 4.35 (td, *J* = 9.7, 7.4 Hz, 1H), 4.22 (td, *J* = 9.7, 7.3 Hz, 1H), 3.30 – 3.20 (m, 2H), 2.88 (dd, *J* = 11.5, 3.3 Hz, 1H), 1.99 (s, 3H), 1.92 (dtd, *J* = 13.1, 11.3, 4.5 Hz, 1H), 1.26 (d, *J* = 4.4 Hz, 1H), 1.14 (dddd, *J* = 13.2, 11.4, 5.3, 3.3 Hz, 1H), 1.06 – 0.97 (m, 1H), 0.76 (t, *J* = 7.3 Hz, 3H).

^{13}C NMR (150 MHz, $CDCl_3$) δ 174.0, 166.5, 142.3, 142.2, 135.3, 131.6, 131.2, 128.5 (2C), 128.0 (2C), 127.6, 127.2 (2C), 126.6, 126.0 (2C), 124.8, 124.6, 117.9, 61.1, 55.1, 49.4, 30.8, 27.9, 24.4, 20.9, 14.1.

IR (neat) ν: 2958, 1716, 1670, 1635, 1598, 1522, 1480, 1462, 1447, 1417, 1369, 1309, 1293, 1223, 756, 712, 701 cm^{-1}.

$[\alpha]_D^{20}$ = -0.35 (c = 2.0, $CHCl_3$).

HRMS (ESI$^+$): exact mass calculated for $[M+H]^+$ ($C_{28}H_{31}N_2O_2$) requires m/z 427.2380, found m/z 427.2386.

Enantiomeric ratio = 90:10 was determined by chiral HPLC analysis: Chiralpak IC, *n*-heptane+0.1%IPA/EtOH 80:20, 1 mL/min, 25 °C, detection at 210 nm, retention time (min): 29.2 (major) and 22.7 (minor).

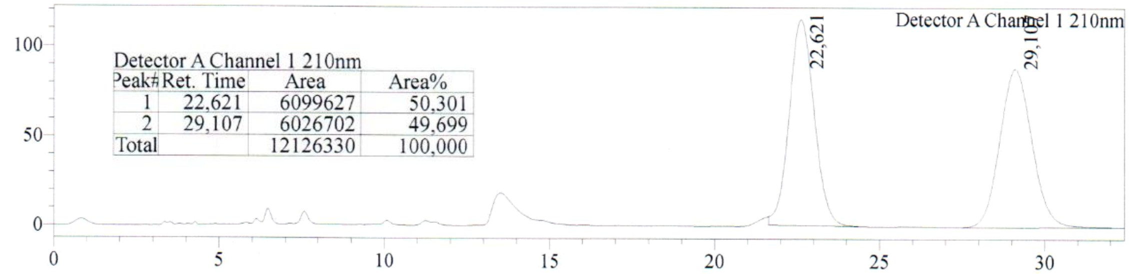

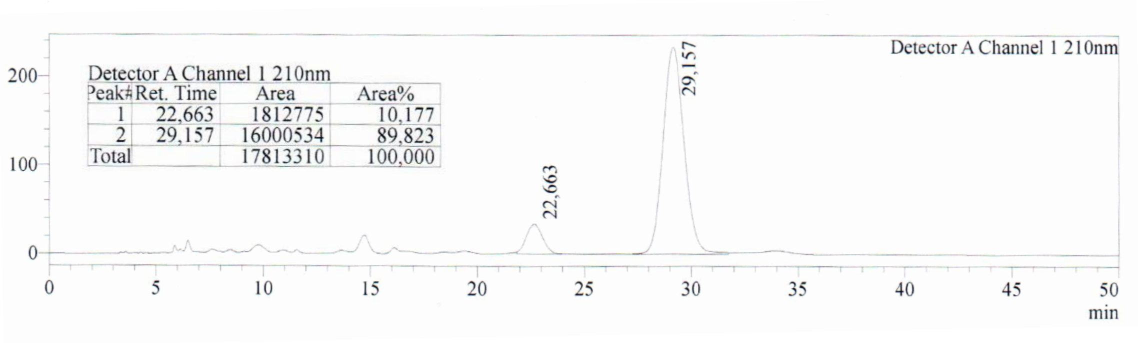

(3*S*,4*S*)-4-Benzamido-4-phenyl-3-(pyrrolidine-1-carbonyl)pentyl 2-(3-cyano-4-isobutoxyphenyl)-4-methylthiazole-5-carboxylate (3p)

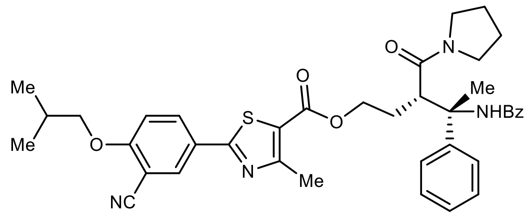

Prepared according to the general procedure in 52% (71 mg, 0.104 mmol) yield as a white foam (heptane/ethyl acetate 1:1, Rf = 0.25). The *d.r.* was determined by crude NMR as 12:1.

^{1}H NMR (600 MHz, $CDCl_3$) δ 8.95 (s, 1H), 8.16 (d, *J* = 2.2 Hz, 1H), 8.08 (dd, *J* = 8.8, 2.3 Hz, 1H), 8.00 – 7.96 (m, 2H), 7.50 (dd, *J* = 8.4, 6.1 Hz, 1H), 7.46 (dd, *J* = 11.4, 4.4 Hz, 2H), 7.33 (dt, *J* = 15.4, 7.7 Hz, 4H), 7.23 (t, *J* = 7.0 Hz, 1H), 7.03 (d, *J* = 8.9 Hz, 1H), 4.17 – 4.12 (m, 1H), 3.97 (d, *J* = 4.3 Hz, 1H), 3.91 (d, *J* = 6.5 Hz, 2H), 3.69 (dt, *J* = 14.0, 6.9 Hz, 1H), 3.63 – 3.50 (m, 3H), 2.97 (dd, *J* = 11.2, 2.7 Hz, 1H), 2.66 (s, 3H), 2.27 (ddt, *J* = 16.0, 11.3, 4.7 Hz, 1H), 2.21 (dt, *J* = 13.3, 6.6 Hz, 1H), 2.00 (dd, *J* = 12.7, 6.5 Hz, 1H), 1.95 (d, *J* = 4.9 Hz, 3H), 1.94 – 1.88 (m, 2H), 1.68 – 1.60 (m, 1H), 1.30 – 1.25 (m, 1H), 1.10 (s, 3H), 1.09 (s, 3H).

^{13}C NMR (150 MHz, $CDCl_3$) δ 172.6, 167.3, 166.6, 162.6, 161.5, 161.4, 141.9, 135.1, 132.5, 132.1, 131.3, 128.5 (2C), 128.1 (2C), 127.1 (2C), 126.8, 125.9 (2C), 125.8, 121.0, 115.3, 112.7, 103.0, 75.7, 63.3, 60.9, 51.0, 47.8, 46.1, 28.1, 27.5, 26.0, 24.3, 24.2, 19.0 (2C), 17.5.

IR (neat) ν: 2970, 2877, 1714, 1671, 1621, 1606, 1579, 1522, 1487, 1450, 1392, 1321, 1294, 1260, 1227, 1099, 1012, 759, 701 cm^{-1}.

$[\alpha]_D^{20}$ = 0.16 (c = 2.0, $CHCl_3$).

HRMS (ESI$^+$): exact mass calculated for $[M+H]^+$ ($C_{39}H_{43}N_4O_5S$) requires m/z 679.2949, found m/z 679.2946.

Enantiomeric ratio = 90:10 was determined by chiral HPLC analysis: Chiralpak IA-3, ACN, 0.7 mL/min, 25 °C, detection at 230 nm, retention time (min): 4.7 (major) and 5.5 (minor).

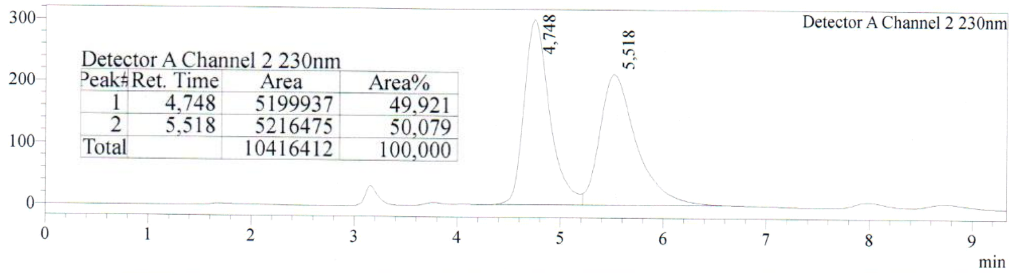

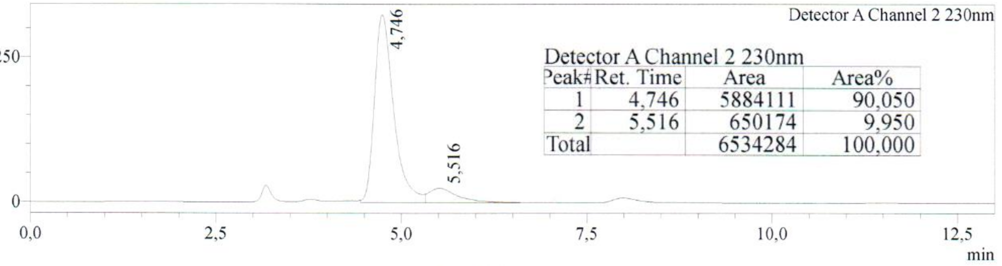

(3*S*,4*S*)-4-Benzamido-4-phenyl-3-(pyrrolidine-1-carbonyl)pentyl 2-(1-(4-chlorobenzoyl)-5-methoxy-2-methyl-1*H*-indol-3-yl)acetate (3q)

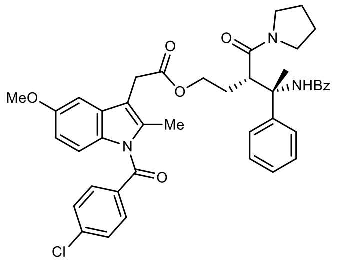

Prepared according to the general procedure in 50% yield (72 mg, 0.1 mmol) as a white foam (heptane/ethyl acetate 1:1, Rf = 0.22). The *d.r.* was determined by crude NMR as 10:1.

^{1}H NMR (600 MHz, $CDCl_3$) δ 9.00 (s, 1H), 7.96 (d, *J* = 7.1 Hz, 2H), 7.67 (d, *J* = 8.4 Hz, 2H), 7.50 – 7.46 (m, 3H), 7.44 (dd, *J* = 10.0, 4.7 Hz, 2H), 7.37 – 7.30 (m, 4H), 7.23 (ddd, *J* = 5.0, 4.2, 2.4 Hz, 1H), 6.91 (d, *J* = 2.4 Hz, 1H), 6.85 (d, *J* = 9.0 Hz, 1H), 6.69 (dd, *J* = 9.0, 2.5 Hz, 1H), 4.06 – 3.99 (m, 1H), 3.82 (s, 3H), 3.72 – 3.66 (m, 1H), 3.57 – 3.50 (m, 3H), 3.49 – 3.43 (m, 1H), 3.36 (dt, *J* = 9.5, 7.0 Hz, 1H), 3.07 (dt, *J* = 9.6, 7.3 Hz, 1H), 2.81 (dd, *J* = 11.7, 3.0 Hz, 1H), 2.32 (s, 3H), 2.13 (ddt, *J* = 15.9, 11.9, 4.2 Hz, 1H), 1.89 (s, 3H), 1.85 – 1.71 (m, 3H), 1.60 (dt, *J* = 19.8, 7.0 Hz, 1H), 1.51 – 1.43 (m, 1H).

^{13}C NMR (150 MHz, $CDCl_3$) δ 172.5, 170.2, 168.2, 166.5, 156.0, 141.9, 139.5, 136.0, 135.1, 133.6, 131.2, 131.1 (2C), 130.7, 130.5, 129.2, 128.5 (2C), 128.1 (2C), 127.1(2C), 126.7 (2C), 126.0 (2C), 114.8, 112.1, 111.2, 101.7, 62.9, 60.6, 55.7, 50.7, 47.3, 45.9, 30.2, 27.5, 25.8, 24.2, 24.2, 13.2.

IR (neat) ν: 3312, 2972, 2882, 1735, 1672, 1524, 1478, 1453, 1400, 1357, 1317, 1261, 1224, 1164, 1089, 1069, 1031, 1015, 926, 834, 804, 755, 702, 667, 570, 482 cm^{-1}.

$[\alpha]_D^{20}$ = -0.53 (c = 3.0, $CHCl_3$).

HRMS (ESI$^+$): exact mass calculated for $[M+Na]^+$ ($C_{42}H_{42}N_3{}^{35}ClO_6Na$) requires m/z 742.2654, found m/z 742.2648.

Enantiomeric ratio = 95:5 was determined by chiral HPLC analysis: Chiralpak IC, MeOH, 1 mL/min, 25 °C, detection at 230 nm, retention time (min): 12.9 (major) and 14.4 (minor).

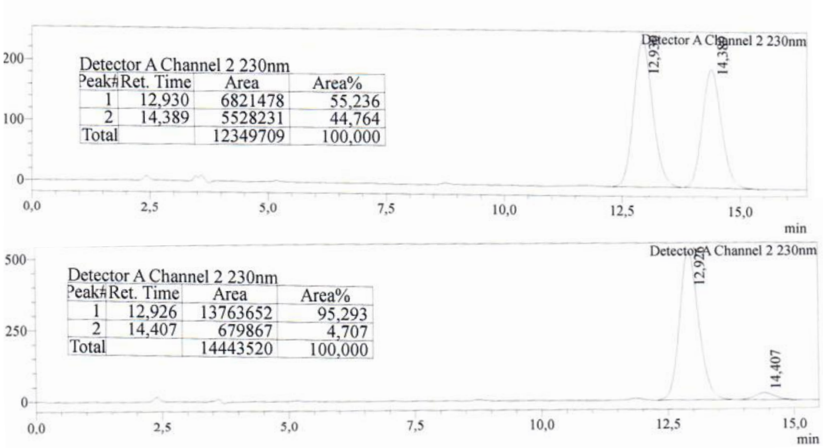

(*R*)-2,5,7,8-Tetramethyl-2-((4*R*,8*R*)-4,8,12-trimethyltridecyl)chroman-6-yl(3*S*,4*S*)-4-benzamido-4-phenyl-3-(pyrrolidine-1-carbonyl)pentanoate (3r)

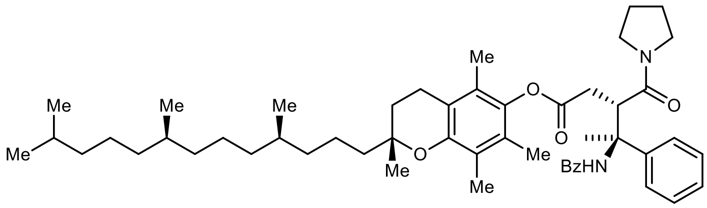

Prepared according to the general procedure in 41% yield (66 mg. 0.082 mmol) as a colorless oil (heptane/ethyl acetate 1:1, Rf = 0.56). The *d.r.* was determined by crude NMR to be >20: 1.

^{1}H NMR (600 MHz, CDCl$_3$) δ 9.04 (s, 1H), 8.06 – 7.98 (m, 2H), 7.55 – 7.47 (m, 3H), 7.44 – 7.36 (m, 4H), 7.29 (ddd, *J* = 8.5, 5.8, 2.5 Hz, 1H), 3.98 (dt, *J* = 9.7, 6.9 Hz, 1H), 3.69 (dt, *J* = 9.7, 7.0 Hz, 1H), 3.61 – 3.49 (m, 2H), 3.37 – 3.26 (m, 2H), 2.54 (br s, 2H), 2.34 (d, *J* = 14.6 Hz, 1H), 2.04 (s, 3H), 2.02 – 1.86 (m, 10H), 1.83 – 1.68 (m, 4H), 1.53 (dt, *J* = 13.2, 6.6 Hz, 3H), 1.46 – 1.19 (m, 15H), 1.18 – 1.02 (m, 7H), 0.89 – 0.84 (m, 12H).

^{13}C NMR (150 MHz, CDCl$_3$) δ 172.3, 171.4, 166.6, 149.5, 141.5, 140.1, 135.0, 131.3, 128.8, 128.6(2C), 128.4(2C), 127.9, 127.2(2C), 127.0, 126.0(2C), 123.0, 117.3, 75.0, 60.4, 49.7, 47.8, 46.2, 39.3, 37.4 (3C), 37.4, 37.2, 33.3, 32.8, 32.6, 31.0, 30.7, 27.9, 25.9, 24.8, 24.4, 23.9, 22.7, 22.6, 20.9, 20.5 (2C), 19.7, 19.6, 13.0, 12.2, 11.8.

IR (neat) ν: 2927, 2869, 1748, 1716, 1675, 1622, 1524, 1487, 1452, 1374, 1224, 1153, 760, 700 cm^{-1}.

$[\alpha]_D^{20}$ = -0.14 (c = 2.0, CHCl$_3$).

HRMS (ESI$^+$): exact mass calculated for $[M+H]^+$ ($C_{52}H_{75}N_2O_5$) requires m/z 807.5670, found m/z 807.5678.

***N*-((2*S*,3*S*)-2-(4-Fluorophenyl)-3-(pyrrolidine-1-carbonyl)hexan-2-yl)benzamide (3s)**

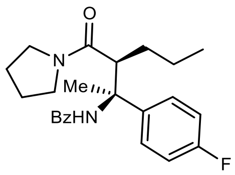

Prepared according to the general procedure in 58% yield (46 mg, 0.12 mmol) as a white foam (heptane/ethyl acetate 1:1, Rf = 0.38). The *d.r.* was determined by crude NMR as 12:1.

^{1}H NMR (600 MHz, CDCl$_3$) δ 9.16 (s, 1H), 8.00 – 7.95 (m, 2H), 7.48 (ddd, *J* = 6.2, 3.6, 1.4 Hz, 1H), 7.47 – 7.42 (m, 2H), 7.29 (dd, *J* = 8.9, 5.2 Hz, 2H), 7.00 (t, *J* = 8.7 Hz, 2H), 3.67 (dt, *J* = 9.8, 6.9 Hz, 1H), 3.62 – 3.53 (m, 3H), 2.64 (dd, *J* = 11.6, 3.3 Hz, 1H), 2.02 (tq, *J* = 12.1, 5.9 Hz, 2H), 1.97 – 1.91 (m, 2H), 1.88 (d, *J* = 5.5 Hz, 3H), 1.84 – 1.76 (m, 1H), 1.23 – 1.14 (m, 1H), 1.01 (dddd, *J* = 12.8, 10.8, 5.3, 3.3 Hz, 1H), 0.97 – 0.90 (m, 1H), 0.75 (t, *J* = 7.2 Hz, 3H).

^{13}C NMR (150 MHz, CDCl$_3$) δ 173.7, 166.5, 161.4 (d, $^{1}J_{C\text{-}F}$ = 244.9 Hz), 138.2 (d, $^{4}J_{C\text{-}F}$ = 3.6 Hz), 135.1, 131.2, 128.5 (2C), 127.6 (d, $^{3}J_{C\text{-}F}$ = 8.4 Hz, 2C), 127.1 (2C), 114.7 (d, $^{2}J_{C\text{-}F}$ = 21.4 Hz, 2C), 60.4, 54.4, 47.8, 45.8, 30.5, 26.1, 24.4, 24.3, 20.7, 14.0.

^{19}F NMR (377 MHz, CDCl$_3$) δ -117.1.

IR (neat) ν: 2959, 2874, 1716, 1669, 1619, 1579, 1522, 1510, 1487, 1453, 1368, 1340, 1310, 1226, 836, 759, 712, 587 cm^{-1}.

[α]$_D^{20}$ = -0.30 (c = 1.0, CHCl$_3$).

HRMS (ESI$^+$): exact mass calculated for [M+H]$^+$ ($C_{24}H_{30}N_2O_2F$) requires m/z 397.2286, found m/z 397.2287.

Enantiomeric ratio = 97:3 was determined by chiral HPLC analysis: Chiralpak IC, *n*-heptane+0.1%IPA/EtOH 80:20, 1 mL/min, 25 °C, detection at 230 nm, retention time (min): 10.4 (major) and 9.1 (minor).

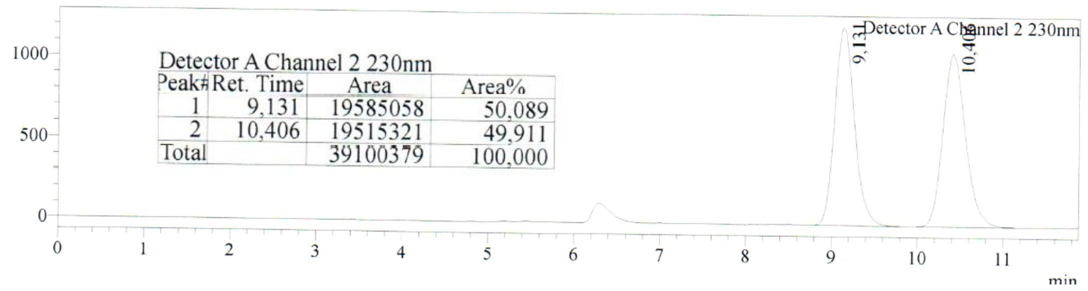

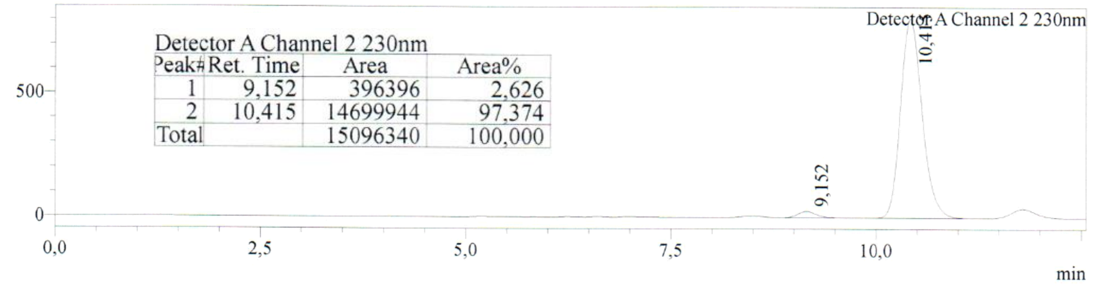

***N*-((2*S*,3*S*)-2-(4-Chlorophenyl)-3-(pyrrolidine-1-carbonyl)hexan-2-yl)benzamide (3t)**

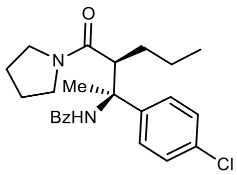

Prepared according to the general procedure in 62% yield (51 mg, 0.124 mmol) as a white foam (heptane/ethyl acetate 1:1, Rf = 0.38). The *d.r.* was determined by crude NMR as 14:1.

^{1}H NMR (600 MHz, CDCl$_3$) δ 9.16 (s, 1H), 7.97 (d, *J* = 7.0 Hz, 2H), 7.52 – 7.47 (m, 1H), 7.47 – 7.41 (m, 2H), 7.33 – 7.28 (m, 2H), 7.28 – 7.23 (m, 2H), 3.67 (dt, *J* = 9.7, 6.9 Hz, 1H), 3.63 – 3.53 (m, 3H), 2.63 (dd, *J* = 11.6, 3.3 Hz, 1H), 2.10 – 1.98 (m, 2H), 1.98 – 1.90 (m, 2H), 1.87 (s, 3H), 1.85 – 1.76 (m, 1H), 1.23 – 1.14 (m, 1H), 1.04 – 0.91 (m, 2H), 0.76 (t, *J* = 7.2 Hz, 3H).

^{13}C NMR (150 MHz, CDCl$_3$) δ 173.6, 166.5, 141.2, 135.0, 132.3, 131.3, 128.5 (2C), 128.1 (2C), 127.6 (2C), 127.1 (2C), 60.4, 54.2, 47.8, 45.9, 30.5, 26.1, 24.4, 24.3, 20.8, 14.1.

IR (neat) ν: 3300, 2959, 2931, 2874, 1668, 1619, 1579, 1521, 1489, 1453, 1443, 1399, 1371, 1311, 1227, 1187, 1095, 1012, 911, 830, 753, 713, 584, 574 cm^{-1}.

$[\alpha]_D^{20}$ = -0.38 (c = 3.0, CHCl$_3$).

HRMS (ESI$^+$): exact mass calculated for $[M+Na]^+$ ($C_{24}H_{29}N_2O_2{}^{35}ClNa$) requires m/z 435.1810, found m/z 435.1810.

Enantiomeric ratio = 93:7 was determined by chiral HPLC analysis: Chiralpak IC, *n*-heptane+0.1%IPA/IPA 85:15, 1 mL/min, 25 °C, detection at 230 nm, retention time (min): 10.0 (major) and 11.1 (minor).

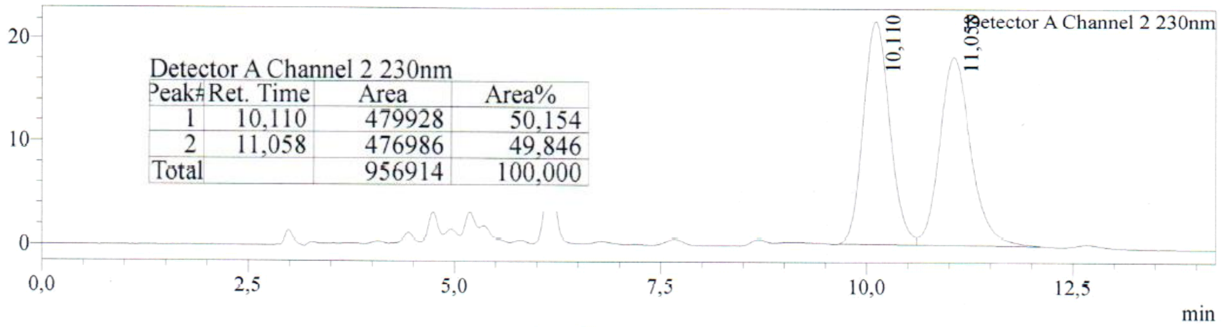

Detector A Channel 2 230nm

Peak#	Ret. Time	Area	Area%
1	10,110	479928	50,154
2	11,058	476986	49,846
Total		956914	100,000

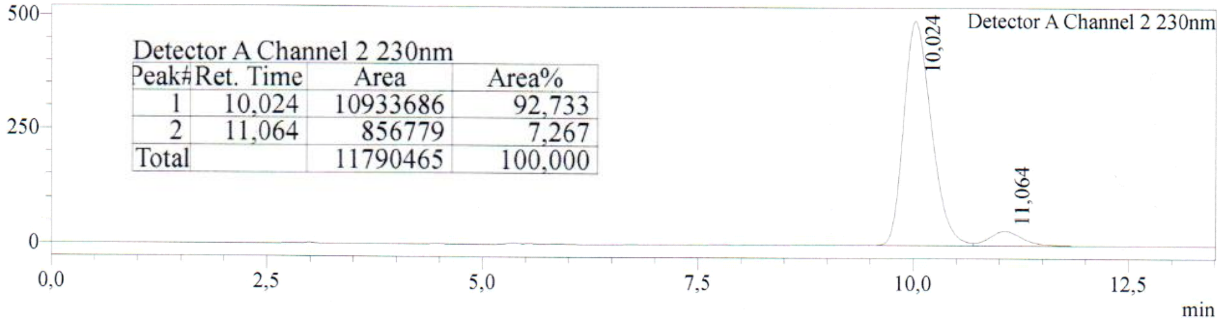

Detector A Channel 2 230nm

Peak#	Ret. Time	Area	Area%
1	10,024	10933686	92,733
2	11,064	856779	7,267
Total		11790465	100,000

***N*-((2*S*,3*S*)-2-(4-Bromophenyl)-3-(pyrrolidine-1-carbonyl)hexan-2-yl)benzamide (3u)**

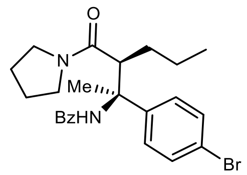

Prepared according to the general procedure in 67% yield (61 mg, 0.134 mmol) as a yellow foam (heptane/ethyl acetate 1:1, Rf = 0.38). The *d.r.* was determined by crude NMR as 17:1.

^{1}H NMR (700 MHz, $CDCl_3$) δ 9.16 (s, 1H), 8.02 – 7.93 (m, 2H), 7.53 – 7.41 (m, 5H), 7.20 (d, *J* = 8.6 Hz, 2H), 3.67 (dt, *J* = 9.6, 6.9 Hz, 1H), 3.64 – 3.53 (m, 3H), 2.63 (dd, *J* = 11.7, 3.3 Hz, 1H), 2.03 (tq, *J* = 12.7, 6.3 Hz, 2H), 1.98 – 1.92 (m, 2H), 1.87 (s, 3H), 1.80 (ddd, *J* = 23.9, 11.4, 4.5 Hz, 1H), 1.19 (tdd, *J* = 12.2, 7.3, 4.5 Hz, 1H), 1.04 – 0.98 (m, 1H), 0.98 – 0.90 (m, 1H), 0.76 (t, *J* = 7.3 Hz, 3H).

^{13}C NMR (175 MHz, $CDCl_3$) δ 173.6, 166.5, 141.7, 135.0, 131.3 (2C), 131.0 (2C), 128.5 (2C), 127.9, 127.1 (2C), 120.5, 60.4, 54.1, 47.8, 45.9, 30.5, 26.1, 24.4, 24.2, 20.7, 14.1.

IR (neat) ν: 3299, 2959, 2874, 1667, 1618, 1603, 1579, 1521, 1487, 1453, 1442, 1395, 1371, 1310, 1255, 1187, 825, 735, 698, 573 cm^{-1}.

$[\alpha]_D^{20}$ = -0.09 (c = 1.0, $CHCl_3$).

HRMS (ESI$^+$): exact mass calculated for $[M+H]^+$ ($C_{24}H_{30}BrN_2O_2$) requires m/z 459.1465, found m/z 459.1468.

Enantiomeric ratio = 90:10 was determined by chiral HPLC analysis: Lux-Cellulose 1, *n*-heptane+0.1%IPA/IPA 80:20, 1 mL/min, 25 °C, detection at 230 nm, retention time (min): 8.0 (major) and 8.9 (minor).

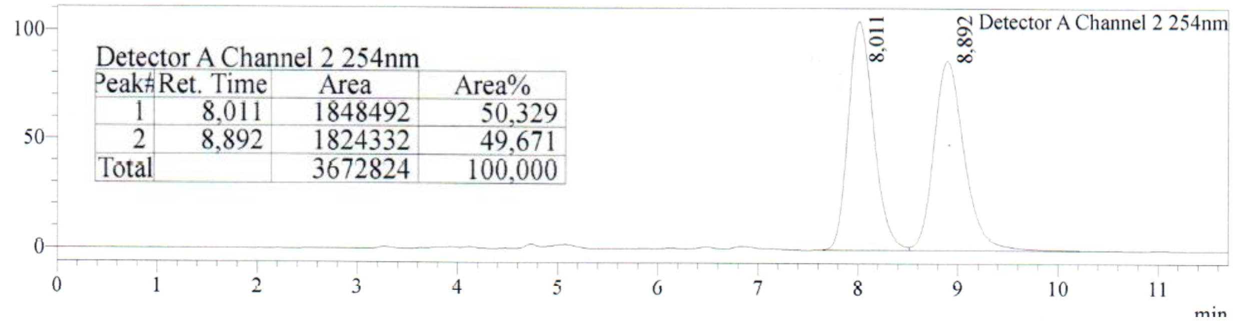

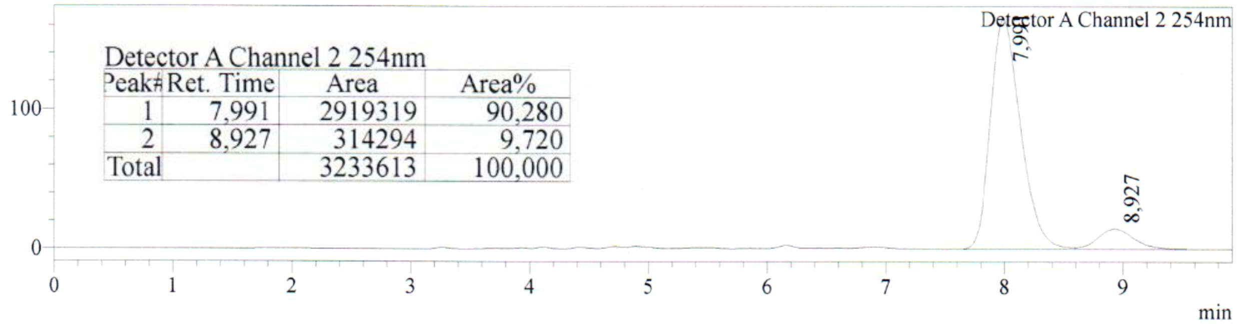

***N*-((2*S*,3*S*)-2-(4-Iodophenyl)-3-(pyrrolidine-1-carbonyl)hexan-2-yl)benzamide (3v)**

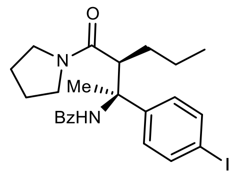

Prepared according to the general procedure in 72% yield (73 mg, 0.144 mmol) as a yellow foam (heptane/ethyl acetate 1:1, Rf = 0.38). The *d.r.* was determined by crude NMR as 15:1.

^{1}H NMR (700 MHz, $CDCl_3$) δ 9.16 (s, 1H), 8.01 – 7.94 (m, 2H), 7.64 (d, *J* = 8.6 Hz, 2H), 7.51 – 7.43 (m, 3H), 7.08 (d, *J* = 8.6 Hz, 2H), 3.67 (dt, *J* = 9.6, 6.9 Hz, 1H), 3.62 – 3.53 (m, 3H), 2.62 (dd, *J* = 11.7, 3.3 Hz, 1H), 2.03 (tq, *J* = 12.6, 6.3 Hz, 2H), 1.97 – 1.91 (m, 2H), 1.86 (s, 3H), 1.79 (ddd, *J* = 23.8, 11.4, 4.4 Hz, 1H), 1.22 – 1.15 (m, 1H), 1.05 – 0.99 (m, 1H), 0.98 – 0.91 (m, 1H), 0.76 (t, *J* = 7.2 Hz, 3H).

^{13}C NMR (175 MHz, $CDCl_3$) δ 173.6, 166.5, 142.4, 137.0 (2C), 134.9, 131.3 (2C), 128.6 (2C), 128.2, 127.1(2C), 92.2, 60.5, 54.1, 47.8, 45.9, 30.5, 26.1, 24.4, 24.2, 20.7, 14.1.

IR (neat) ν: 3294, 2969, 2929, 2873, 1738, 1667, 1618, 16002, 1580, 1522, 1486, 1452, 1390, 1341, 1310, 1277, 1190, 1004, 751, 712, 697, 667, 572 cm^{-1}.

$[\alpha]_D^{20}$ = -0.064 (c = 1.0, $CHCl_3$).

HRMS (ESI$^+$): exact mass calculated for $[M+H]^+$ ($C_{24}H_{30}IN_2O_2$) requires m/z 459.1465, found m/z 459.1468.

Enantiomeric ratio = 96:4 was determined by chiral HPLC analysis: Lux-Cellulose 1, *n*-heptane+0.1%IPA/IPA 80:20, 1 mL/min, 25 °C, detection at 230 nm, retention time (min): 8.2 (major) and 9.7 (minor).

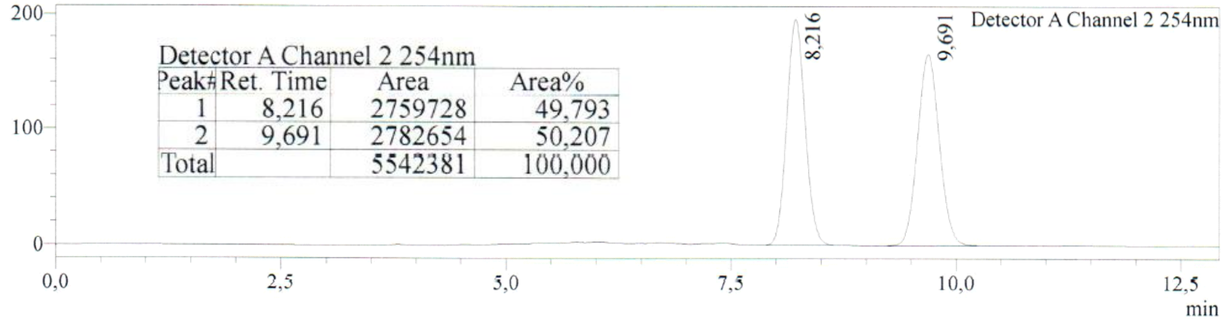

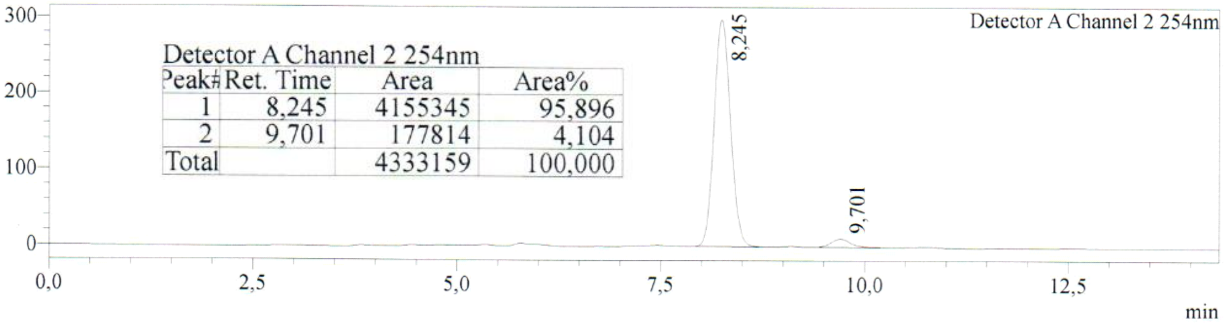

***N*-((2*S*,3*S*)-3-(Pyrrolidine-1-carbonyl)-2-(4-(trifluoromethyl)phenyl)hexan-2-yl)benzamide (3w)**

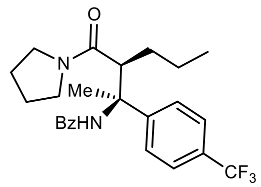

Prepared according to the general procedure in 60% yield (53 mg, 0.12 mmol) as a white solid (heptane/ethyl acetate 1:1, Rf = 0.35). The *d.r.* was determined by crude NMR as 18:1.

^{1}H NMR (600 MHz, $CDCl_3$) δ 9.23 (s, 1H), 8.02 – 7.94 (m, 2H), 7.58 (d, *J* = 8.3 Hz, 2H), 7.54 – 7.40 (m, 5H), 3.68 (dt, *J* = 9.5, 6.9 Hz, 1H), 3.65 – 3.51 (m, 3H), 2.67 (dd, *J* = 11.7, 3.0 Hz, 1H), 2.11 – 1.99 (m, 2H), 1.99 – 1.89 (m, 5H), 1.89 – 1.77 (m, 1H), 1.23 – 1.14 (m, 1H), 0.95 (ddd, *J* = 11.9, 5.6, 2.7 Hz, 2H), 0.75 (t, *J* = 7.2 Hz, 3H).

^{13}C NMR (150 MHz, $CDCl_3$) δ 173.4, 166.6, 146.7, 134.8, 131.4, 128.7 (q, $^{2}J_{C-F}$ = 33.0 Hz, 2C), 128.5 (2C), 127.1 (2C), 126.5, 125.0 (q, $^{3}J_{C-F}$ = 3.0 Hz, 2C), 124.2 (q, $^{1}J_{C-F}$ = 270.0 Hz), 60.6, 54.0, 47.8, 45.9, 30.4, 26.1, 24.4, 24.3, 20.7, 14.01.

^{19}F NMR (565 MHz, $CDCl_3$) δ -62.38.

IR (neat) ν: 3020, 2969, 2877, 1716, 1670, 1619, 1522, 1483, 1454, 1365, 1328, 1219, 1165, 1119, 1069, 764, 713, 668 cm^{-1}.

$[\alpha]_D^{20}$ = -0.56 (c = 2.0, $CHCl_3$).

HRMS (ESI$^+$): exact mass calculated for $[M+H]^+$ ($C_{25}H_{30}F_3N_2O_2$) requires m/z 447.2254, found m/z 447.2251.

Enantiomeric ratio = 95:5 was determined by chiral HPLC analysis: Chiralpak IC, *n*-heptane+0.1%IPA/IPA 80:20, 1 mL/min, 25 °C, detection at 230 nm, retention time (min): 22.7 (major) and 14.5 (minor).

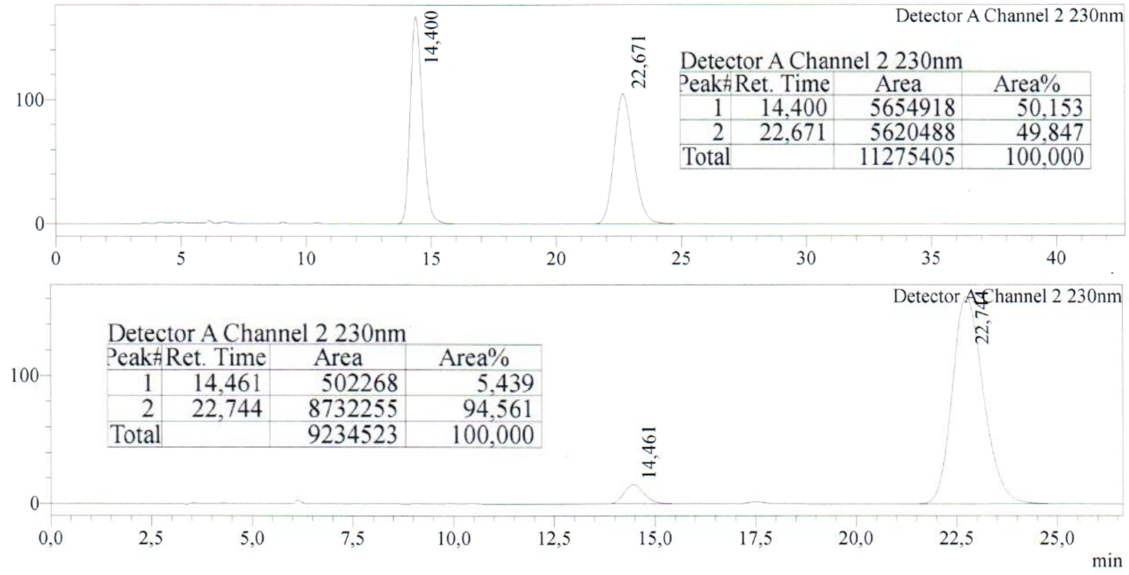

***N*-((2S,3S)-3-(Pyrrolidine-1-carbonyl)-2-(p-tolyl)hexan-2-yl)benzamide (3x)**

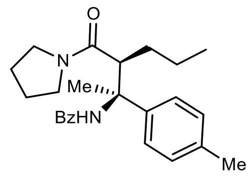

Prepared according to the general procedure in 65% yield (51 mg, 0.13 mmol) as a white foam (heptane/ethyl acetate 1:1, Rf = 0.38). The *d.r.* was determined by crude NMR as 18:1.

^{1}H NMR (600 MHz, $CDCl_3$) δ 9.09 (s, 1H), 7.99 (d, *J* = 6.9 Hz, 2H), 7.53 – 7.42 (m, 3H), 7.21 (d, *J* = 8.2 Hz, 2H), 7.12 (d, *J* = 8.1 Hz, 2H), 3.69 (dt, *J* = 9.7, 6.9 Hz, 1H), 3.64 – 3.53 (m, 3H), 2.67 (dd, *J* = 11.6, 3.3 Hz, 1H), 2.32 (s, 3H), 2.02 (tq, *J* = 12.5, 6.3 Hz, 2H), 1.97 – 1.91 (m, 2H), 1.88 (s, 3H), 1.80 (ddd, *J* = 24.1, 11.4, 4.4 Hz, 1H), 1.23 – 1.14 (m, 1H), 1.13 – 1.04 (m, 1H), 0.94 (ddd, *J* = 17.9, 9.9, 5.4 Hz, 1H), 0.75 (t, *J* = 7.2 Hz, 3H).

^{13}C NMR (150 MHz, $CDCl_3$) δ 174.0, 166.4, 139.4, 135.9, 131.1, 128.6 (2C), 128.4 (2C), 127.2 (2C), 125.9 (2C), 60.5, 54.4, 47.8, 45.8, 31.8, 30.5, 26.1, 24.4, 24.3, 20.9, 20.8, 14.1.

IR (neat) ν: 3300, 2959, 2929, 2873, 1669, 1618, 1579, 1520, 1486, 1452, 1442, 1370, 1310, 1227, 1188, 1030, 910, 819, 753, 710, 587, 576 cm^{-1}.

$[\alpha]_D^{20}$ = -0.48 (c = 2.0, $CHCl_3$).

HRMS (ESI$^+$): exact mass calculated for $[M+Na]^+$ ($C_{25}H_{32}N_2O_2Na$) requires m/z 415.2356, found m/z 415.2356.

Enantiomeric ratio = 99.9:0.1 was determined by chiral HPLC analysis: Chiralpak IC, *n*-heptane+0.1%IPA/EtOH 85:15, 1 mL/min, 25 °C, detection at 230 nm, retention time (min): 21.8 (major) and 20.2 (minor).

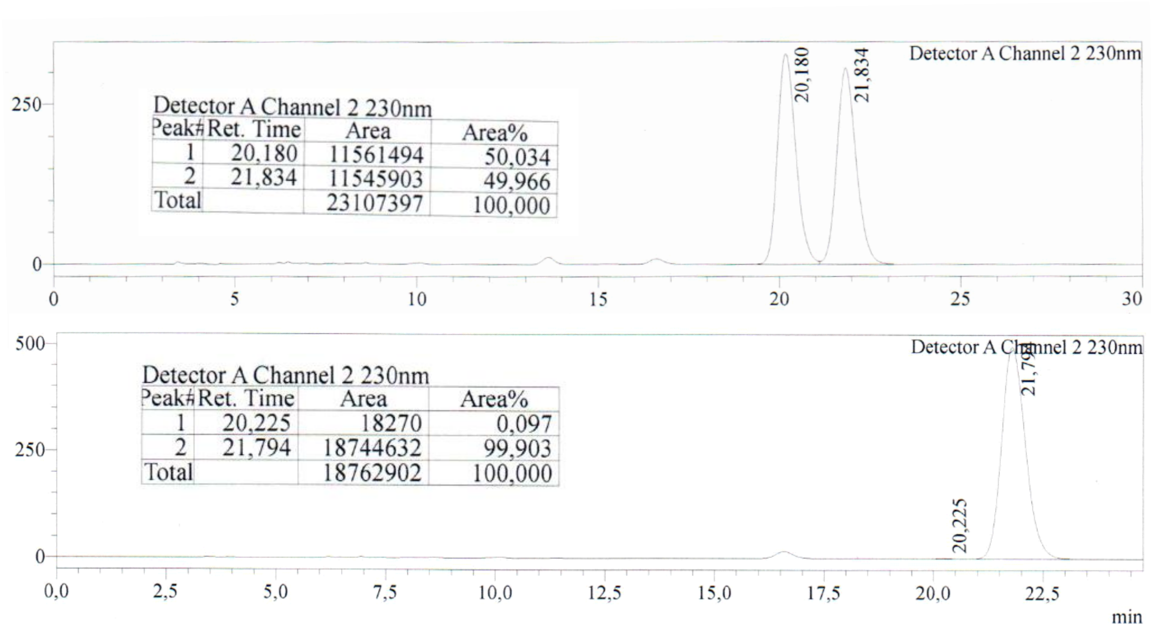

N-((2S,3S)-2-([1,1'-Biphenyl]-4-yl)-3-(pyrrolidine-1-carbonyl)hexan-2-yl)benzamide (3y)

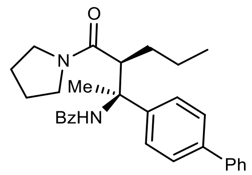

Prepared according to the general procedure in 60% yield (55 mg, 0.12 mmol) as a white foam (heptane/ethyl acetate 1:1, Rf = 0.38). The *d.r.* was determined by crude NMR as 8:1.

^{1}H NMR (600 MHz, $CDCl_3$) δ 9.17 (s, 1H), 8.02 (dd, *J* = 8.2, 1.4 Hz, 2H), 7.61 (dd, *J* = 8.3, 1.1 Hz, 2H), 7.57 (d, *J* = 8.5 Hz, 2H), 7.54 – 7.45 (m, 3H), 7.45 – 7.38 (m, 4H), 7.37 – 7.30 (m, 1H), 3.72 (dt, *J* = 9.7, 6.9 Hz, 1H), 3.67 – 3.54 (m, 3H), 2.73 (dd, *J* = 11.6, 3.3 Hz, 1H), 2.09 – 1.99 (m, 2H), 1.99 – 1.91 (m, 5H), 1.89 – 1.80 (m, 1H), 1.21 (tdd, *J* = 11.6, 7.3, 4.4 Hz, 1H), 1.16 – 1.07 (m, 1H), 0.97 (ddt, *J* = 17.8, 7.1, 5.3 Hz, 1H), 0.77 (t, *J* = 7.2 Hz, 3H).

^{13}C NMR (150 MHz, $CDCl_3$) δ 173.9, 166.5, 141.6, 140.8, 139.1, 135.2, 131.2, 128.6 (2C), 128.5 (2C), 127.2 (2C), 127.1, 127.0 (2C), 126.6 (2C), 126.5 (2C), 60.6, 54.3, 47.8, 45.8, 30.6, 26.1, 24.4, 24.3, 20.8, 14.1.

IR (neat) ν: 3028, 2958, 2873, 1716, 1670, 1619, 1523, 1487, 1452, 1368, 1340, 1311, 1225, 840, 765, 700, 668 cm^{-1}.

$[\alpha]_D^{20}$ = 0.031 (c = 2.0, $CHCl_3$).

HRMS (ESI$^+$): exact mass calculated for $[M+Na]^+$ ($C_{30}H_{34}N_2O_2Na$) requires m/z 477.2512, found m/z 477.2511.

Enantiomeric ratio = 92:8 was determined by chiral HPLC analysis: Chiralpak IC, *n*-heptane+0.1%IPA/EtOH 80:20, 1 mL/min, 25 °C, detection at 230 nm, retention time (min): 14.9 (major) and 19.6 (minor).

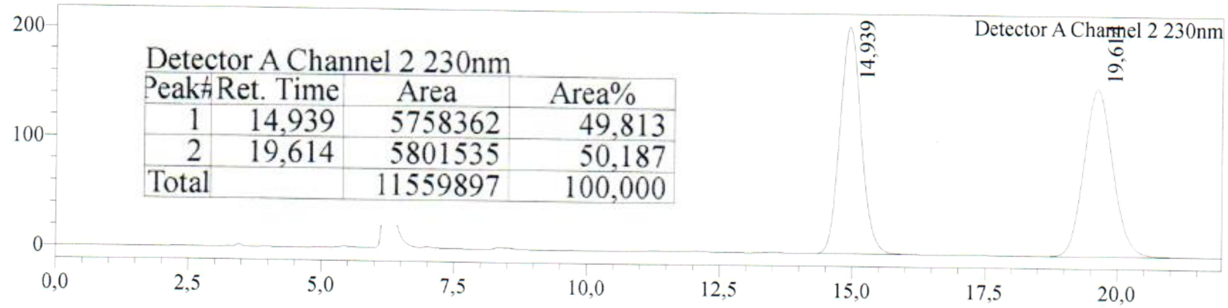

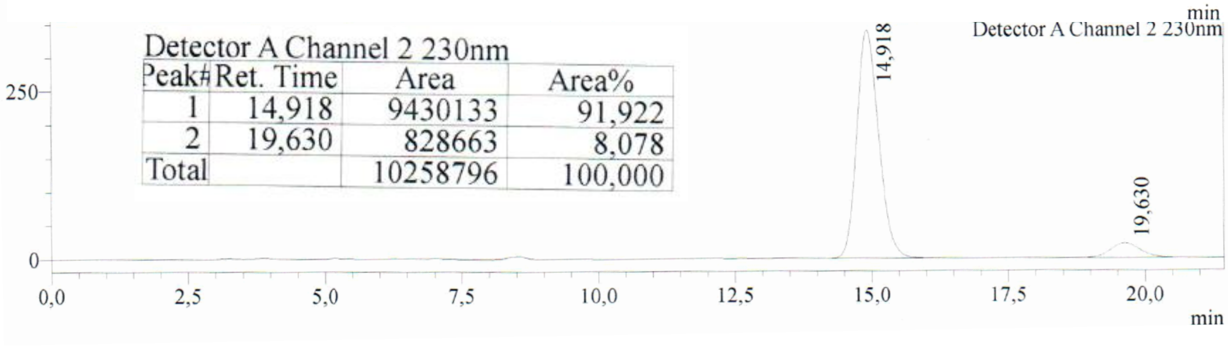

***N*-((2*S*,3*S*)-3-(Pyrrolidine-1-carbonyl)-2-(m-tolyl)hexan-2-yl)benzamide (3z)**

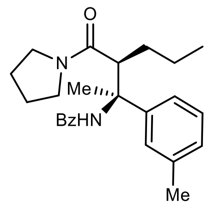

Prepared according to the general procedure in 65% yield (51 mg, 0.13 mmol) as a yellow foam (heptane/ethyl acetate 1:1, Rf = 0.38). The *d.r.* was determined by crude NMR to be >20:1.

^{1}H NMR (600 MHz, $CDCl_3$) δ 9.07 (s, 1H), 8.03 – 7.95 (m, 2H), 7.52 – 7.41 (m, 3H), 7.20 (t, *J* = 7.6 Hz, 1H), 7.17 – 7.08 (m, 2H), 7.03 (d, *J* = 7.4 Hz, 1H), 3.69 (dt, *J* = 9.6, 6.9 Hz, 1H), 3.63 – 3.53 (m, 3H), 2.68 (dd, *J* = 11.6, 3.3 Hz, 1H), 2.35 (s, 3H), 2.09 – 1.97 (m, 2H), 1.97 – 1.91 (m, 2H), 1.90 (d, *J* = 7.9 Hz, 3H), 1.79 (ddd, *J* = 24.1, 11.4, 4.4 Hz, 1H), 1.23 – 1.13 (m, 1H), 1.06 (tdd, *J* = 13.0, 5.0, 3.5 Hz, 1H), 0.94 (ddd, *J* = 17.9, 9.8, 5.6 Hz, 1H), 0.75 (t, *J* = 7.2 Hz, 3H).

^{13}C NMR (150 MHz, $CDCl_3$) δ 173.9, 166.4, 142.3, 137.3, 135.4, 131.0, 128.4 (2C), 127.7, 127.3, 127.1 (2C), 126.4, 123.4, 60.6, 54.1, 47.8, 45.8, 30.5, 26.1, 24.4, 24.3, 21.8, 20.7, 14.0.

IR (neat) ν: 2958, 2874, 1716, 1670, 1619, 1579, 1487, 1453, 1368, 1339, 1311, 1225, 756, 707 cm^{-1}.

$[\alpha]_D^{20}$ = -0.74 (c = 2.0, $CHCl_3$).

HRMS (ESI^+): exact mass calculated for $[M+Na]^+$ ($C_{25}H_{32}N_2O_2Na$) requires m/z 415.2356, found m/z 415.2358.

Enantiomeric ratio = 98:2 was determined by chiral HPLC analysis: Chiralpak IC, *n*-heptane+0.1%IPA/EtOH 80:20, 1 mL/min, 25 °C, detection at 230 nm, retention time (min): 12.6 (major) and 10.5 (minor).

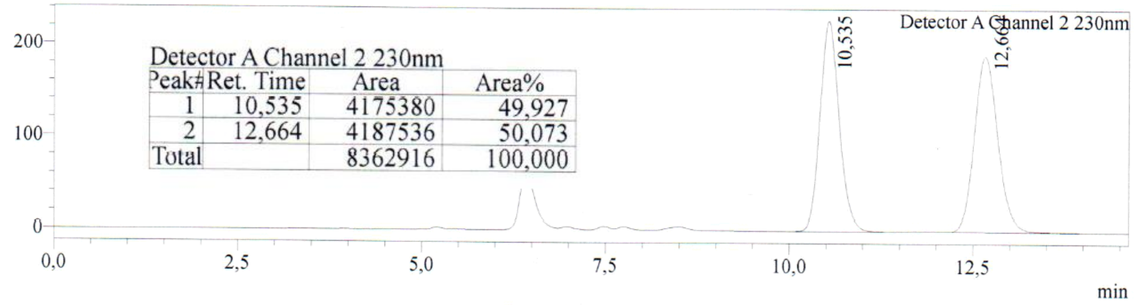

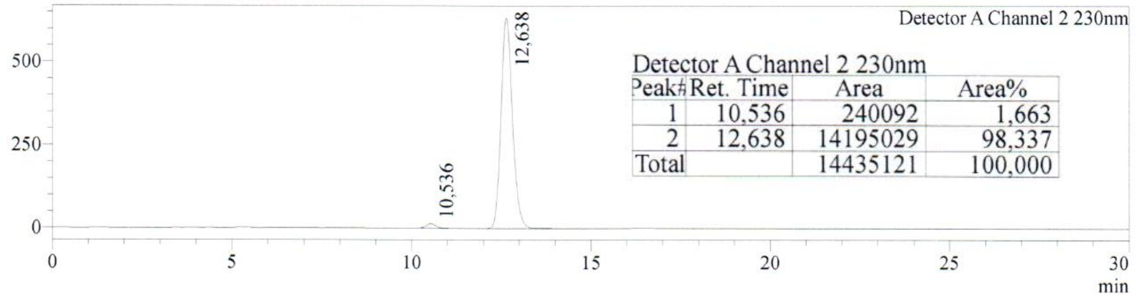

***N*-((2*S*,3*S*)-2-(3-Methoxyphenyl)-3-(pyrrolidine-1-carbonyl)hexan-2-yl)benzamide (3aa)**

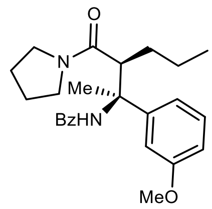

Prepared according to the general procedure in 53% yield (43 mg, 0.106 mmol) as a yellow foam (heptane/ethyl acetate 1:1, Rf = 0.25). The *d.r.* was determined by crude NMR as 8:1.

^{1}H NMR (600 MHz, $CDCl_3$) δ 9.08 (s, 1H), 8.00 – 7.95 (m, 2H), 7.51 – 7.41 (m, 3H), 7.24 (t, *J* = 8.0 Hz, 1H), 6.94 (ddd, *J* = 7.9, 1.7, 0.7 Hz, 1H), 6.90 – 6.87 (m, 1H), 6.77 (ddd, *J* = 8.2, 2.5, 0.6 Hz, 1H), 3.79 (s, 3H), 3.67 (dt, *J* = 9.8, 6.8 Hz, 1H), 3.62 – 3.52 (m, 3H), 2.68 (dd, *J* = 11.6, 3.4 Hz, 1H), 2.06 – 1.97 (m, 2H), 1.96 – 1.90 (m, 2H), 1.88 (s, 3H), 1.82 – 1.75 (m, 1H), 1.21 – 1.13 (m, 1H), 1.07 (dddd, *J* = 13.0, 11.3, 5.2, 3.4 Hz, 1H), 0.94 (dddd, *J* = 12.5, 10.8, 7.2, 5.4 Hz, 1H), 0.75 (t, *J* = 7.2 Hz, 3H).

^{13}C NMR (150 MHz, $CDCl_3$) 173.8, 166.4, 159.3, 144.3, 135.3, 131.1, 128.8, 128.4 (2C), 127.1 (2C), 118.8, 113.0, 110.8, 60.6, 55.2, 54.2, 47.8, 45.8, 30.5, 26.1, 24.4, 24.3, 20.7, 14.1.

IR (neat) ν: 3017, 2970, 2958, 1716, 1671, 1618, 1579, 1522, 1487, 1453, 1365, 1224, 762, 668, 530, 422 cm^{-1}.

$[\alpha]_D^{20}$ = -0.63 (c = 2.0, $CHCl_3$).

HRMS (ESI$^+$): exact mass calculated for $[M+Na]^+$ ($C_{25}H_{32}N_2O_3Na$) requires m/z 431.2305, found m/z 431.2302.

Enantiomeric ratio = 93:7 was determined by chiral HPLC analysis: Chiralpak IC, *n*-heptane+0.1%IPA/EtOH 80:20, 1 mL/min, 25 °C, detection at 230 nm, retention time (min): 18.7 (major) and 22.4 (minor).

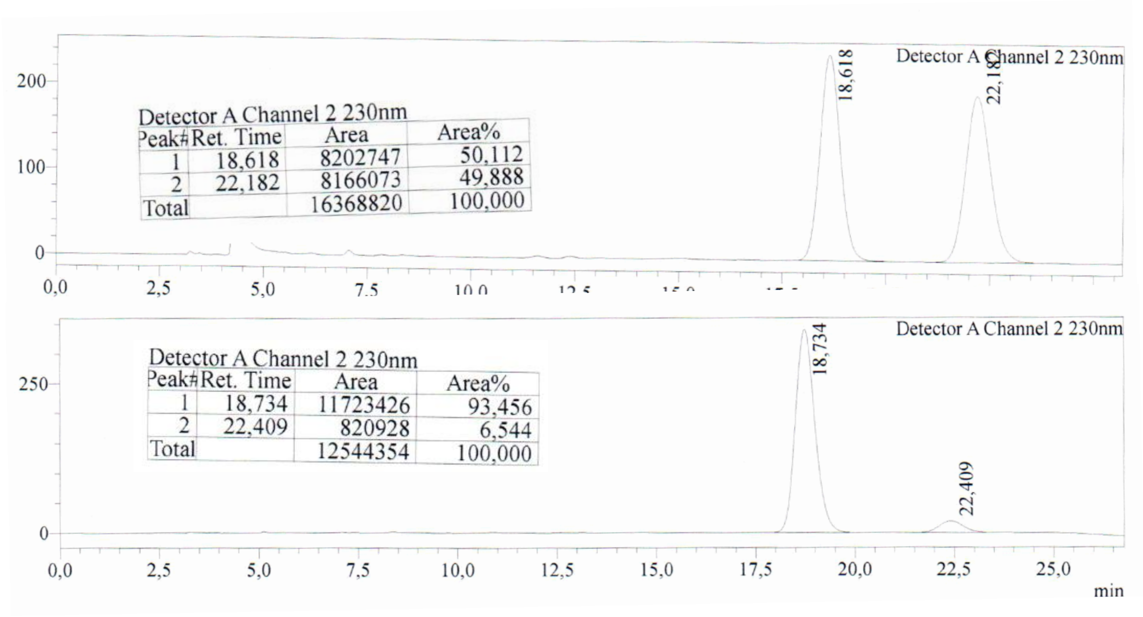

***N*-((2S,3S)-3-(pyrrolidine-1-carbonyl)-2-(o-tolyl)hexan-2-yl)benzamide (3ab)**

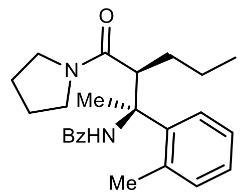

Prepared according to the general procedure in 43% yield (34 mg, 0.086 mmol) as a yellow foam (heptane/ethyl acetate 1:1, Rf = 0.40). The *d.r.* was determined by crude NMR to be >20:1.

^{1}H NMR (700 MHz, $CDCl_3$) δ 9.23 (s, 1H), 7.99 (d, *J* = 7.1 Hz, 2H), 7.51 – 7.42 (m, 3H), 7.37 (d, *J* = 7.4 Hz, 1H), 7.18 – 7.09 (m, 3H), 3.71 (dd, *J* = 16.4, 7.1 Hz, 1H), 3.64 – 3.54 (m, 3H), 2.96 (s, 1H), 2.52 (s, 3H), 2.09 – 1.98 (m, 5H), 1.94 (p, *J* = 6.8 Hz, 2H), 1.91 – 1.85 (m, 1H), 1.24 – 1.16 (m, 1H), 1.09 (s, 1H), 0.97 (qd, *J* = 12.6, 6.5 Hz, 1H), 0.76 (t, *J* = 7.2 Hz, 3H).

^{13}C NMR (150 MHz, $CDCl_3$) δ 173.9, 166.6, 140.2*, 135.4, 133.0, 133.0*, 131.1, 128.5 (2C), 128.3*, 127.2 (2C), 126.8, 125.8, 62.0, 47.7, 45.9, 31.0, 26.1, 26.0, 24.4, 23.5, 20.9, 14.1.

*Due to steric hindrance, three aromatic carbon signals are not visible in the ^{13}C NMR spectrum. However, the signals can be visualized by analysis of the HSQC and HMBC spectra (see page S113).

IR (neat) ν: 2959, 2874, 1716, 1670, 1618, 1578, 1524, 1486, 1452, 1369, 1340, 1225, 756, 712 cm^{-1}.

$[\alpha]_D^{20}$ = -0.36 (c = 1.0, $CHCl_3$).

HRMS (ESI$^+$): exact mass calculated for $[M+Na]^+$ ($C_{25}H_{32}N_2O_2Na$) requires m/z 415.2356, found m/z 415.2355.

Enantiomeric ratio = 99.5:0.5 was determined by chiral HPLC analysis: Lux-Cellulose 1, *n*-heptane+0.1%IPA/IPA 80:20, 1 mL/min, 25 °C, detection at 254 nm, retention time (min): 9.7 (major) and 6.8 (minor).

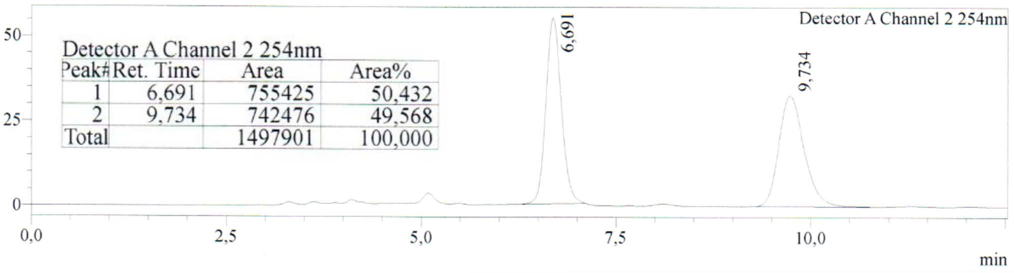

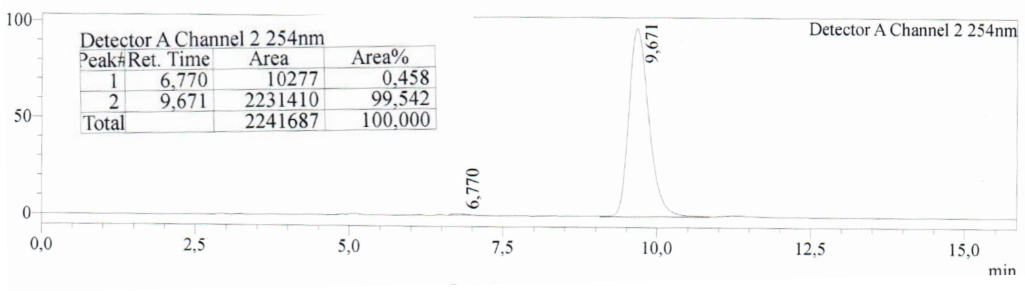

***N*-((2S,3S)-2-(Naphthalen-2-yl)-3-(pyrrolidine-1-carbonyl)hexan-2-yl)benzamide (3ac)**

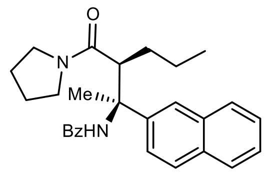

Prepared according to the general procedure in 72% yield (62 mg, 0.144 mmol) as a yellow foam (heptane/ethyl acetate 1:1, Rf = 0.40). The *d.r.* was determined by crude NMR as 13:1.

^{1}H NMR (700 MHz, $CDCl_3$) δ 9.25 (s, 1H), 8.03 (d, *J* = 7.0 Hz, 2H), 7.84 – 7.75 (m, 4H), 7.53 – 7.41 (m, 6H), 3.76 (dt, *J* = 9.4, 6.9 Hz, 1H), 3.67 – 3.57 (m, 3H), 2.79 (dd, *J* = 11.7, 3.3 Hz, 1H), 2.09 – 2.00 (m, 5H), 1.99 – 1.92 (m, 2H), 1.87 (ddd, *J* = 24.4, 11.5, 4.4 Hz, 1H), 1.18 (tdd, *J* = 11.8, 7.3, 4.5 Hz, 1H), 1.09 – 1.04 (m, 1H), 0.93 – 0.86 (m, 1H), 0.70 (t, *J* = 7.3 Hz, 3H).

^{13}C NMR (175 MHz, $CDCl_3$) δ 173.9, 166.6, 140.4, 135.3, 133.3, 132.3, 131.2, 128.5 (2C), 128.2, 127.4, 127.3, 127.2 (2C), 125.7, 125.6, 124.9, 124.4, 60.8, 54.6, 47.9, 45.9, 30.6, 26.1, 24.5, 24.4, 20.7, 14.0.

IR (neat) ν: 3296, 2969, 2930, 2873, 1737, 1629, 1619, 1580, 1524, 1485, 1453, 1443, 1371, 1311, 1228, 1191, 749, 709, 668 cm^{-1}.

$[\alpha]_D^{20}$ = -0.12 (c = 1.0, $CHCl_3$).

HRMS (ESI^+): exact mass calculated for $[M+Na]^+$ ($C_{28}H_{32}N_2O_2Na$) requires m/z 451.2356, found m/z 451.2356.

Enantiomeric ratio = 96:4 was determined by chiral HPLC analysis: Chiralpak IC, *n*-heptane+0.1%IPA/EtOH 80:20, 1 mL/min, 25 °C, detection at 254 nm, retention time (min): 8.2 (major) and 9.7 (minor).

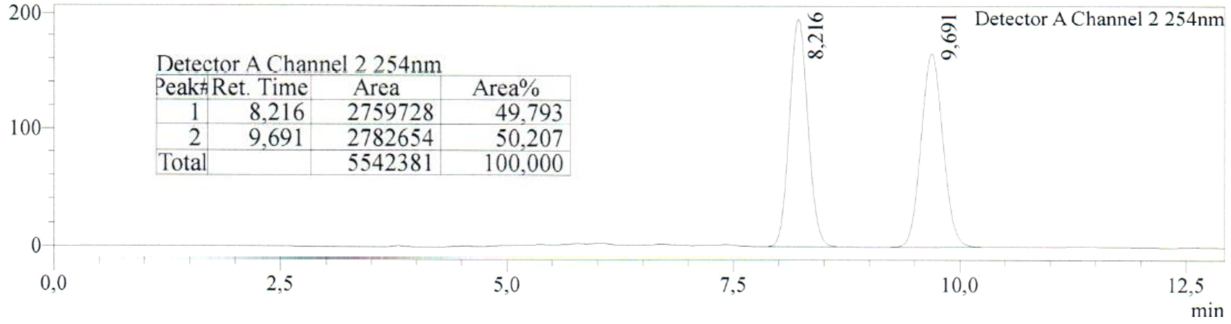

Detector A Channel 2 254nm

Peak#	Ret. Time	Area	Area%
1	8,216	2759728	49,793
2	9,691	2782654	50,207
Total		5542381	100,000

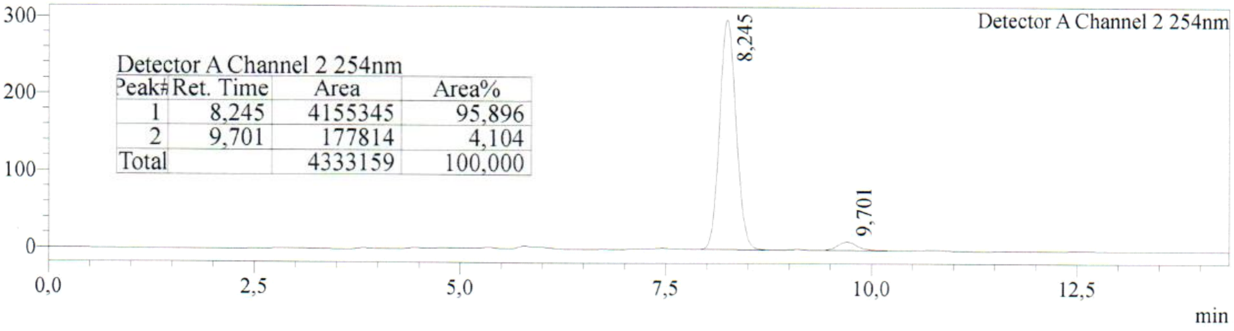

Detector A Channel 2 254nm

Peak#	Ret. Time	Area	Area%
1	8,245	4155345	95,896
2	9,701	177814	4,104
Total		4333159	100,000

***N*-((3*S*,4*S*)-3-Phenyl-4-(pyrrolidine-1-carbonyl)heptan-3-yl)benzamide (3ad)**

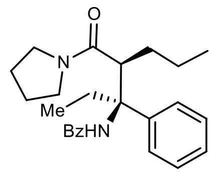

Prepared according to the general procedure in 29% yield (23 mg, 0.058 mmol) as a yellow oil (heptane/ethyl acetate 1:1, Rf = 0.45). The *d.r.* was determined by crude NMR as 12:1.

^{1}H NMR (600 MHz, $CDCl_3$) δ 9.09 (s, 1H), 8.03 (d, *J* = 6.7 Hz, 2H), 7.54 – 7.44 (m, 3H), 7.30 (d, *J* = 4.6 Hz, 4H), 7.21 (dt, *J* = 5.4, 3.7 Hz, 1H), 3.67 (dt, *J* = 13.6, 6.8 Hz, 1H), 3..48-3.59 (m, 3H), 2.83 (app. dq, *J* = 15.6, 7.8 Hz, 1H), 2.75 (dd, *J* = 11.5, 3.1 Hz, 1H), 2.15 (app. dq, *J* = 14.3, 7.1 Hz, 1H), 2.00 (p, *J* = 6.7 Hz, 2H), 1.92 (p, *J* = 6.7 Hz, 2H), 1.77 – 1.67 (m, 1H), 1.32 – 1.26 (m, 1H), 1.20 – 1.12 (m, 1H), 0.97 – 0.91 (m, 1H), 0.86 (t, *J* = 7.5 Hz, 3H), 0.71 (t, *J* = 7.2 Hz, 3H).

^{13}C NMR (150 MHz, $CDCl_3$) δ 174.3, 166.6, 143.2, 135.2, 131.2, 128.5 (2C), 127.8 (2C), 127.2 (2C), 126.4, 126.1 (2C), 64.3, 53.1, 47.8, 46.0, 32.1, 31.9, 26.1, 24.3, 20.3, 14.1, 9.0.

IR (neat) ν: 3298, 2961, 2928, 2874, 1737, 1671, 1619, 1523, 1487, 1451, 1444, 1340, 1311, 1226, 1090, 1030, 754, 700, 584, 572 cm^{-1}.

$[\alpha]_D^{20}$ = -0.23 (c = 1.0, $CHCl_3$).

HRMS (ESI$^+$): exact mass calculated for $[M+Na]^+$ ($C_{25}H_{32}N_2O_2Na$) requires m/z 415.2356, found m/z 415.2356.

Enantiomeric ratio = 99.8:0.2 was determined by chiral HPLC analysis: Chiralpak IC, *n*-heptane+0.1%IPA/IPA 85:15, 1 mL/min, 25 °C, detection at 230 nm, retention time (min): 23.0 (major) and 11.8 (minor).

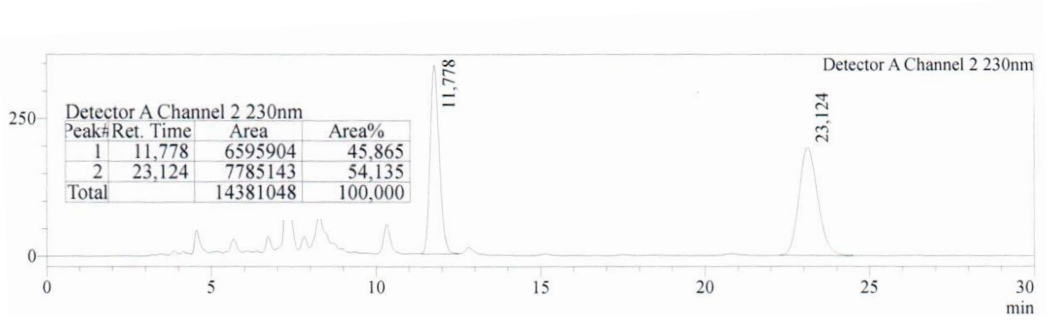

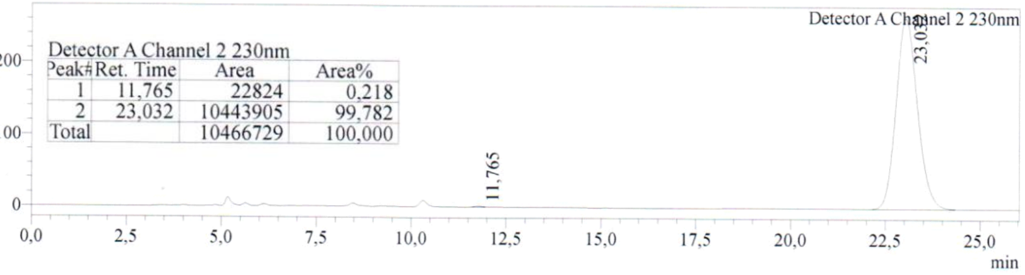

***N*-((2S,3S)-3-(Pyrrolidine-1-carbonyl)-2-(thiophen-3-yl)hexan-2-yl)benzamide (3ae)**

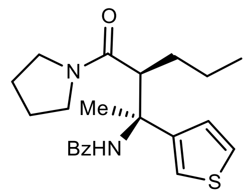

Prepared according to the general procedure in 51% yield (39 mg, 0.102 mmol) as a white foam (heptane/ethyl acetate 1:1, Rf = 0.32). The *d.r.* was determined by crude NMR to be >20:1.

^{1}H NMR (600 MHz, $CDCl_3$) δ 9.08 (s, 1H), 8.01 – 7.92 (m, 2H), 7.50 – 7.46 (m, 1H), 7.44 (t, *J* = 7.3 Hz, 2H), 7.23 (dd, *J* = 5.0, 3.0 Hz, 1H), 7.04 (dd, *J* = 2.9, 1.2 Hz, 1H), 6.99 (dd, *J* = 5.0, 1.2 Hz, 1H), 3.64 (dt, *J* = 9.6, 6.9 Hz, 1H), 3.61 – 3.50 (m, 3H), 2.73 (dd, *J* = 11.5, 3.0 Hz, 1H), 2.06 – 1.97 (m, 2H), 1.97 – 1.89 (m, 2H), 1.89 – 1.81 (m, 4H), 1.24 – 1.14 (m, 2H), 1.03 – 0.95 (m, 1H), 0.79 (t, *J* = 7.1 Hz, 3H).

^{13}C NMR (150 MHz, $CDCl_3$) δ 173.8, 166.5, 144.9, 135.3, 131.1, 128.4 (2C), 127.1 (2C), 126.9, 124.6, 120.2, 59.0, 54.2, 47.7, 45.8, 30.8, 26.1, 24.9, 24.4, 20.8, 14.1.

IR (neat) ν: 2959, 2873, 1716, 1669, 1619, 1579, 1521, 1487, 1453, 1368, 1313, 1226, 758, 712, 666 cm^{-1}.

$[\alpha]_D^{20}$ = -0.63 (c = 2.0, $CHCl_3$).

HRMS (ESI$^+$): exact mass calculated for $[M+Na]^+$ ($C_{22}H_{28}N_2O_2SNa$) requires m/z 407.1764, found m/z 407.1762.

Enantiomeric ratio = 92:8 was determined by chiral HPLC analysis: Lux-Cellulose 1, *n*-heptane+0.1%IPA/IPA 75:25, 1 mL/min, 25 °C, detection at 230 nm, retention time (min): 8.8 (major) and 7.5 (minor).

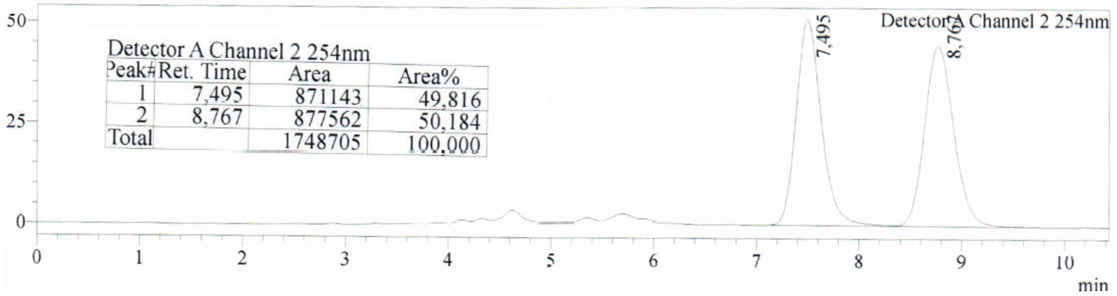

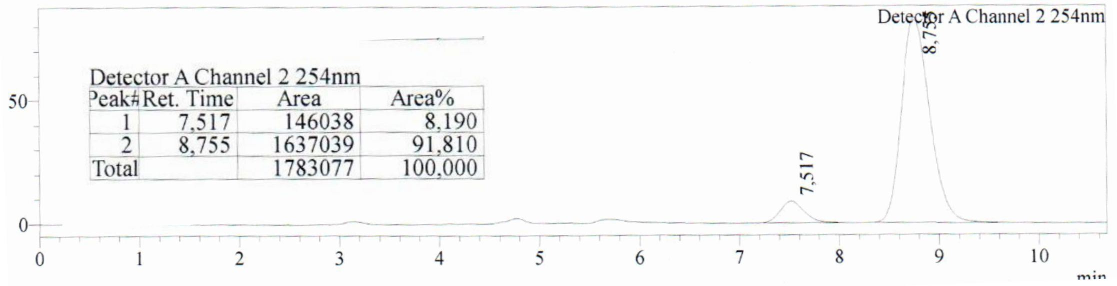

(*S*)-*N*-(4-Propyl-5-(pyrrolidine-1-carbonyl)octan-4-yl)benzamide (3af)

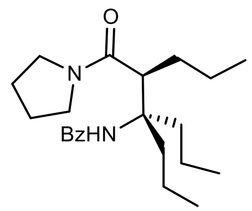

Prepared according to the general procedure in 44% yield (33 mg, 0.088 mmol) as a colorless oil (heptane/ethyl acetate 1:1, Rf = 0.44).

^{1}H NMR (600 MHz, $CDCl_3$) δ 8.67 (s, 1H), 7.89 (dd, *J* = 7.8, 1.4 Hz, 2H), 7.46 – 7.36 (m, 3H), 3.58 – 3.44 (m, 4H), 3.01 – 2.92 (m, 1H), 2.78 (dd, *J* = 11.6, 3.2 Hz, 1H), 2.04 – 1.92 (m, 2H), 1.92 – 1.76 (m, 5H), 1.59 (tdd, *J* = 13.8, 5.5, 3.4 Hz, 1H), 1.52 – 1.46 (m, 1H), 1.46 – 1.39 (m, 1H), 1.37 – 1.23 (m, 4H), 1.17 (ddd, *J* = 13.3, 9.6, 5.0 Hz, 1H), 0.98 (t, *J* = 7.2 Hz, 3H), 0.92 (t, *J* = 7.3 Hz, 3H), 0.83 (t, *J* = 7.2 Hz, 3H).

^{13}C NMR (150 MHz, $CDCl_3$) δ 174.8, 166.8, 136.1, 130.7, 128.4 (2C), 126.9 (2C), 61.5, 48.1, 47.6, 45.7, 40.8, 36.0, 30.7, 26.1, 24.3, 20.8, 17.6, 17.2, 14.8, 14.7, 14.4.

IR (neat) ν: 2958, 2932, 2872, 1716, 1666, 1622, 1602, 1579, 1531, 1488, 1453, 1441, 1636, 1339, 1226, 756, 706 cm^{-1}.

$[\alpha]_D^{20}$ = -0.40 (c = 2.0, $CHCl_3$).

HRMS (ESI$^+$): exact mass calculated for $[M+Na]^+$ ($C_{23}H_{36}N_2O_2Na$) requires m/z 395.2669, found m/z 395.2668.

Enantiomeric ratio = 88:12 was determined by chiral HPLC analysis: Chiralpak IC, *n*-heptane+0.1%IPA/IPA 85:15, 1 mL/min, 25 °C, detection at 254 nm, retention time (min): 13.9 (major) and 11.1 (minor).

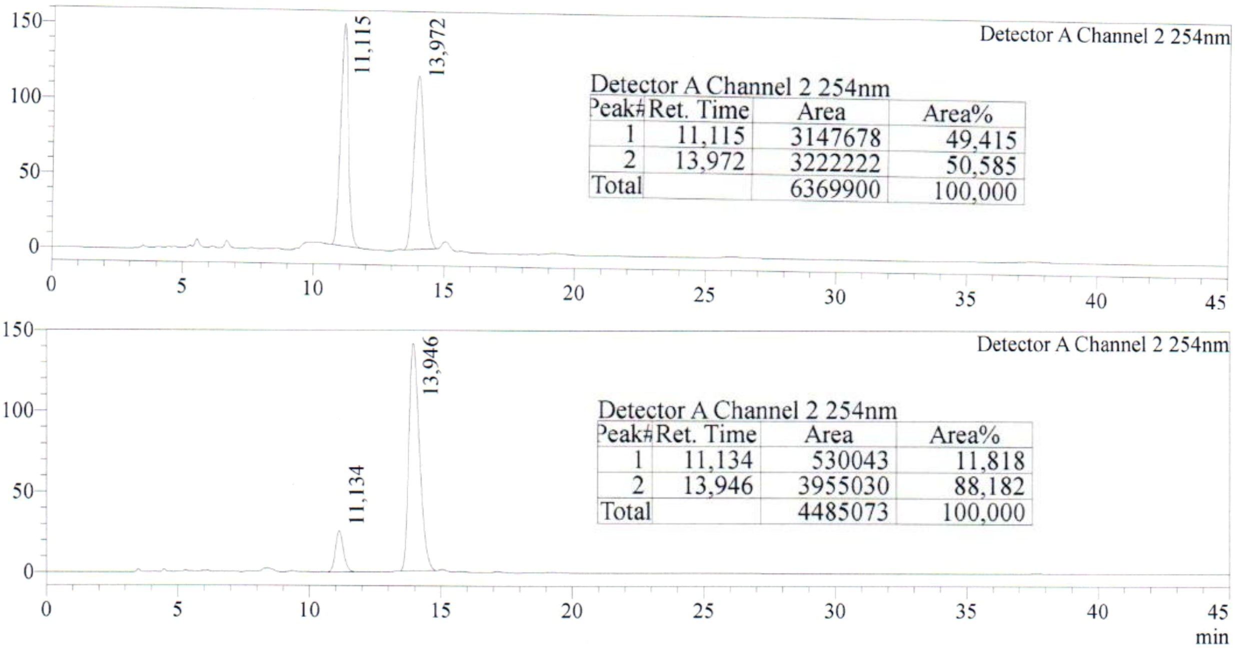

(*S*)-*N*-(1-(1-oxo-1-(pyrrolidin-1-yl)pentan-2-yl)cyclopentadecyl)benzamide (3ag)

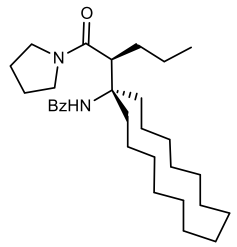

Prepared according to the general procedure in 45% yield (43 mg, 0.09 mmol) as a colorless oil (heptane/ethyl acetate 1:1, Rf = 0.62).

^{1}H NMR (600 MHz, CDCl$_3$) δ 8.47 (s, 1H), 7.93 – 7.87 (m, 2H), 7.41 (d, *J* = 7.4 Hz, 3H), 3.63 – 3.47 (m, 4H), 3.13 – 3.03 (m, 1H), 2.84 (dd, *J* = 11.4, 2.9 Hz, 1H), 2.04 – 1.94 (m, 2H), 1.89 (dt, *J* = 13.2, 6.6 Hz, 3H), 1.86 – 1.77 (m, 1H), 1.73 – 1.58 (m, 3H), 1.50 – 1.17 (m, 26H), 0.91 (t, *J* = 7.3 Hz, 3H).

^{13}C NMR (150 MHz, CDCl$_3$) δ 174.83, 166.72, 136.28, 130.71, 128.38 (2C), 126.88 (2C), 62.12, 48.53, 47.63, 45.67, 38.28, 31.76, 30.58, 27.94, 27.92, 26.96, 26.90, 26.83 (2C), 26.78, 26.60, 26.25, 26.18, 26.11, 24.37, 22.33, 22.24, 20.97, 14.44.

IR (neat) ν: 2928, 2857, 1717, 1668, 1624, 1578, 1535, 1489, 1451, 1365, 1340, 1226, 757, 705 cm^{-1}.

[α]$_D^{20}$ = -0.38 (c = 2.0, CHCl$_3$).

HRMS (ESI$^+$): exact mass calculated for [M+Na]$^+$ ($C_{31}H_{50}N_2O_2Na$) requires m/z 505.3764, found m/z 505.3765.

Enantiomeric ratio = 93:7 was determined by chiral HPLC analysis: Chiralpak IC, *n*-heptane+0.1%IPA/IPA 80:20, 1 mL/min, 25 °C, detection at 254 nm, retention time (min): 15.0 (major) and 13.6 (minor).

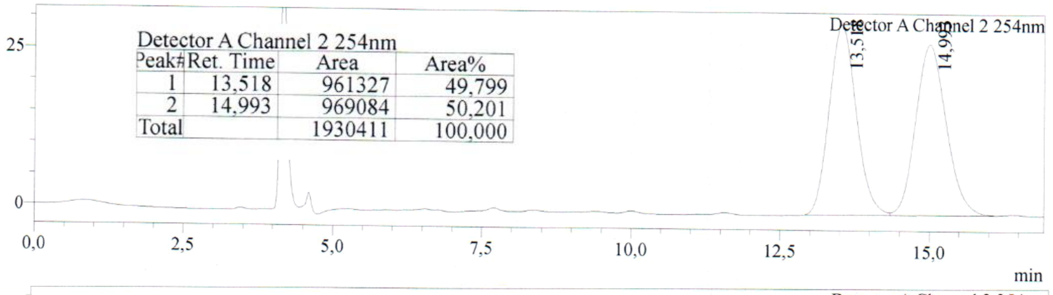

Detector A Channel 2 254nm

Peak#	Ret. Time	Area	Area%
1	13,518	961327	49,799
2	14,993	969084	50,201
Total		1930411	100,000

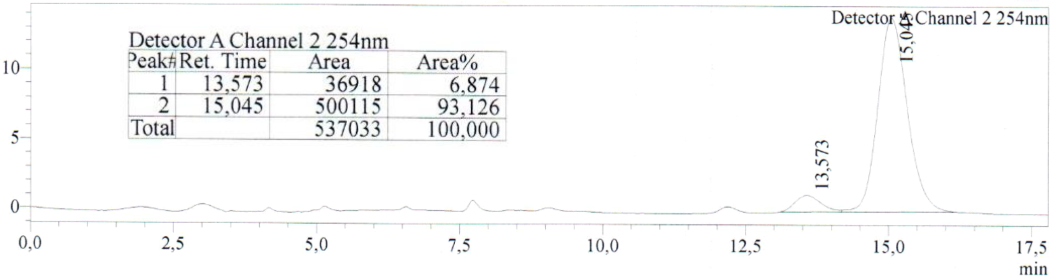

Detector A Channel 2 254nm

Peak#	Ret. Time	Area	Area%
1	13,573	36918	6,874
2	15,045	500115	93,126
Total		537033	100,000

***N*-((*S*)-1-((*S*)-1-Oxo-1-(pyrrolidin-1-yl)pentan-2-yl)-2,3-dihydro-1H-inden-1-yl)benzamide (3ah)**

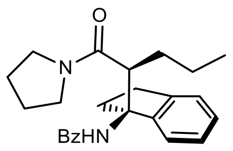

Prepared according to the general procedure in 81% yield (63 mg, 0.162 mmol) as a brown foam (heptane/ethyl acetate 1:1, Rf = 0.38). The *d.r.* was determined by crude NMR to be >20:1.

^{1}H NMR (600 MHz, $CDCl_3$) δ 9.22 (s, 1H), 7.97 – 7.86 (m, 2H), 7.49 – 7.44 (m, 1H), 7.44 – 7.38 (m, 2H), 7.27 (m, 2H), 7.23 (td, *J* = 7.2, 1.3 Hz, 1H), 7.21 – 7.17 (m, 1H), 3.64 – 3.54 (m, 3H), 3.49 (dt, *J* = 9.9, 6.8 Hz, 1H), 3.12 – 3.02 (m, 1H), 2.98 – 2.88 (m, 2H), 2.71 (dd, *J* = 11.7, 3.5 Hz, 1H), 2.08 – 1.90 (m, 6H), 1.63 – 1.56 (m, 1H), 1.27 (tdd, *J* = 12.0, 7.3, 4.6 Hz, 1H), 1.10 – 0.99 (m, 1H), 0.86 (t, *J* = 7.3 Hz, 3H).

^{13}C NMR (150 MHz, $CDCl_3$) δ 174.2, 165.7, 144.0, 141.1, 135.3, 131.1, 128.4 (2C), 127.4, 127.1 (2C), 125.7, 125.3, 125.2, 68.3, 48.2, 47.4, 45.9, 36.1, 30.4, 29.8, 26.2, 24.4, 21.0, 14.3.

IR (neat) ν: 3307, 2960, 2932, 2873, 1738, 1669, 1620, 1603, 1579, 1521, 1482, 1452, 1377, 1363, 1310, 1252, 1187, 755, 697, 571 cm^{-1}.

$[\alpha]_D^{20}$ = -0.76 (c = 2.0, $CHCl_3$).

HRMS (ESI$^+$): exact mass calculated for $[M+Na]^+$ ($C_{25}H_{30}N_2O_2Na$) requires m/z 413.2199, found m/z 413.2196.

Enantiomeric ratio = 93:7 was determined by chiral HPLC analysis: Chiralpak IC, *n*-heptane+0.1%IPA/EtOH 85:15, 1 mL/min, 25 °C, detection at 230 nm, retention time (min): 43.3 (major) and 37.1 (minor).

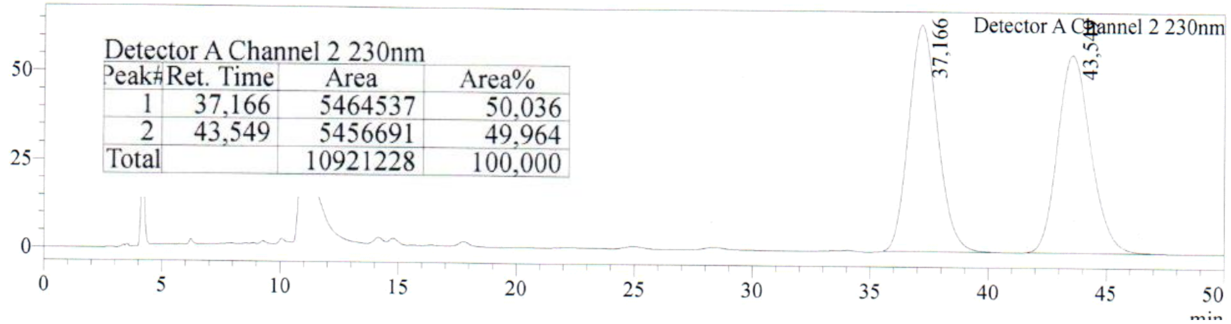

Detector A Channel 2 230nm

Peak#	Ret. Time	Area	Area%
1	37,166	5464537	50,036
2	43,549	5456691	49,964
Total		10921228	100,000

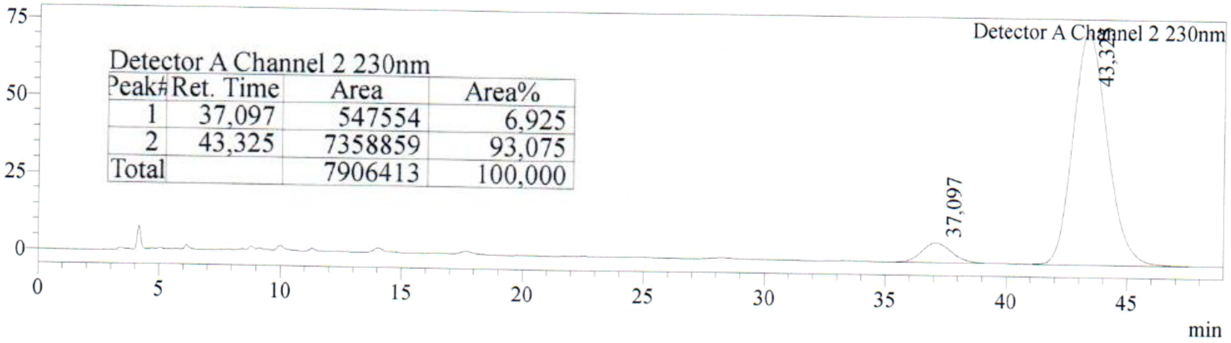

Detector A Channel 2 230nm

Peak#	Ret. Time	Area	Area%
1	37,097	547554	6,925
2	43,325	7358859	93,075
Total		7906413	100,000

***N*-((*S*)-4-((*S*)-1-Oxo-1-(pyrrolidin-1-yl)pentan-2-yl)chroman-4-yl)benzamide (3ai)**

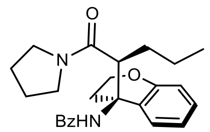

Prepared according to the general procedure in 55% yield (45 mg, 0.11 mmol) as a white foam (heptane/ethyl acetate 1:1, Rf = 0.28). The *d.r.* was determined by crude NMR to be >20:1.

^{1}H NMR (600 MHz, CDCl$_3$) δ 9.43 (s, 1H), 7.92 (d, *J* = 7.0 Hz, 2H), 7.50 – 7.45 (m, 1H), 7.45 – 7.41 (m, 2H), 7.34 (dd, *J* = 7.8, 1.5 Hz, 1H), 7.15 (ddd, *J* = 8.5, 7.2, 1.6 Hz, 1H), 6.89 – 6.82 (m, 2H), 4.39 (ddd, *J* = 11.3, 5.5, 3.0 Hz, 1H), 4.33 (td, *J* = 11.9, 3.3 Hz, 1H), 3.64 – 3.55 (m, 3H), 3.51 (dt, *J* = 10.1, 6.8 Hz, 1H), 3.22 (ddd, *J* = 14.0, 12.4, 5.6 Hz, 1H), 3.08 (dd, *J* = 11.6, 3.7 Hz, 1H), 2.06 – 1.87 (m, 5H), 1.78 (dt, *J* = 14.0, 3.1 Hz, 1H), 1.63 (ddd, *J* = 17.0, 8.4, 4.4 Hz, 1H), 1.33 – 1.28 (m, 1H), 1.15 – 1.06 (m, 1H), 0.87 (t, *J* = 6.3 Hz, 3H).

^{13}C NMR (150 MHz, CDCl$_3$) δ 174.0, 165.7, 153.7, 135.1, 131.2, 128.9 (2C), 128.4, 127.1 (2C), 123.4, 119.6, 117.2, 63.3, 55.7, 49.6, 47.6, 46.0, 31.9, 30.8, 30.6, 26.2, 24.2, 20.9, 14.4.

IR (neat) ν: 3293, 2962, 2930, 2873, 1669, 1618, 1580, 1520, 1487, 1452, 1341, 1307, 1285, 1254, 1187, 756, 573 cm^{-1}.

$[\alpha]_D^{20}$ = -0.49 (c = 1.0, CHCl$_3$).

HRMS (ESI$^+$): exact mass calculated for $[M+Na]^+$ ($C_{25}H_{31}N_2O_3$) requires m/z 407.2329, found m/z 407.2327.

Enantiomeric ratio = 98:2 was determined by chiral HPLC analysis: Chiralpak IC, *n*-heptane+0.1%IPA/EtOH 80:20, 1 mL/min, 25 °C, detection at 230 nm, retention time (min): 12.2 (major) and 18.7 (minor).

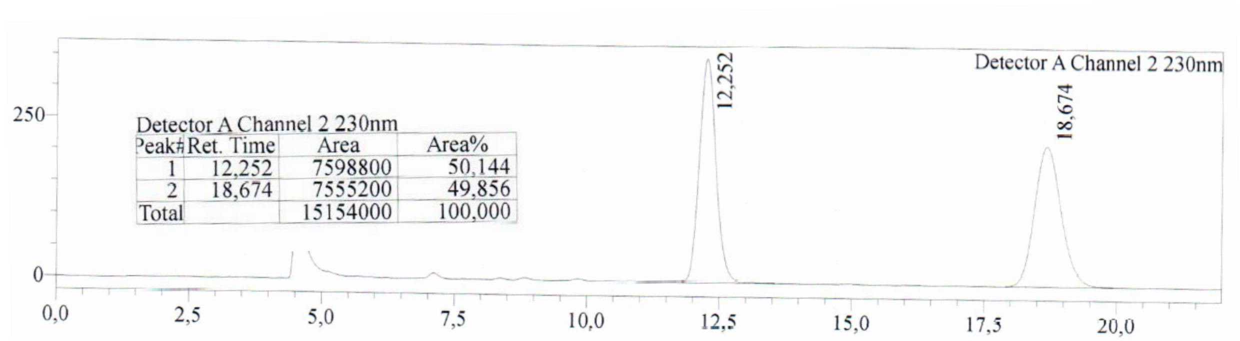

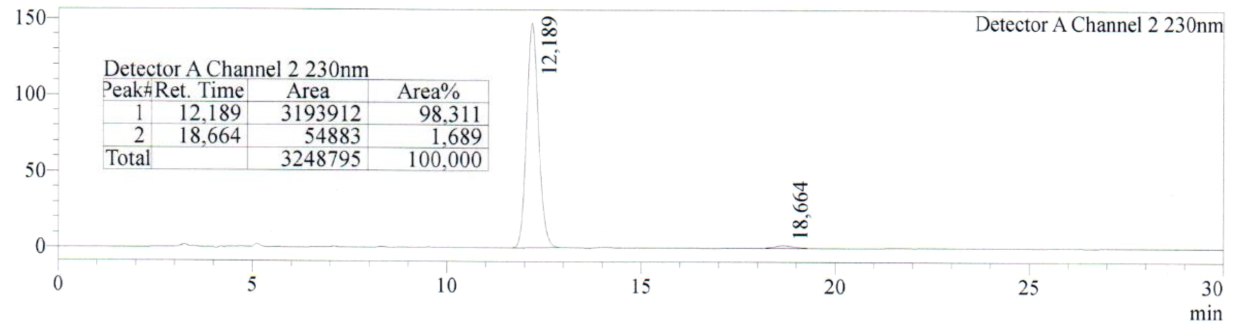

***N*-((*S*)-1-((*S*)-1-Oxo-1-(pyrrolidin-1-yl)pentan-2-yl)-1,2,3,4-tetrahydronaphthalen-1-yl)benzamide (3aj)**

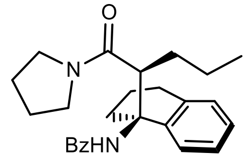

Prepared according to the general procedure in 47% yield (38 mg, 0.094 mmol) as a brown foam (heptane/ethyl acetate 1:1, Rf = 0.38). The d.r. was determined by crude NMR as 10:1.

^{1}H NMR (600 MHz, $CDCl_3$) δ 9.65 (s, 1H), 7.94 (d, *J* = 6.9 Hz, 2H), 7.48 – 7.40 (m, 3H), 7.38 (d, *J* = 7.7 Hz, 1H), 7.14 (app qd, *J* = 8.0, 3.4 Hz, 2H), 7.11 – 7.07 (m, 1H), 3.64 – 3.53 (m, 4H), 3.01 (ddd, *J* = 15.5, 14.5, 6.7 Hz, 2H), 2.88 – 2.79 (m, 2H), 2.06 – 1.89 (m, 7H), 1.77 (dt, *J* = 13.6, 3.7 Hz, 1H), 1.55 – 1.48 (m, 1H), 1.25 – 1.18 (m, 1H), 1.03 (tdd, *J* = 12.1, 7.2, 4.8 Hz, 1H), 0.85 (t, *J* = 7.2 Hz, 3H).

^{13}C NMR (150 MHz, $CDCl_3$) δ 174.7, 165.6, 137.1, 135.9, 135.4, 131.0, 129.2, 128.6 (2C), 128.4, 127.1 (2C), 126.6, 124.7, 59.1, 49.3, 47.4, 45.9, 32.0, 31.3, 29.1, 26.2, 24.3, 20.9, 20.5, 14.4.

IR (neat) ν: 3291, 2957, 2928, 2873, 1667, 1616, 1518, 1485, 1451, 1378, 1339, 1272, 1073, 1043, 753, 707, 598, 572 cm^{-1}.

$[\alpha]_D^{20}$ = -0.41 (c = 1.0, $CHCl_3$).

HRMS (ESI$^+$): exact mass calculated for $[M+Na]^+$ ($C_{26}H_{32}N_2O_2Na$) requires m/z 427.2356, found m/z 427.2356.

Enantiomeric ratio = 99:1 was determined by chiral HPLC analysis: Chiralpak IC, *n*-heptane+0.1%IPA/IPA 85:15, 1 mL/min, 25 °C, detection at 230 nm, retention time (min): 13.4 (major) and 19.6 (minor).

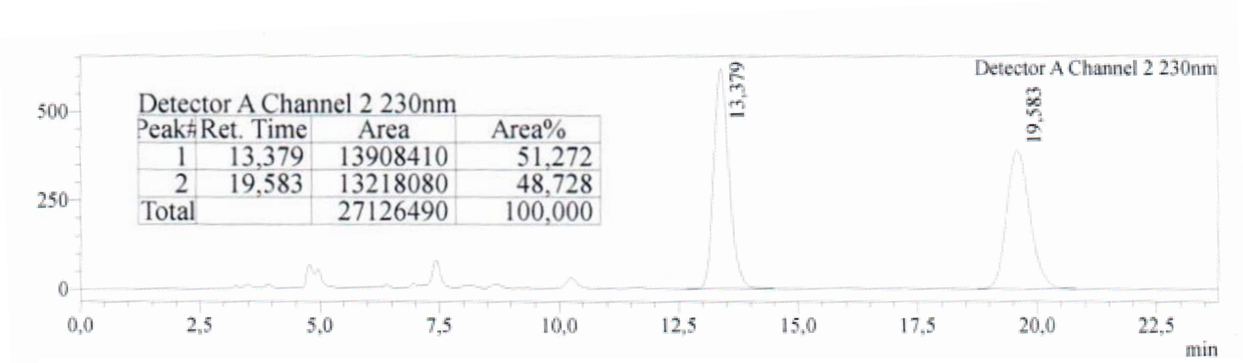

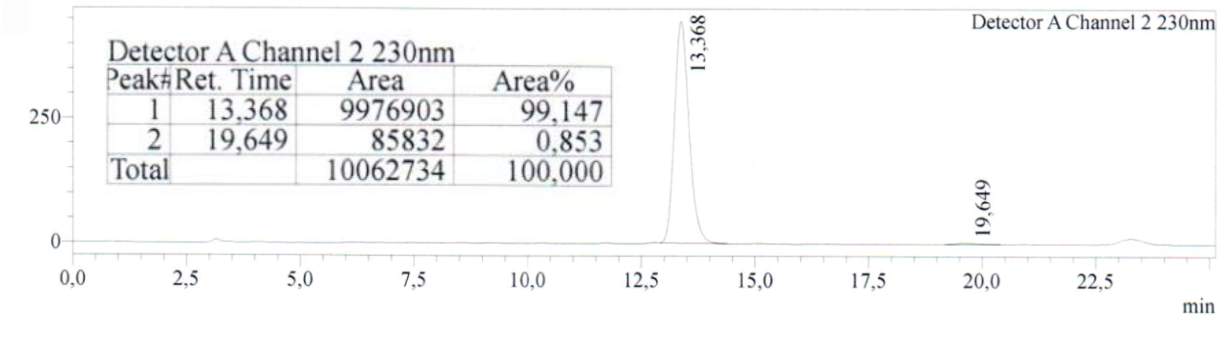

5. Comparison to conventional Mannich reaction and synthetic applications

5.1 Comparison to conventional Mannich reaction

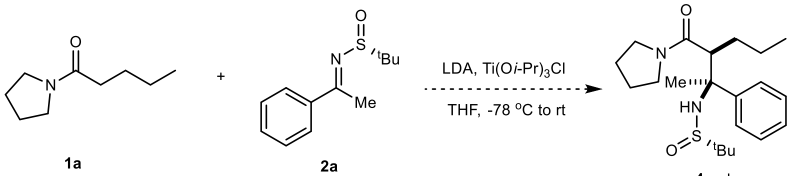

1a **2a** **4**, nd

The procedure employed for a traditional Mannich process was developed by Ellman *et al.*[24]

A solution of *i*-Pr_2NH (62 µL, 0.44 mmol, 2.20 equiv.) in THF (0.20 M) was cooled to 0 °C. *n*-Butyllithium (0.21 mL, 0.42 mmol, 2.00 M, 2.10 equiv.) was added *via* syringe and the solution was stirred for 30 min. The solution was then cooled to -78 °C and a solution of the amide **1a** (60 mg, 0.40 mmol, 2.00 equiv) in THF (1 mL) was added *via* syringe and the reaction solution was stirred for 30 min. To this solution was added Ti(O*i*-Pr)$_3$Cl in THF (1.00 M, 0.84 mL, 0.84 mmol, 4.20 equiv.) and the mixture was stirred for 30 min. A solution of the sulfinimine **2a** (44 mg, 0.20 mmol, 1.00 equiv.) in THF (5.00 M, 40 µL) was slowly added *via* syringe and the solution was stirred for 10 h at -78 °C. A saturated aqueous solution of NH_4Cl (5 mL) was added and the suspension was warmed to room temperature. The desired product was not detected in the crude mixture, as judged by LC/MS analysis. Instead, large proportions of both starting material remains were observed.

5.2 Preparation of β-amino acid 5 from indoline amide 3o

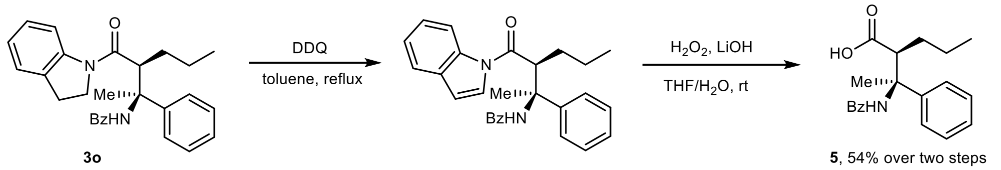

3o **5**, 54% over two steps 88.5:11.5 *e.r.*

The two-step procedure was partially adapted from a previous report.[25]

Amide **3o** (25 mg, 0.058 mmol, 1.00 equiv.) and 2,3-dichloro-5,6-dicyano-1,4-benzoquinone (DDQ, 27 mg, 0.12 mmol, 2.00 equiv.) were dissolved with anhydrous toluene (1.00 mL) in an oven-dried vial. The capped vial was then placed in an oil bath at 110 °C and the reaction mixture was stirred until TLC analysis indicated full conversion (5 h). After allowing to cool to room temperature, the mixture was filtered through a column loaded with silica gel (eluting with 33% ethyl acetate in heptane), thereby removing excess DDQ. Upon concentration of the resulting filtrate, the residue was dissolved in THF (1.1 mL) and water (0.3 mL). The mixture was cooled to 0 °C and LiOH (5 mg, 0.19 mmol, 3.25 equiv.) and H_2O_2 (30%, 0.066 mL, 0.58 mmol, 10.0 equiv.) was added. The reaction mixture was allowed to warm to room temperature overnight (14 h). At this point, a saturated aqueous solution of sodium thiosulfate ($Na_2S_2O_3$, 1 mL) was added and the mixture was diluted with water (15 mL). The resulting solution was acidified to pH 1 using 1 N HCl and was then extracted with CH_2Cl_2 (5 x 10 mL). The combined organic layers were

dried over anhydrous magnesium sulfate, the dried solution was filtered and the filtrate was concentrated under reduced pressure to afford the crude product. Flash column chromatography on silica gel (2-5% MeOH in CH_2Cl_2) afforded 19.0 mg (54%) of the desired β-amino acid (CH_2Cl_2/MeOH 20:1, Rf = 0.48).

^{1}H NMR (600 MHz, $CDCl_3$) δ 7.87 (d, *J* = 7.4 Hz, 2H), 7.72 (s, 1H), 7.52 (t, *J* = 7.4 Hz, 1H), 7.45 (t, *J* = 7.6 Hz, 2H), 7.35 – 7.28 (m, 4H), 7.23 (t, *J* = 6.7 Hz, 1H), 2.68 (dd, *J* = 11.9, 2.8 Hz, 1H), 2.00 (s, 3H), 1.68 – 1.59 (m, 1H), 1.16 – 1.03 (m, 2H), 0.90 – 0.82 (m, 1H), 0.76 (t, *J* = 7.2 Hz, 3H).

^{13}C NMR (150 MHz, $CDCl_3$) δ 178.7, 166.9, 141.7, 135.0, 131.6, 128.7(2C), 128.2(2C), 127.0 (2C), 126.9, 125.8 (2C), 60.2, 56.9, 29.5, 25.2, 20.8, 13.6.

IR (neat) ν: 3024, 2959, 2928, 1715, 1675, 1637, 1603, 1578, 1533, 1490, 1447, 1377, 1314, 1226, 1194, 759, 713, 700 cm^{-1}.

$[\alpha]_D^{20}$ = -0.046 (c = 0.5, $CHCl_3$).

HRMS (ESI$^+$): exact mass calculated for $[M+H]^+$ ($C_{20}H_{24}N_2O_3$) requires m/z 326.1751, found m/z 326.1750.

Enantiomeric ratio = 88.5:11.5 was determined by chiral HPLC analysis: Chiralpak IC, *n*-heptane/IPA/TFA 85:15:0.1, 1 mL/min, 25 °C, detection at 210 nm, retention time (min): 11.3 (major) and 10.3 (minor).

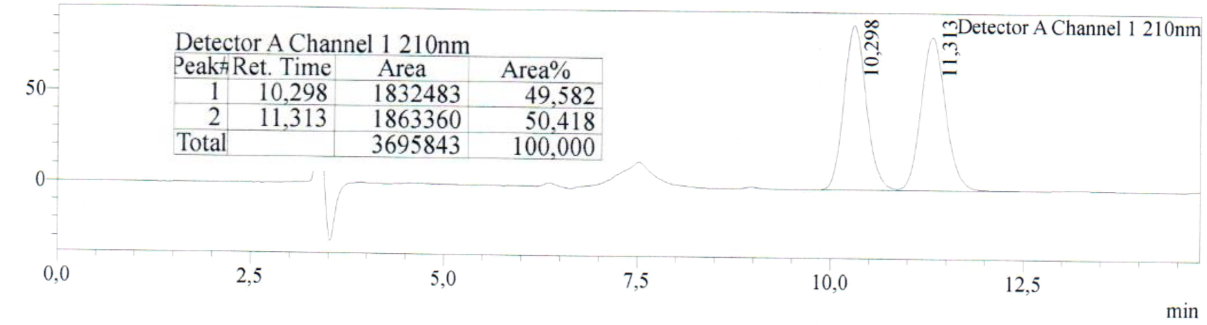

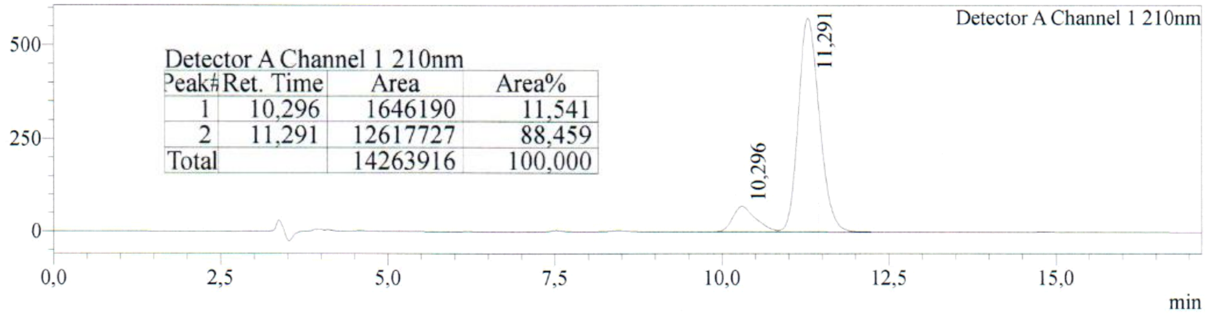

5.3 Synthesis of the piperidine derivative 6

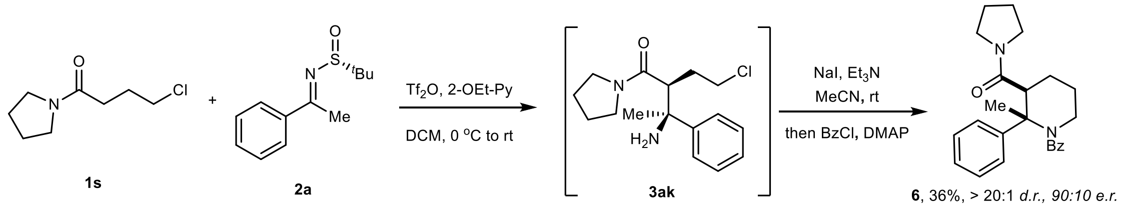

To a solution of amide **1s** (38 mg, 0.20 mmol, 1.00 equiv.), 2-ethoxypyridine (54 µL, 0.44 mmol, 2.20 equiv.) in CH_2Cl_2 (1 mL) in flame-dried Schlenk tube was added triflic anhydride (40 µL, 0.24 mmol) dropwise under argon at 0 °C. After stirring for 30 min at 0 °C, sulfinimine (89 mg, 0.40 mmol, 2.00 equiv.) in CH_2Cl_2 (1 mL) was added and the reaction stirred for a further 5 min at 0 °C. After stirring at room temperature for 24 h, the β-amino amide **3ak** was observed by LC/MS analysis.

The CH_2Cl_2 was then removed by rotary evaporation, after which triethylamine (84 µL, 0.60 mmol, 3.00 equiv.), sodium iodide (6 mg, 0.04 mmol, 0.20 equiv.) and MeCN (1 mL) were added to the residue. The reaction was then stirred at room temperature for 24 h, after which full consumption of the β-amino amide was observed (monitored by LC/MS analysis). At this point, triethylamine (84 µL, 0.60 mmol, 3.00 equiv.), 4-dimethylaminopyridine (DMAP, 5.0 mg, 0.04 mmol, 0.20 equiv.) and benzoyl chloride (70 µL, 0.60 mmol, 3.00 equiv.) were added. The resulting mixture was stirred at room temperature for an additional 14 h before the addition of a saturated aqueous solution of sodium bicarbonate. The biphasic mixture was extracted with CH_2Cl_2 (twice) and the combined organic layers were dried over anhydrous magnesium sulfate. The dried solution was filtered and the filtrate was concentrated under reduced pressure to afford the crude product. The *d.r.* was identified to be >20:1 by crude ^{1}H NMR. The product (major diastereomer) was obtained after purification by flash column chromatography on silica gel to give the desired product in 36% overall yield (27 mg, 0.072 mmol, heptane/ethyl acetate 1:2, Rf = 0.30).

^{1}H NMR (600 MHz, $CDCl_3$) δ 7.49 (dd, *J* = 7.5, 1.9 Hz, 2H), 7.35 (dd, *J* = 16.2, 7.2 Hz, 5H), 7.17 (d, *J* = 8.0 Hz, 2H), 7.06 (t, *J* = 7.3 Hz, 1H), 3.77 (dd, *J* = 8.8, 4.9 Hz, 1H), 3.33 (ddd, *J* = 13.5, 9.8, 3.5 Hz, 1H), 3.27 – 3.16 (m, 1H), 3.14 – 3.02 (m, 1H), 2.91 (ddd, *J* = 22.8, 14.2, 5.1 Hz, 2H), 2.09 – 1.95 (m, 5H), 1.83 (dd, *J* = 9.2, 3.7 Hz, 1H), 1.71 (dd, *J* = 11.0, 6.4 Hz, 2H), 1.57 (d, *J* = 6.9 Hz, 2H), 1.46 – 1.38 (m, 1H), 1.32 – 1.25 (m, 1H).

^{13}C NMR (150 MHz, $CDCl_3$) δ 173.1, 170.4, 146.6, 137.8, 130.1, 128.5(2C), 128.1(2C), 127.8(2C), 126.1, 124.5(2C), 63.1, 52.1, 46.0, 45.7, 45.6, 29.7, 26.0, 24.1, 24.0, 16.0.

IR (neat) ν: 2970, 2870, 1716, 1632, 1444, 1396, 1371, 1225, 763, 700 cm^{-1}.

$[\alpha]_D^{20}$ = 0.48 (c = 0.5, $CHCl_3$).

HRMS (ESI^+): exact mass calculated for $[M+Na]^+$ ($C_{24}H_{28}N_2O_2Na$) requires m/z 399.2043, found m/z 399.2045.

Enantiomeric ratio = 90:10 was determined by chiral HPLC analysis: Lux-Cellulose 1, *n*-heptane+0.1%IPA/IPA 90:10, 1 mL/min, 25 °C, detection at 210 nm, retention time (min): 20.0 (major) and 44.7 (minor).

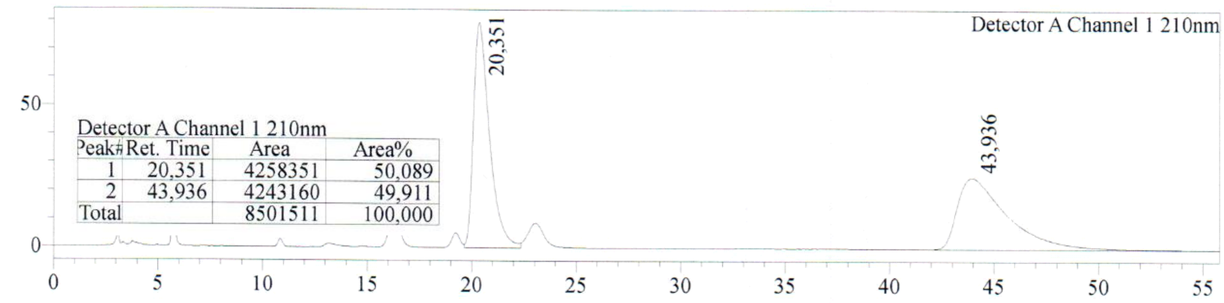

Detector A Channel 1 210nm

Peak#	Ret. Time	Area	Area%
1	20,351	4258351	50,089
2	43,936	4243160	49,911
Total		8501511	100,000

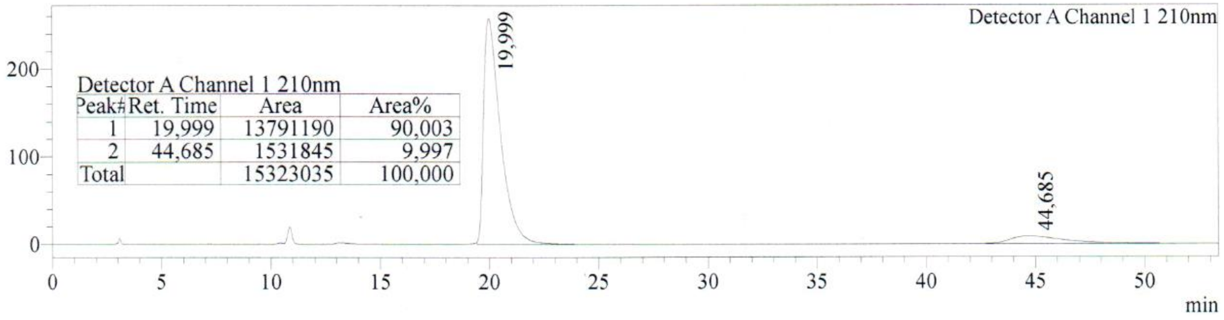

Detector A Channel 1 210nm

Peak#	Ret. Time	Area	Area%
1	19,999	13791190	90,003
2	44,685	1531845	9,997
Total		15323035	100,000

6. Limitations

The substrates giving only trace amount of the product were listed in the table below.

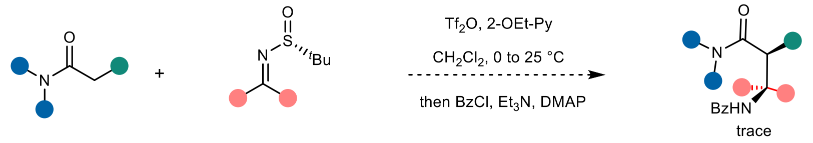

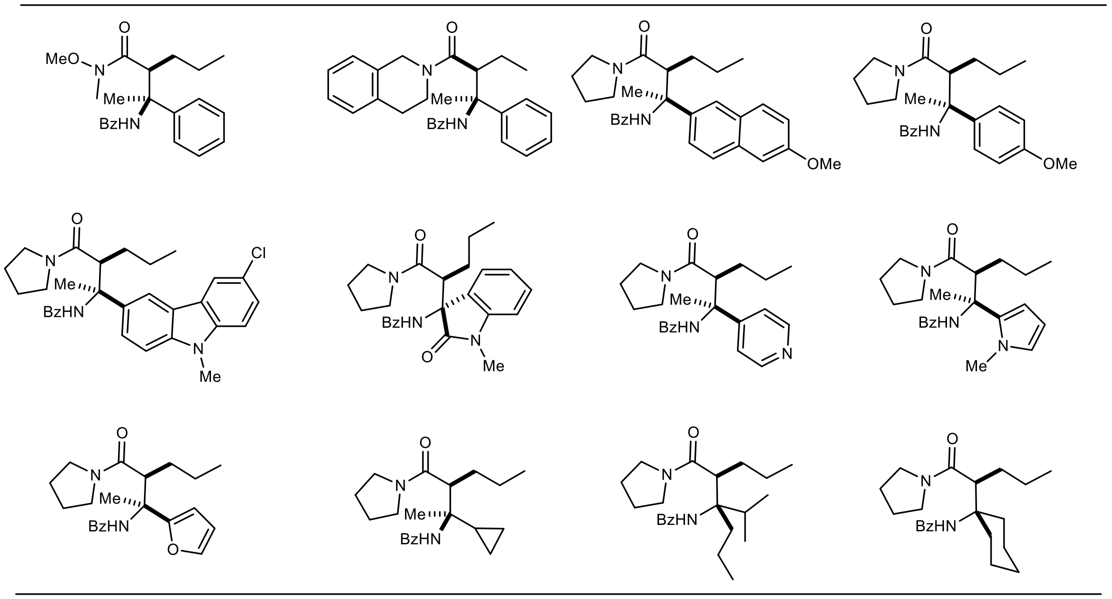

In the reaction with sulfinimine derived from 4-phenylbutan-2-one (E/Z = 4.6/1), the product was isolated in 68% yields with two diastereomers (2.4:1).

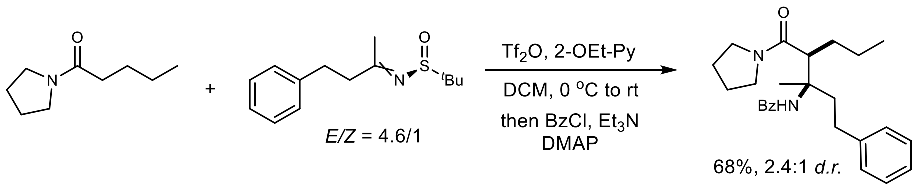

7. *In situ* NMR studies of the reaction

For detection of possible intermediates present in the reaction mixture, *in situ* NMR studies of a standard reaction were performed. The experiment was performed in an oven-dried NMR tube with a screw cup equipped with a septum, using a 700 MHz NMR spectrometer (Bruker Avance III HD) without internal stirring. The reaction was performed according to general procedure D using racemic (*E*)-2-methyl-*N*-(1-phenylethylidene)propane-2-sulfinamide (89 mg, 0.40 mmol, 2.00 equiv.), 1-(pyrrolidine-1-yl)-pentan-1-one (31 mg, 0.20 mmol, 1.00 equiv.), 2-ethyoxypyridine (54 mg, 0.44 mmol, 2.20 equiv.), triflic anhydride (40 µL, 0.24 mmol, 1.20 equiv.), triethylamine (139 µL, 1.00 mmol, 5.00 equiv.), 4-dimethylaminopyridine (5 mg, 0.04 mmol, 0.20 equiv.) and benzoyl chloride (116 µL, 1.00 mmol, 5.00 equiv.) in CD_2Cl_2 (2.0 mL) with CH_2Br_2 (14 µL, 0.20 mmol, 1.00 equiv.) as internal standard.

The detected species are presented in the scheme below:

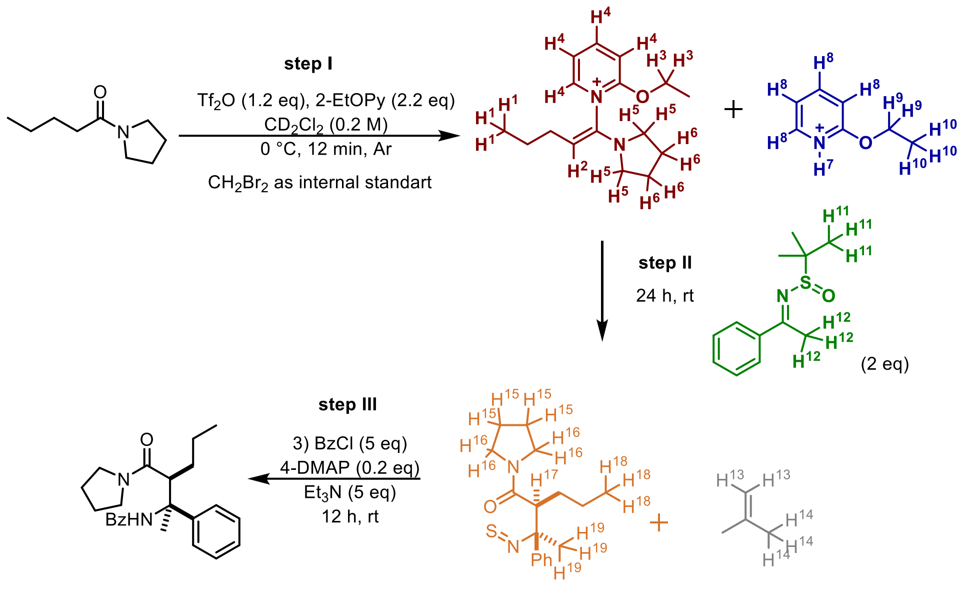

After the amide activation step (step I), clean conversion of starting amide to the pyridinium intermediate (maroon) was observed, as shown by the spectra below, recorded 12 min after addition of Tf_2O (signals were assigned based on integrals values with an assistant of COSY).

After the addition of sulfinimine (step II), isobutylene was found in the reaction mixture. In addition, the amount of protonated pyridine was twice as high as after the amide activation (step I), providing additional proof for the deprotonation of the rearrangement product and elimination of isobutylene.

Isobutylene: **^{1}H NMR** (700 MHz, CD_2Cl_2): δ = 4.65 (hept, *J* = 1.3 Hz, 2H), 1.72 (t, *J* = 1.3 Hz, 2H); **^{13}C NMR** (176 MHz, CD_2Cl_2): δ =110.3 (CH_2), 24.5 (CH_3), signal of C is not visible (according to cross-peaks in HSQC). Data is in correspondence with published spectra [Anthony Fernandes. Etude et réactivité des cations silyliums. Chimie organique. Université de Bordeaux, 2018. Français. page 268. NNT: 2018BORD0255. tel-03092253].

During the benzylation step (step III), it was not possible to determine any intermediates. Direct conversion of the intermediate from the previous step to the product was observed.

I step: ^{1}H NMR of the reaction mixture 12 min after Tf_2O addition.

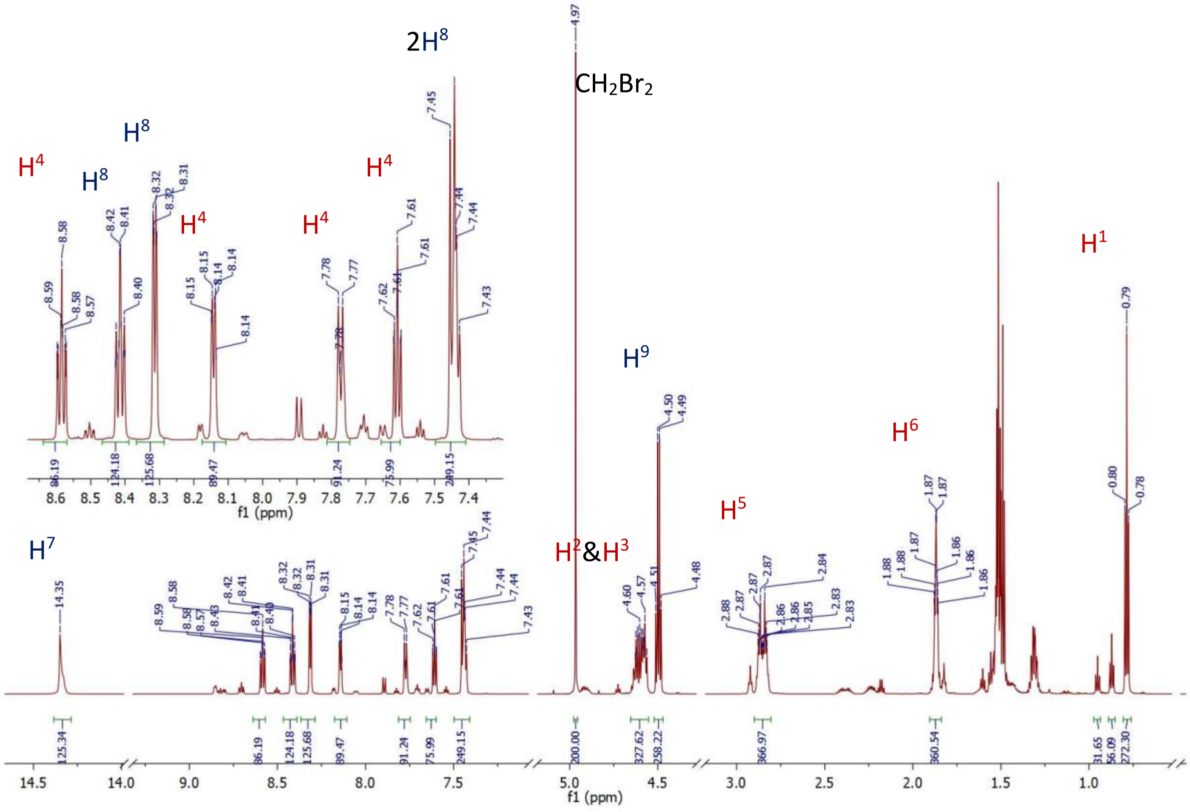

2H^8
CH$_2$Br$_2$
H^4
H^8
H^8
H^4
H^4
H^4
H^9
H^1
H^6
H^5
H^7
H^2&H^3
f1 (ppm)

II step: 2 copies highlighting different signals in ^1H NMR spectra of the reaction mixture 10 h after sulfinamide addition.

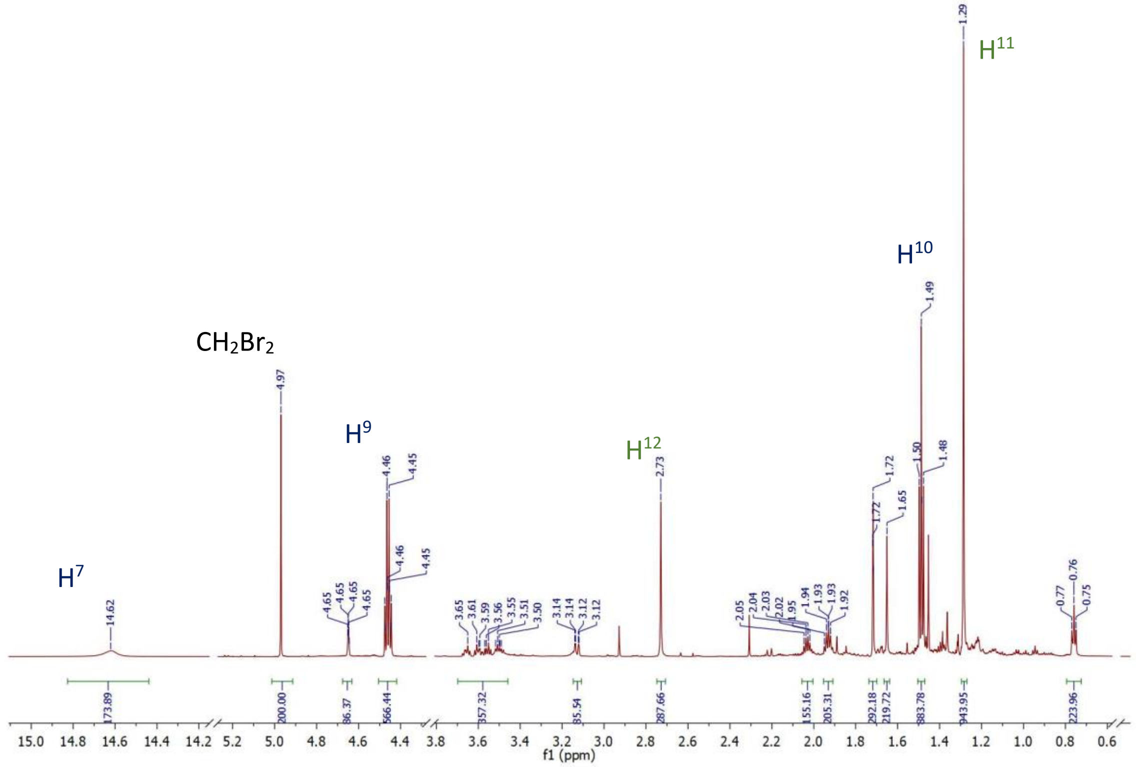

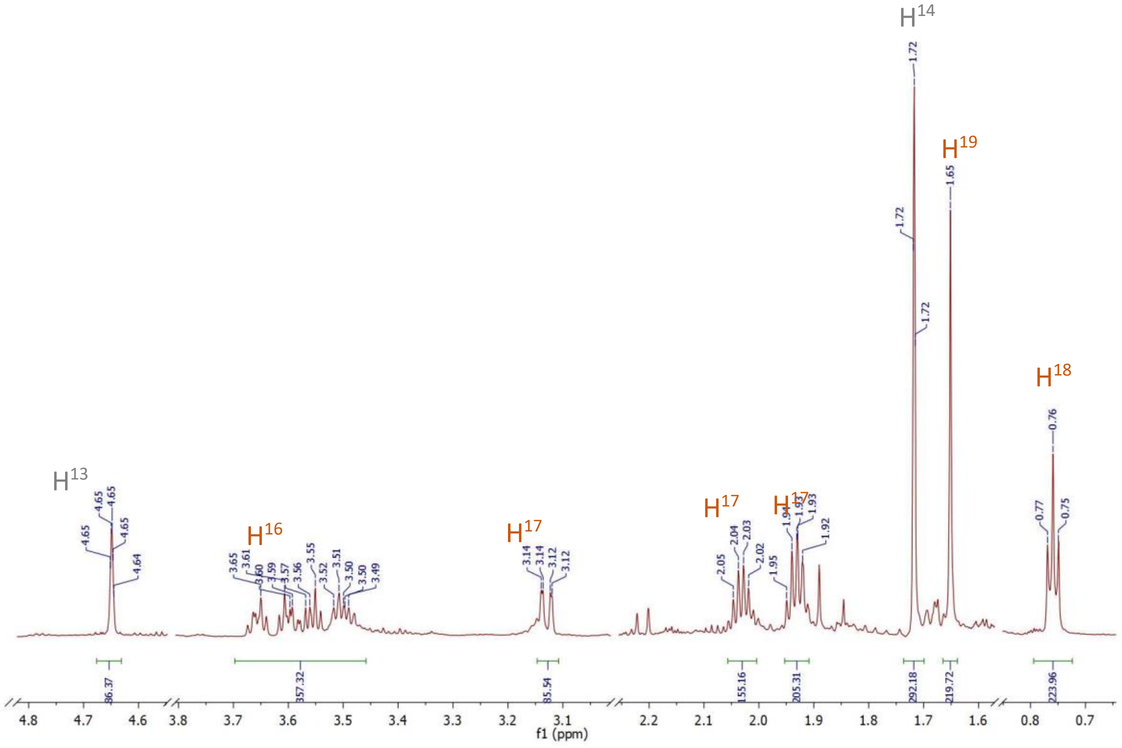

II step: ^{1}H and HSQC(^{1}H-^{13}C) of the reaction mixture 10 h after sulfinamide addition highlighting isobutylene signals.

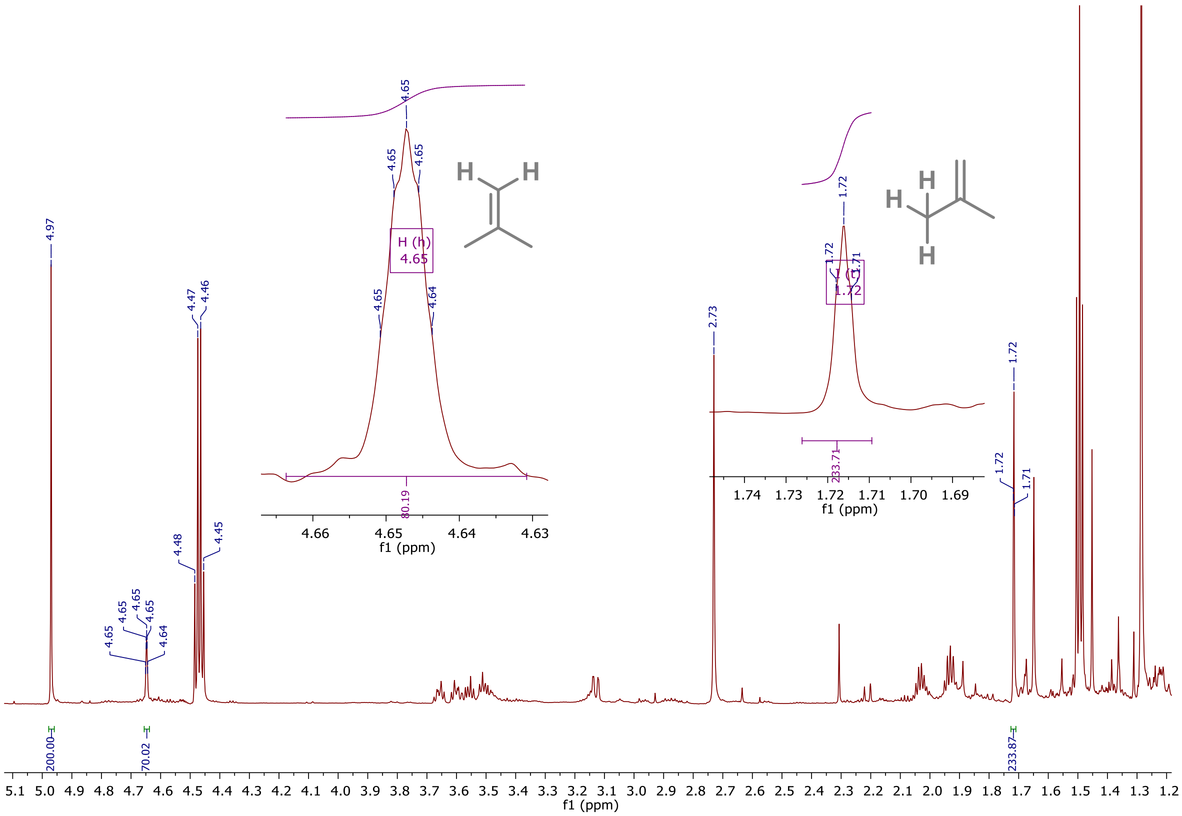

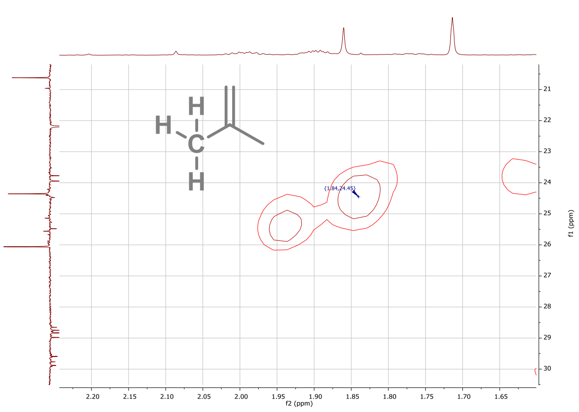

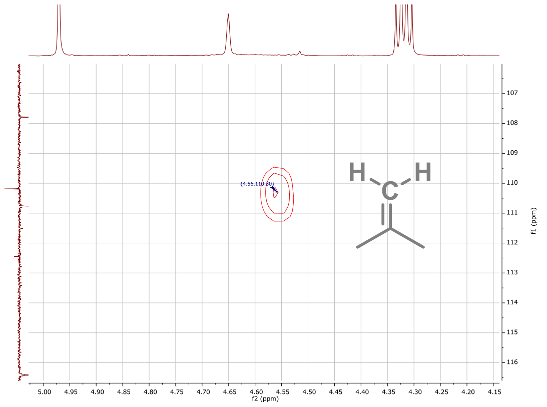

Changes in integrals of representative ^{1}H NMR signals after sulfinimine addition.

Sulfinimine (H^{12} intensity/3)

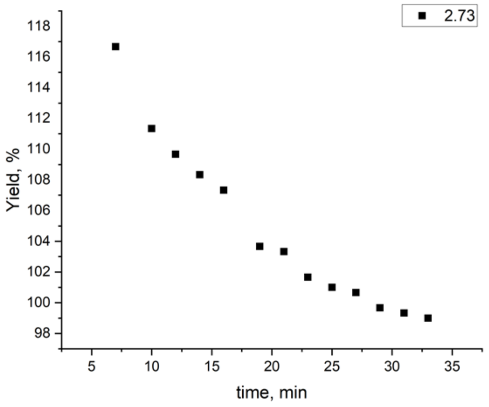

Product (H^{19} intensity/3)

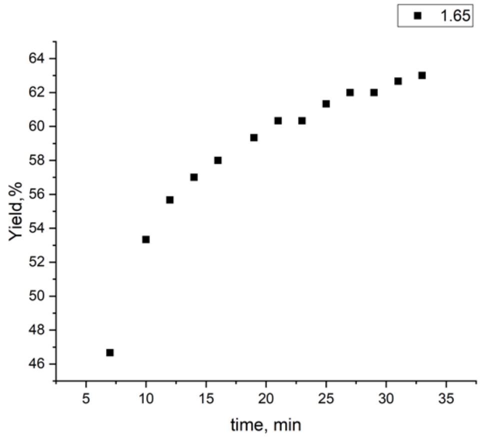

8. ^{18}O-labeling experiment

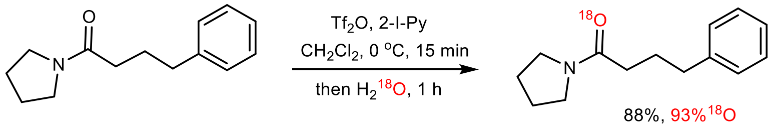

Preparation of ^{18}O-labeled amide: the ^{18}O-labeled amide was prepared according to the reported procedure.[5] The isotopic enrichment was determined by HRMS analysis to be 93%.

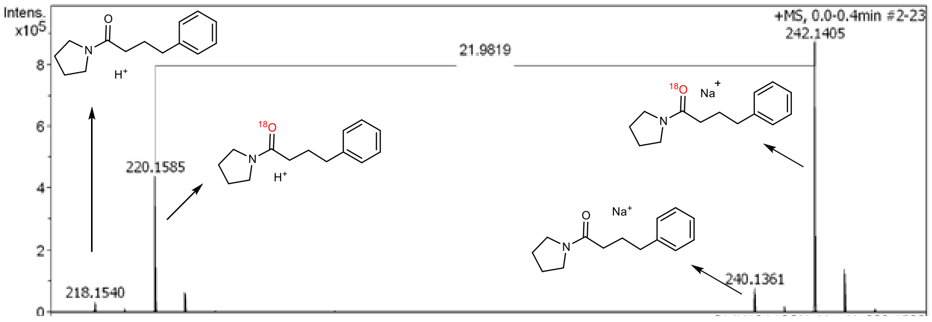

#	m/z	I
1	218.1540	34357
2	219.1576	10350
3	220.1585	439240
4	221.1617	63526
5	222.1648	4371
6	226.1662	3018
7	240.1361	78389
8	241.1397	21787
9	242.1405	870020
10	243.1437	139761
11	244.1467	9658
12	301.0752	2805
13	359.1018	11158
14	360.1050	2784
15	443.2575	5194
16	445.3170	4459
17	457.2814	2553
18	458.2832	4759
19	458.7844	2490
20	459.2869	29998
21	460.2903	12773
22	461.2914	176251
23	462.2945	55437
24	463.2966	7111
25	485.2503	4250
26	487.3087	25701
27	488.3122	9097
28	520.2257	4020
29	688.4789	3806
30	904.5854	3015

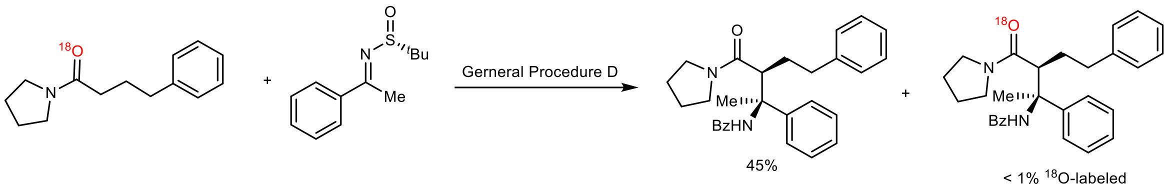

The prepared ^{18}O-labeled amide was used as the starting material in the reaction with sulfinimine **2a** under standard conditions. The product was obtained in 45% isolated yield. HRMS analysis of the product determined the retention of the isotopic label to be less than 1%.

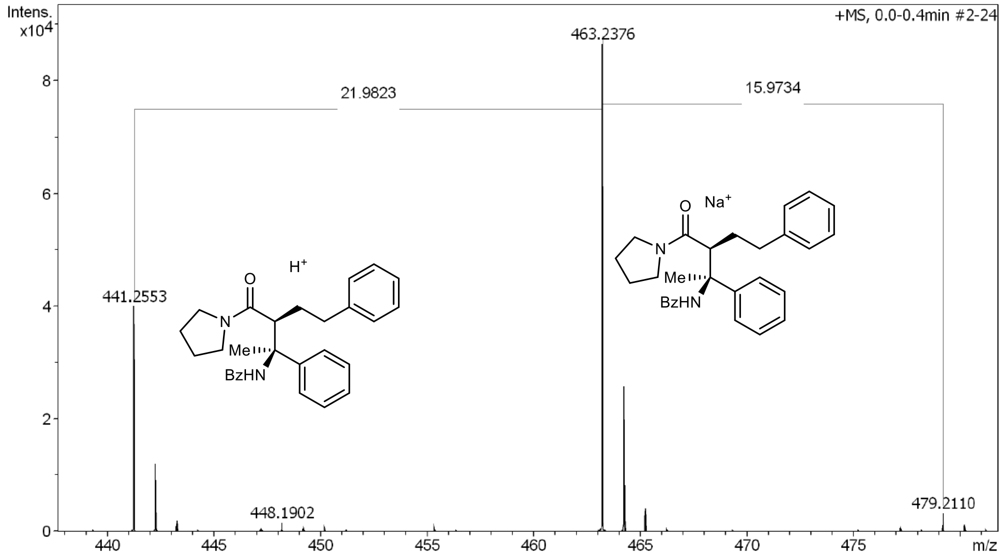

#	m/z	I
1	158.1542	3562
2	230.2115	1652
3	304.2616	4794
4	320.2016	1808
5	321.1992	1214
6	335.2126	7918
7	336.2159	1930
8	340.2617	2590
9	370.1810	2752
10	380.2020	1184
11	425.2239	1340
12	441.2553	36793
13	442.2584	11054
14	443.2614	1802
15	448.1902	1464
16	463.2376	79564
17	464.2405	23719
18	465.2434	3786
19	479.2110	2988
20	497.2215	1505
21	527.3933	1434
22	571.4196	1685
23	598.4020	2120
24	615.4460	1581
25	903.4852	68352
26	904.4881	41669
27	905.4913	12124
28	906.4939	2407
29	937.4689	1924
30	938.4724	1359

9. Reaction with aldimine

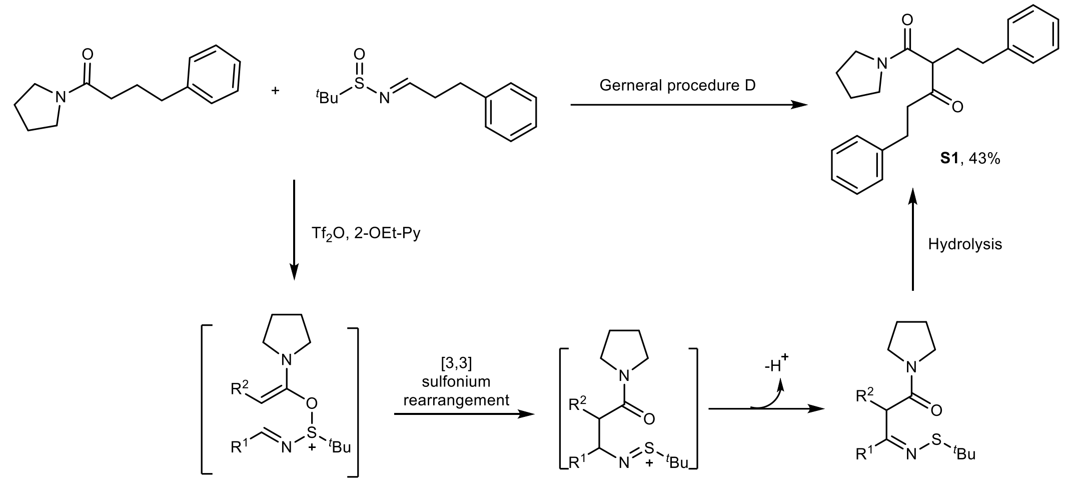

The title 1,3-dicarbonyl compound was isolated in 43% yield when the title amide and aldimine were used as the starting materials using general procedure D. The proposed mechanism is illustrated in the scheme above.

^{1}H NMR (700 MHz, $CDCl_3$) δ 7.36 – 7.25 (m, 4H), 7.25 – 7.10 (m, 6H), 3.51 – 3.44 (m, 1H), 3.40 (ddd, *J* = 14.4, 7.7, 4.7 Hz, 2H), 3.19 – 3.11 (m, 2H), 2.95 – 2.77 (m, 4H), 2.70 – 2.62 (m, 1H), 2.60 – 2.52 (m, 1H), 2.31 (dtd, *J* = 14.5, 8.4, 6.3 Hz, 1H), 2.13 (ddt, *J* = 13.5, 8.6, 6.6 Hz, 1H), 1.91 – 1.78 (m, 4H).

^{13}C NMR (175 MHz, $CDCl_3$) δ 205.8, 166.9, 140.9, 149.9, 128.5 (2C), 128.5 (2C), 128.4 (2C), 128.3 (2C), 126.1, 126.0, 58.9, 46.6, 46.1, 41.0, 33.4, 30.1, 29.4, 25.9, 24.1.

IR (neat) ν: 3024, 2959, 2928, 1715, 1675, 1637, 1603, 1578, 1533, 1490, 1447, 1377, 1314, 1226, 1194, 759, 713, 700 cm^{-1}.

HRMS (ESI$^+$): exact mass calculated for $[M+H]^+$ ($C_{23}H_{28}NO_2$) requires m/z 350.2115, found m/z 350.2113.

10. Gram-scale reaction

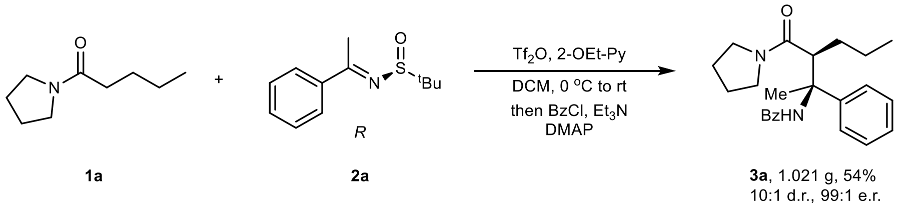

To a cooled (0 °C) mixture of amide **1a** (776 mg, 5.0 mmol, 1.00 equiv.), 2-OEt-pyridine (1343 µL, 11.0 mmol, 2.20 equiv.) in CH_2Cl_2 (10 mL) under argon in flame-dried Schlenk tube triflic anhydride (1009 µL, 6.0 mmol, 1.20 equiv.) was added dropwise. After stirring for 30 min at 0 °C, sulfinimine **2a** (2.233 g, 10.0 mmol, 2.00 equiv.) in CH_2Cl_2 (10 mL) was added and the reaction mixture was stirred for a further 5 min at 0 °C. After 24 h of stirring at room temperature, triethylamine (1.742 mL, 12.5 mmol, 2.50 equiv.), 4-dimethylaminopyridine (DMAP, 125 mg, 1.0 mmol, 0.20 equiv.) and benzoyl chloride (1.451 mL, 12.5 mmol, 2.50 equiv.) were added. After full consumption of the unprotected β-amino amide (monitored by LC/MS), excess benzoyl chloride was quenched by the addition of a saturated aqueous solution of sodium

bicarbonate. The resulting biphasic mixture was extracted with CH_2Cl_2 (twice). The combined organic layers were dried over anhydrous magnesium sulfate, the dried solution was filtered and the filtrate was concentrated under reduced pressure to afford the crude product. At this point, the diastereomeric ratio was determined ^{1}H-NMR analysis (10:1 *d.r.*). The product (3a major diastereomer) was obtained after purification by flash column chromatography on silica gel (Hep/EA 2:1, Rf = 0.3) in 54% yield (1.021 g, 99:1 *e.r.*).

11. Computational Details

The conformational space of all molecules was initially searched using meta-dynamics simulations based on tight-binding quantum chemical calculations as implemented in CREST.[26,27]

The structures located with the CREST were then subjected to B3LYP-D3BJ/def2-SVP[28–34] geometry optimization. The nature of all stationary points (minima and transition states) was verified through the computation of the vibrational frequencies. The thermal corrections to the Gibbs free energy were combined with the single point energies calculated at the DLPNO-CCSD(T)/def2-TZVP[35,36] level of theory to yield DLPNO-CCSD(T)/def2-TZVP//B3LYP-D3BJ/def2-SVP and B3LYP-D3BJ/def2-TZVP//B3LYP-D3BJ/def2-SVP Gibbs free energies ("G_{298}") at 298.15 K. All energies are reported in kcal mol^{-1}.

The polarizable continuum model (PCM) with SMD parameters[37,38] was applied to consider solvent (DCM) effects for both B3LYP-D3BJ geometries and energies. The Conductor-like Polarizable Continuum Model (CPCM) was applied to consider solvent effects at the DLPNO-CCSD(T)/def2-TZVP level of theory.[39] Free energies in solution have been corrected to a reference state of 1 mol l^{-1} at 298.15 K through the addition of RTln(24.46) = +7.925 kJ mol^{-1} to the gas phase (1 atm) free energies.

The DFT calculations were performed with the Gaussian 16 program package[40], and the DLPNO-CCSD(T) with the ORCA 5.0.1.[41] The SAPT analysis was performed at the SAPT0/jun-cc-pvdz level of theory on the optimized geometries applying the PSI4 software.[42, 43, 44]

The influence of dispersion effects on the transition states structures

Supplementary Table S3 | Structural parameters of the transition state optimized at the DFT level of theory, applying dispersion correction (B3LYP-D3BJ) and neglecting dispersion effects (B3LYP).

Transition state	Bond lengths, Å B3LYP-D3BJ/def2-SVP	Bond lengths, Å B3LYP/def2-SVP	Bond lengths change, Å
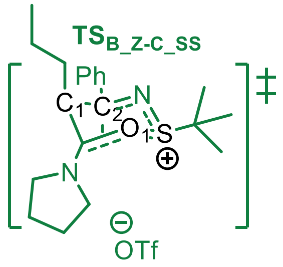	r(C1-C2) = 2.855<br>r(S-O1) = 2.071	r(C1-C2) = 3.766<br>r(S-O1) = 2.273	Δr(C1-C2) = 0.911<br>Δr(S-O1) = 0.202
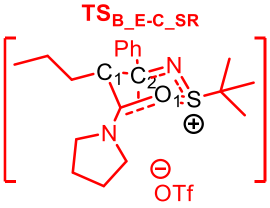	r(C1-C2) = 2.694<br>r(S-O1) = 1.845	r(C1-C2) = 2.910<br>r(S-O1) = 1.986	Δr(C1-C2) = 0.216<br>Δr(S-O1) = 0.141

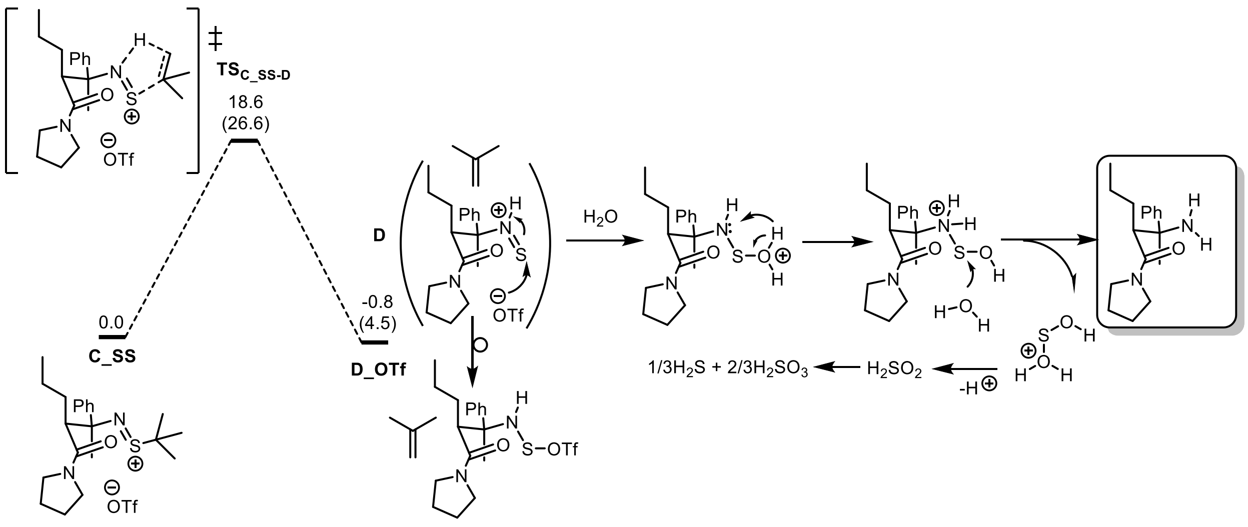

Figure S1 Computed reaction profile (B3LYP-D3(BJ)/def2-TZVP//B3LYP-D3(BJ)/def2-SVP, $\Delta G_{298,\ DCM}$) for the intramolecular C–S bond cleavage in the intermediate **C_SS** (taken as a reference 0.0 kcal mol^{-1}), and the hypothetical path to the experimentally observed product. The energy values in brackets are computed at the DLPNO-CCSD(T)/def2-TZVP//B3LYP-D3(BJ)/def2-SVP level of theory.

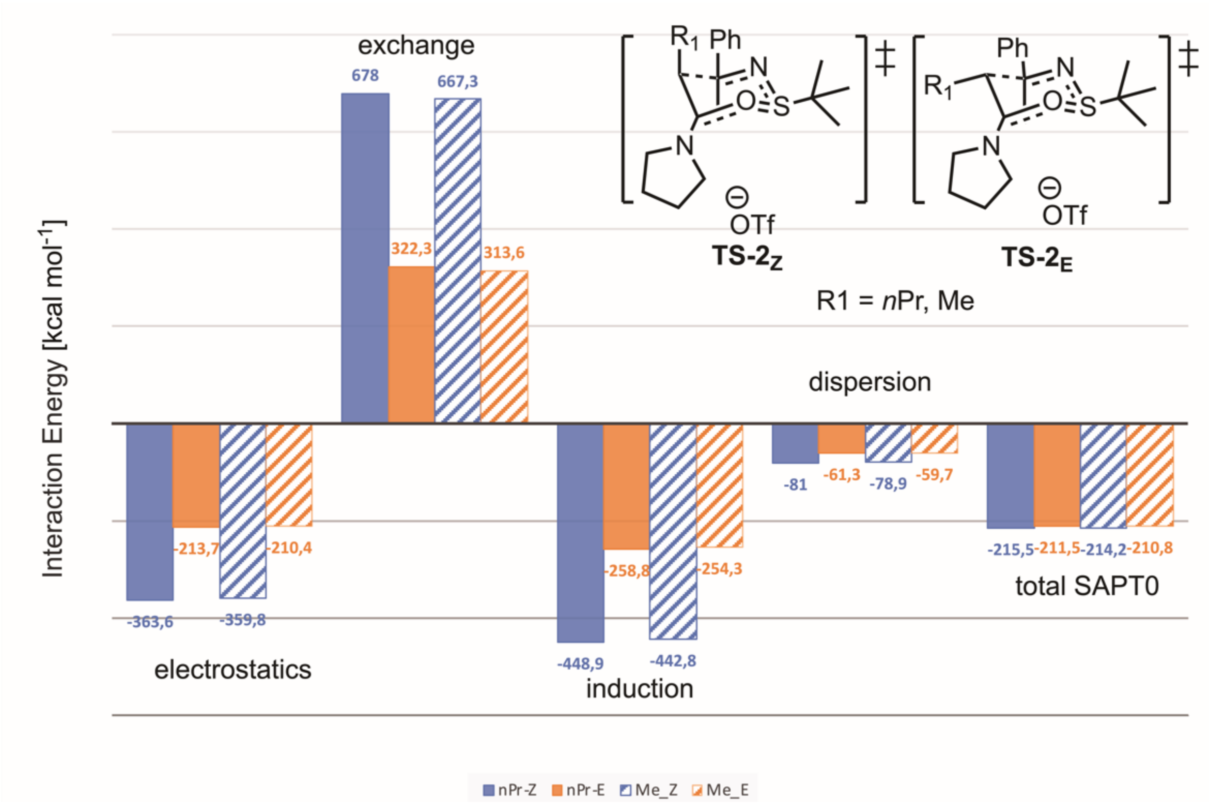

Figure S2 SAPT0 analysis of **TS-2$_Z$** and **TS-2$_E$** for the *n*-propyl (solid bars) and methyl (hatched bars) substituents R_1 in comparison. Level of theory: SAPT0/jun-cc-pvdz.

12. X-Ray Crystallographic data for compound 3a

The X-ray intensity data was measured on Bruker D8 Venture diffractometer equipped with multilayer monochromator, Mo K/α INCOATEC micro focus sealed tube and Oxford cooling system. The structure was solved by *Direct Methods*. Non-hydrogen atoms were refined with *anisotropic displacement parameters*. Hydrogen atoms were inserted at calculated positions and refined with riding model. The following software was used: *Bruker SAINT software package*[i] using a narrow-frame algorithm for frame integration, *SADABS*[ii] for absorption correction, *OLEX2*[iii] for structure solution, refinement, molecular diagrams and graphical user-interface, *Shelxle*[iv] for refinement and graphical user-interface *SHELXS-2015*[v] for structure solution, *SHELXL-2015*[vi] for refinement, *Platon*[vii] for symmetry check. Experimental data and CCDC-Codes Experimental data (Available online: http://www.ccdc.cam.ac.uk/conts/retrieving.html) can be found in Table S4. Crystal data, data collection parameters, and structure refinement details are given in Table S5. Asymmetric Unit visualized in Figure S3.

Supplementary Table S4 Experimental parameter and CCDC-Code.

**Sample**	**Machine**	**Source**	**Temp.**	**Detector Distance**	**Time/ Frame**	**#Frames**	**Frame width**	**CCDC**
	Bruker		[K]	[mm]	[s]		[°]	
**3a**	D8	Mo	150	40	5	4969	0.360	2153021

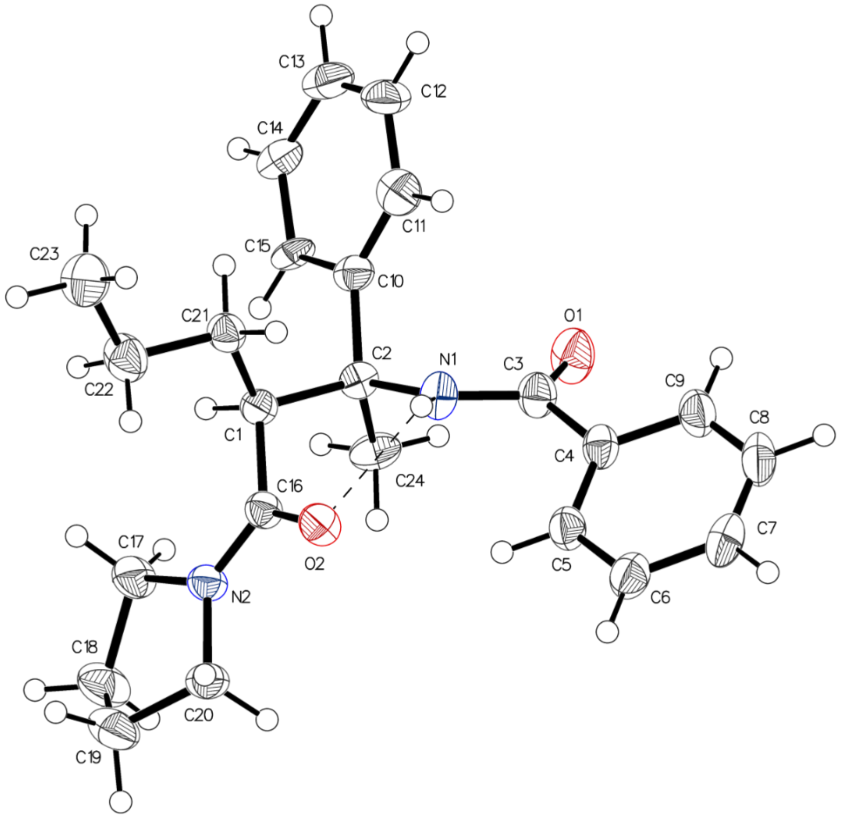

Figure S3. Asymmetric Unit of drawn with 50% displacement ellipsoid. The bond precision for C-C single bonds is 0.0023 Å. Disorder omitted for clarity. The degree of Disorder is 18% in two parts for the atoms located on the phenyl ring C11-C15. The chiral interpretation with the help of Flack and Hooft parameter (-0.2,-0.1) is, with respect to the weak scattering power in the molecule, acceptable and can be determined for C1 and C2 (S,S). One intra-molecular hydrogen bond of moderate to weak character (according to Jeffrey 1997) is located at N1H to O2. The packing is characterized by VdW interactions and so far not visualized separately.

Supplementary Table S5 Sample and crystal data, Data collection and structure refinement

Identification code	mf131p_P21
Empirical formula	$C_{24}H_{30}N_2O_2$
Formula weight	378.50
Temperature/K	150.0
Crystal system	monoclinic
Space group	$P2_1$
a/Å	9.0305(6)
b/Å	12.7227(9)
c/Å	9.4541(5)
α/°	90
β/°	101.223(2)
γ/°	90
Volume/Å^3	1065.43(12)
Z	2
ρ_{calc}g/cm^3	1.180
μ/mm^{-1}	0.075
F(000)	408.0
Crystal size/mm^3	0.222 × 0.131 × 0.08
Radiation	MoKα (λ = 0.71073)
2Θ range for data collection/°	4.392 to 64.108
Index ranges	-13 ≤ h ≤ 13, -18 ≤ k ≤ 18, -14 ≤ l ≤ 14
Reflections collected	85794

Independent reflections	7429 [R_{int} = 0.0537, R_{sigma} = 0.0286]
Data/restraints/parameters	7429/12/300
Goodness-of-fit on F^2	1.084
Final R indexes [I>=2σ (I)]	R_1 = 0.0382, wR_2 = 0.0987
Final R indexes [all data]	R_1 = 0.0508, wR_2 = 0.1032
Largest diff. peak/hole / e Å^{-3}	0.26/-0.23
Flack parameter	-0.1(3)

----

[i] Bruker SAINT v8.38B Copyright © 2005-2019 Bruker AXS

[ii] Sheldrick, G. M. (1996). *SADABS.* University of Göttingen, Germany.

[iii] Dolomanov, O.V., Bourhis, L.J., Gildea, R.J, Howard, J.A.K. & Puschmann, H. , OLEX2, (2009), J. Appl. Cryst. 42, 339-341.

[iv] C. B. Huebschle, G. M. Sheldrick and B. Dittrich, ShelXle: a Qt graphical user interface for SHELXL, J. Appl. Cryst., 44, (2011) 1281-1284.

[v] Sheldrick, G. M. (2015). *SHELXS v 2016/4* University of Göttingen, Germany.

[vi] Sheldrick, G. M. (2015). *SHELXL v 2016/4* University of Göttingen, Germany.

[vii] A. L. Spek, Acta Cryst. 2009, D65, 148-155.

13. NMR spectra

MF293

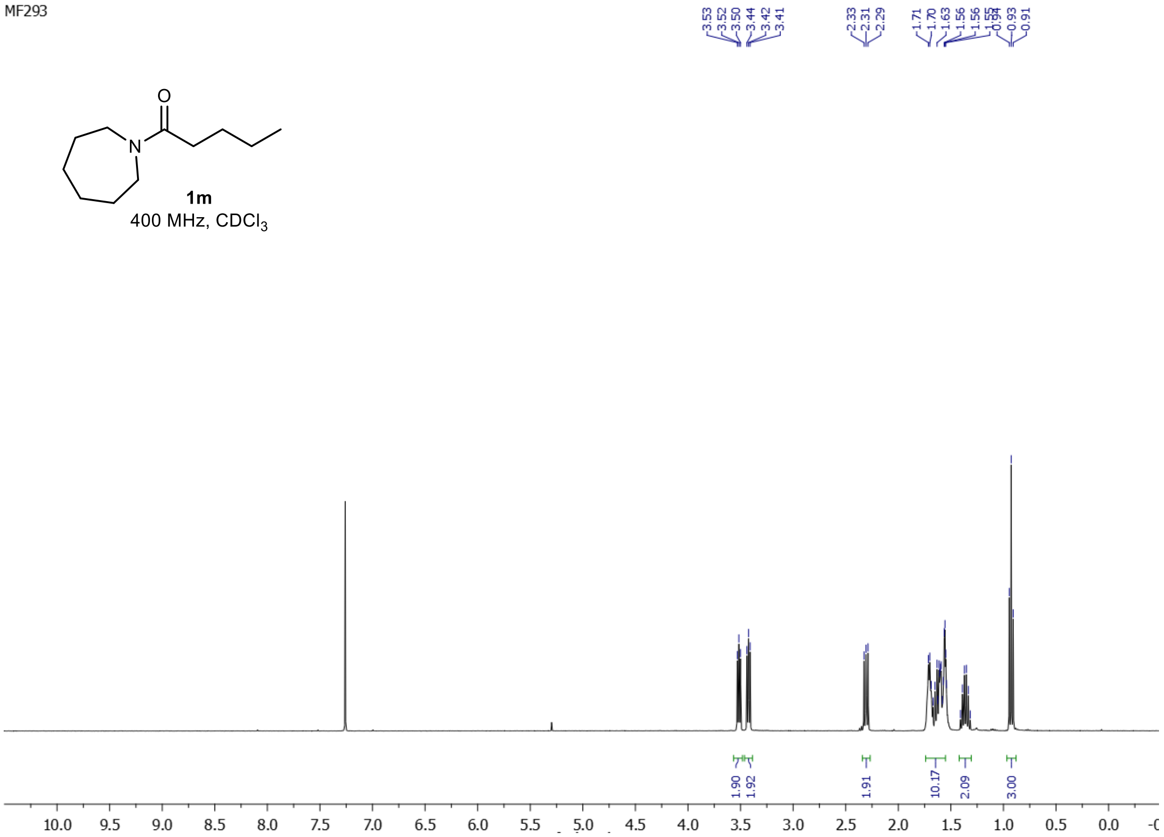

1m
400 MHz, $CDCl_3$

MF293

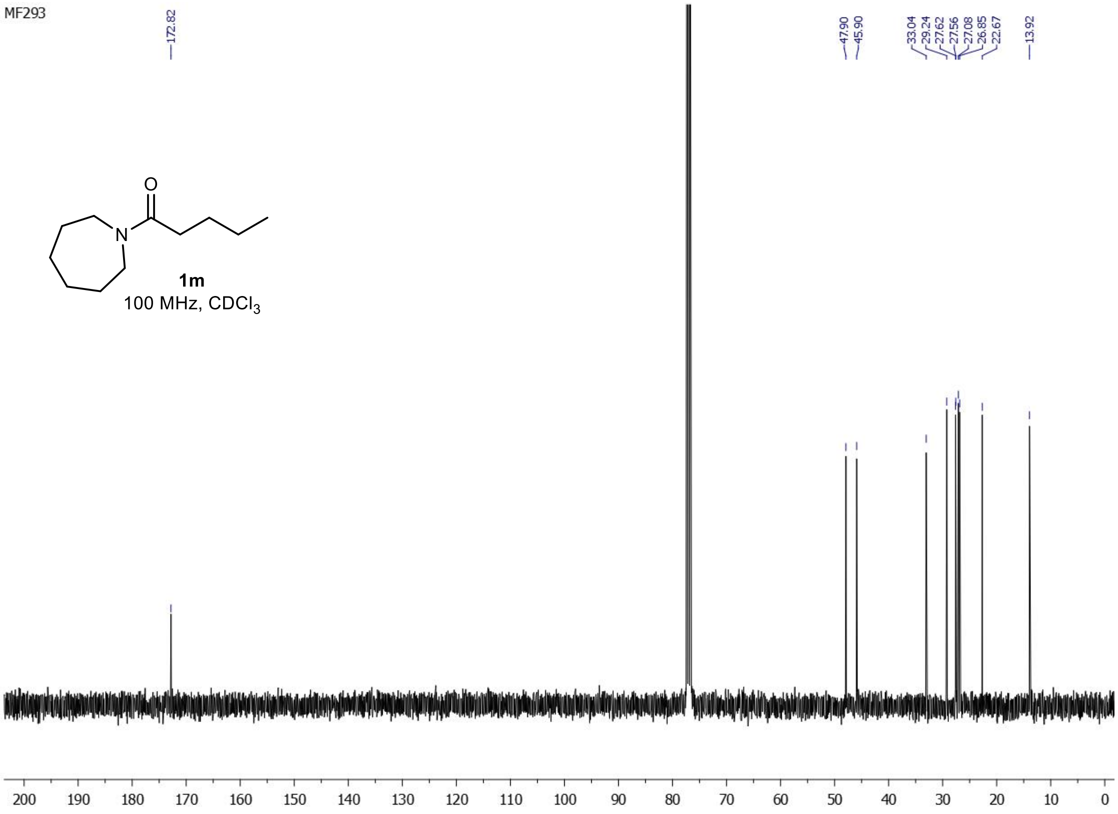

1m
100 MHz, $CDCl_3$

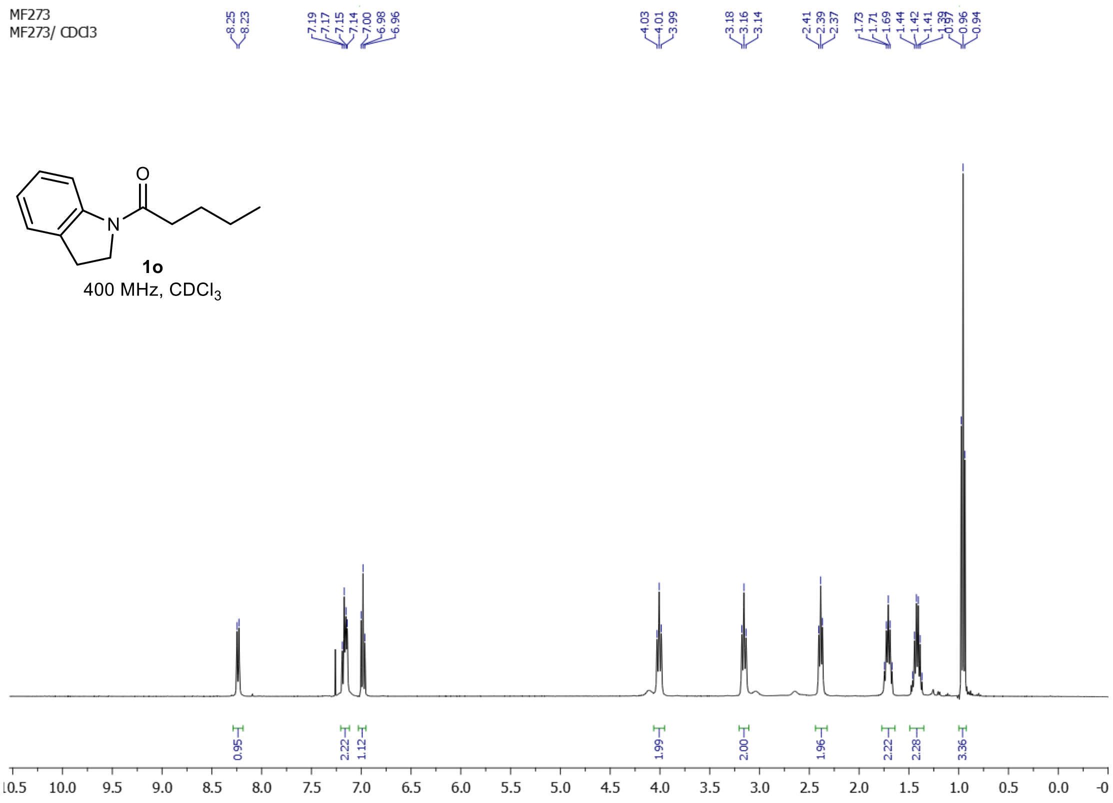
MF273
MF273/ CDCl3
1o
400 MHz, CDCl$_3$

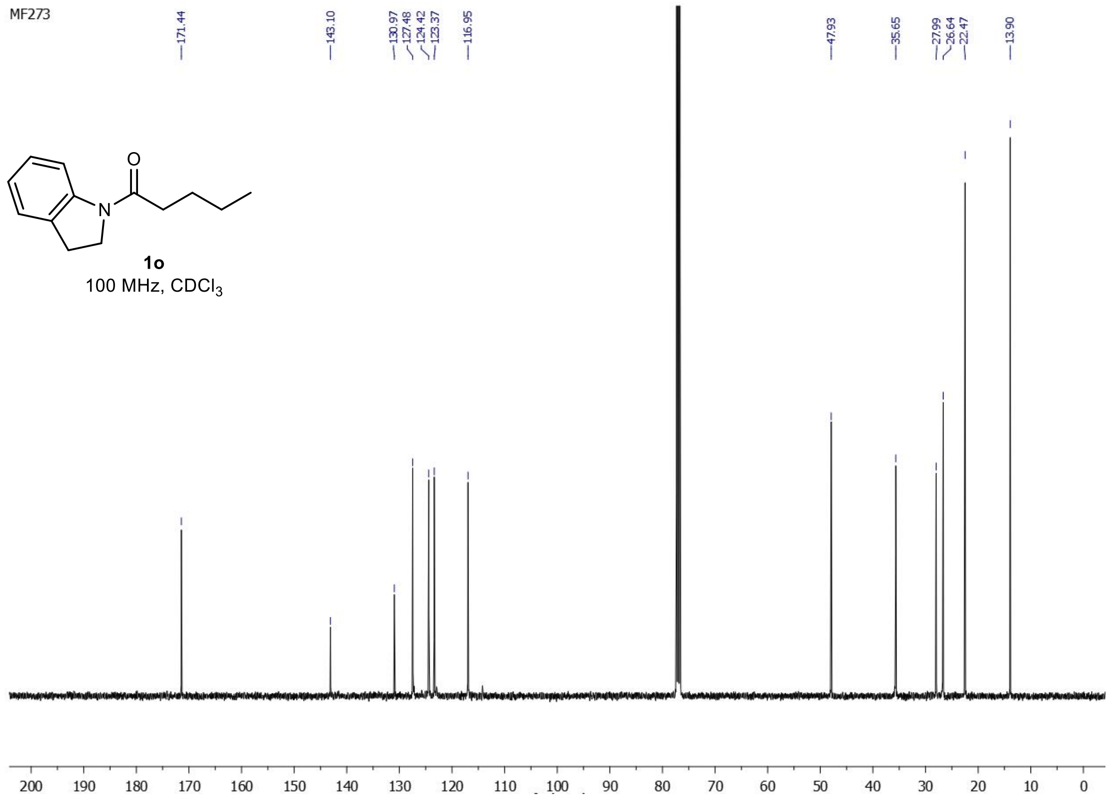
MF273
1o
100 MHz, CDCl$_3$

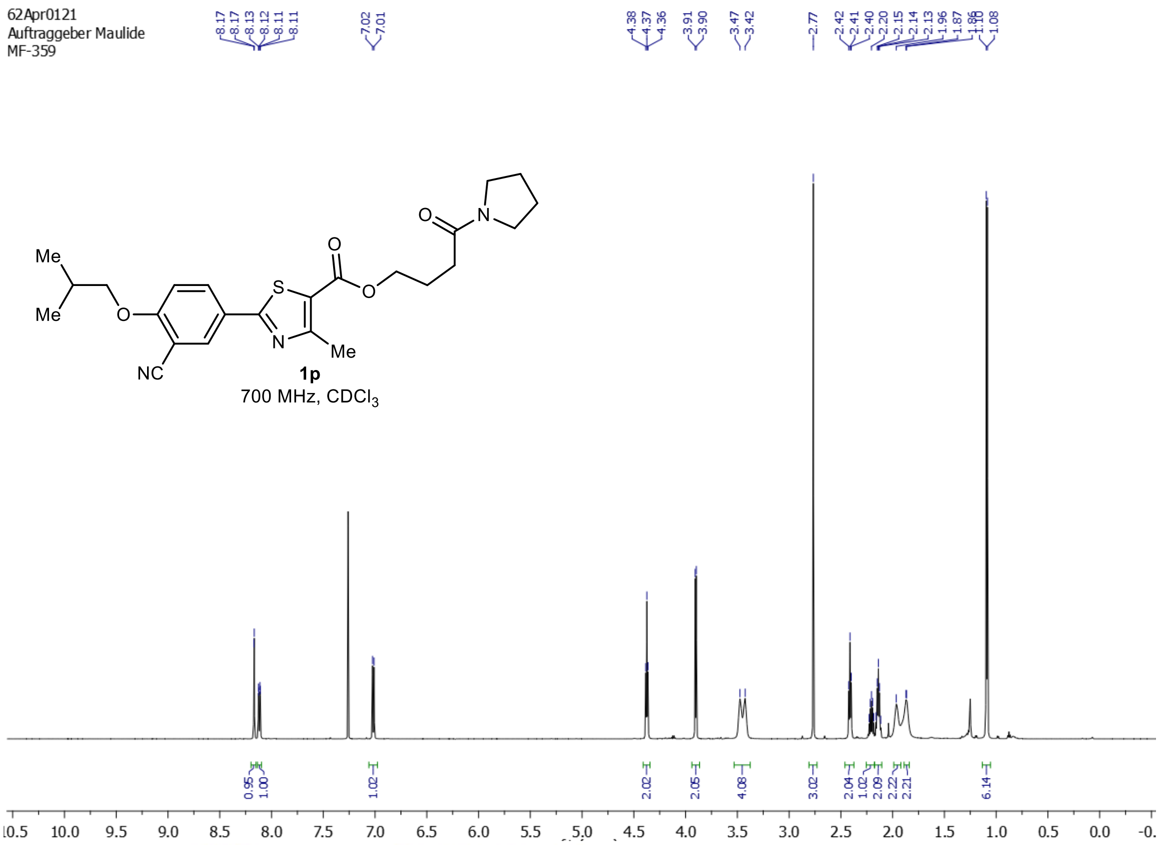
62Apr0121
Auftraggeber Maulide
MF-359
8.17 8.17 8.13 8.12 8.11 8.11 7.02 7.01 4.38 4.37 4.36 3.91 3.90 3.47 3.42 2.77 2.42 2.41 2.40 2.20 2.15 2.14 2.13 1.96 1.87 1.86 1.10 1.08
Me
Me
O
NC
S
N
Me
O
O
O
N
1p
700 MHz, CDCl3
0.95 1.00 1.02 2.02 2.05 4.08 3.02 2.04 1.02 2.09 2.22 2.21 6.14
10.5 10.0 9.5 9.0 8.5 8.0 7.5 7.0 6.5 6.0 5.5 5.0 4.5 4.0 3.5 3.0 2.5 2.0 1.5 1.0 0.5 0.0 -0.

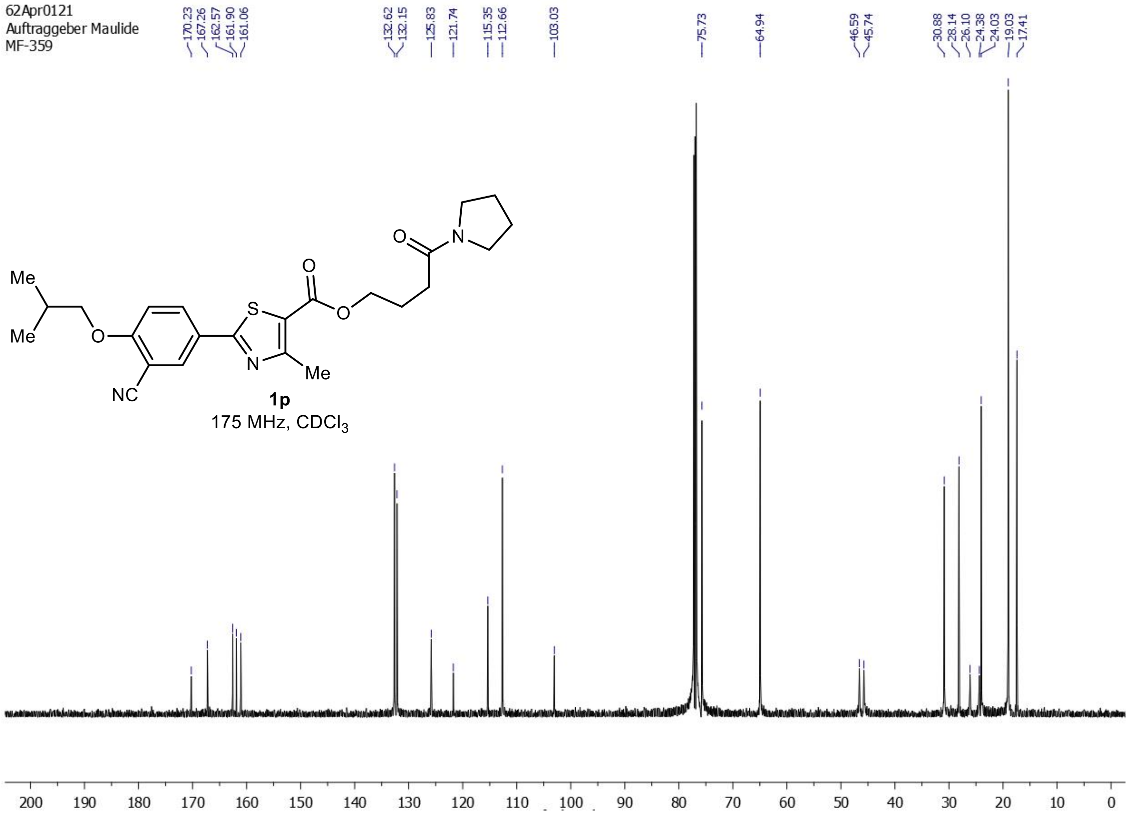
62Apr0121
Auftraggeber Maulide
MF-359
170.23 167.26 162.57 161.90 161.06 132.62 132.15 125.83 121.74 115.35 112.66 103.03 75.73 64.94 46.59 45.74 30.88 28.14 26.10 24.38 24.03 19.03 17.41
Me
Me
O
NC
S
N
Me
O
O
O
N
1p
175 MHz, CDCl3
200 190 180 170 160 150 140 130 120 110 100 90 80 70 60 50 40 30 20 10 0

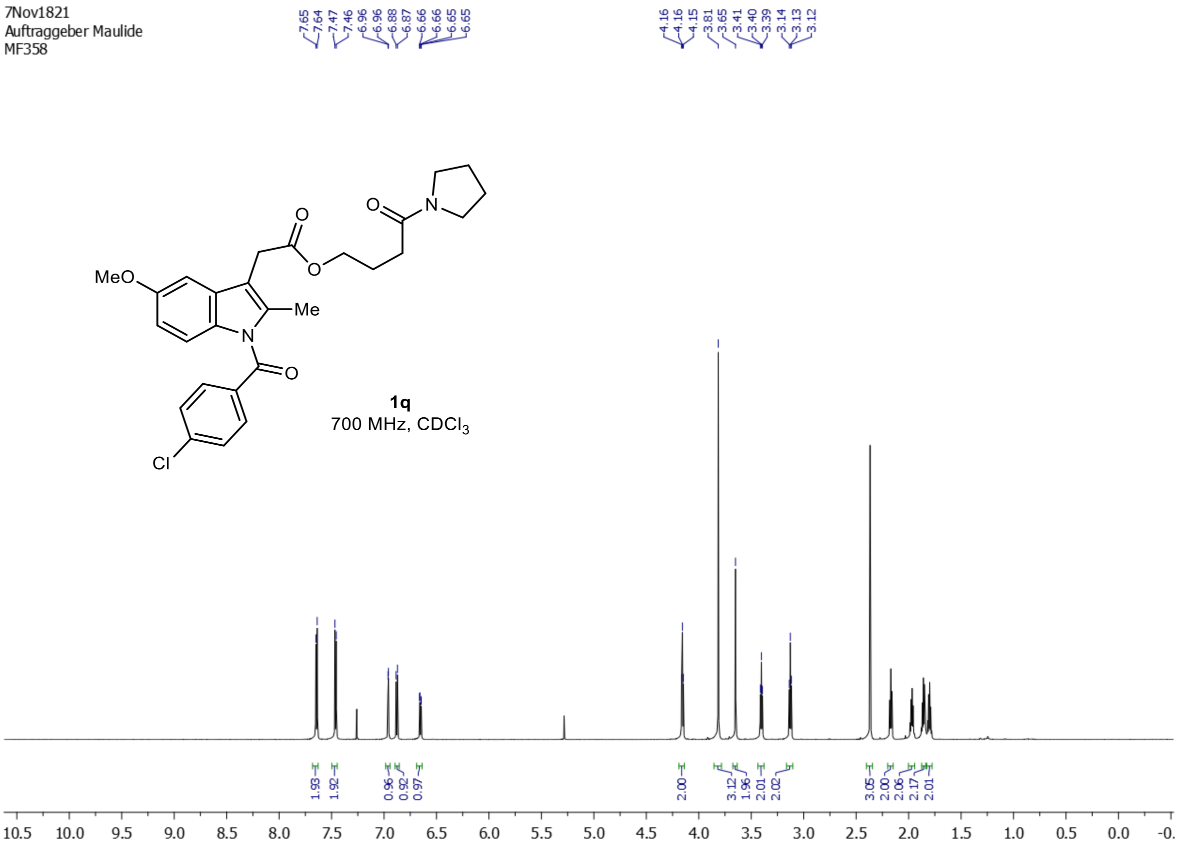
7Nov1821
Auftraggeber Maulide
MF358
7.65
7.64
7.47
7.46
6.96
6.96
6.88
6.87
6.66
6.66
6.65
6.65
4.16
4.16
4.15
3.81
3.65
3.41
3.40
3.39
3.14
3.13
3.12
MeO
Me
N
O
O
O
O
N
Cl
1q
700 MHz, CDCl3
1.93
1.92
0.96
0.92
0.97
2.00
3.12
1.96
2.01
2.02
3.05
2.00
2.06
2.17
2.01
10.5
10.0
9.5
9.0
8.5
8.0
7.5
7.0
6.5
6.0
5.5
5.0
4.5
4.0
3.5
3.0
2.5
2.0
1.5
1.0
0.5
0.0
-0.

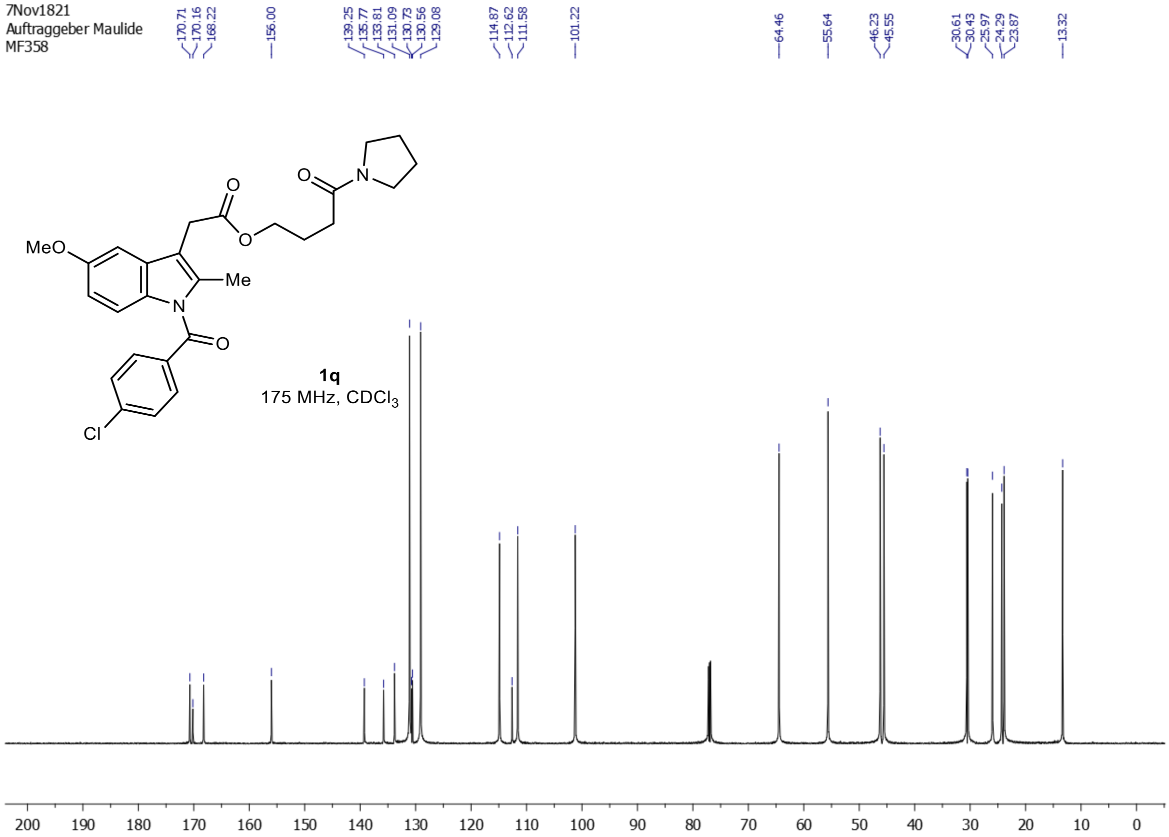
7Nov1821
Auftraggeber Maulide
MF358
170.71
170.16
168.22
156.00
139.25
135.77
133.81
131.09
130.73
130.56
129.08
114.87
112.62
111.58
101.22
64.46
55.64
46.23
45.55
30.61
30.43
25.97
24.29
23.87
13.32
MeO
Me
N
O
O
O
O
N
Cl
1q
175 MHz, CDCl3
200
190
180
170
160
150
140
130
120
110
100
90
80
70
60
50
40
30
20
10
0

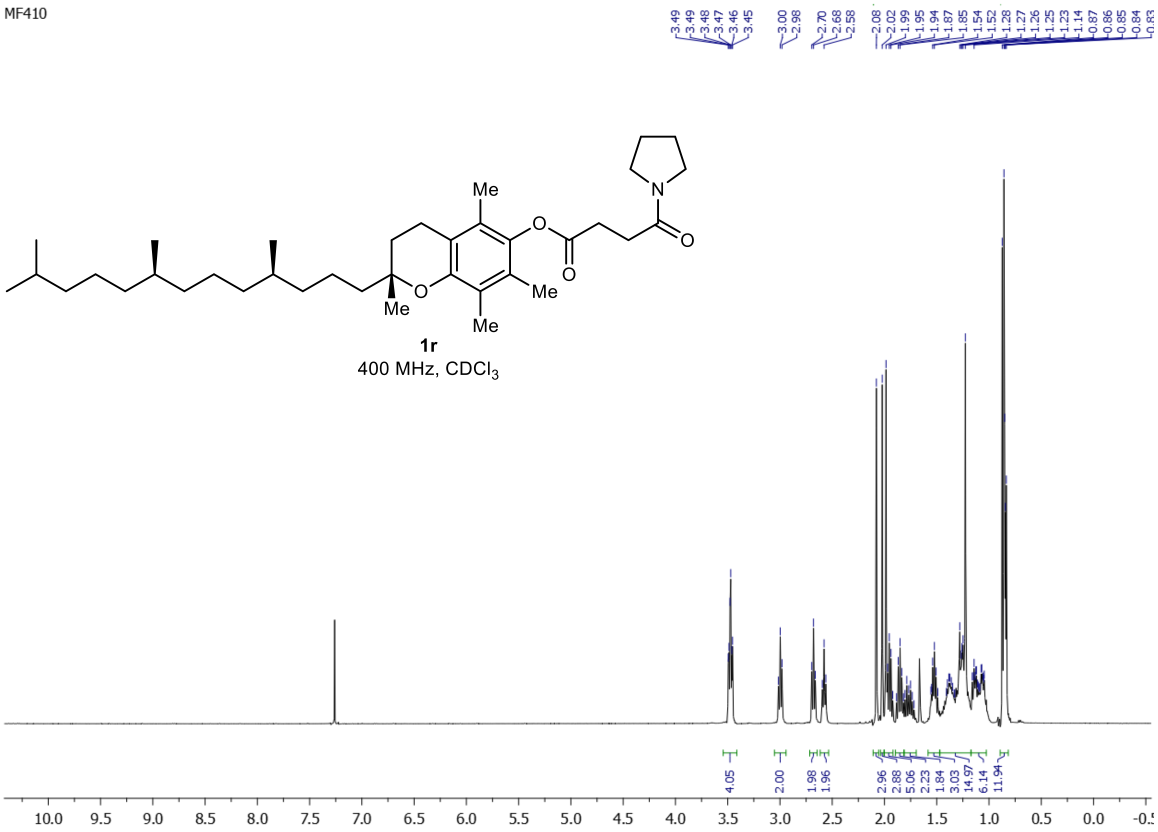
MF410
3.49 3.49 3.48 3.47 3.46 3.45 3.00 2.98 2.70 2.68 2.58 2.08 2.02 1.99 1.95 1.94 1.87 1.85 1.54 1.52 1.28 1.27 1.26 1.25 1.23 1.14 0.87 0.86 0.85 0.84
Me
O
N
Me
O
O
O
Me
Me
1r
400 MHz, CDCl3
4.05 2.00 1.98 1.96 2.96 2.88 5.06 2.23 1.84 3.03 14.97 6.14 11.94
10.0 9.5 9.0 8.5 8.0 7.5 7.0 6.5 6.0 5.5 5.0 4.5 4.0 3.5 3.0 2.5 2.0 1.5 1.0 0.5 0.0

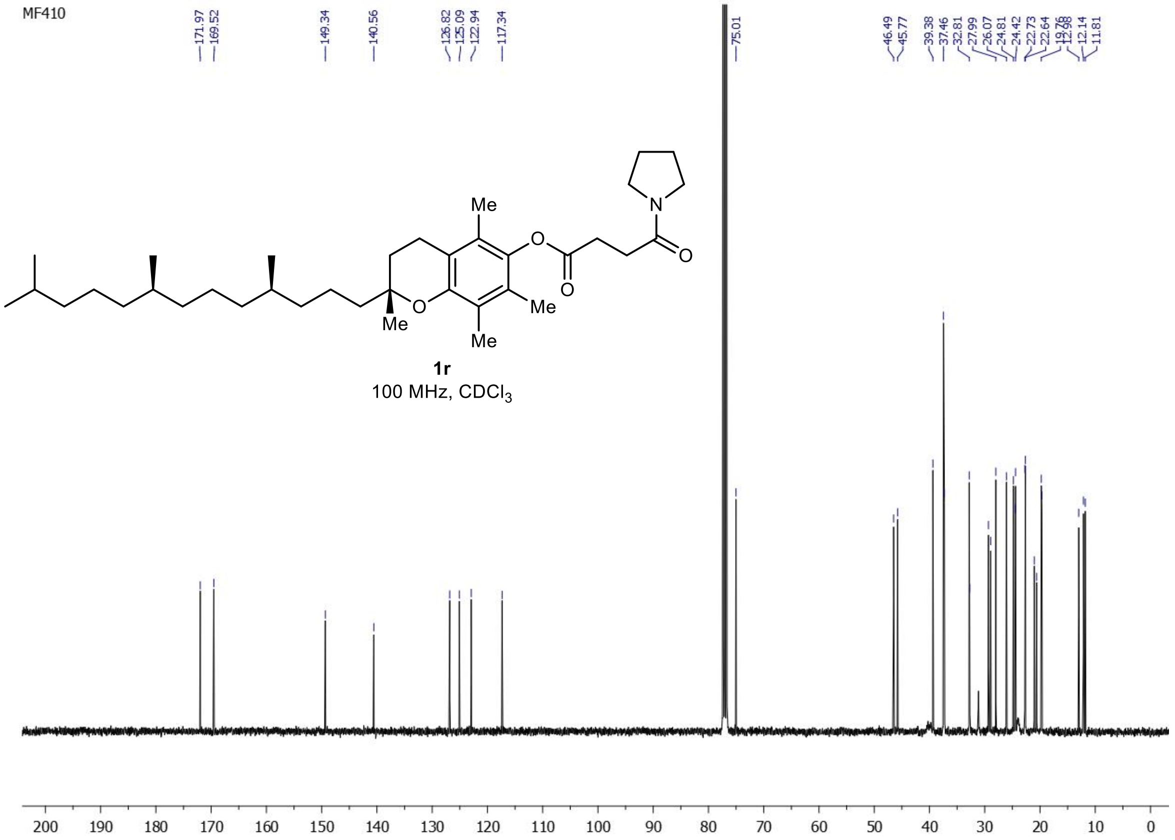
MF410
171.97 169.52 149.34 140.56 126.82 125.09 122.94 117.34 75.01 46.49 45.77 39.38 37.46 32.81 27.99 26.07 24.81 24.42 22.73 22.64 12.14 11.81
Me
O
N
Me
O
O
O
Me
Me
1r
100 MHz, CDCl3
200 190 180 170 160 150 140 130 120 110 100 90 80 70 60 50 40 30 20 10 0

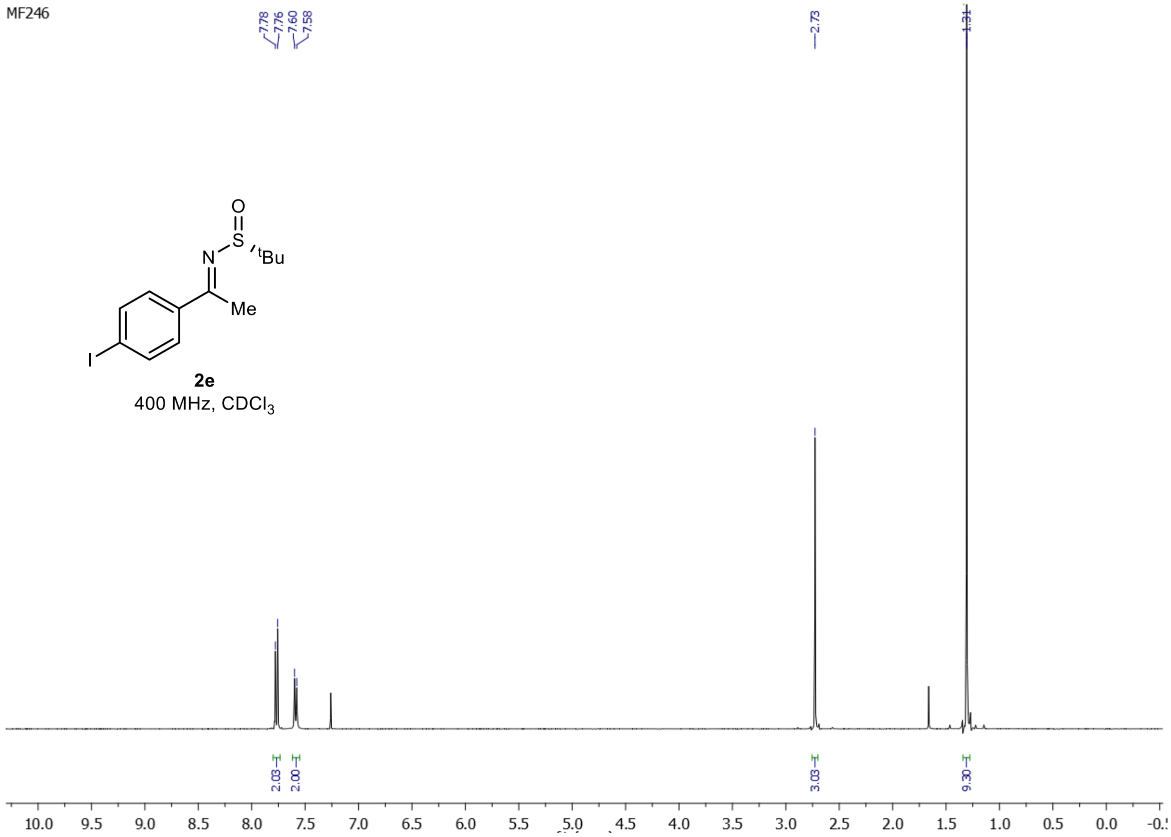
MF246
7.78
7.76
7.60
7.58
2.73
1.31
O
S
N
tBu
Me
I
2e
400 MHz, CDCl3
2.03
2.00
3.03
9.30
10.0
9.5
9.0
8.5
8.0
7.5
7.0
6.5
6.0
5.5
5.0
4.5
4.0
3.5
3.0
2.5
2.0
1.5
1.0
0.5
0.0

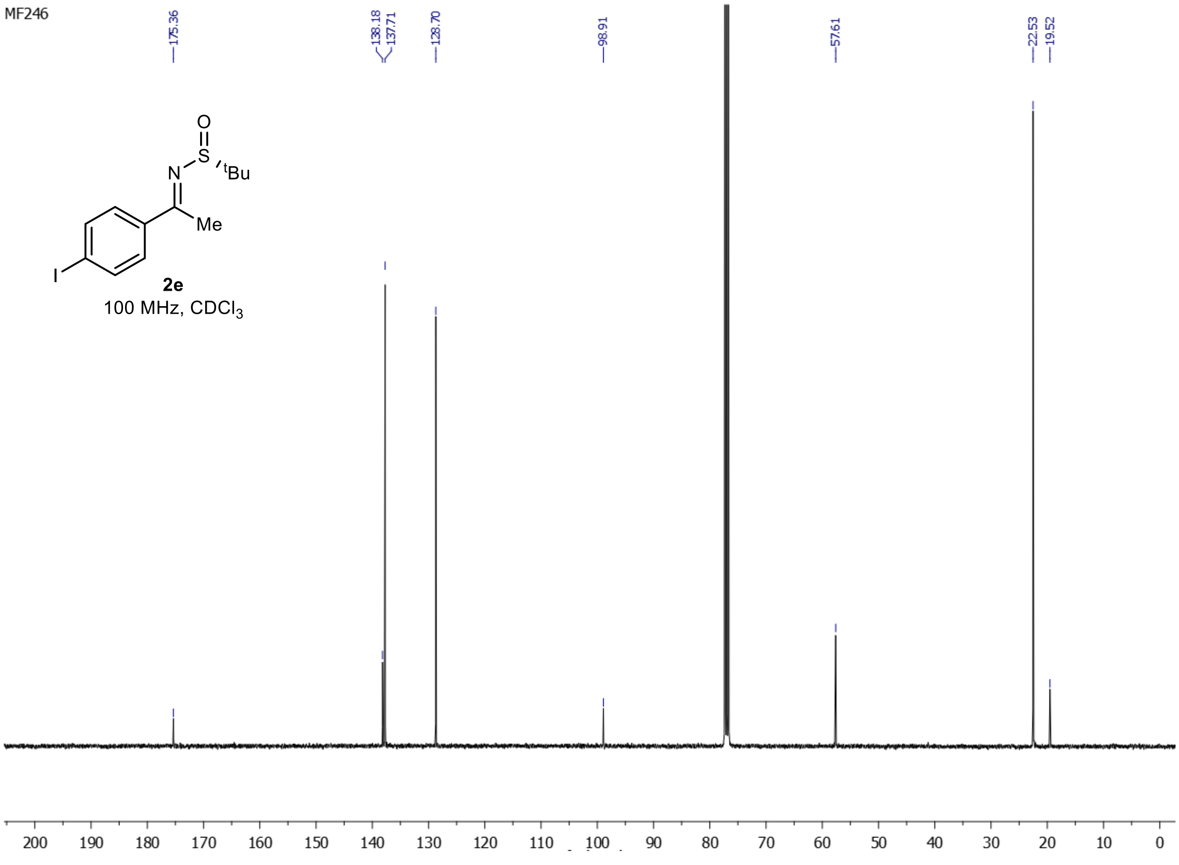
MF246
175.36
138.18
137.71
128.70
98.91
57.61
22.53
19.52
O
S
N
tBu
Me
I
2e
100 MHz, CDCl3
200
190
180
170
160
150
140
130
120
110
100
90
80
70
60
50
40
30
20
10
0

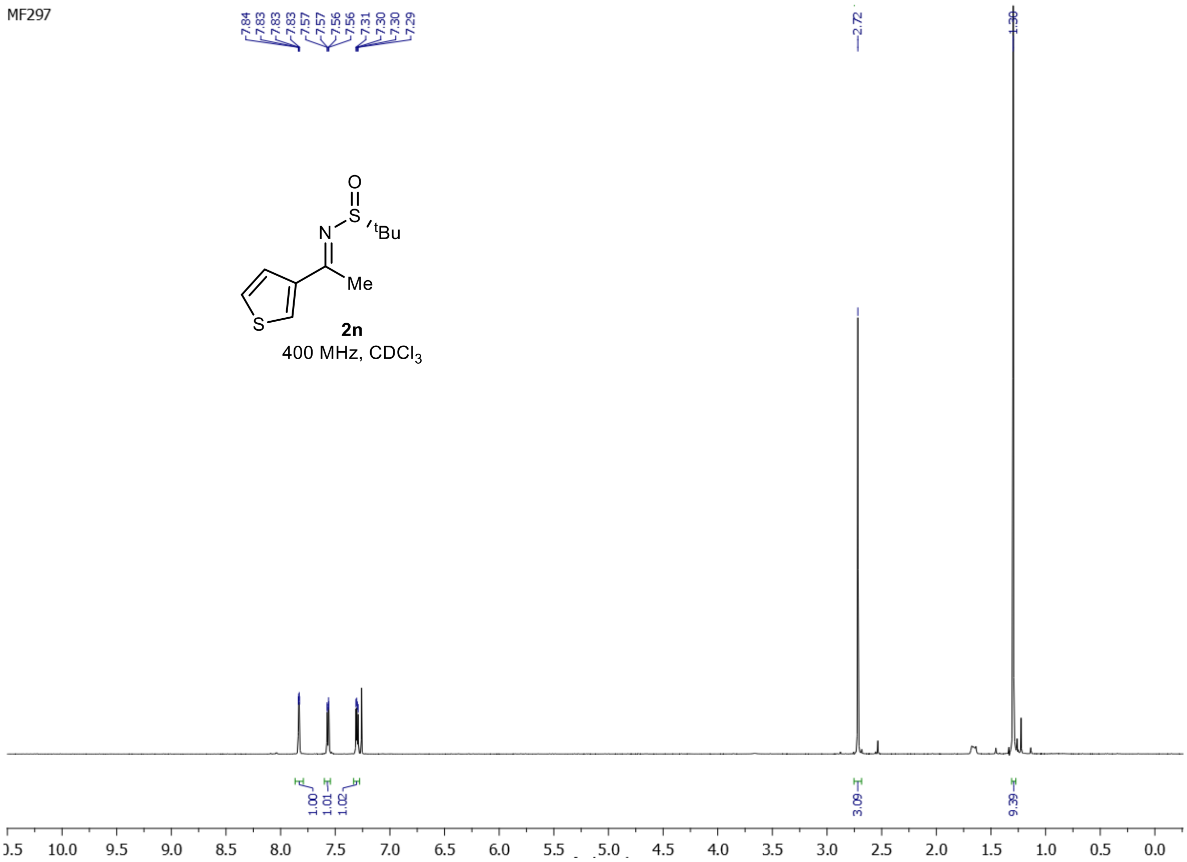
MF297
7.84
7.83
7.83
7.83
7.57
7.57
7.56
7.56
7.31
7.30
7.30
7.29
2.72
1.30
O
S
N
tBu
Me
S
2n
400 MHz, CDCl3
1.00
1.01
1.02
3.09
9.39
10.0
9.5
9.0
8.5
8.0
7.5
7.0
6.5
6.0
5.5
5.0
4.5
4.0
3.5
3.0
2.5
2.0
1.5
1.0
0.5
0.0

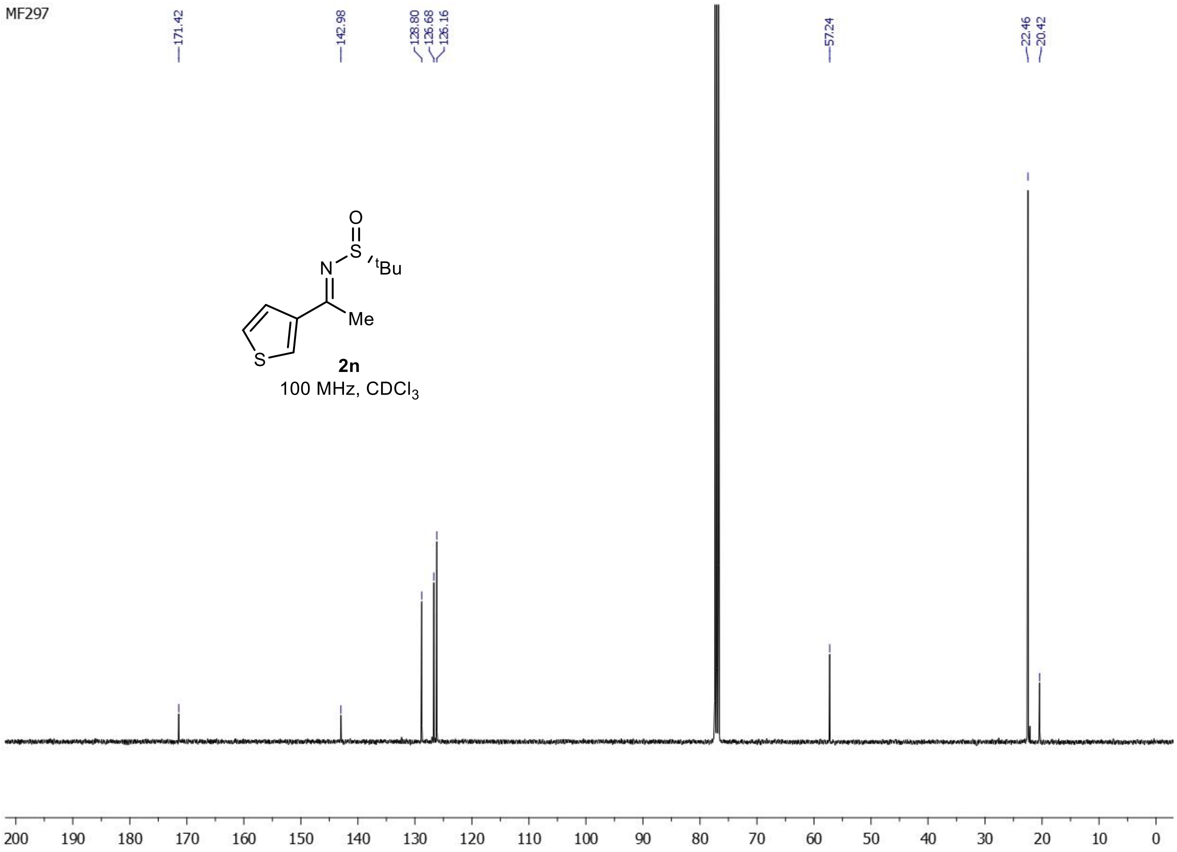
MF297
171.42
142.98
128.80
126.68
126.16
57.24
22.46
20.42
O
S
N
tBu
Me
S
2n
100 MHz, CDCl3
200
190
180
170
160
150
140
130
120
110
100
90
80
70
60
50
40
30
20
10
0

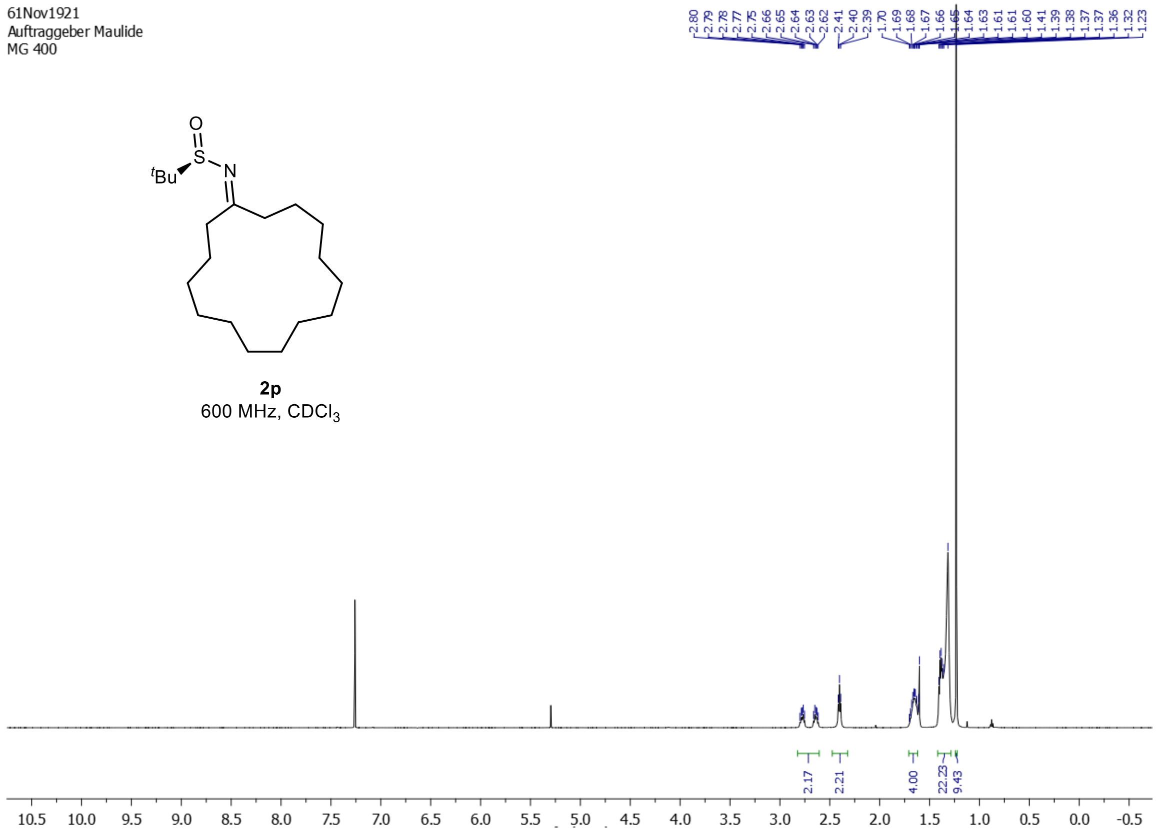

2p
600 MHz, $CDCl_3$

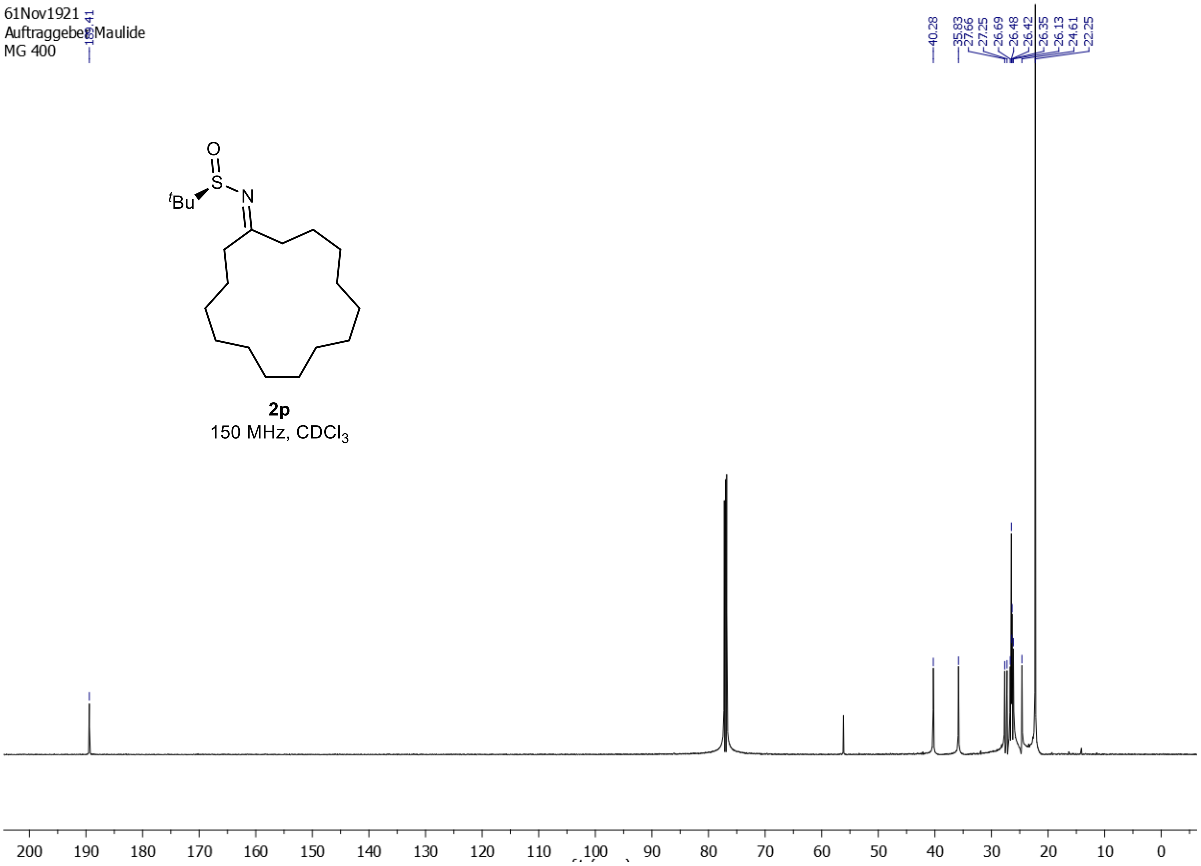

2p
150 MHz, $CDCl_3$

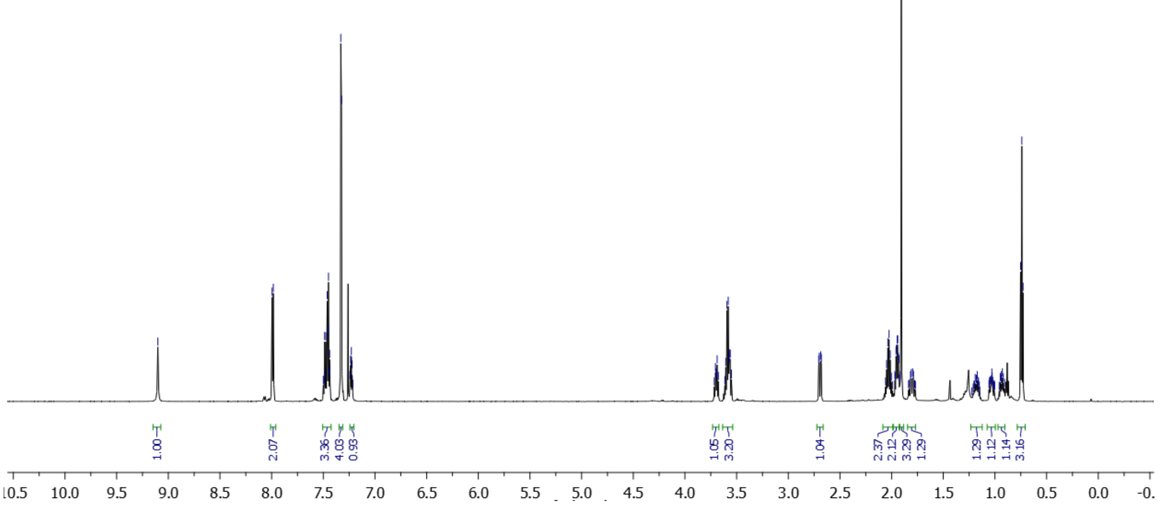

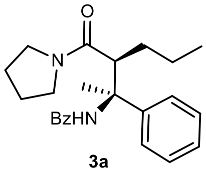

3a
600 MHz, $CDCl_3$

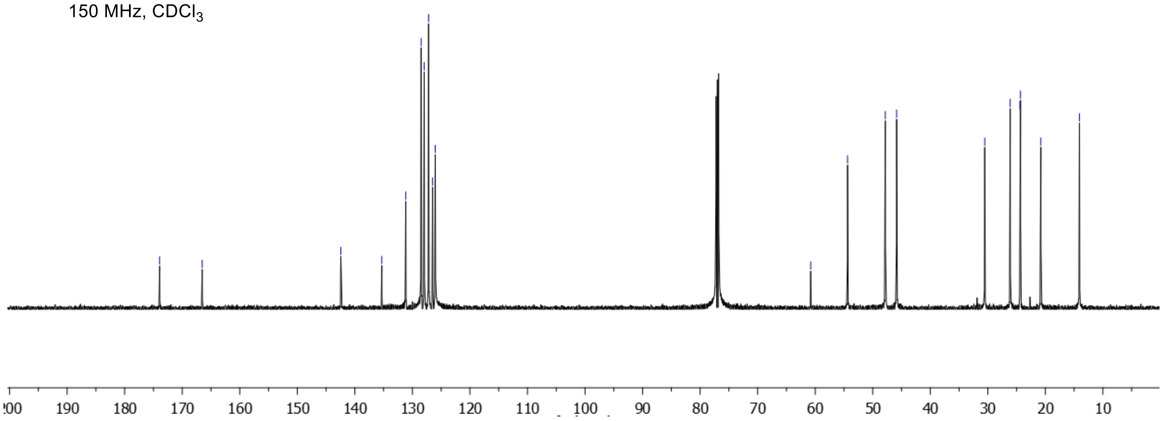

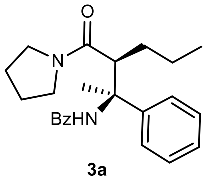

3a
150 MHz, $CDCl_3$

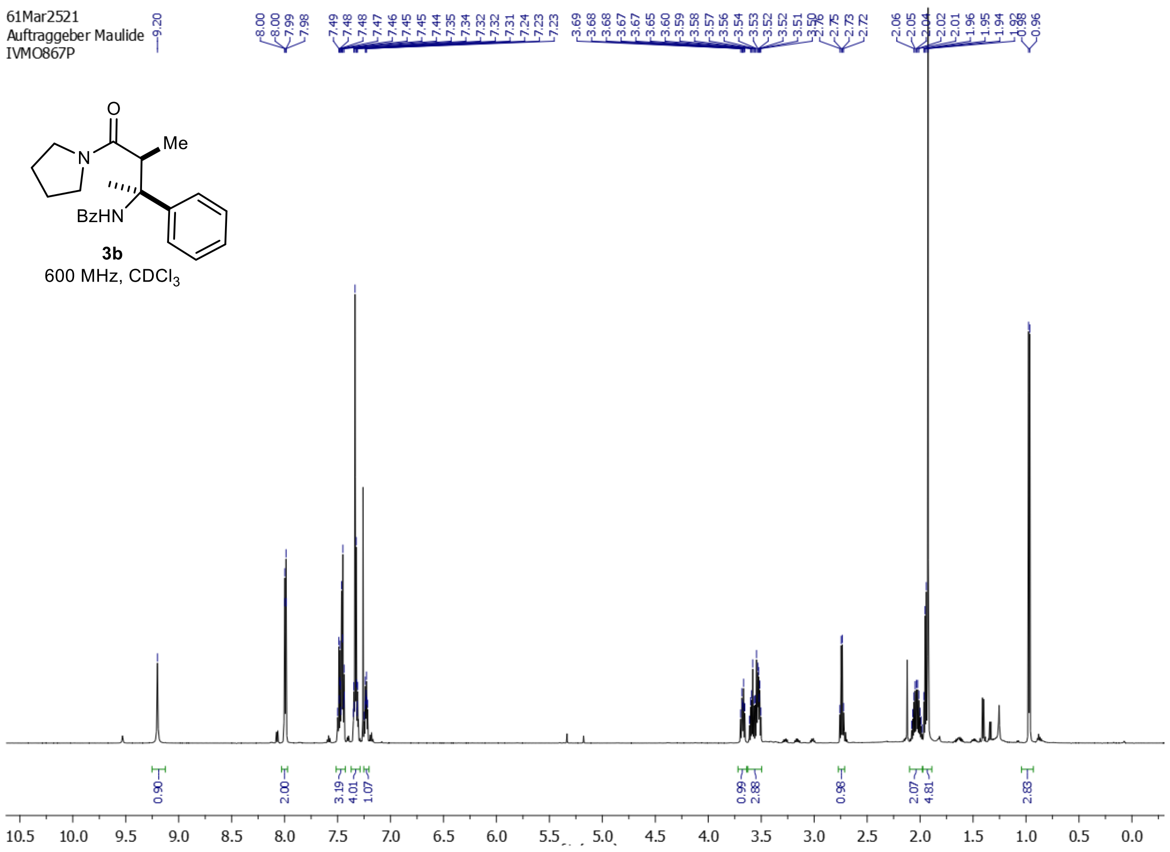
61Mar2521
Auftraggeber Maulide
IVMO867P
9.20
8.00 8.00 7.99 7.98
7.49 7.48 7.48 7.47 7.46 7.45 7.45 7.44 7.35 7.34 7.32 7.32 7.31 7.24 7.23 7.23
3.69 3.68 3.68 3.67 3.67 3.65 3.60 3.59 3.58 3.57 3.56 3.54 3.53 3.52 3.52 3.51
2.75 2.73 2.72
2.06 2.05 2.02 2.01 1.96 1.95 1.94
0.96
O
N
Me
BzHN
3b
600 MHz, $CDCl_3$
0.90
2.00
3.19 4.01 1.07
0.99 2.88
0.98
2.07 4.81
2.83
10.5 10.0 9.5 9.0 8.5 8.0 7.5 7.0 6.5 6.0 5.5 5.0 4.5 4.0 3.5 3.0 2.5 2.0 1.5 1.0 0.5 0.0

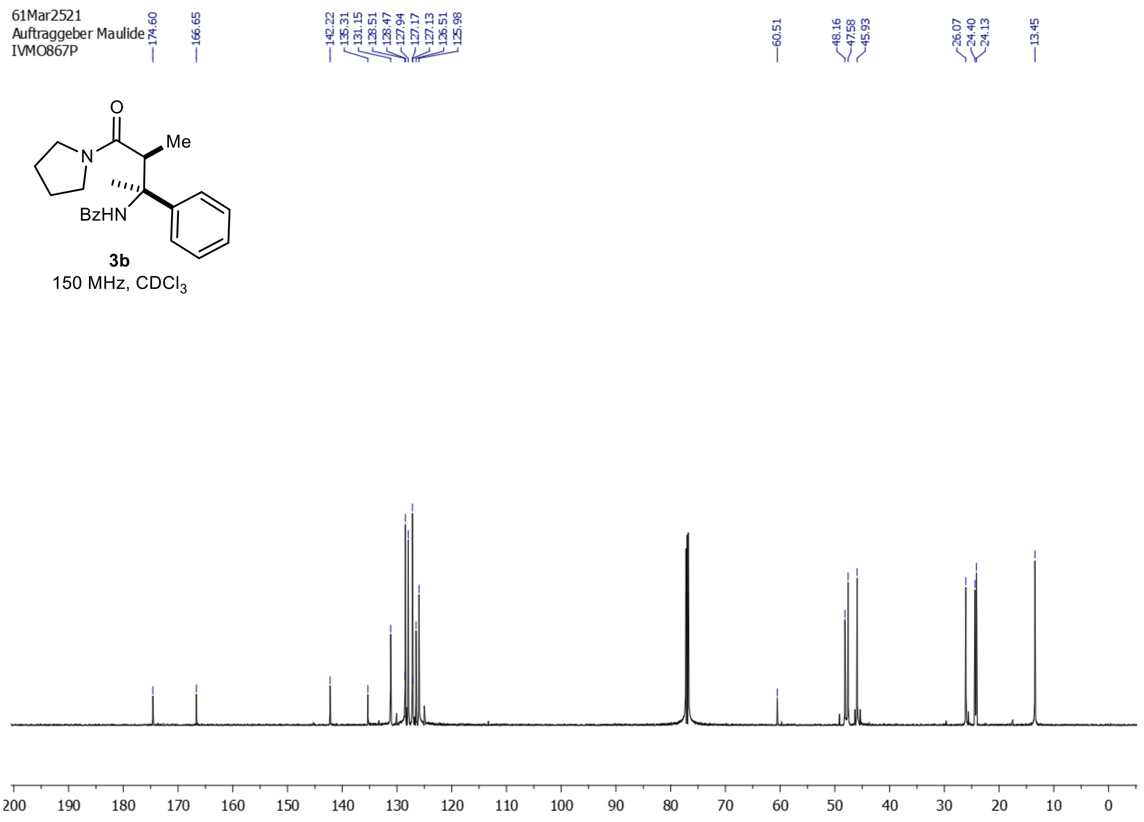
61Mar2521
Auftraggeber Maulide
IVMO867P
174.60
166.65
142.22 135.31 131.15 128.51 128.47 127.94 127.17 127.13 126.51 125.98
60.51
48.16 47.58 45.93
26.07 24.40 24.13
13.45
O
N
Me
BzHN
3b
150 MHz, $CDCl_3$
200 190 180 170 160 150 140 130 120 110 100 90 80 70 60 50 40 30 20 10 0

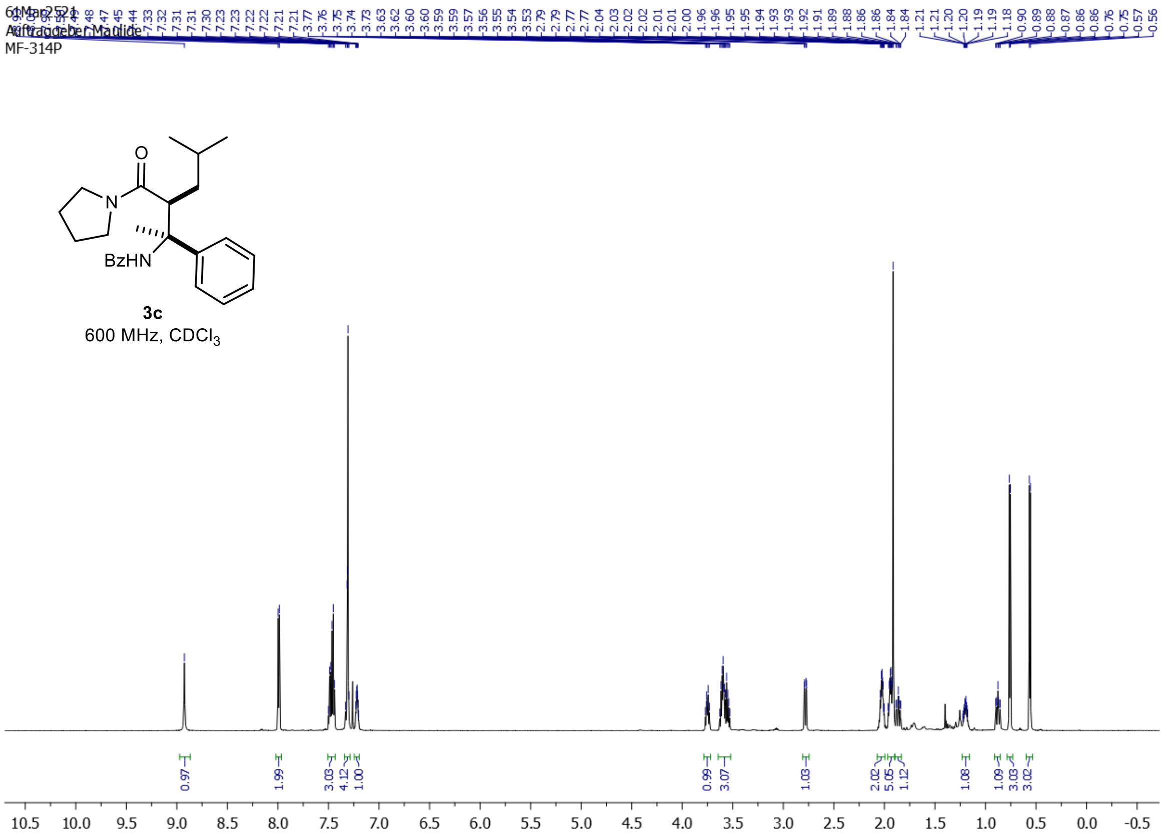
61Mar2521
Auftraggeber Maulide
MF-314P
7.33 7.32 7.31 7.31 7.30 7.23 7.23 7.22 7.22 7.21 7.21 3.77 3.76 3.75 3.74 3.73 3.63 3.62 3.60 3.60 3.59 3.59 3.57 3.56 3.55 3.54 3.53 2.79 2.79 2.77 2.77 2.04 2.03 2.02 2.02 2.01 2.01 2.00 1.96 1.96 1.95 1.95 1.94 1.93 1.93 1.92 1.91 1.89 1.88 1.86 1.86 1.84 1.84 1.21 1.21 1.20 1.20 1.19 1.19 1.18 0.90 0.89 0.88 0.87 0.86 0.86 0.76 0.75 0.57 0.56
O
N
BzHN
3c
600 MHz, $CDCl_3$
0.97 1.99 3.03 4.12 1.00 0.99 3.07 1.03 2.02 5.05 1.12 1.08 1.09 3.03 3.02
10.5 10.0 9.5 9.0 8.5 8.0 7.5 7.0 6.5 6.0 5.5 5.0 4.5 4.0 3.5 3.0 2.5 2.0 1.5 1.0 0.5 0.0 -0.5

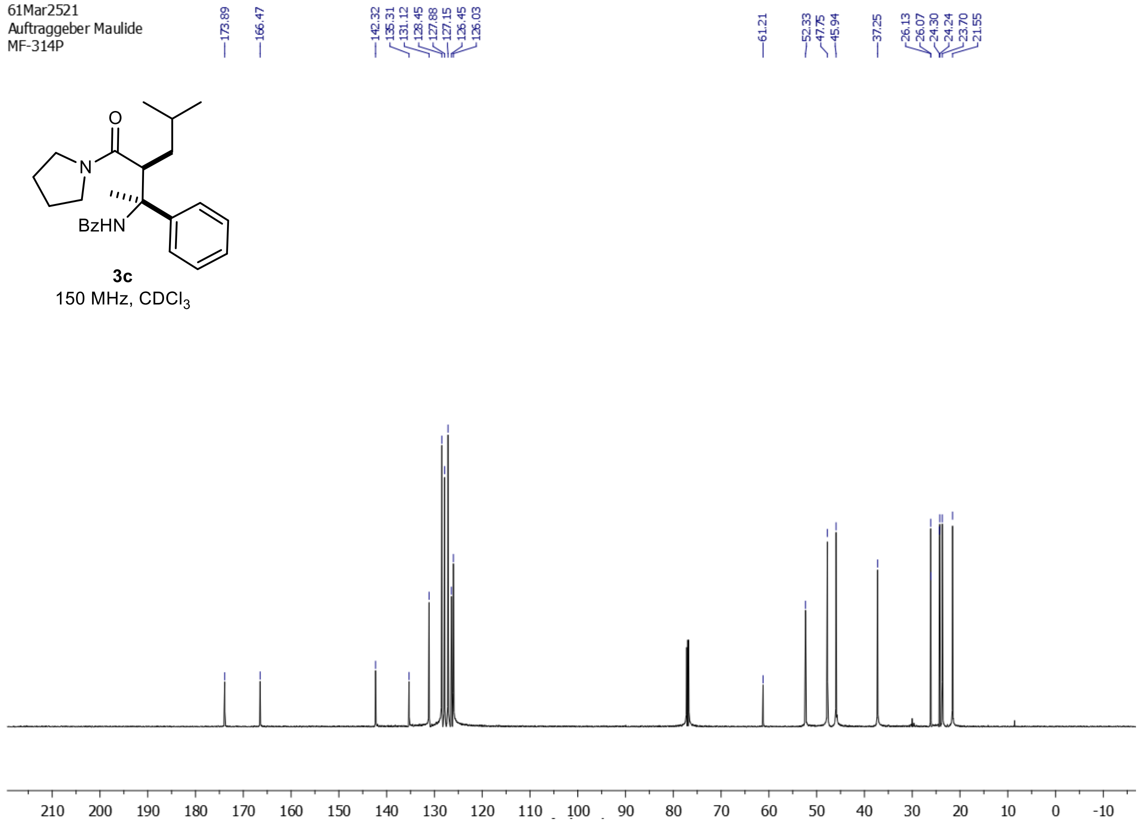
61Mar2521
Auftraggeber Maulide
MF-314P
173.89 166.47 142.32 135.31 131.12 128.45 127.88 127.15 126.45 126.03 61.21 52.33 47.75 45.94 37.25 26.13 26.07 24.30 24.24 23.70 21.55
O
N
BzHN
3c
150 MHz, $CDCl_3$
210 200 190 180 170 160 150 140 130 120 110 100 90 80 70 60 50 40 30 20 10 0 -10

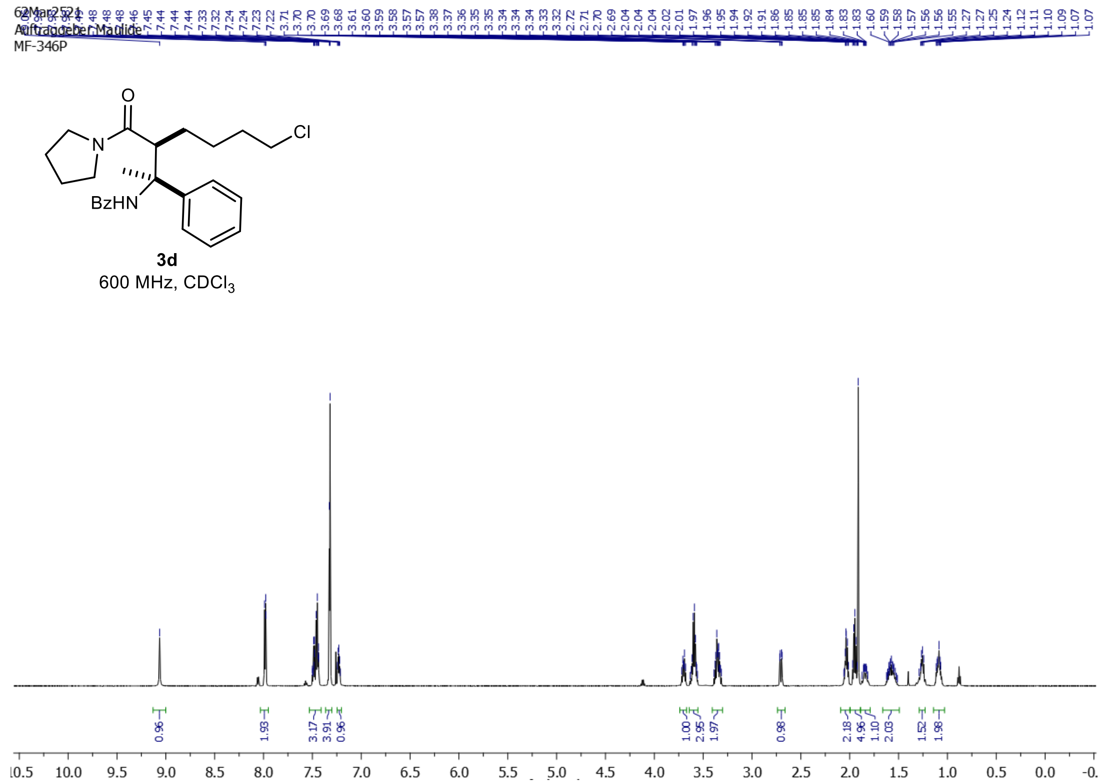

3d
600 MHz, $CDCl_3$

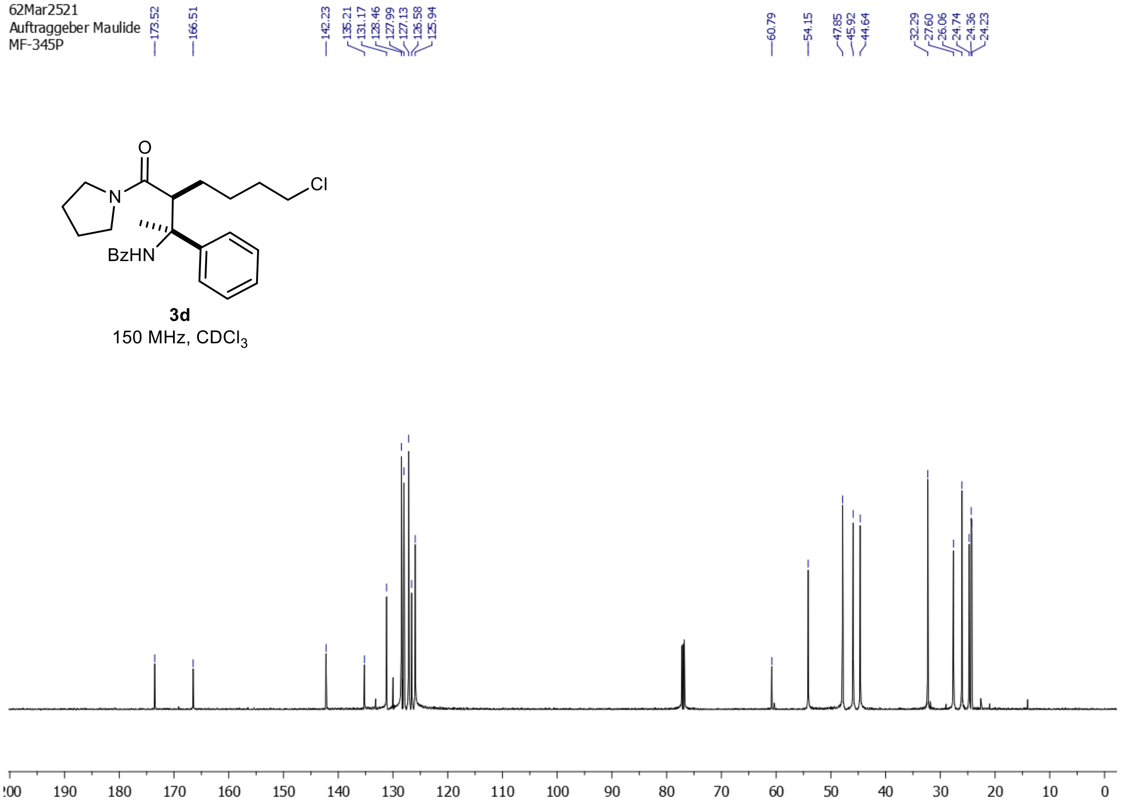

3d
150 MHz, $CDCl_3$

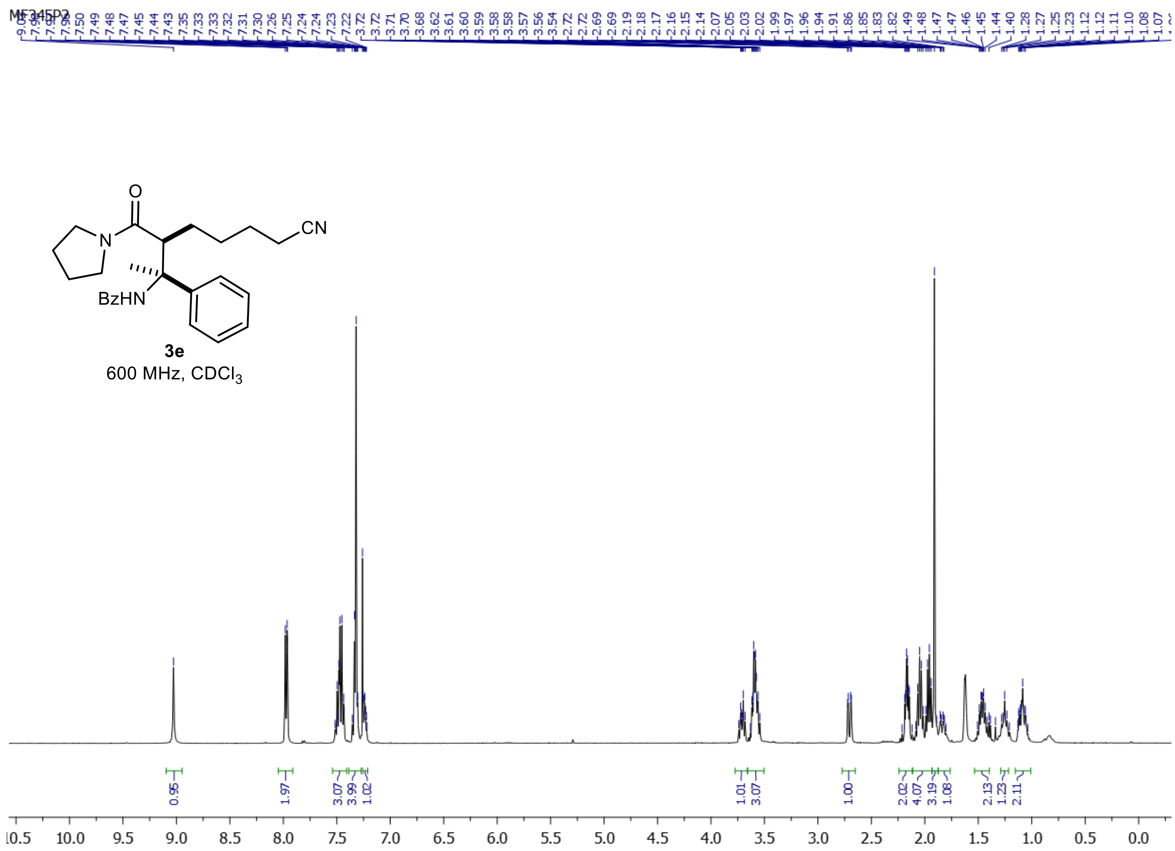
MF345P2
CN
N
O
BzHN
3e
600 MHz, CDCl3
0.95
1.97
3.07
3.99
1.02
1.01
3.07
1.00
2.02
4.07
3.19
1.08
2.13
1.23
2.11

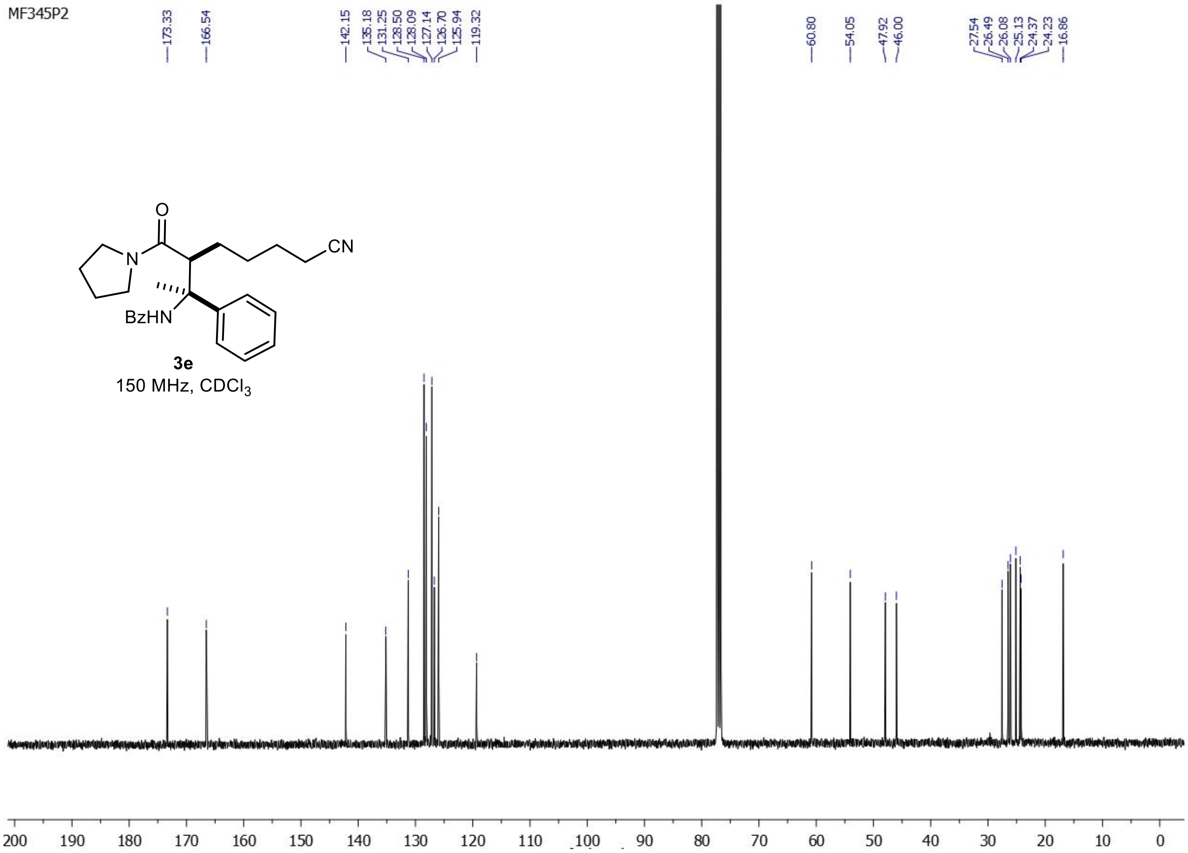
MF345P2
173.33
166.54
142.15
135.18
131.25
128.50
128.09
127.14
126.70
125.94
119.32
60.80
54.05
47.92
46.00
27.54
26.49
26.08
25.13
24.37
24.23
16.86
CN
N
O
BzHN
3e
150 MHz, CDCl3

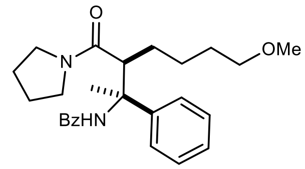

3f
600 MHz, $CDCl_3$

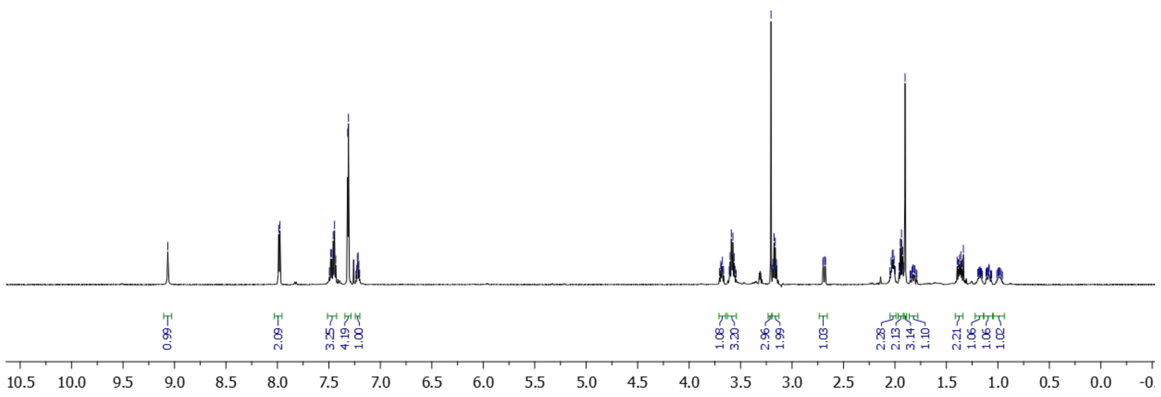

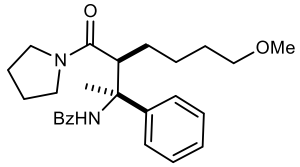

3f
150 MHz, $CDCl_3$

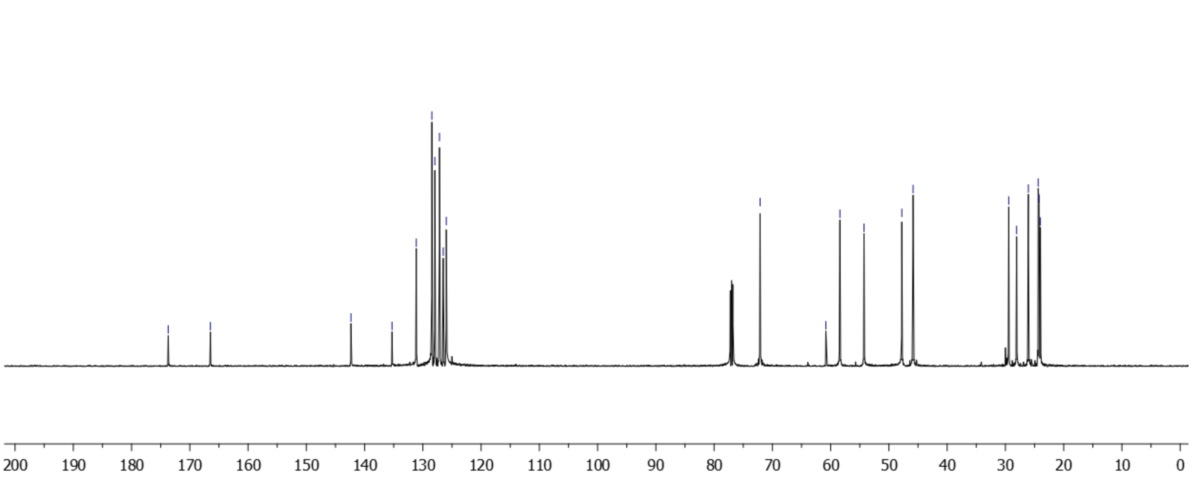

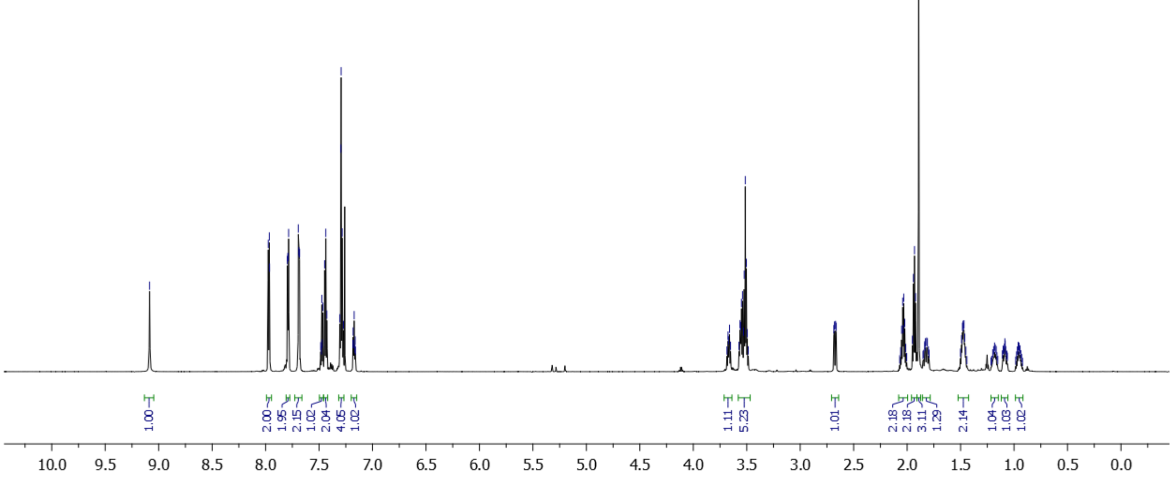

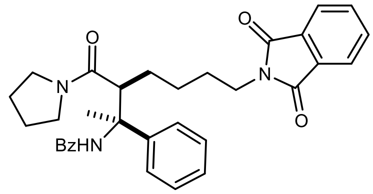

3g
700 MHz, $CDCl_3$

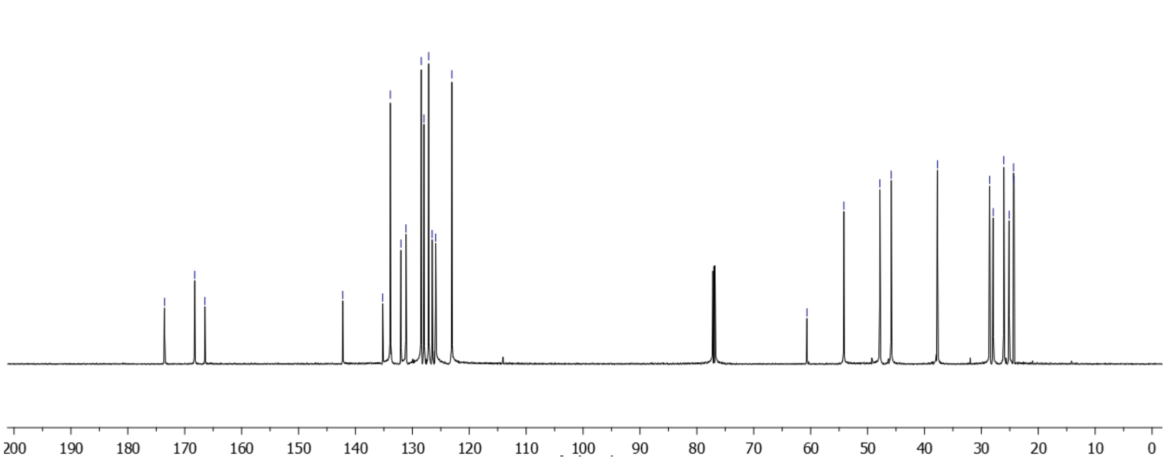

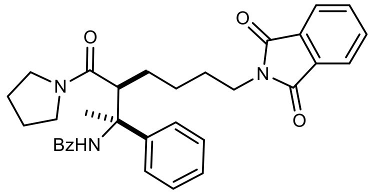

3g
175 MHz, $CDCl_3$

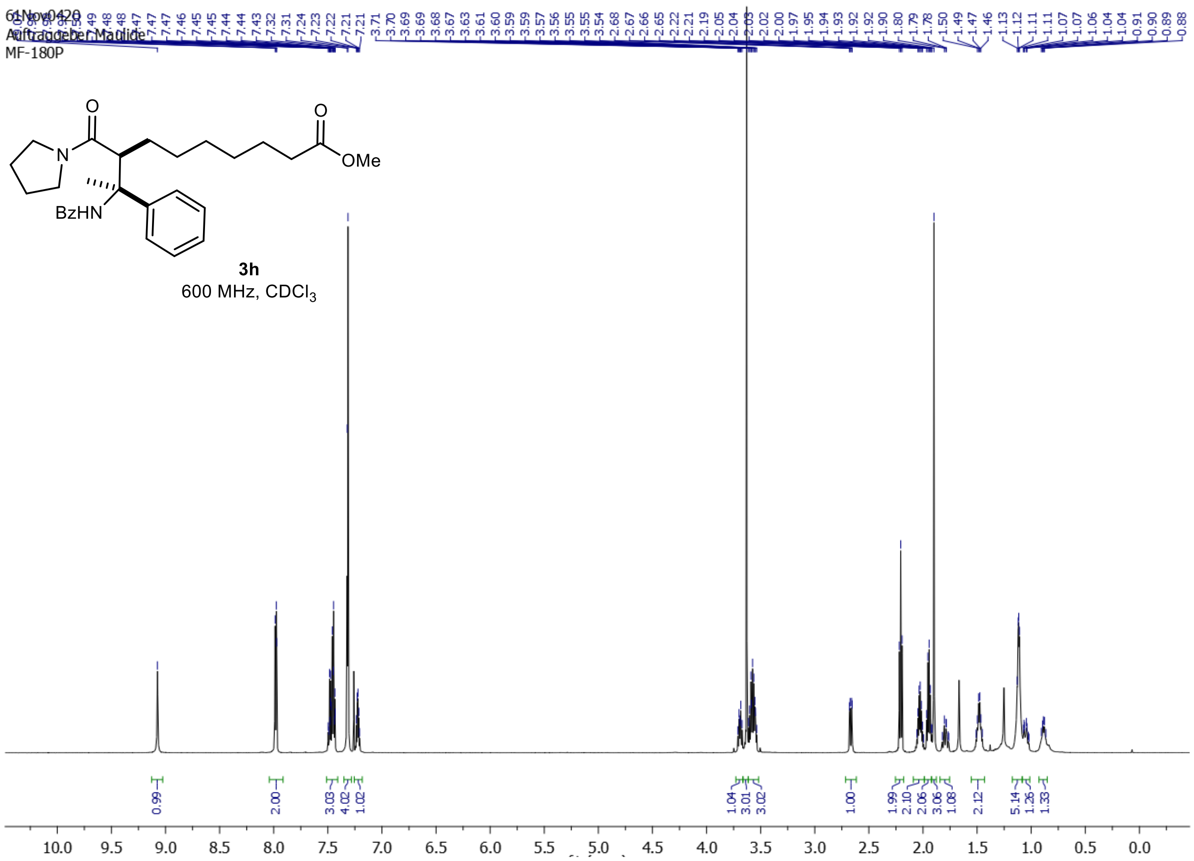

3h
600 MHz, $CDCl_3$

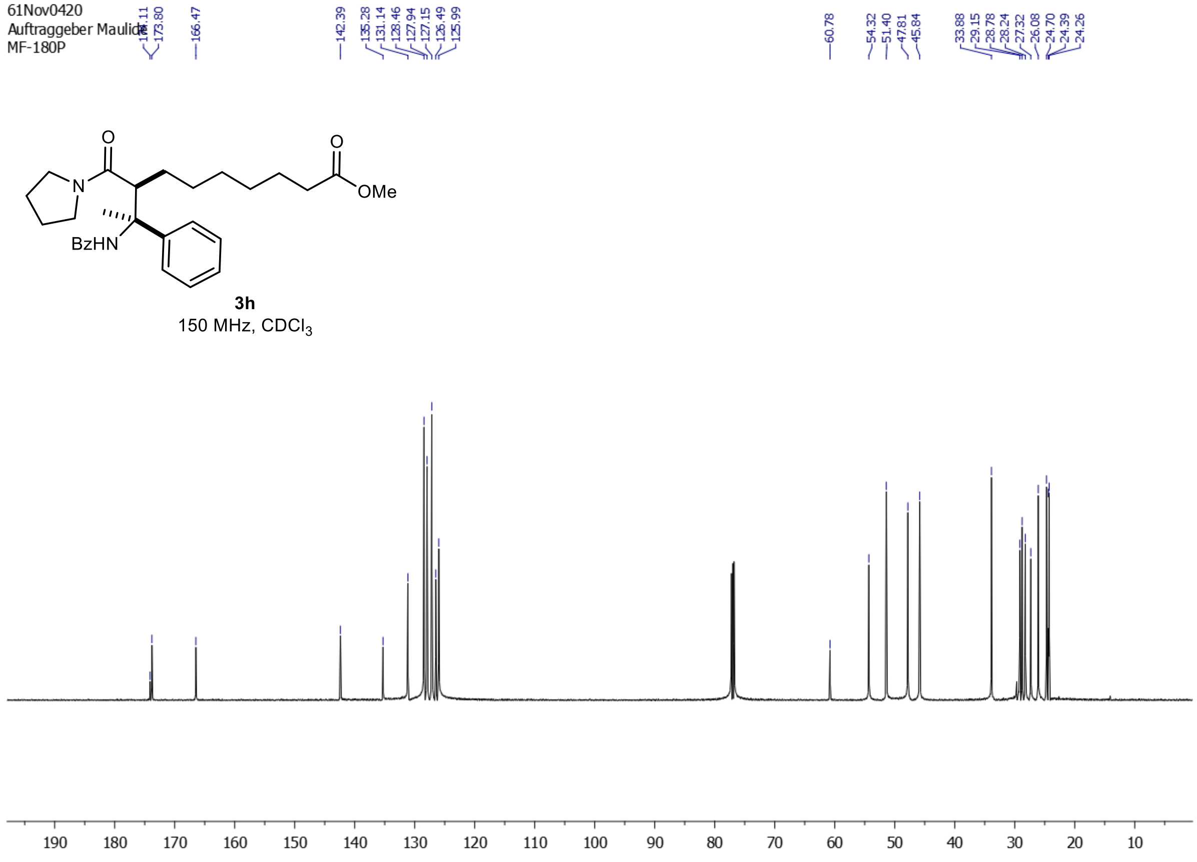

3h
150 MHz, $CDCl_3$

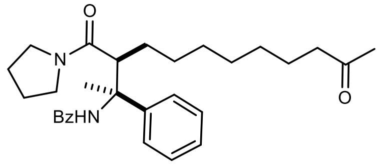

3i
600 MHz, $CDCl_3$

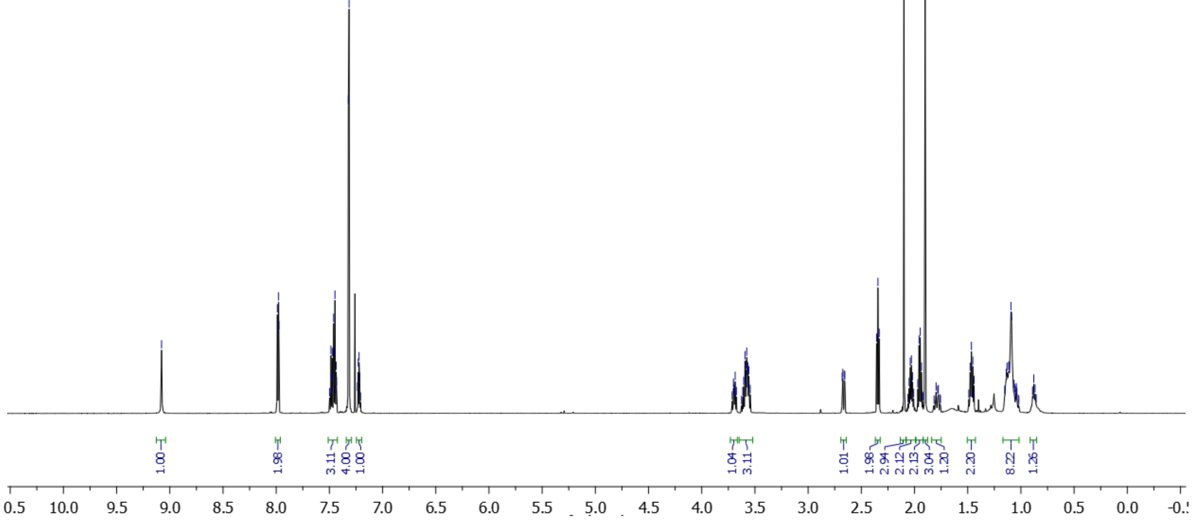

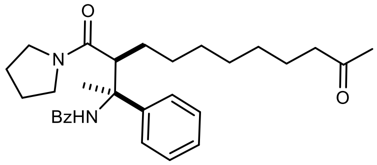

3i
150 MHz, $CDCl_3$

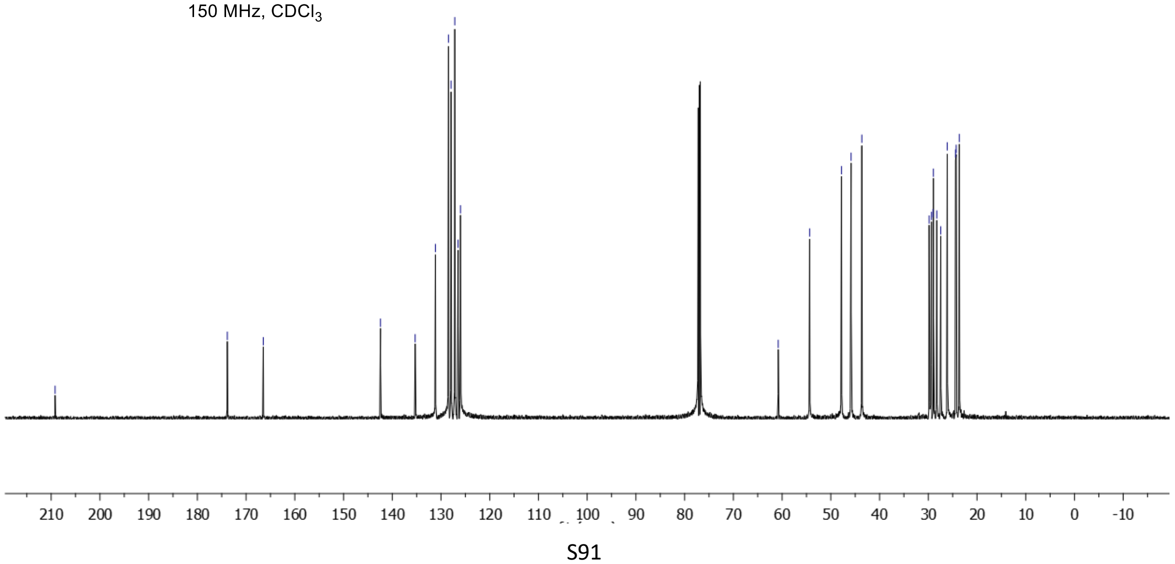

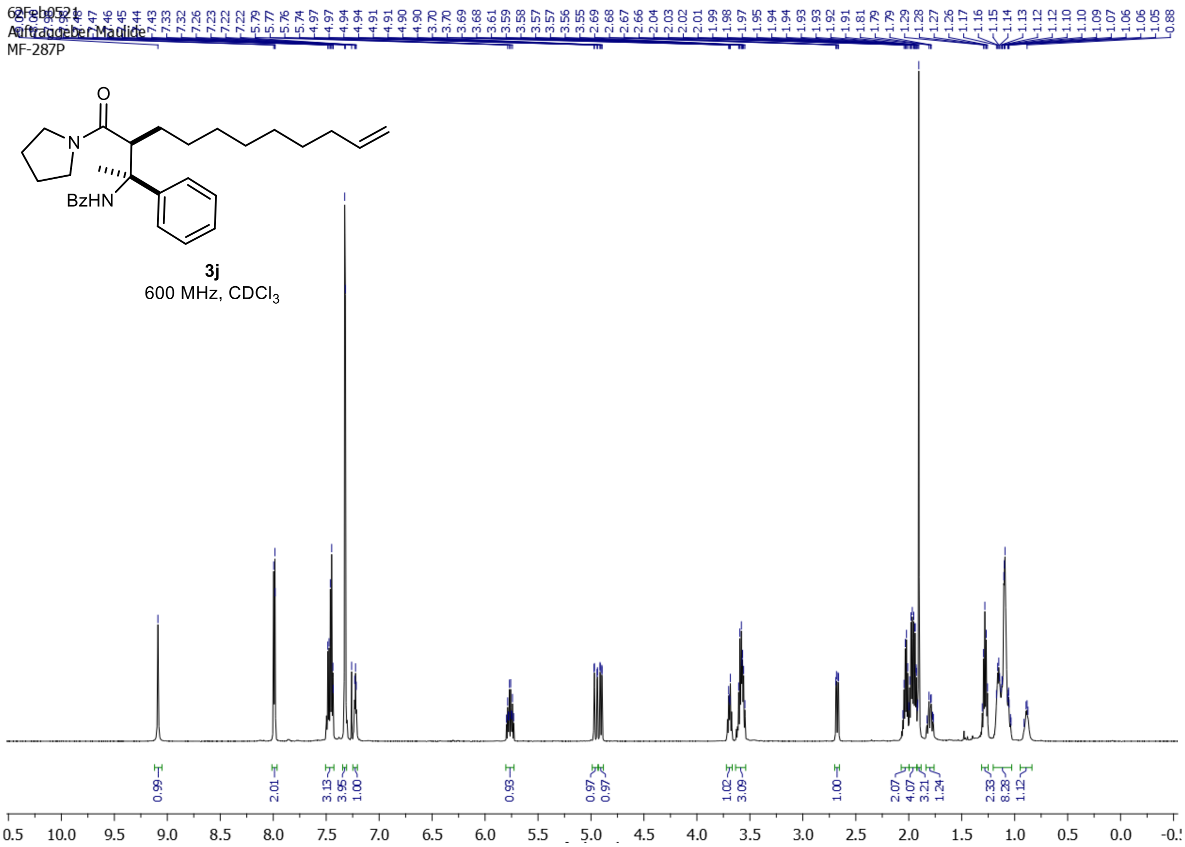
62Feb0521
Auftraggeber Maulide
MF-287P
O
N
BzHN
3j
600 MHz, CDCl3

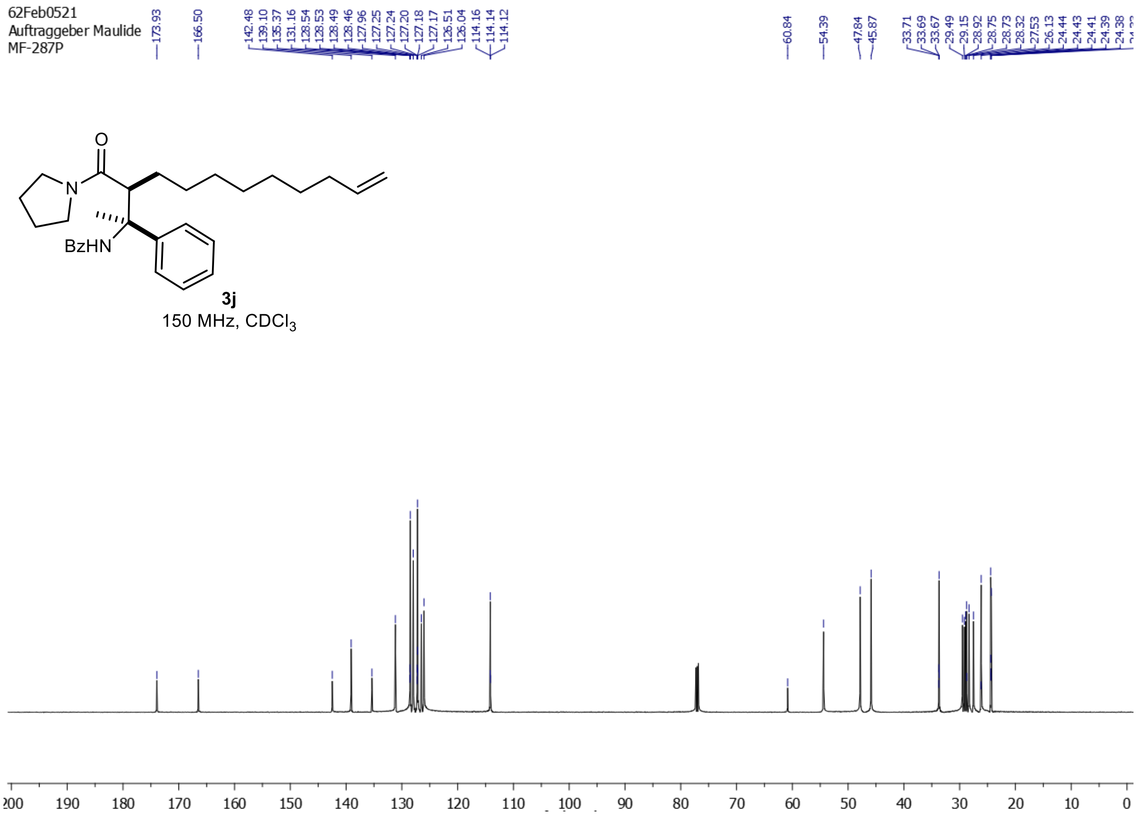
62Feb0521
Auftraggeber Maulide
MF-287P
173.93
166.50
142.48
139.10
135.37
131.16
128.54
128.53
128.49
128.46
127.96
127.25
127.24
127.20
127.18
127.17
126.51
126.04
114.16
114.14
114.12
60.84
54.39
47.84
45.87
33.71
33.69
33.67
29.49
29.15
28.92
28.75
28.73
28.32
27.53
26.13
24.44
24.43
24.41
24.39
24.38
O
N
BzHN
3j
150 MHz, CDCl3

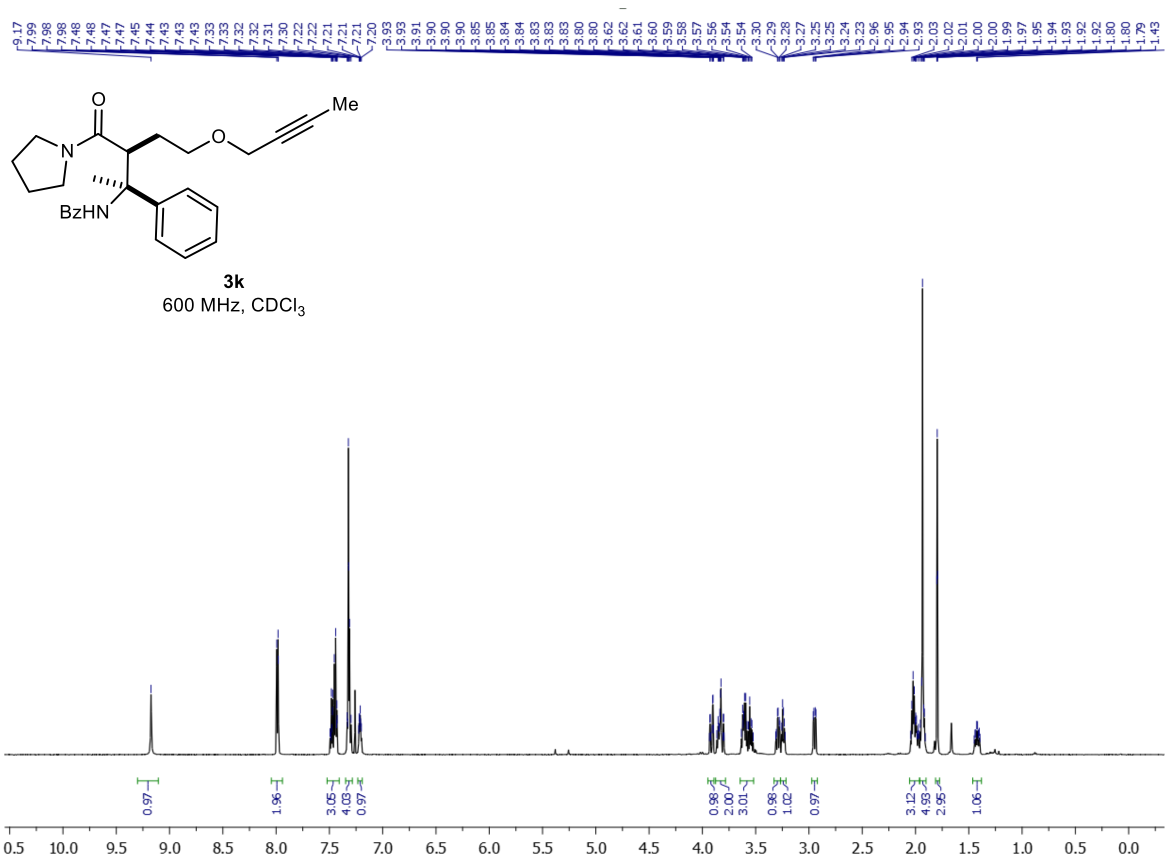

3k
600 MHz, $CDCl_3$

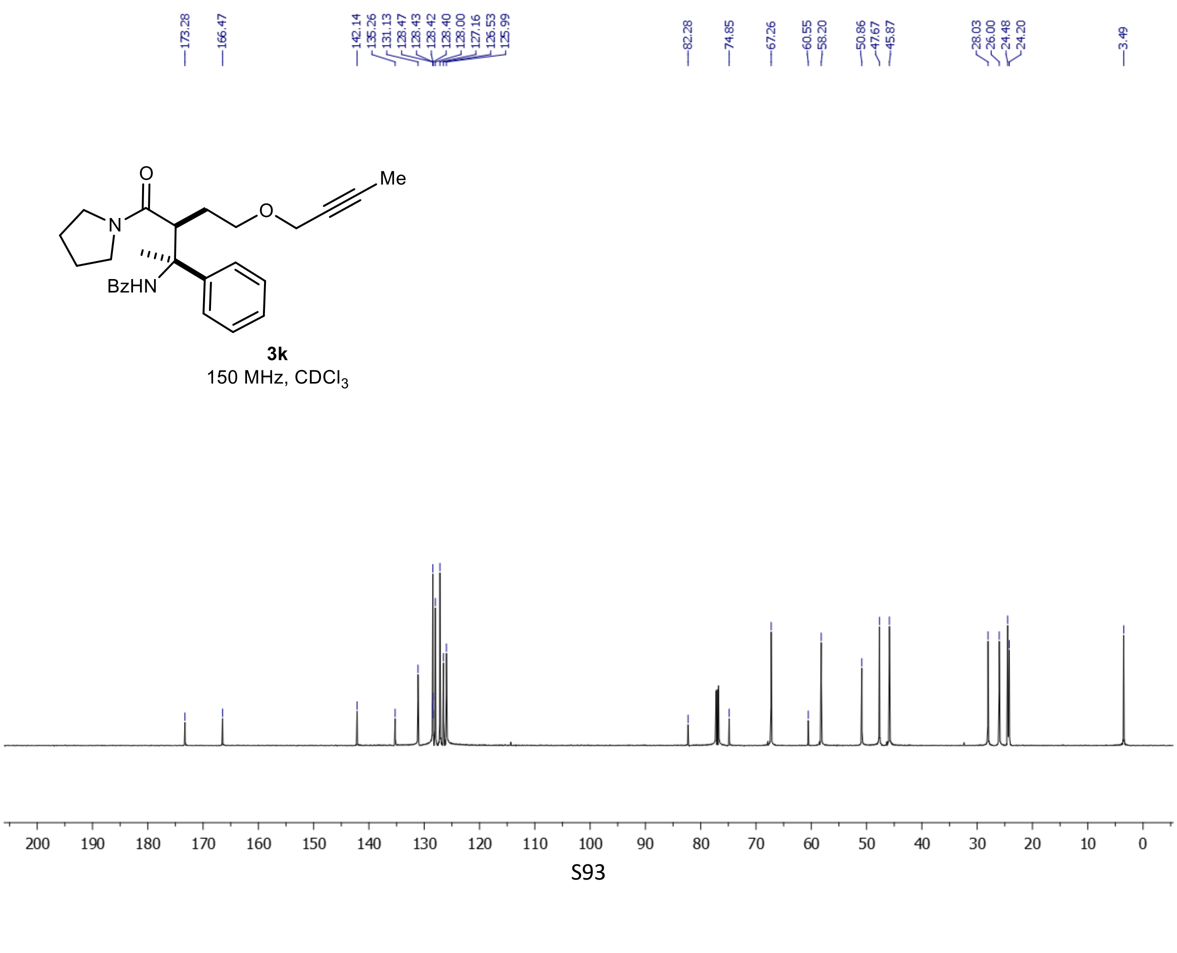

3k
150 MHz, $CDCl_3$

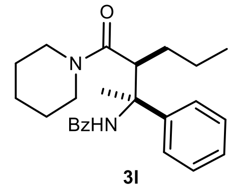

3l
600 MHz, $CDCl_3$

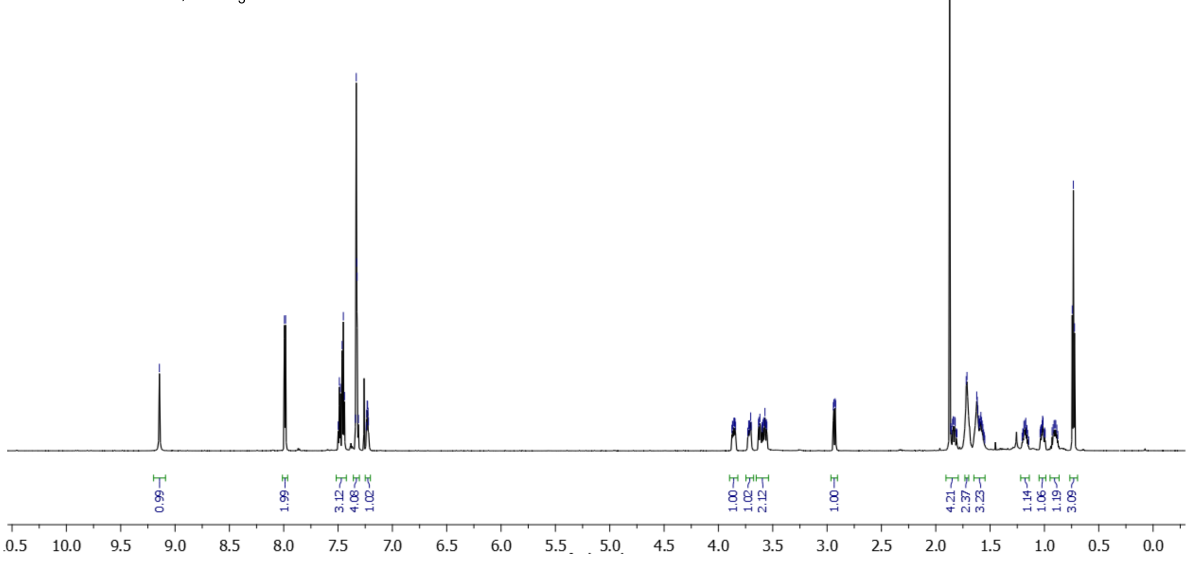

7Feb1821
Auftraggeber Maulide
MF-278P2

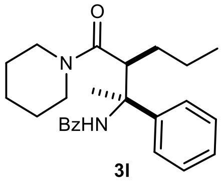

3l
150 MHz, $CDCl_3$

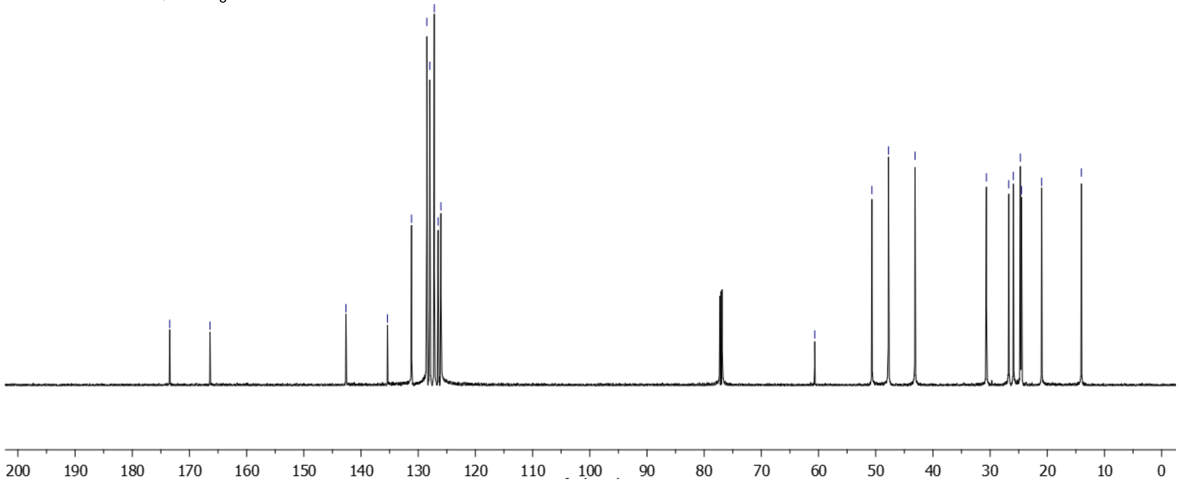

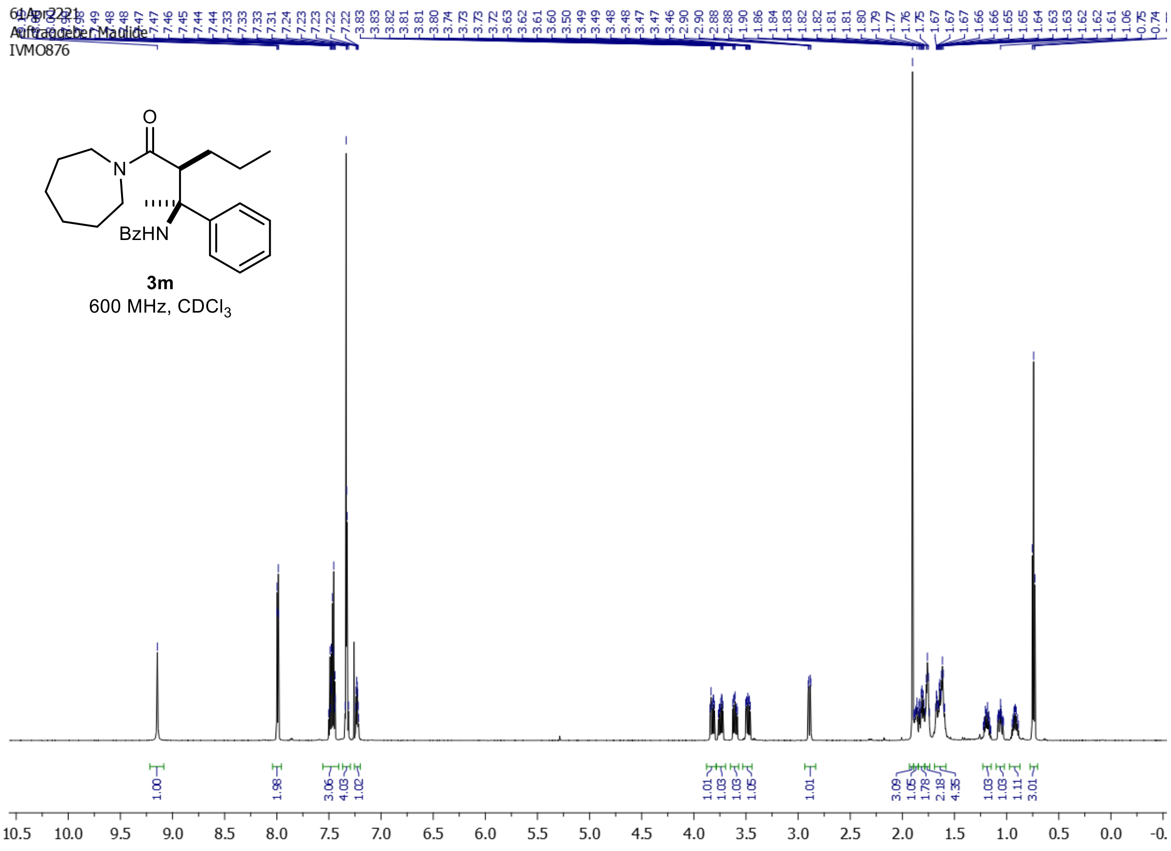
61Apr2221
Auftraggeber Maulide
IVMO876
O
N
BzHN
3m
600 MHz, CDCl3

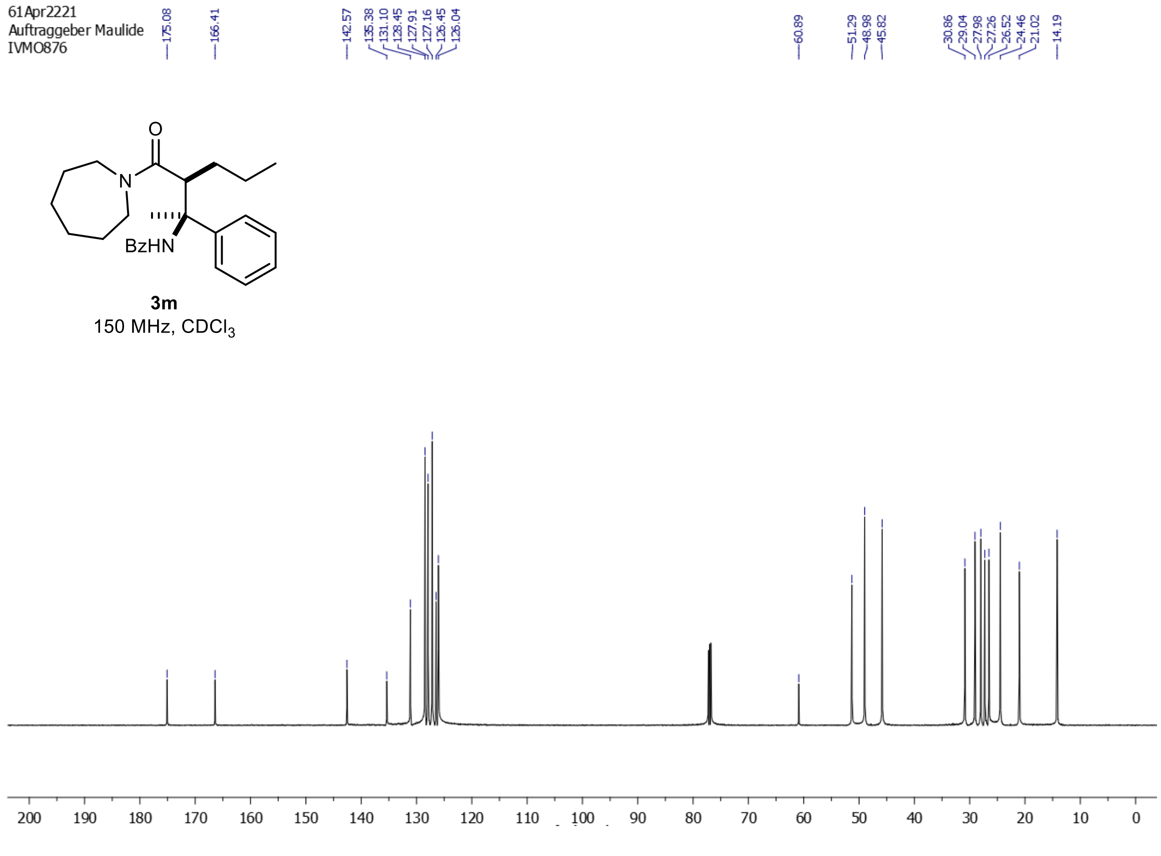
61Apr2221
Auftraggeber Maulide
IVMO876
175.08
166.41
142.57
135.38
131.10
128.45
127.91
127.16
126.45
126.04
60.89
51.29
48.98
45.82
30.86
29.04
27.98
27.26
26.52
24.46
21.02
14.19
O
N
BzHN
3m
150 MHz, CDCl3

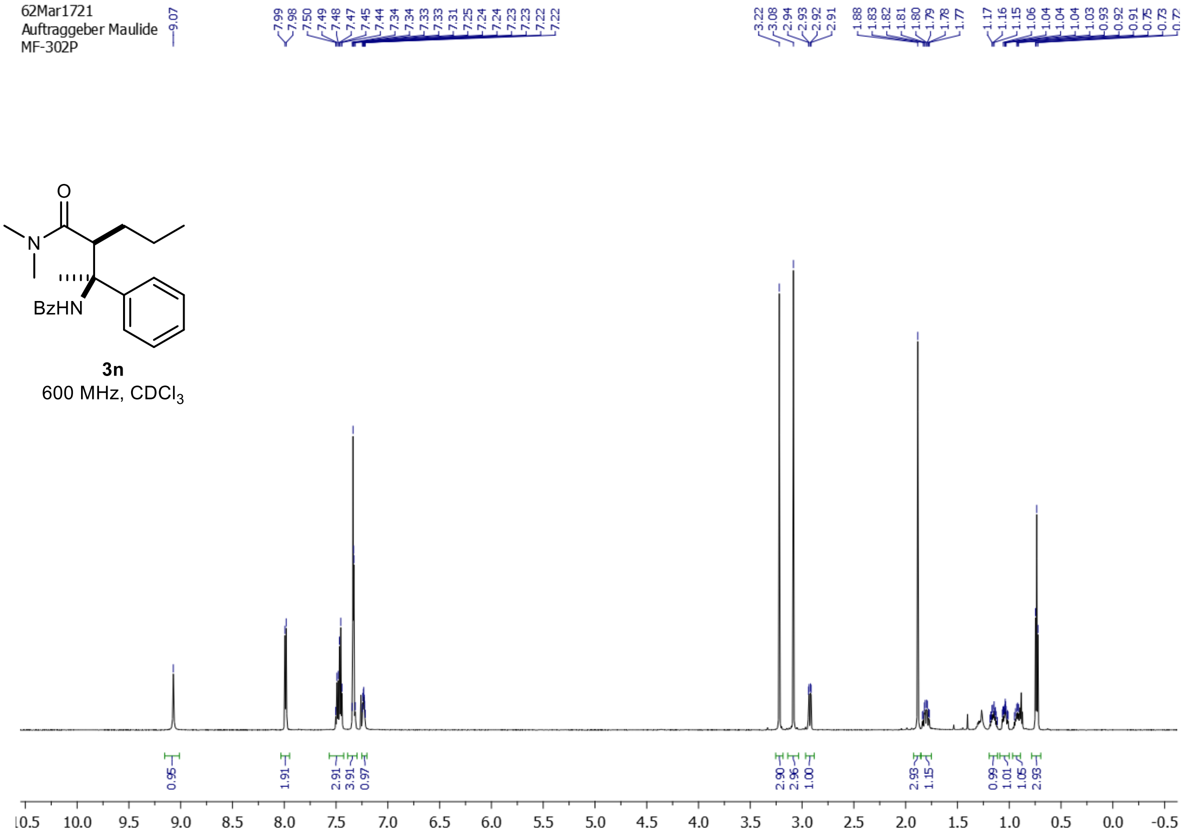

3n
600 MHz, $CDCl_3$

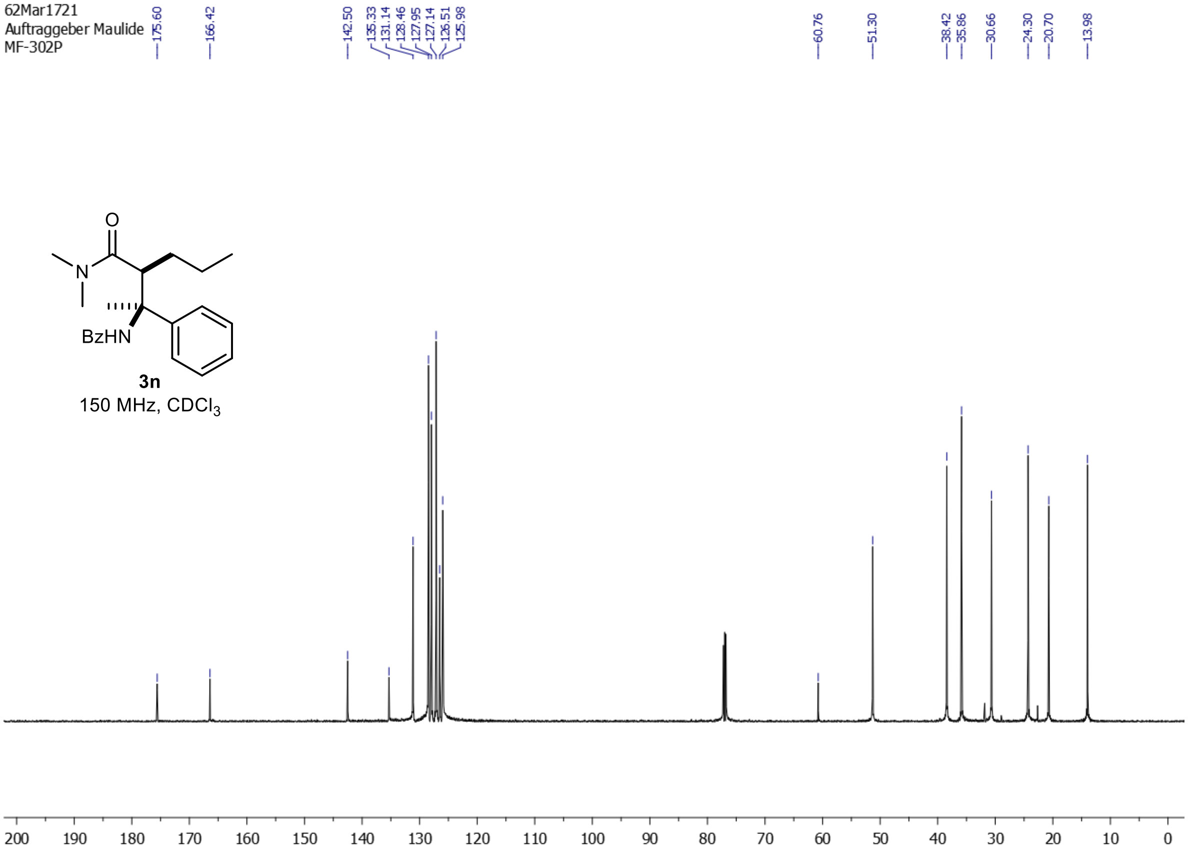

3n
150 MHz, $CDCl_3$

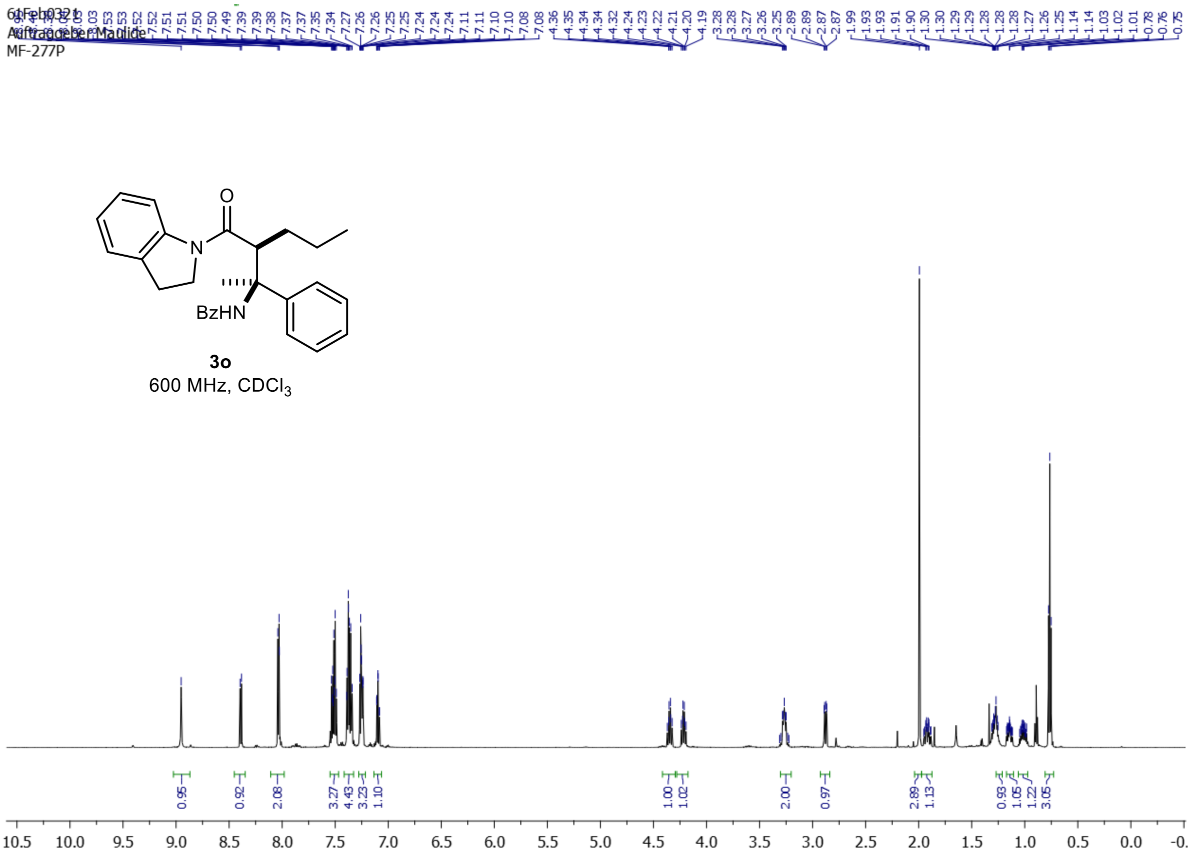
61Feb0321
Auftraggeber Maulide
MF-277P
BzHN
3o
600 MHz, CDCl3
0.95 0.92 2.08 3.27 4.43 3.23 1.10 1.00 1.02 2.00 0.97 2.89 1.13 0.93 1.05 1.22 3.05
10.5 10.0 9.5 9.0 8.5 8.0 7.5 7.0 6.5 6.0 5.5 5.0 4.5 4.0 3.5 3.0 2.5 2.0 1.5 1.0 0.5 0.0

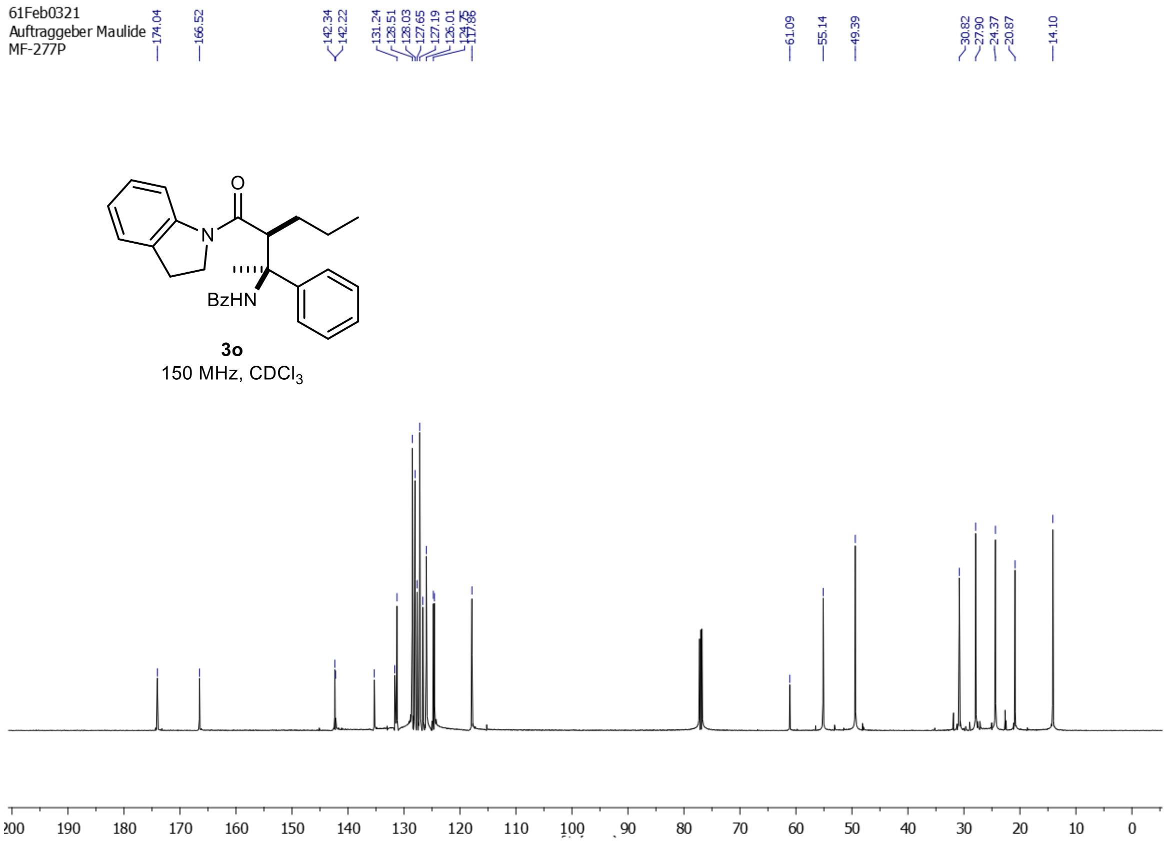
61Feb0321
Auftraggeber Maulide
MF-277P
174.04 166.52 142.34 142.22 131.24 128.51 128.03 127.65 127.19 126.01 117.86 61.09 55.14 49.39 30.82 27.90 24.37 20.87 14.10
BzHN
3o
150 MHz, CDCl3
200 190 180 170 160 150 140 130 120 110 100 90 80 70 60 50 40 30 20 10 0

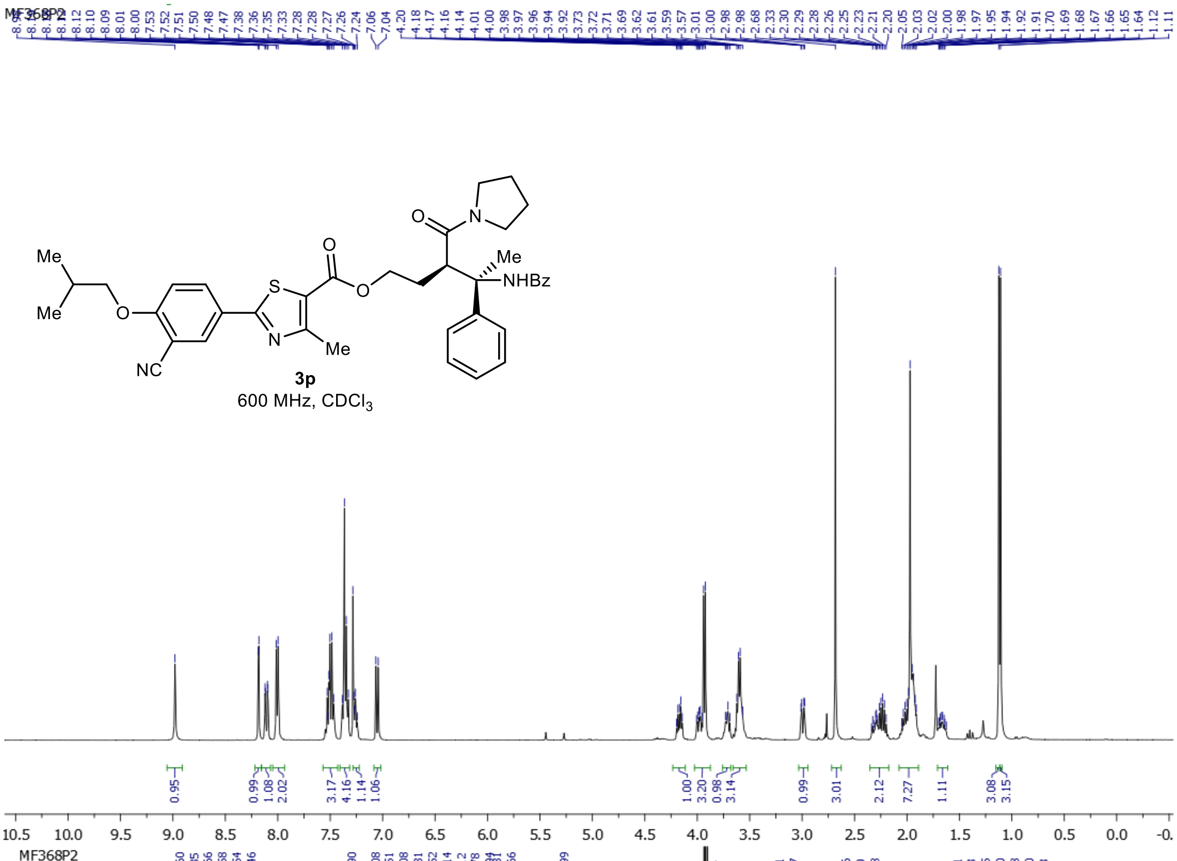
MF368P2
Me
Me
O
NC
S
N
Me
O
O
O
N
Me
NHBz
3p
600 MHz, $CDCl_3$
0.95
0.99
1.08
2.02
3.17
4.16
1.14
1.06
1.00
3.20
0.98
3.14
0.99
3.01
2.12
7.27
1.11
3.08
3.15
10.5 10.0 9.5 9.0 8.5 8.0 7.5 7.0 6.5 6.0 5.5 5.0 4.5 4.0 3.5 3.0 2.5 2.0 1.5 1.0 0.5 0.0

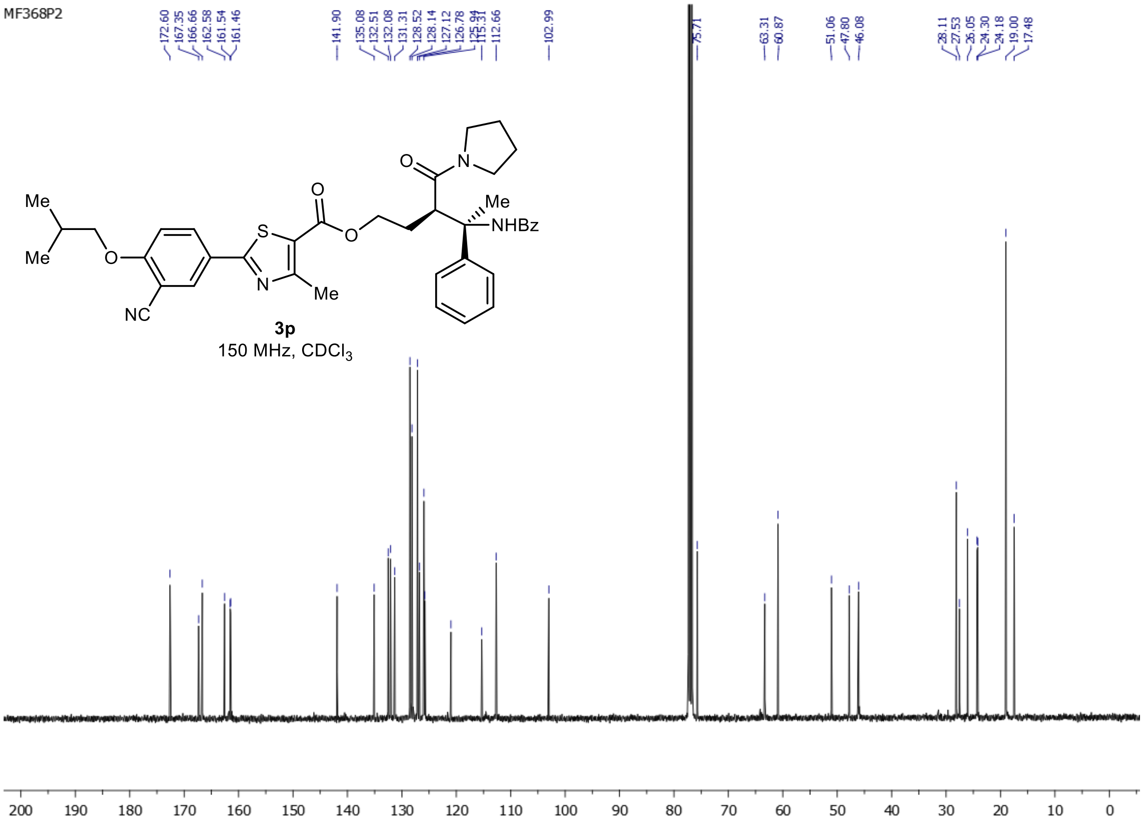
MF368P2
172.60
167.35
166.66
162.58
161.54
161.46
141.90
135.08
132.51
132.08
131.31
128.52
128.14
127.12
126.78
112.66
102.99
75.71
63.31
60.87
51.06
47.80
46.08
28.11
27.53
26.05
24.30
24.18
19.00
17.48
Me
Me
O
NC
S
N
Me
O
O
O
N
Me
NHBz
3p
150 MHz, $CDCl_3$
200 190 180 170 160 150 140 130 120 110 100 90 80 70 60 50 40 30 20 10 0

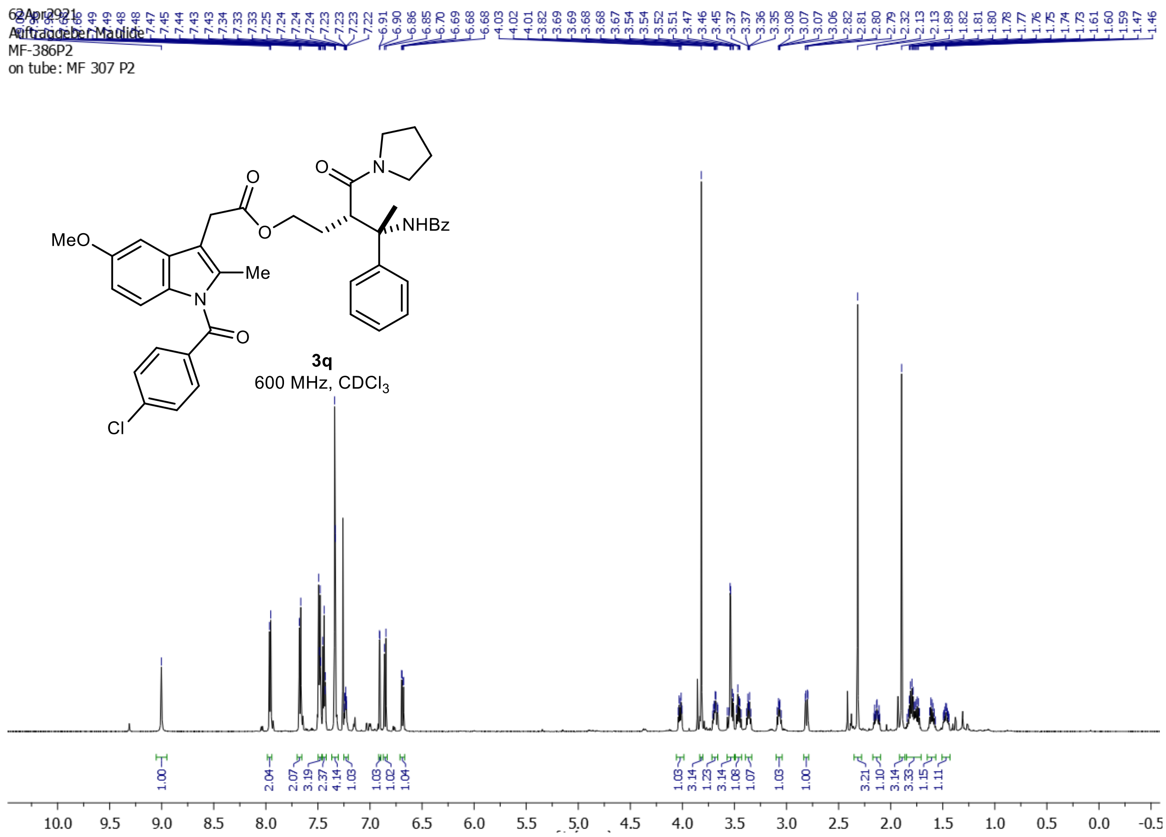
62Apr2921
Auftraggeber Maulide
MF-386P2
on tube: MF 307 P2
7.49 7.48 7.48 7.47 7.45 7.44 7.43 7.43 7.34 7.33 7.33 7.25 7.24 7.24 7.24 7.23 7.23 7.23 7.22 6.91 6.90 6.86 6.85 6.70 6.69 6.68 4.03 4.02 4.01 3.82 3.69 3.69 3.68 3.68 3.67 3.54 3.54 3.52 3.51 3.47 3.46 3.45 3.37 3.37 3.36 3.35 3.08 3.07 3.06 2.82 2.81 2.80 2.79 2.32 2.13 2.13 1.89 1.82 1.81 1.80 1.78 1.77 1.76 1.75 1.74 1.73 1.61 1.60 1.59 1.47 1.46
MeO
Me
N
O
Cl
NHBz
3q
600 MHz, $CDCl_3$
1.00 2.04 2.07 3.19 2.37 4.14 1.03 1.03 1.02 1.04 1.03 3.14 1.23 3.14 1.08 1.07 1.03 1.00 3.21 1.10 3.14 3.33 1.15 1.11
10.0 9.5 9.0 8.5 8.0 7.5 7.0 6.5 6.0 5.5 5.0 4.5 4.0 3.5 3.0 2.5 2.0 1.5 1.0 0.5 0.0 -0.5

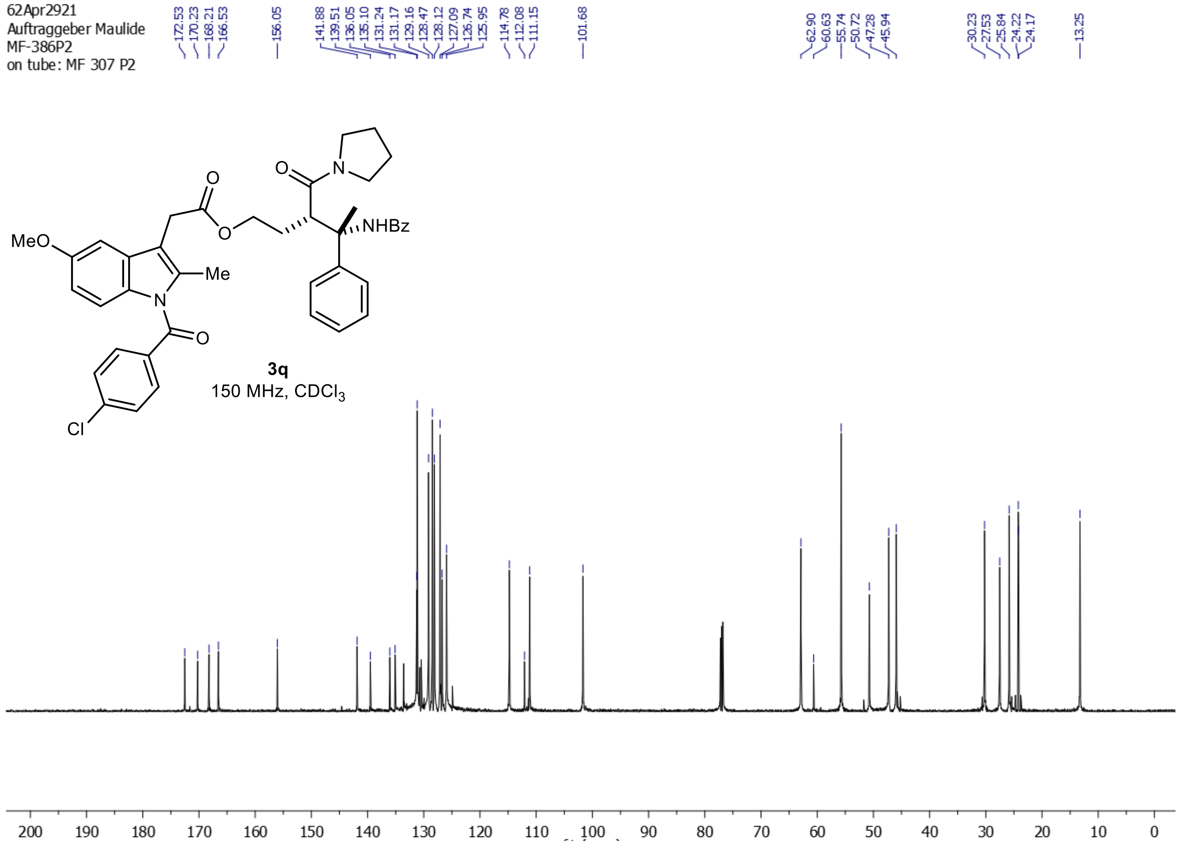
62Apr2921
Auftraggeber Maulide
MF-386P2
on tube: MF 307 P2
172.53 170.23 168.21 166.53 156.05 141.88 139.51 136.05 135.10 131.24 131.17 129.16 128.47 128.12 127.09 126.74 125.95 114.78 112.08 111.15 101.68 62.90 60.63 55.74 50.72 47.28 45.94 30.23 27.53 25.84 24.22 24.17 13.25
MeO
Me
N
O
Cl
NHBz
3q
150 MHz, $CDCl_3$
200 190 180 170 160 150 140 130 120 110 100 90 80 70 60 50 40 30 20 10 0

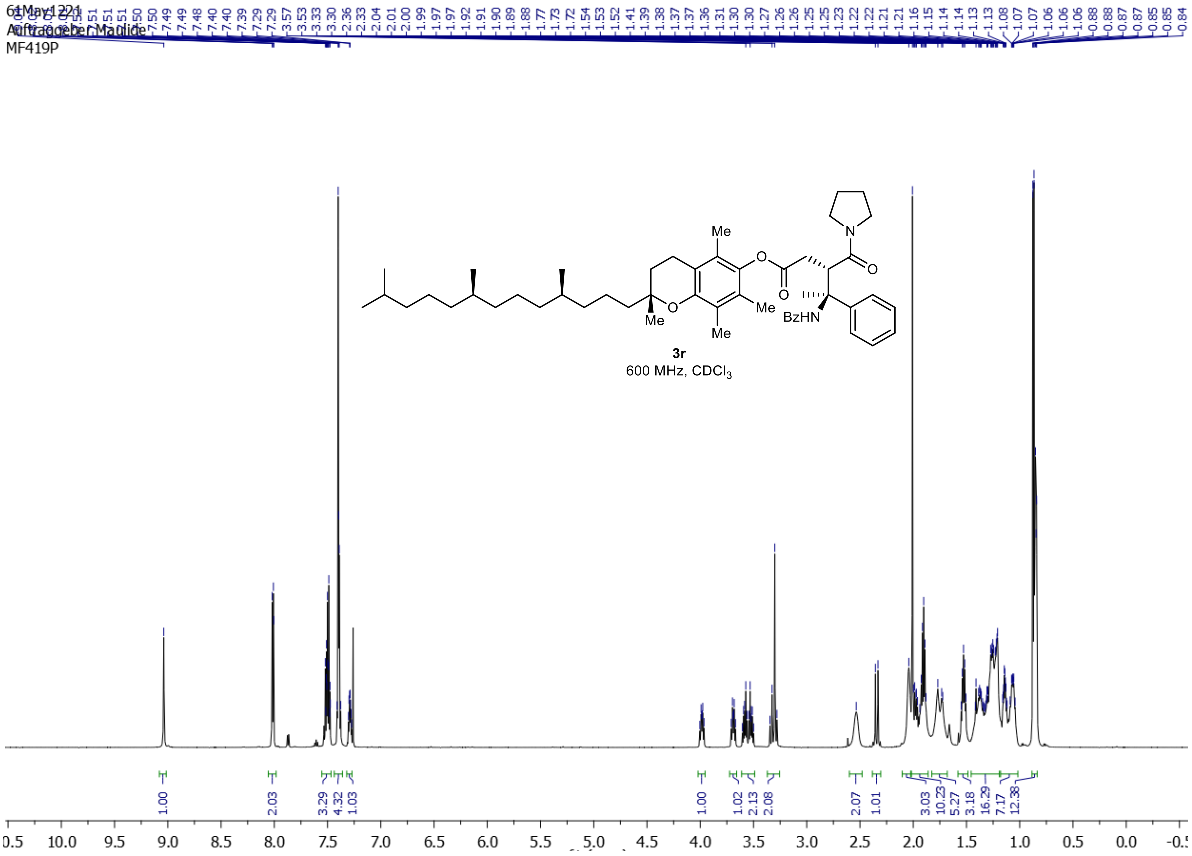
61May1221
Auftraggeber Maulide
MF419P
7.50 7.49 7.48 7.40 7.40 7.39 7.29 7.29 3.57 3.53 3.33 3.30 2.36 2.33 2.04 2.01 2.00 1.99 1.97 1.97 1.92 1.91 1.90 1.89 1.88 1.77 1.73 1.72 1.54 1.53 1.52 1.41 1.39 1.38 1.37 1.37 1.36 1.31 1.30 1.30 1.27 1.26 1.26 1.25 1.25 1.23 1.22 1.22 1.21 1.21 1.16 1.15 1.14 1.14 1.13 1.13 1.08 1.07 1.07 1.06 1.06 0.88 0.88 0.87 0.87 0.85 0.84
Me
O
N
O
O
Me
Me
BzHN
Me
3r
600 MHz, CDCl3
1.00 2.03 3.29 4.32 1.03 1.00 1.02 2.13 2.08 2.07 1.01 3.03 10.23 5.27 3.18 16.29 7.17 12.38
10.0 9.5 9.0 8.5 8.0 7.5 7.0 6.5 6.0 5.5 5.0 4.5 4.0 3.5 3.0 2.5 2.0 1.5 1.0 0.5 0.0

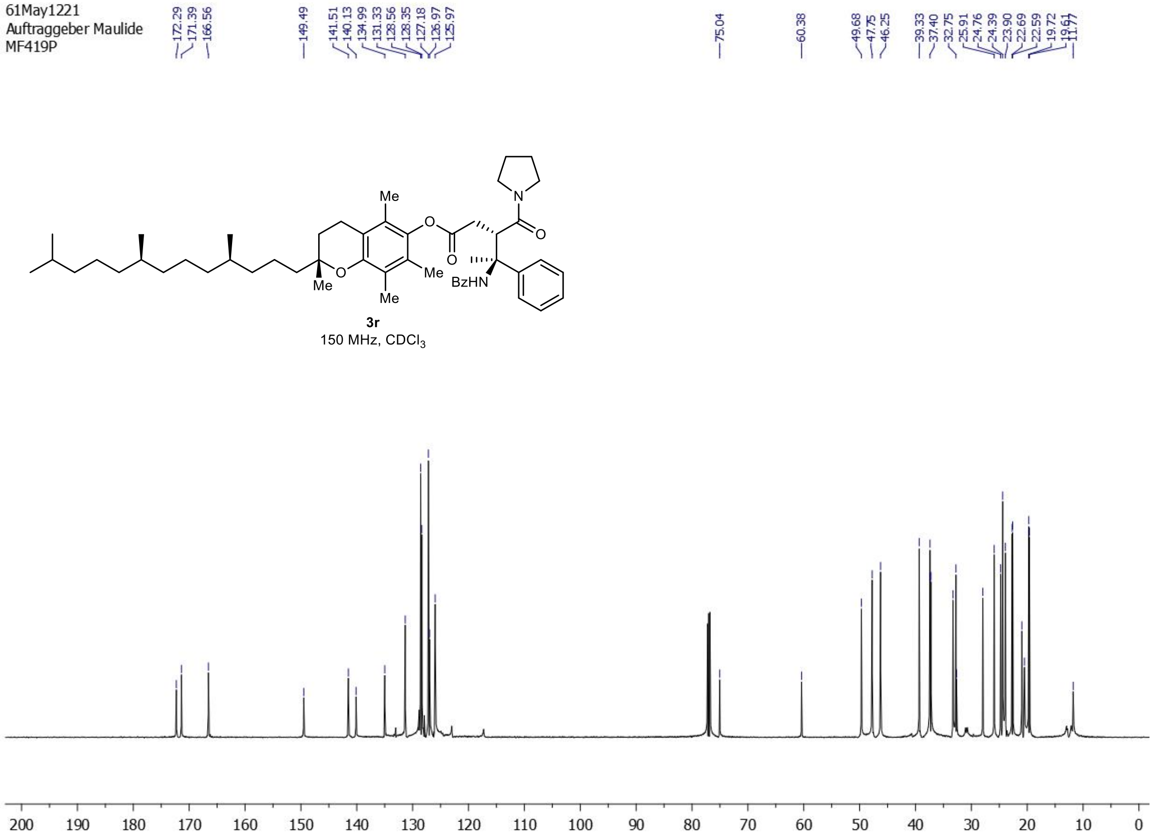
61May1221
Auftraggeber Maulide
MF419P
172.29 171.39 166.56 149.49 141.51 140.13 134.99 131.33 128.56 128.35 127.18 126.97 125.97 75.04 60.38 49.68 47.75 46.25 39.33 37.40 32.75 25.91 24.76 24.39 23.90 22.69 22.59 19.72 19.61 11.77
Me
O
N
O
O
Me
Me
BzHN
Me
3r
150 MHz, CDCl3
200 190 180 170 160 150 140 130 120 110 100 90 80 70 60 50 40 30 20 10 0

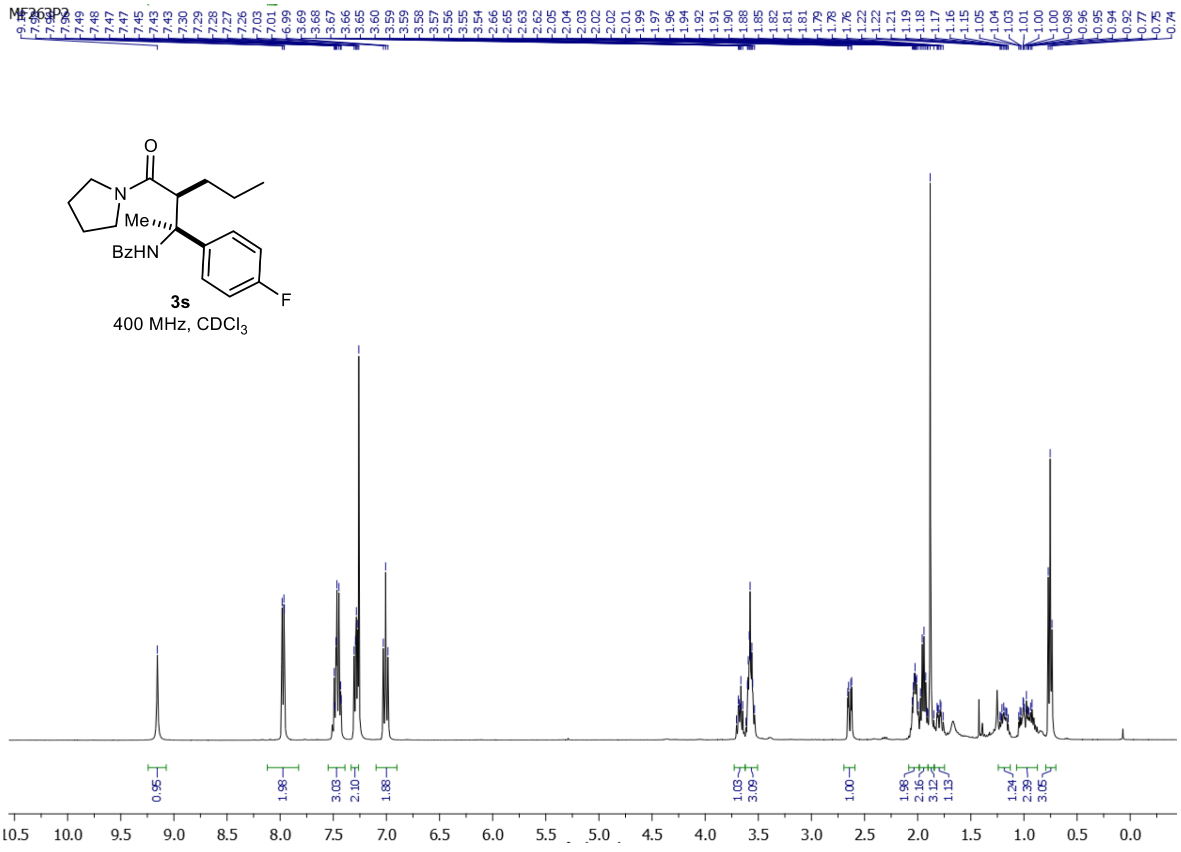
MF263P2
7.49 7.48 7.47 7.45 7.43 7.43 7.30 7.29 7.28 7.27 7.26 7.03 7.01 6.99 3.69 3.68 3.67 3.66 3.65 3.60 3.59 3.59 3.58 3.57 3.56 3.55 3.54 2.66 2.65 2.63 2.62 2.05 2.04 2.03 2.02 2.01 1.99 1.97 1.96 1.94 1.92 1.91 1.90 1.88 1.85 1.82 1.81 1.81 1.79 1.78 1.76 1.22 1.22 1.21 1.19 1.18 1.17 1.16 1.15 1.05 1.04 1.03 1.01 1.00 0.98 0.96 0.95 0.94 0.92 0.77 0.75 0.74
O
N
Me
BzHN
F
3s
400 MHz, CDCl$_3$
0.95
1.98
3.03
2.10
1.88
1.03
3.09
1.00
1.98
2.16
3.12
1.13
1.24
2.39
3.05
10.5 10.0 9.5 9.0 8.5 8.0 7.5 7.0 6.5 6.0 5.5 5.0 4.5 4.0 3.5 3.0 2.5 2.0 1.5 1.0 0.5 0.0

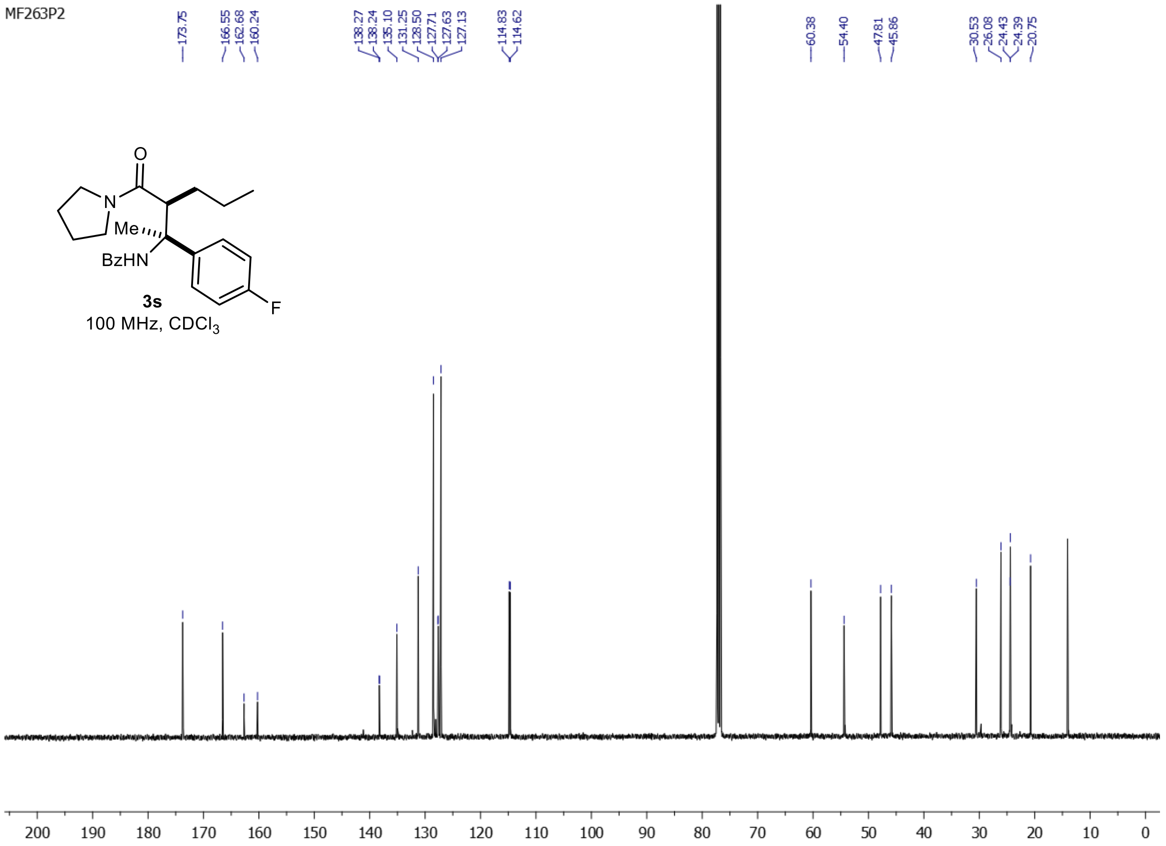
MF263P2
173.75
166.55
162.68
160.24
138.27
138.24
135.10
131.25
128.50
127.71
127.63
127.13
114.83
114.62
60.38
54.40
47.81
45.86
30.53
26.08
24.43
24.39
20.75
O
N
Me
BzHN
F
3s
100 MHz, CDCl$_3$
200 190 180 170 160 150 140 130 120 110 100 90 80 70 60 50 40 30 20 10 0

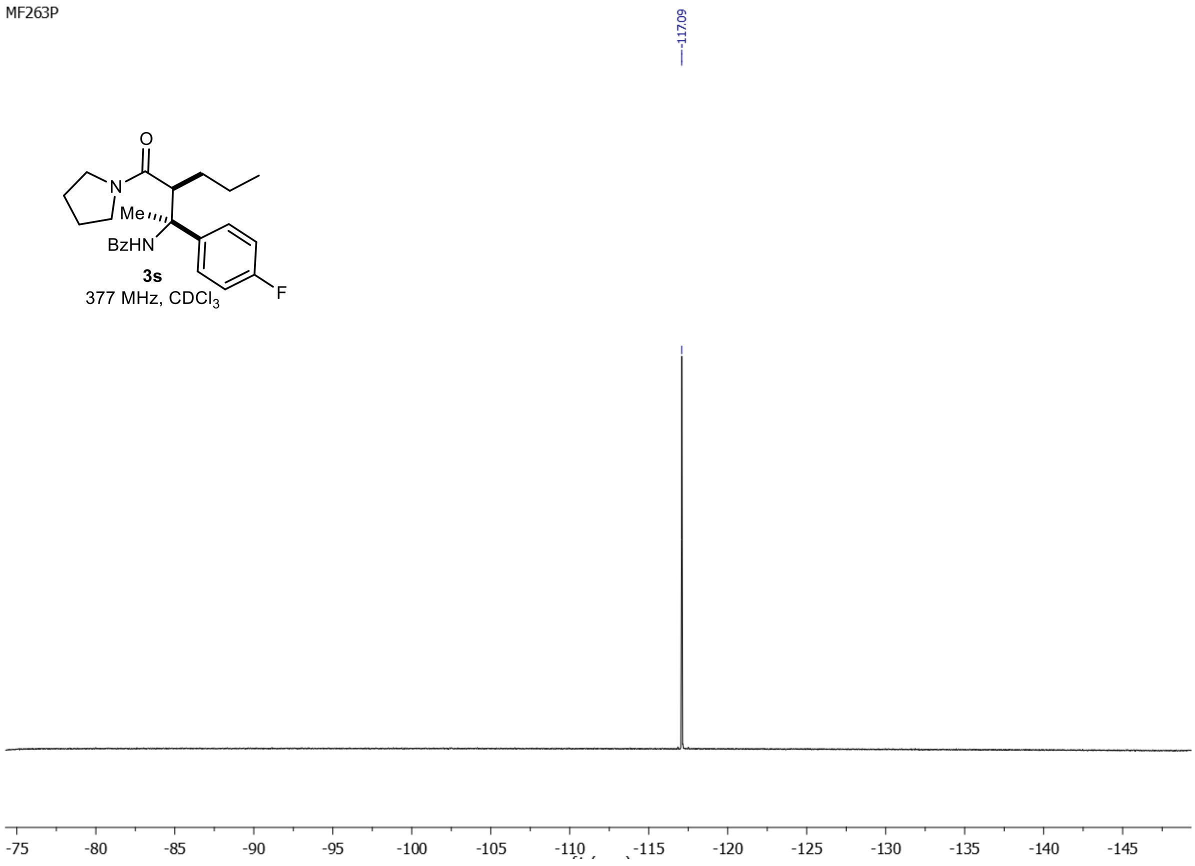
MF263P
-117.09
O
N
Me
BzHN
F
3s
377 MHz, CDCl3
-75
-80
-85
-90
-95
-100
-105
-110
-115
-120
-125
-130
-135
-140
-145

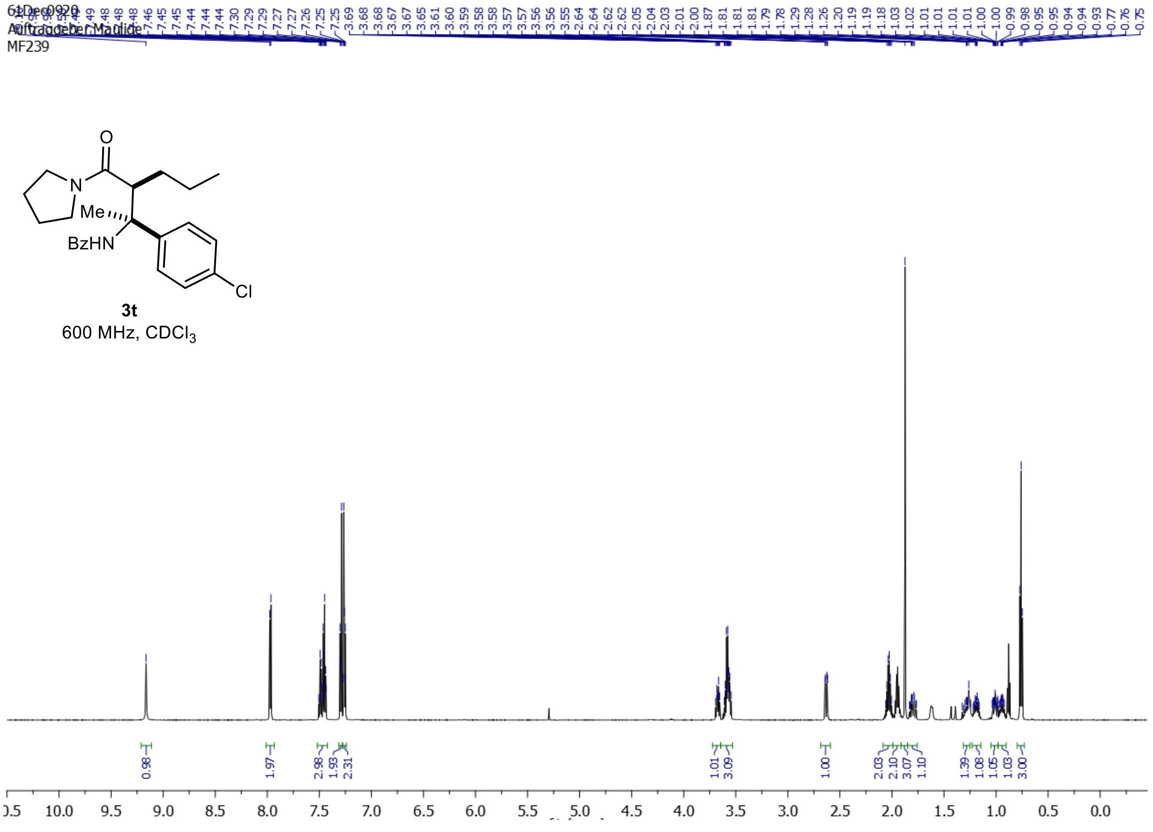
61Dec0920
Auftraggeber Maulide
MF239
3t
600 MHz, CDCl3

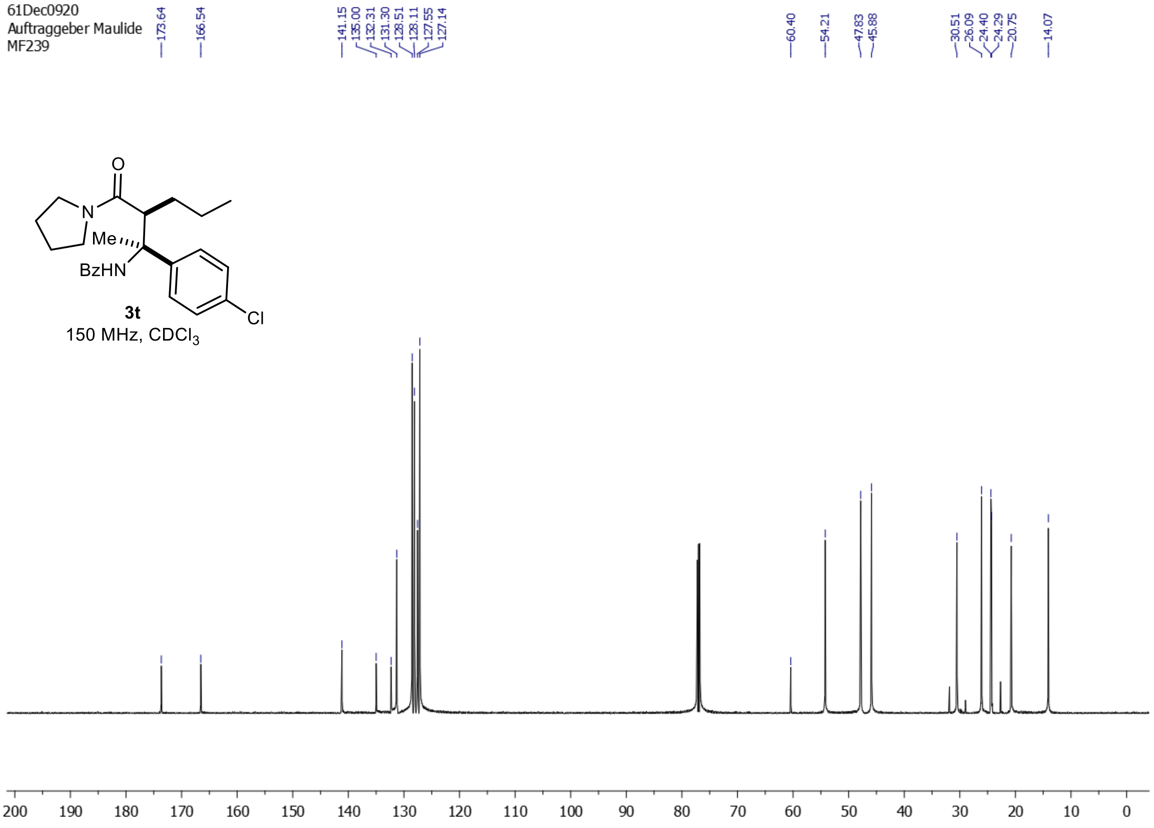
61Dec0920
Auftraggeber Maulide
MF239
3t
150 MHz, CDCl3

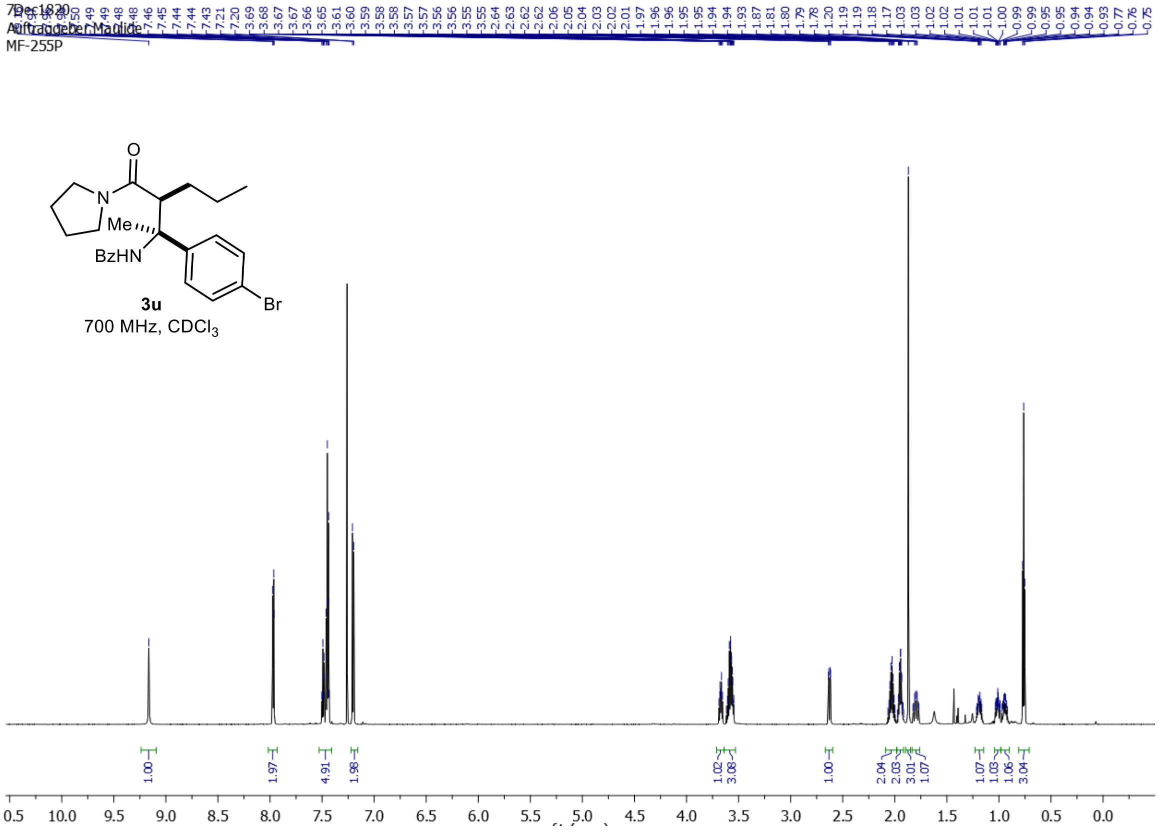
7Dec1820
Auftraggeber Maulide
MF-255P
O
N
Me
BzHN
Br
3u
700 MHz, CDCl3
1.00
1.97
4.91
1.98
1.02
3.08
1.00
2.04
2.03
3.01
1.07
1.07
1.03
1.06
3.04

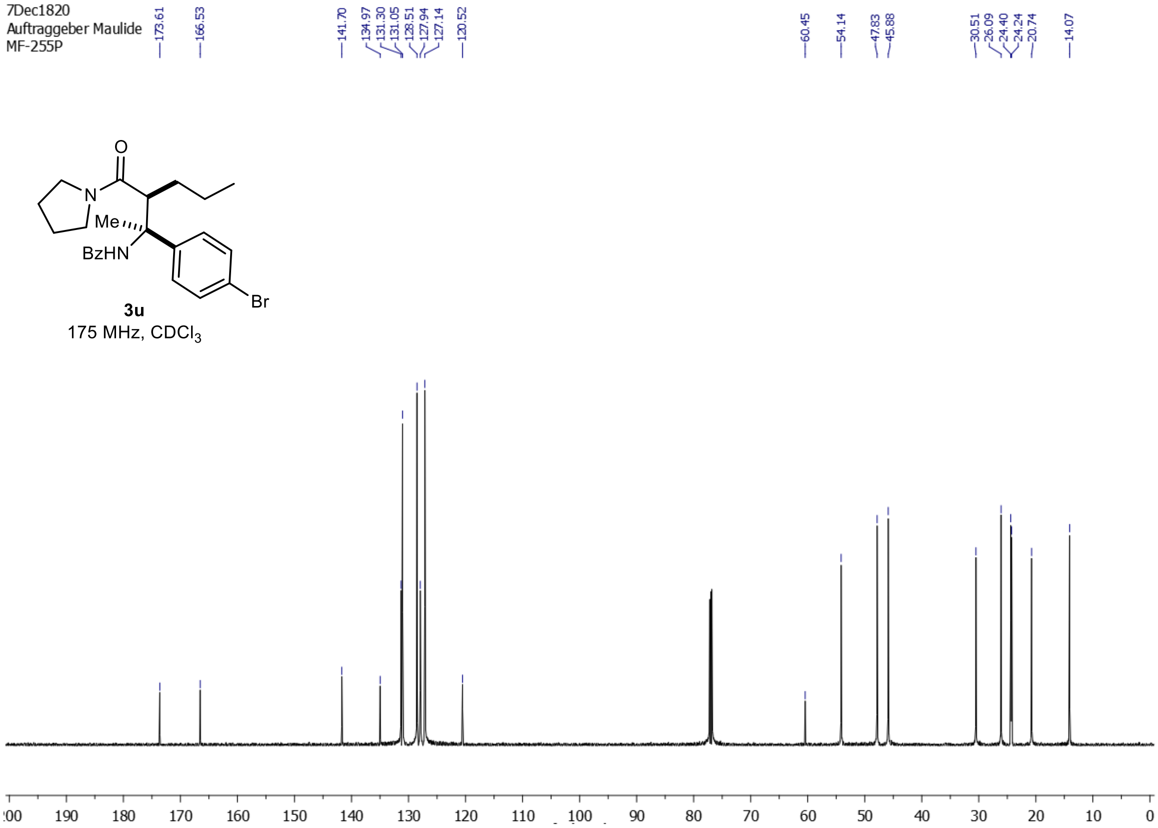
7Dec1820
Auftraggeber Maulide
MF-255P
173.61
166.53
141.70
134.97
131.30
131.05
128.51
127.94
127.14
120.52
60.45
54.14
47.83
45.88
30.51
26.09
24.40
24.24
20.74
14.07
O
N
Me
BzHN
Br
3u
175 MHz, CDCl3

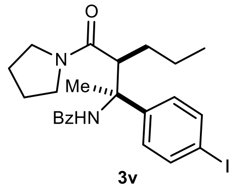

3v

700 MHz, $CDCl_3$

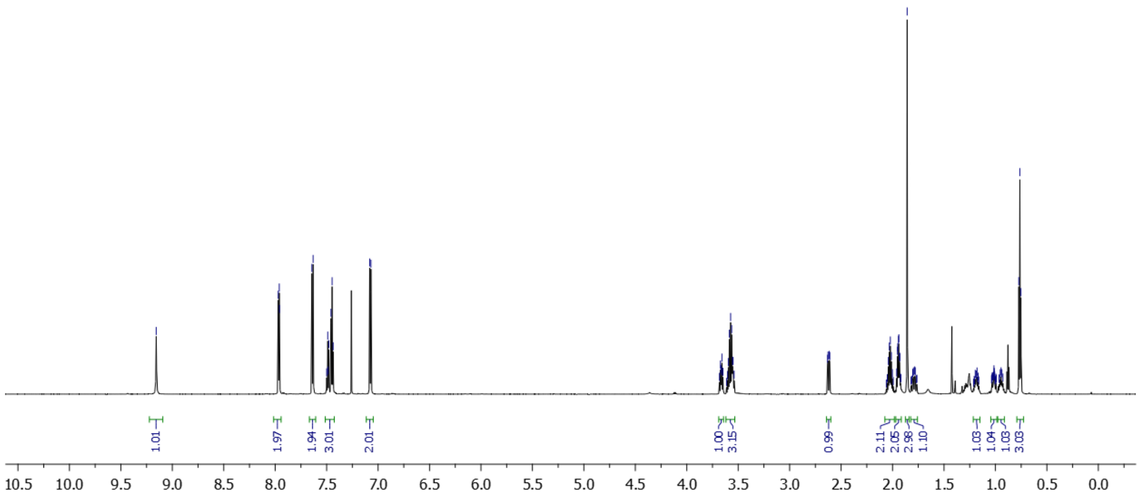

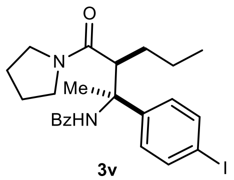

3v

175 MHz, $CDCl_3$

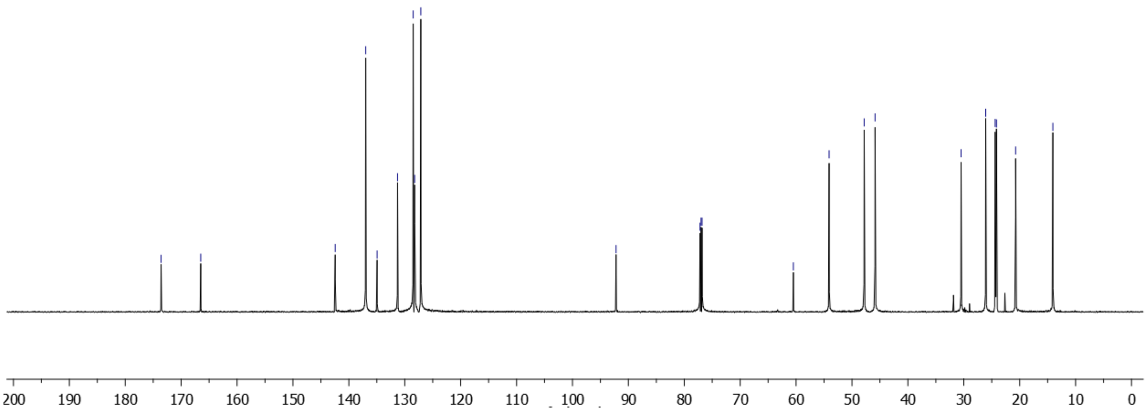

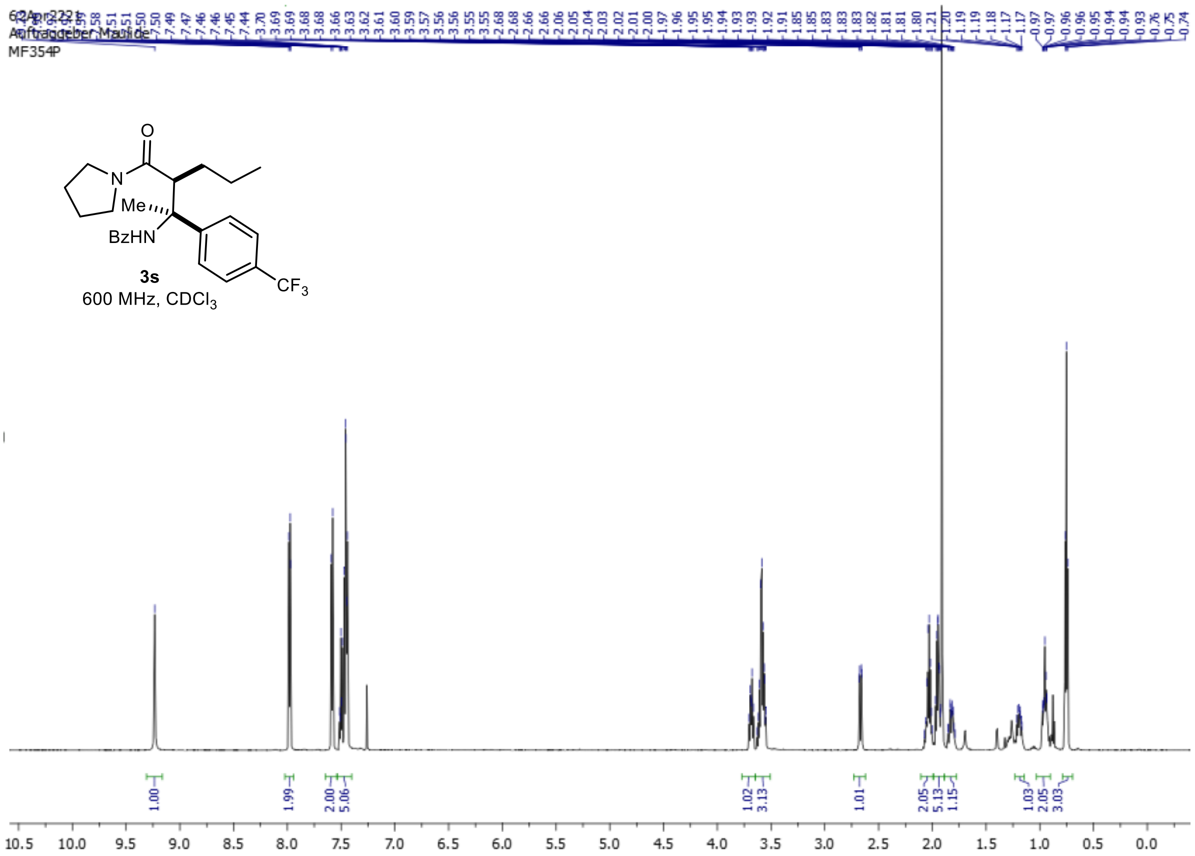
62Apr2221
Auftraggeber Maulide
MF354P
3s
600 MHz, CDCl3
1.00
1.99
2.00
5.06
1.02
3.13
1.01
2.05
5.13
1.15
1.03
2.05
3.03

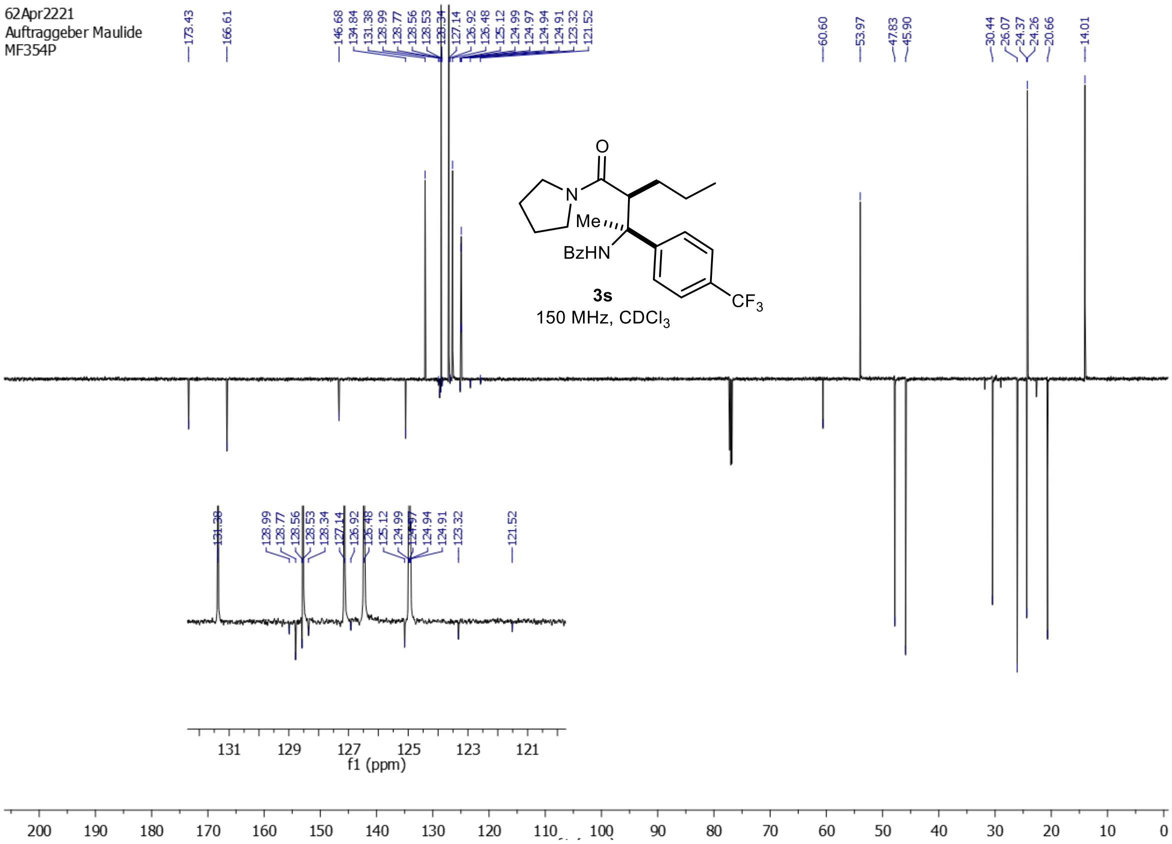
62Apr2221
Auftraggeber Maulide
MF354P
173.43
166.61
146.68
134.84
131.38
128.99
128.77
128.56
128.53
128.34
127.14
126.92
126.48
125.12
124.99
124.97
124.94
124.91
123.32
121.52
60.60
53.97
47.83
45.90
30.44
26.07
24.37
24.26
20.66
14.01
3s
150 MHz, CDCl3
f1 (ppm)

62Apr2221
Auftraggeber Maulide
MF354P

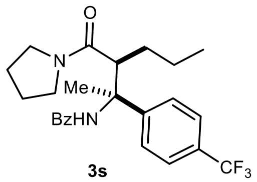

3s
377 MHz, $CDCl_3$

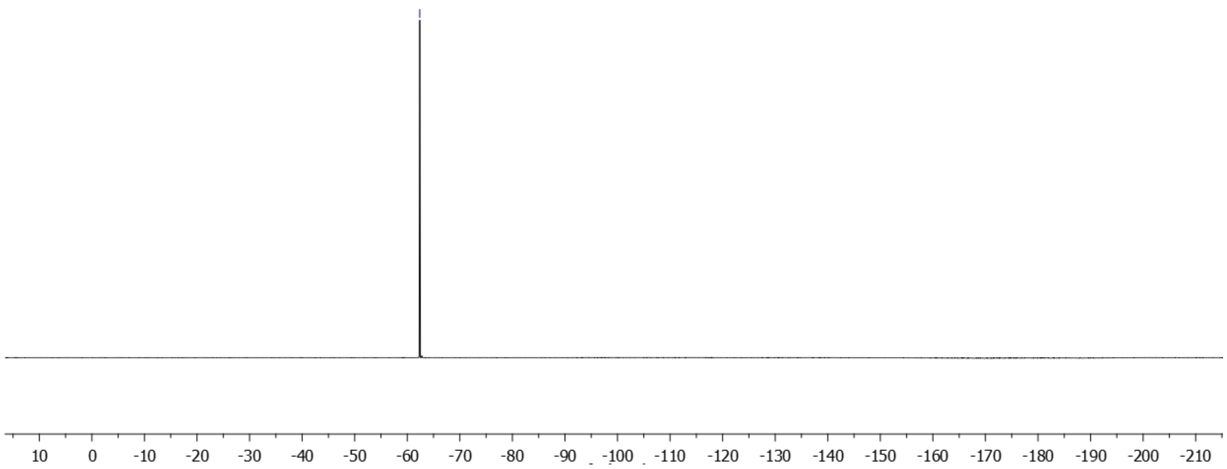

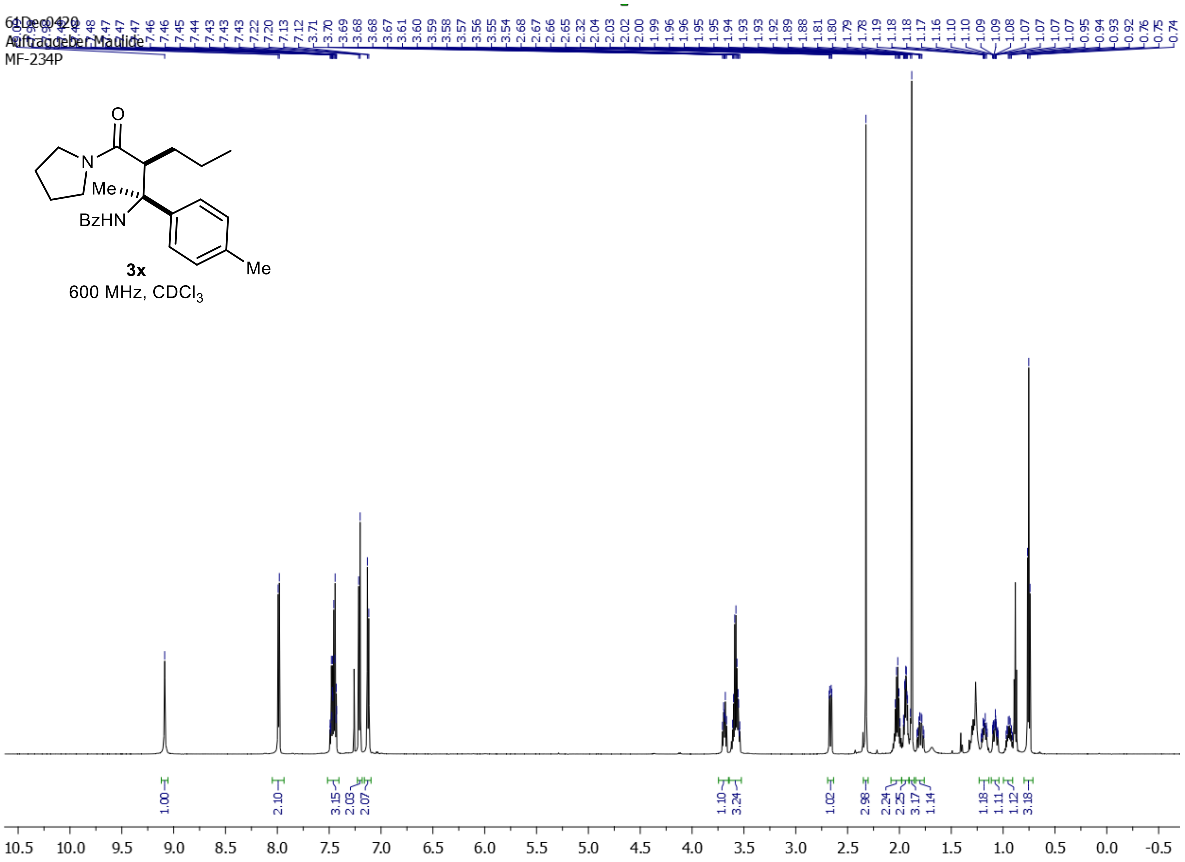
61Dec0420
Auftraggeber Maulide
MF-234P
O
N
Me
BzHN
Me
3x
600 MHz, $CDCl_3$

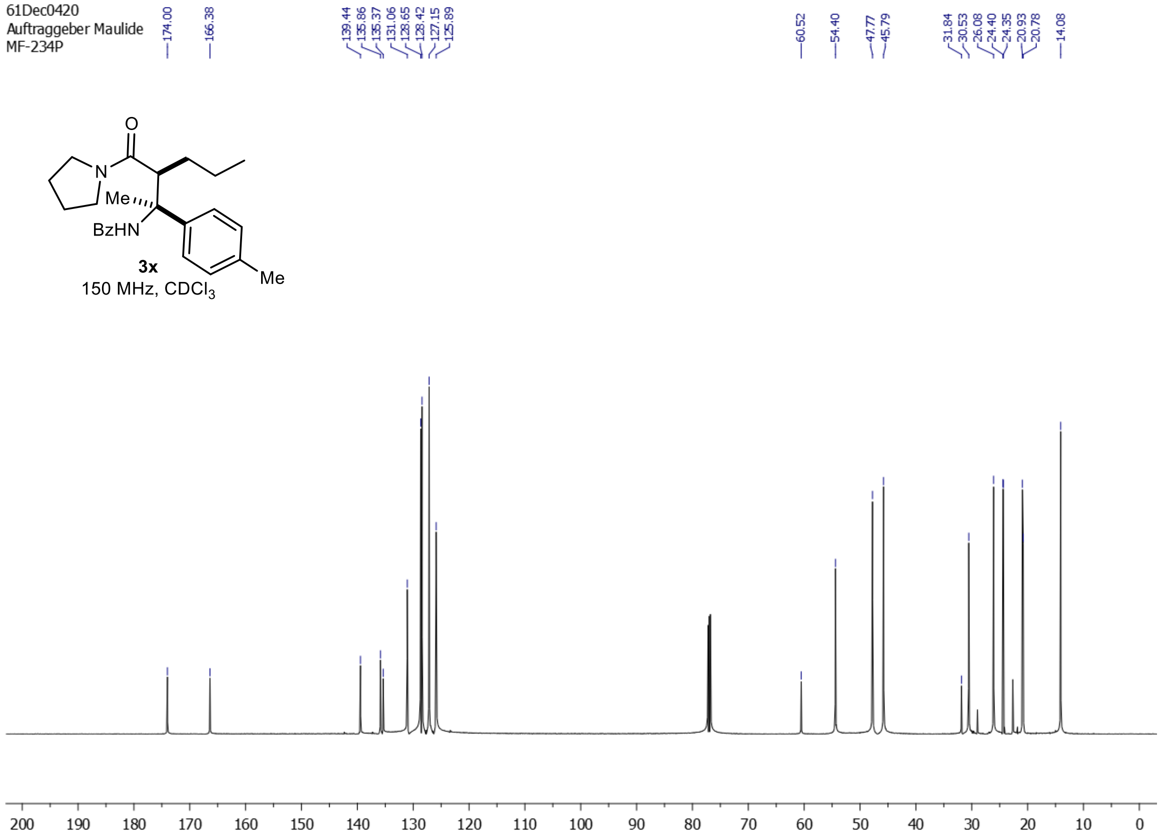
61Dec0420
Auftraggeber Maulide
MF-234P
174.00
166.38
139.44
135.86
135.37
131.06
128.65
128.42
127.15
125.89
60.52
54.40
47.77
45.79
31.84
30.53
26.08
24.40
24.35
20.93
20.78
14.08
O
N
Me
BzHN
Me
3x
150 MHz, $CDCl_3$

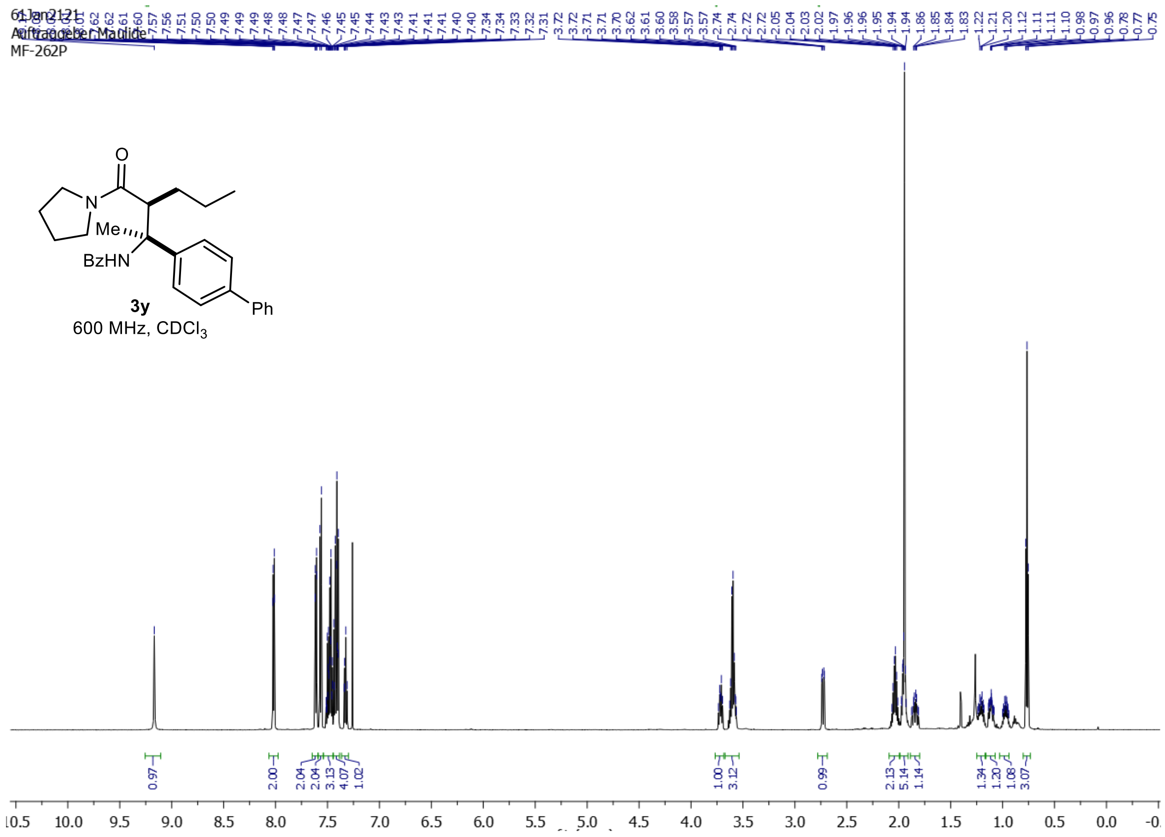
61Jan2121
Auftraggeber Maulide
MF-262P
3y
600 MHz, CDCl$_3$

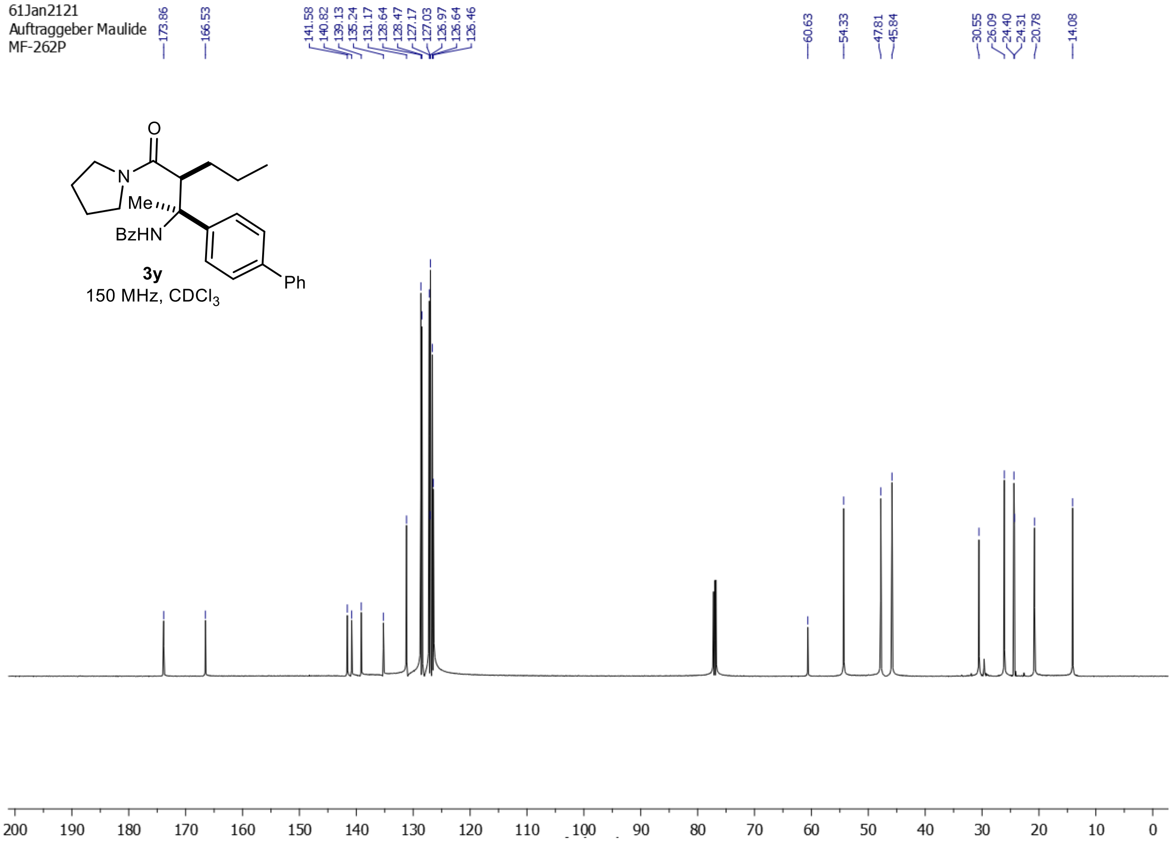
61Jan2121
Auftraggeber Maulide
MF-262P
173.86
166.53
141.58
140.82
139.13
135.24
131.17
128.64
128.47
127.17
127.03
126.97
126.64
126.46
60.63
54.33
47.81
45.84
30.55
26.09
24.40
24.31
20.78
14.08
3y
150 MHz, CDCl$_3$

62Feb0521
Auftraggeber Maulide
MF-271P

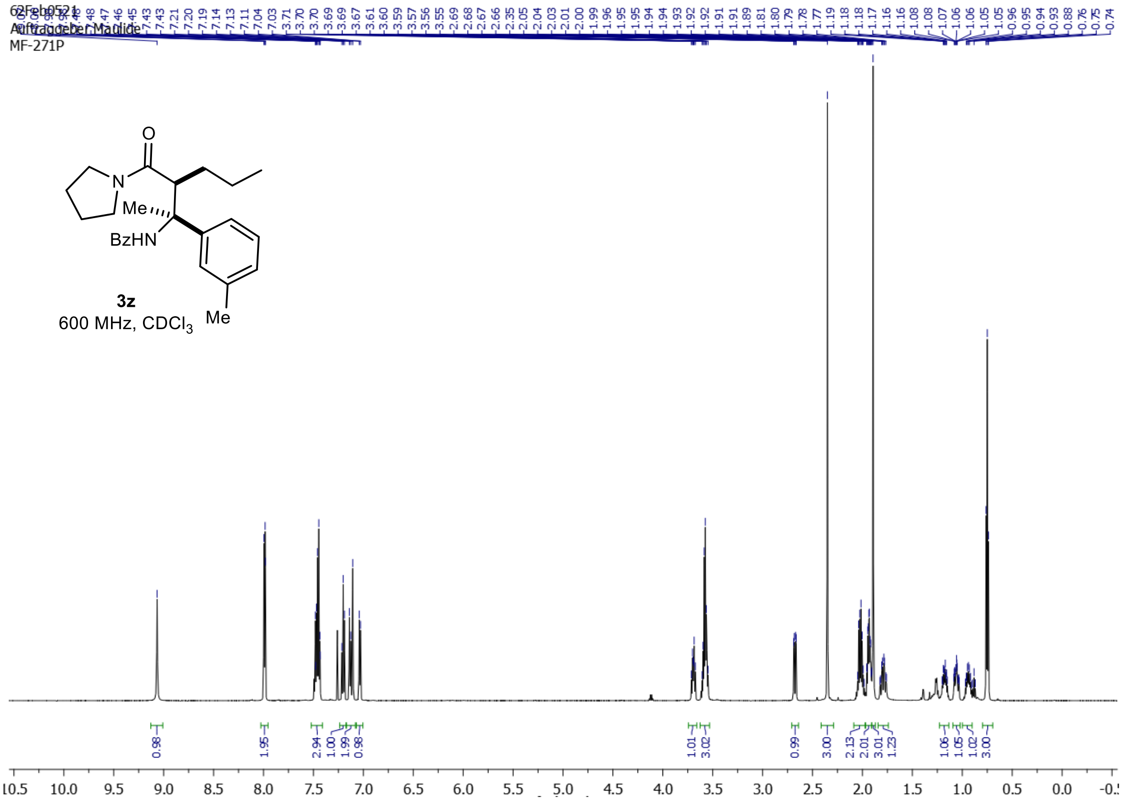

62Feb0521
Auftraggeber Maulide
MF-271P

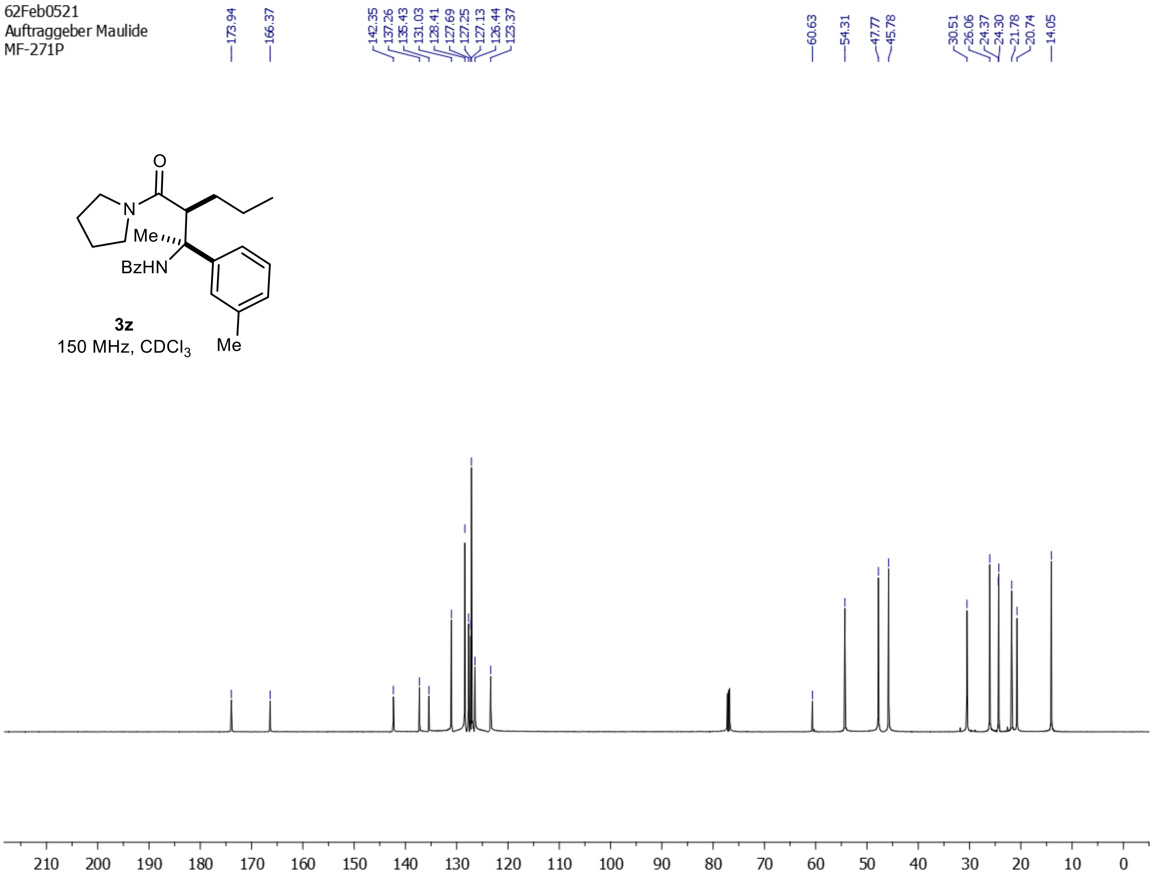

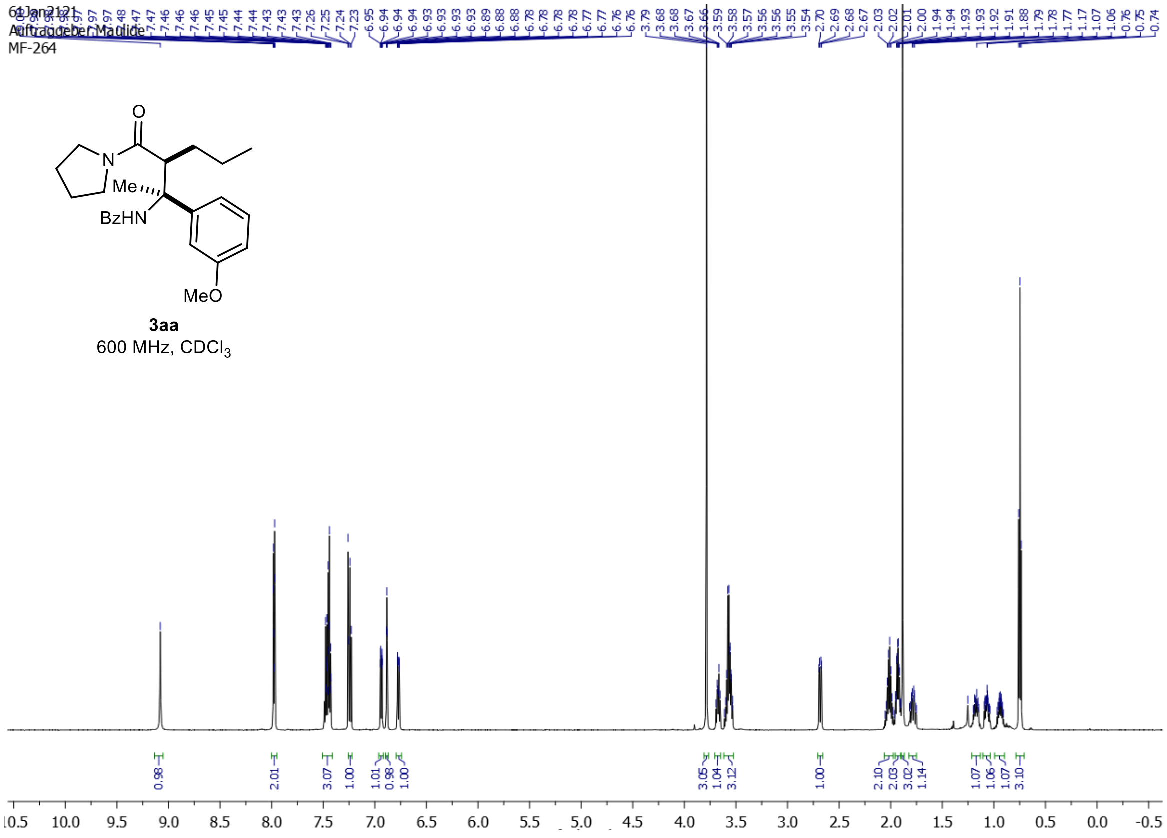
61Jan2121
Auftraggeber Maulide
MF-264
BzHN
Me
MeO
3aa
600 MHz, CDCl3

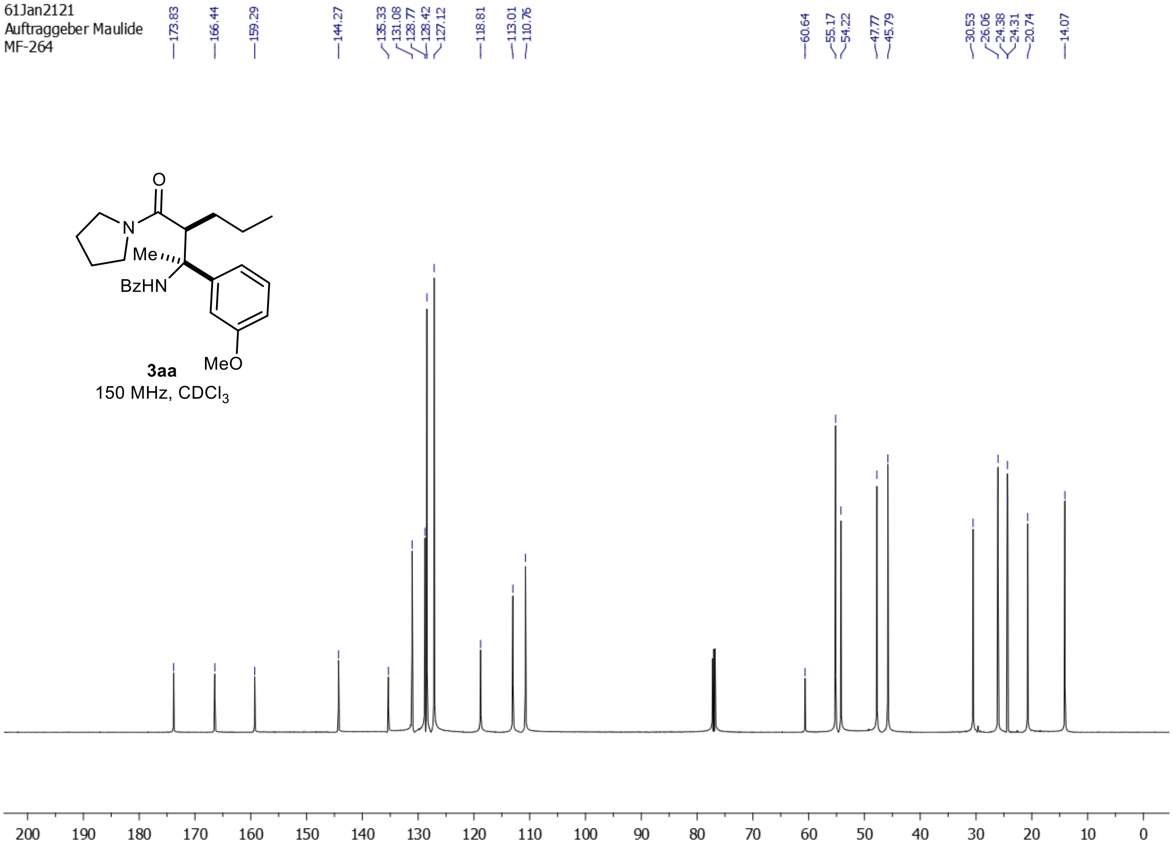
61Jan2121
Auftraggeber Maulide
MF-264
173.83
166.44
159.29
144.27
135.33
131.08
128.77
128.42
127.12
118.81
113.01
110.76
60.64
55.17
54.22
47.77
45.79
30.53
26.06
24.38
24.31
20.74
14.07
BzHN
Me
MeO
3aa
150 MHz, CDCl3

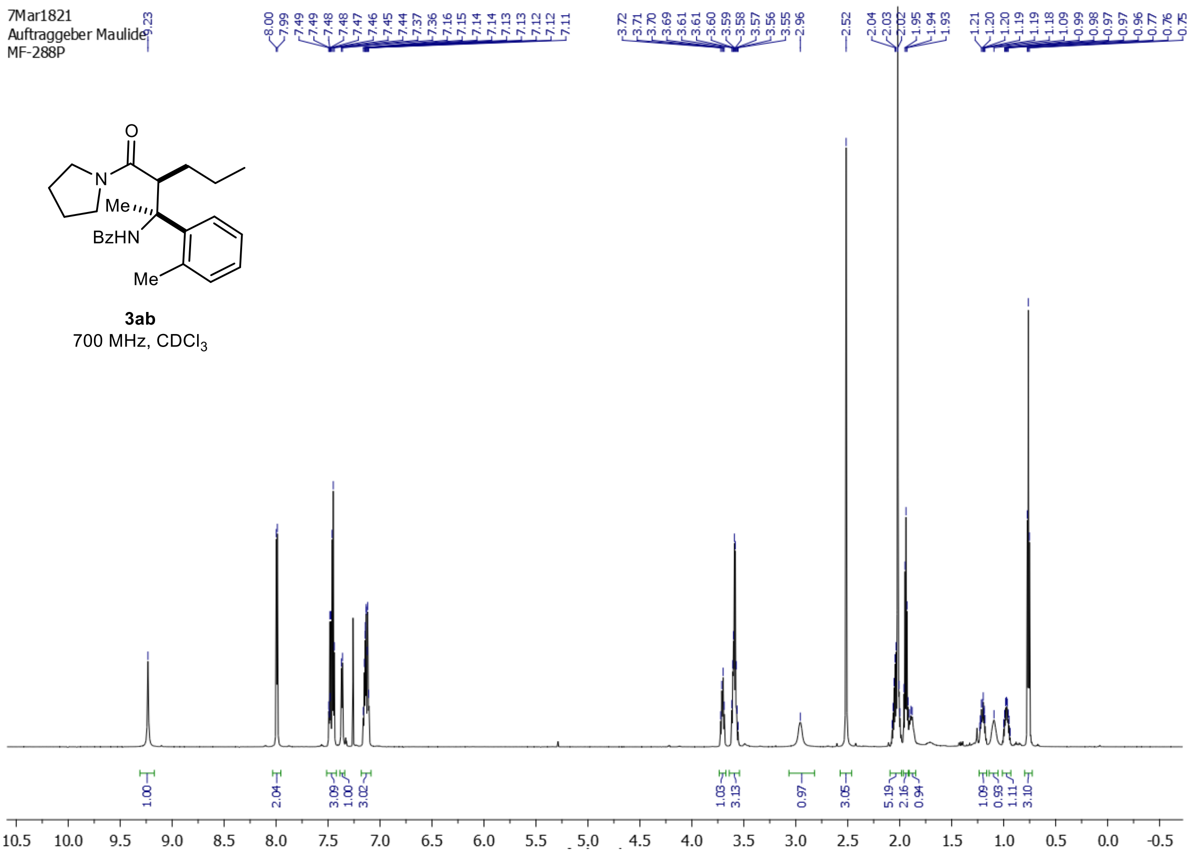
7Mar1821
Auftraggeber Maulide
MF-288P
9.23
8.00 7.99 7.49 7.49 7.48 7.47 7.46 7.45 7.44 7.37 7.36 7.16 7.15 7.14 7.14 7.13 7.13 7.12 7.12 7.11
3.72 3.71 3.70 3.69 3.61 3.61 3.60 3.59 3.58 3.57 3.56 3.55 2.96
2.52
2.04 2.03 2.02 1.95 1.94 1.93
1.21 1.20 1.20 1.19 1.19 1.18 1.09 0.99 0.98 0.97 0.97 0.96 0.77 0.76 0.75
O
N
Me
BzHN
Me
3ab
700 MHz, CDCl3
1.00 2.04 3.09 1.00 3.02 1.03 3.13 0.97 3.05 5.19 2.16 0.94 1.09 0.93 1.11 3.10
10.5 10.0 9.5 9.0 8.5 8.0 7.5 7.0 6.5 6.0 5.5 5.0 4.5 4.0 3.5 3.0 2.5 2.0 1.5 1.0 0.5 0.0 -0.5

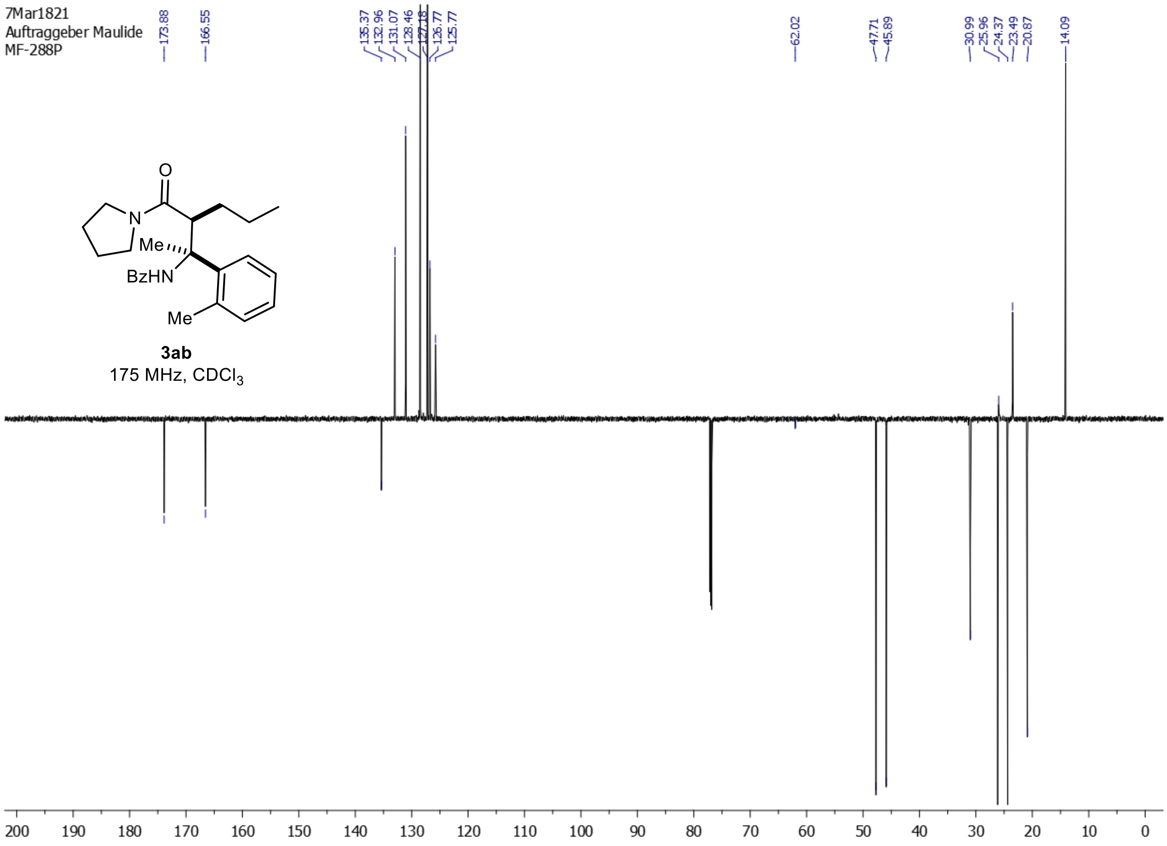
7Mar1821
Auftraggeber Maulide
MF-288P
173.88
166.55
135.37 132.96 131.07 128.46 127.18 126.77 125.77
62.02
47.71 45.89
30.99 25.96 24.37 23.49 20.87
14.09
O
N
Me
BzHN
Me
3ab
175 MHz, CDCl3
200 190 180 170 160 150 140 130 120 110 100 90 80 70 60 50 40 30 20 10 0

HSQC

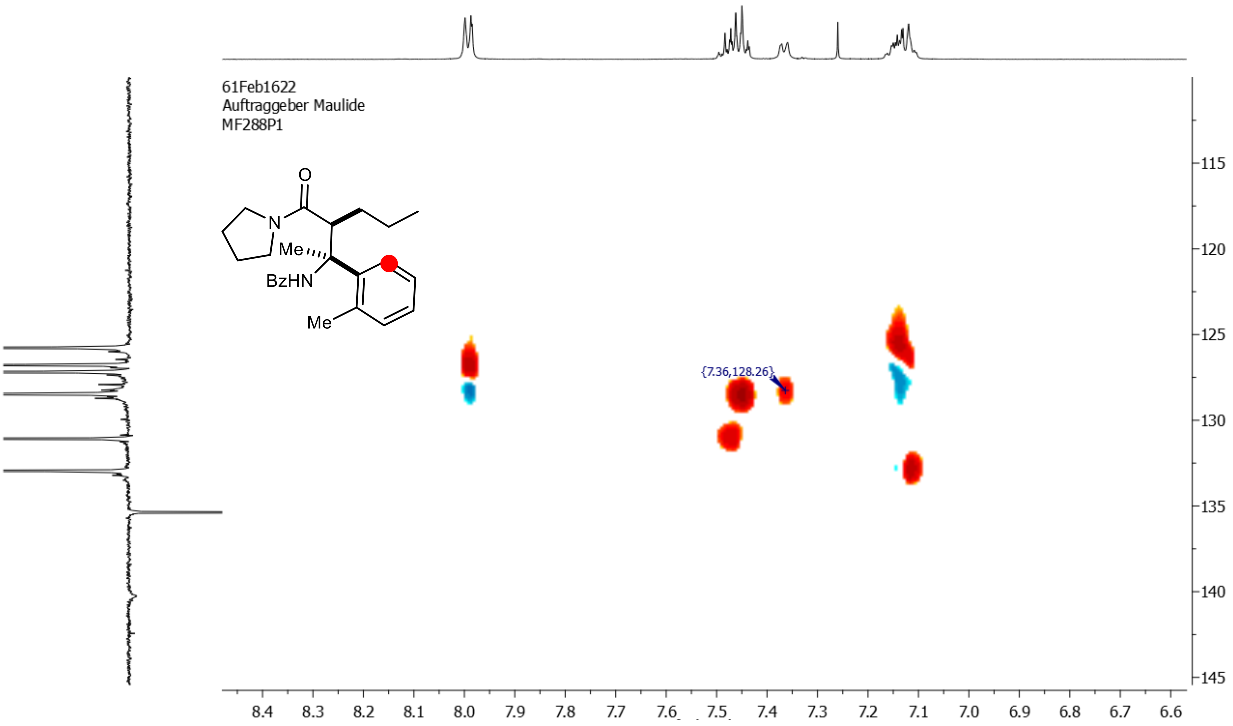
61Feb1622
Auftraggeber Maulide
MF288P1
O
N
Me
BzHN
Me
{7.36,128.26}
115
120
125
130
135
140
145
8.4
8.3
8.2
8.1
8.0
7.9
7.8
7.7
7.6
7.5
7.4
7.3
7.2
7.1
7.0
6.9
6.8
6.7
6.6

HMBC

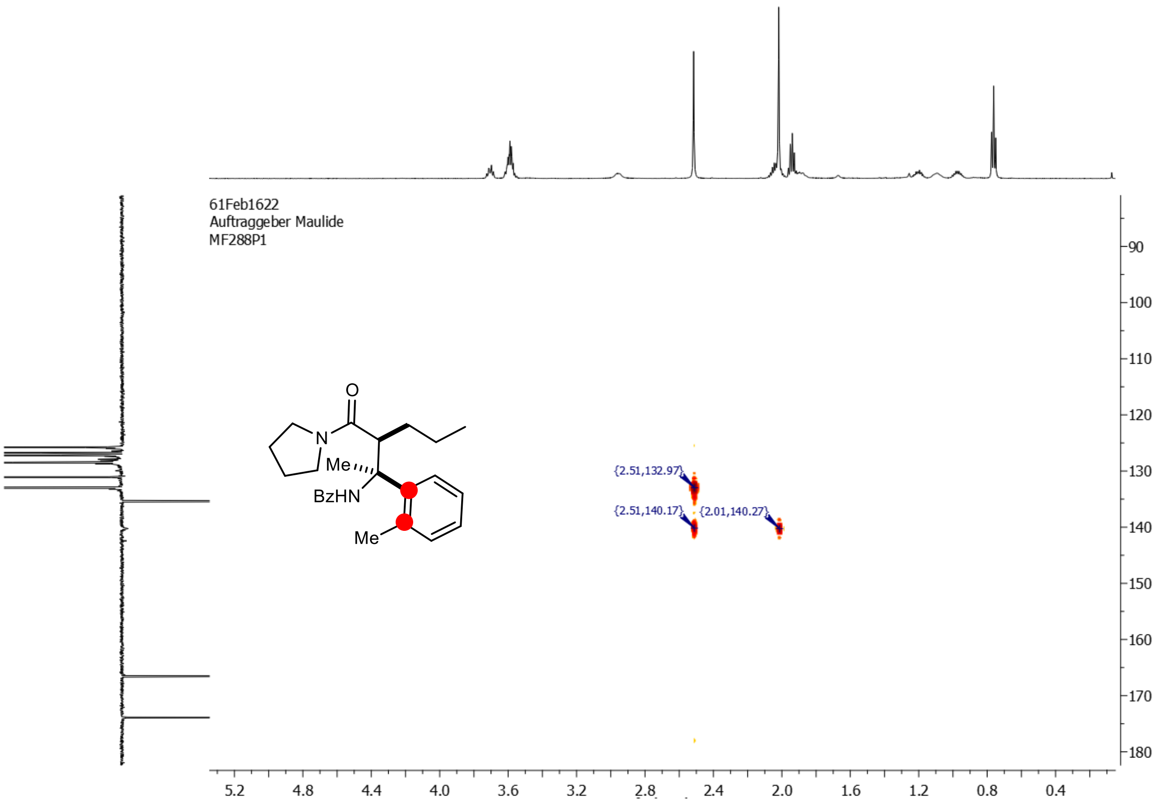
61Feb1622
Auftraggeber Maulide
MF288P1
O
N
Me
BzHN
Me
{2.51,132.97}
{2.51,140.17}
{2.01,140.27}
90
100
110
120
130
140
150
160
170
180
5.2
4.8
4.4
4.0
3.6
3.2
2.8
2.4
2.0
1.6
1.2
0.8
0.4

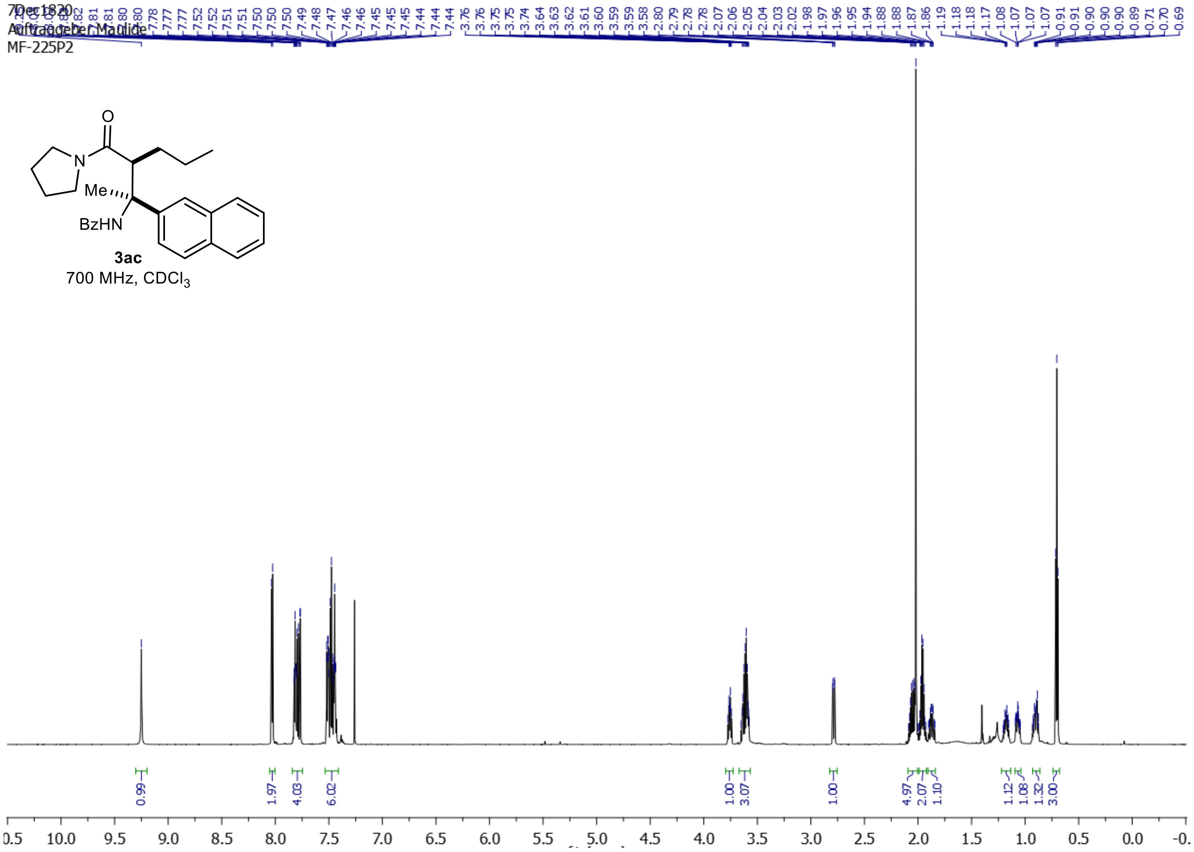
7Dec1820
Auftraggeber Maulide
MF-225P2
Me
BzHN
N
O
3ac
700 MHz, CDCl3

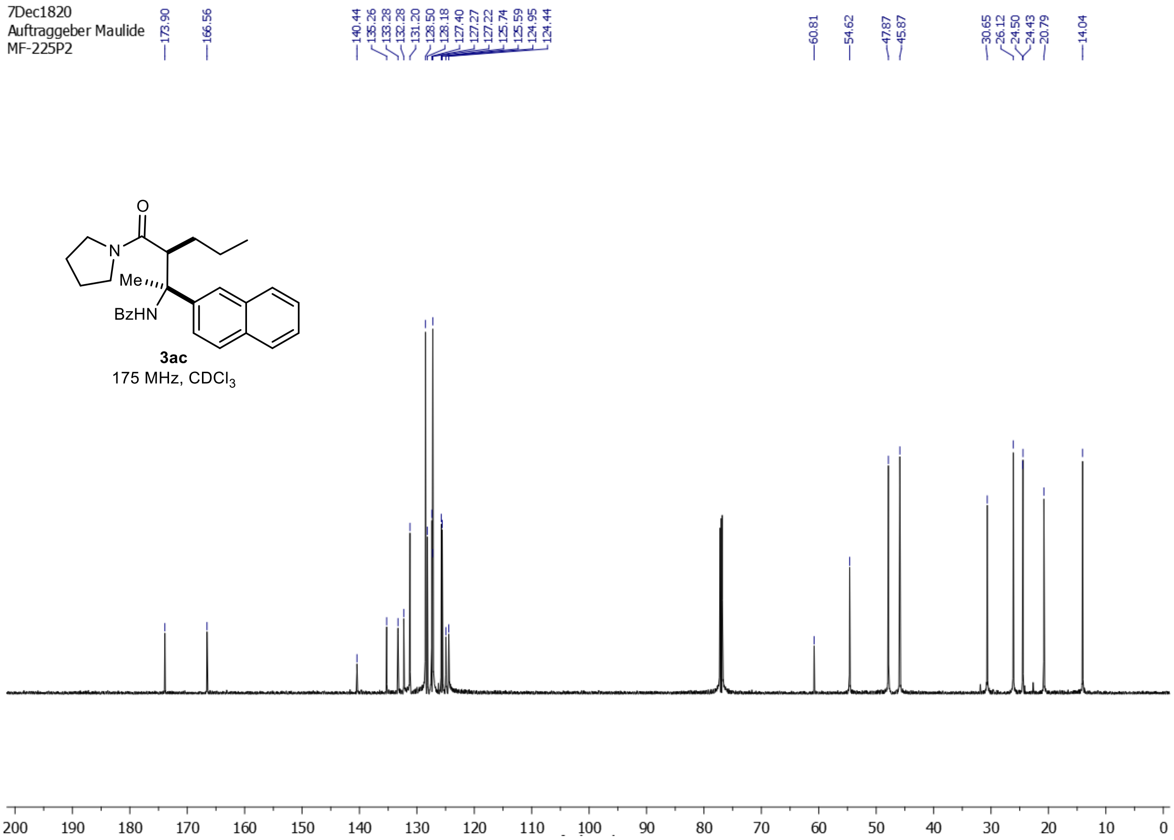
7Dec1820
Auftraggeber Maulide
MF-225P2
173.90
166.56
140.44
135.26
133.28
132.28
131.20
128.50
128.18
127.40
127.27
127.22
125.74
125.59
124.95
124.44
60.81
54.62
47.87
45.87
30.65
26.12
24.50
24.43
20.79
14.04
Me
BzHN
N
O
3ac
175 MHz, CDCl3

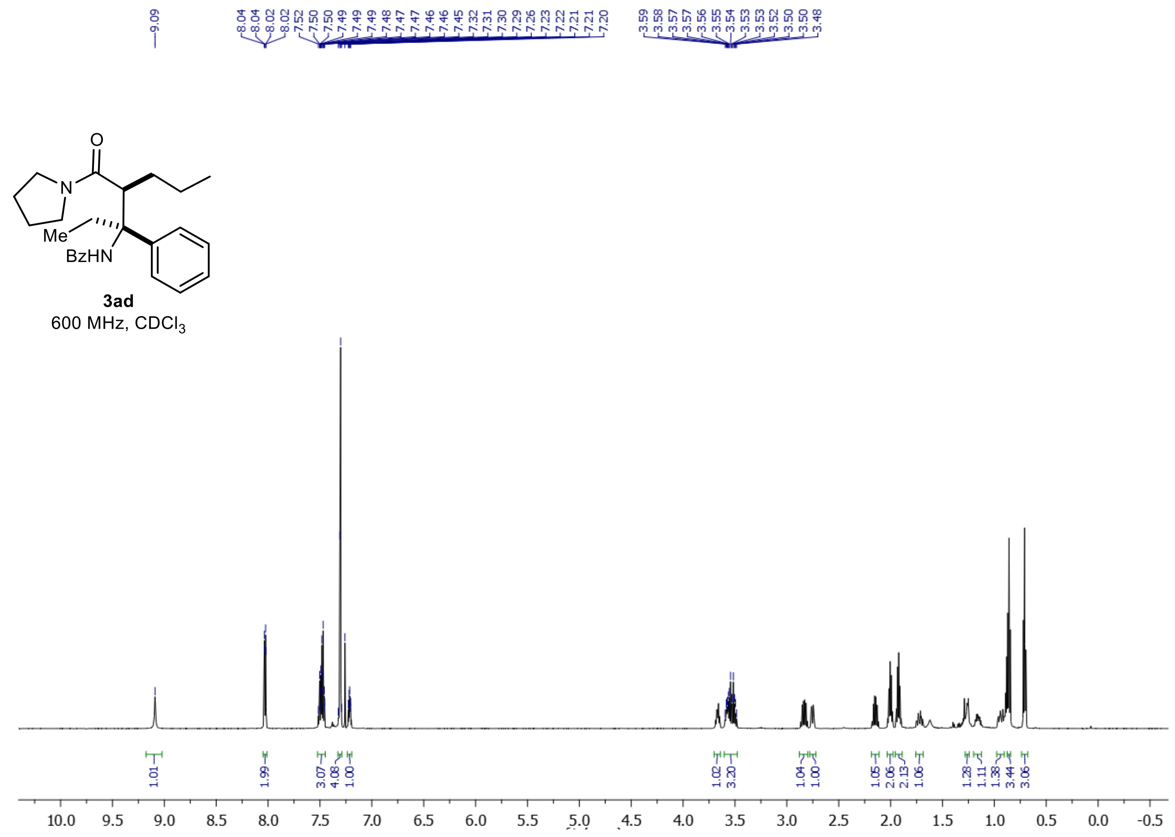
9.09
8.04
8.04
8.02
8.02
7.52
7.50
7.50
7.49
7.49
7.48
7.47
7.47
7.46
7.46
7.45
7.32
7.31
7.30
7.29
7.26
7.23
7.22
7.21
7.21
7.20
3.59
3.58
3.57
3.57
3.56
3.55
3.54
3.53
3.53
3.52
3.50
3.50
3.48
O
N
Me
BzHN
3ad
600 MHz, CDCl3
1.01
1.99
3.07
4.08
1.00
1.02
3.20
1.04
1.00
1.05
2.06
2.13
1.06
1.28
1.11
1.38
3.44
3.06
10.0
9.5
9.0
8.5
8.0
7.5
7.0
6.5
6.0
5.5
5.0
4.5
4.0
3.5
3.0
2.5
2.0
1.5
1.0
0.5
0.0
-0.5

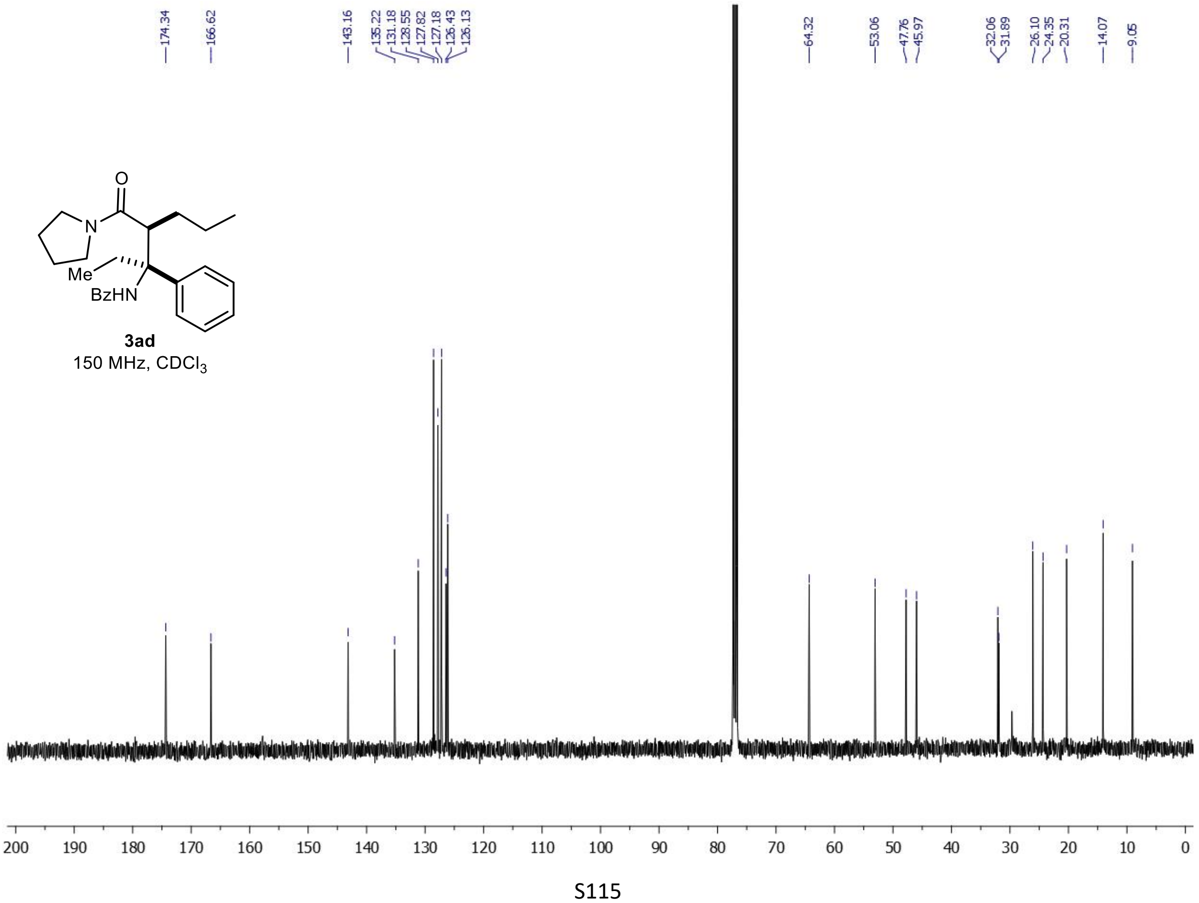
174.34
166.62
143.16
135.22
131.18
128.55
127.82
127.18
126.43
126.13
64.32
53.06
47.76
45.97
32.06
31.89
26.10
24.35
20.31
14.07
9.05
O
N
Me
BzHN
3ad
150 MHz, CDCl3
200
190
180
170
160
150
140
130
120
110
100
90
80
70
60
50
40
30
20
10
0

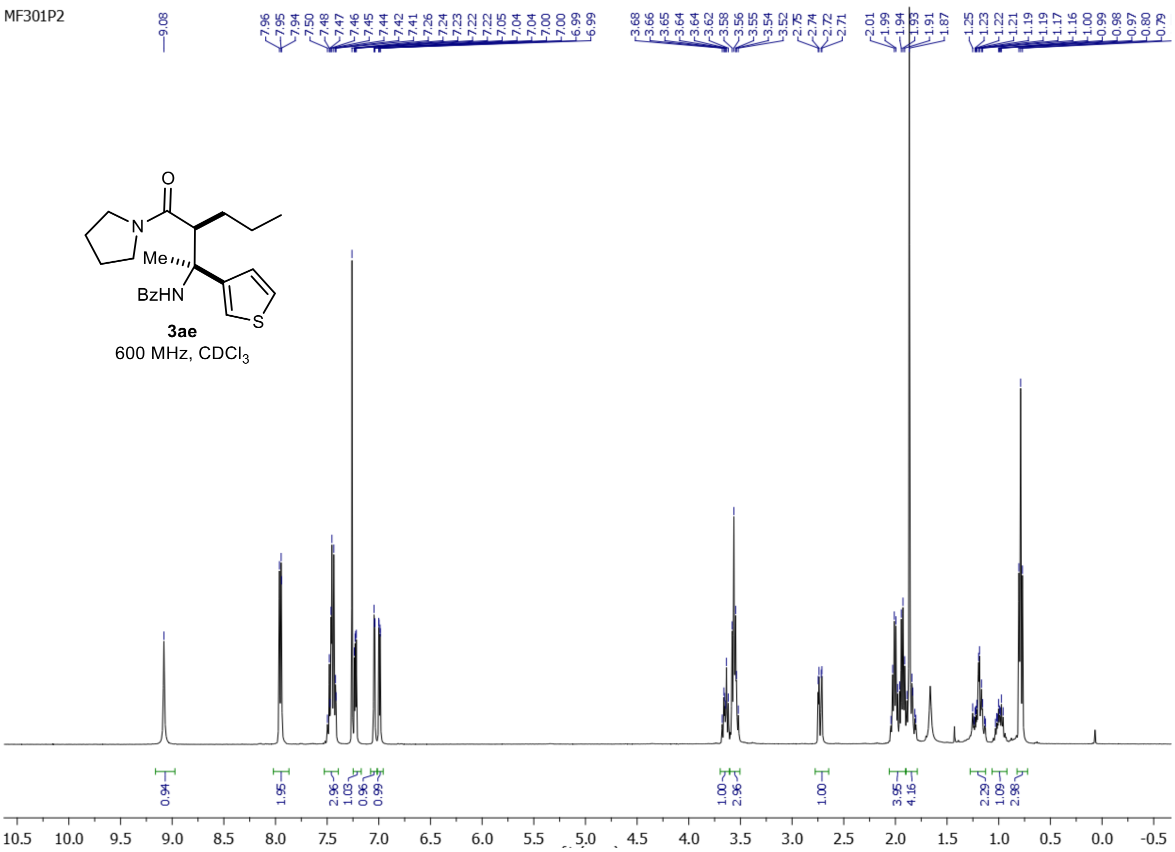

MF301P2
3ae
600 MHz, CDCl3

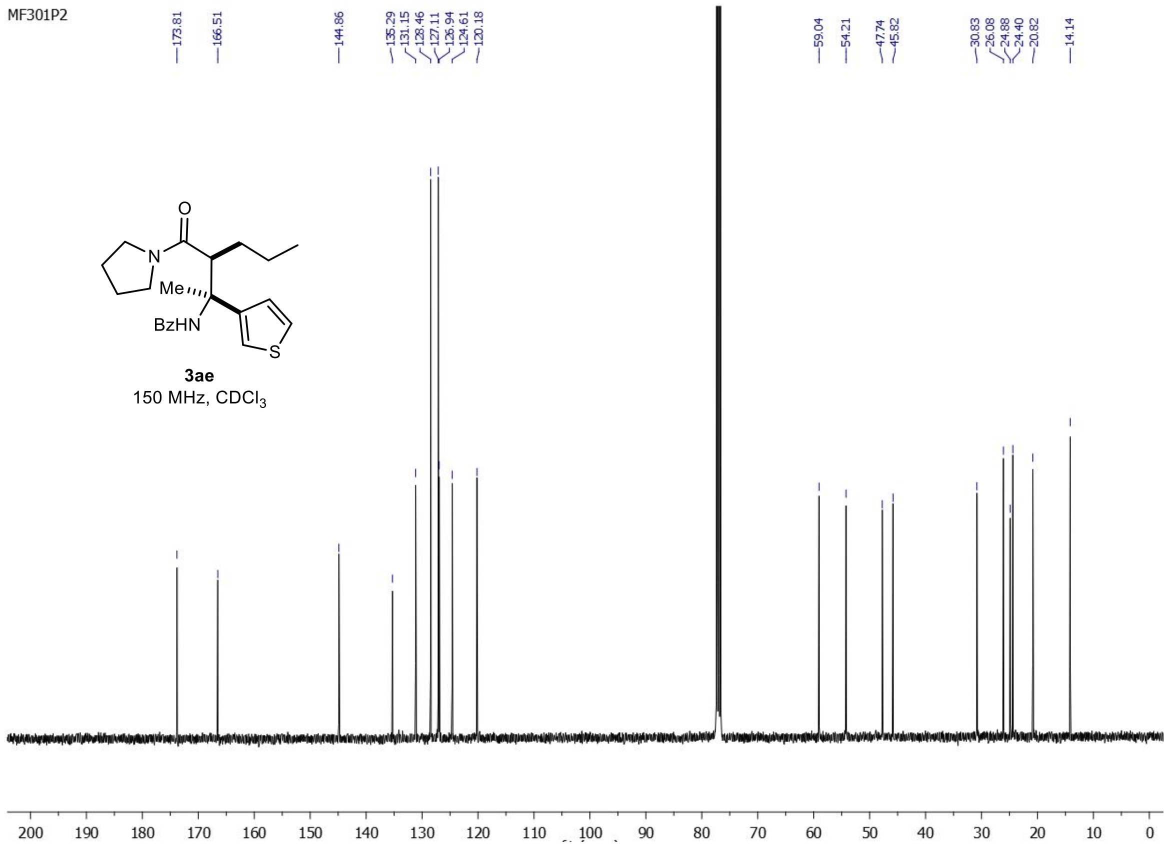

MF301P2
3ae
150 MHz, CDCl3

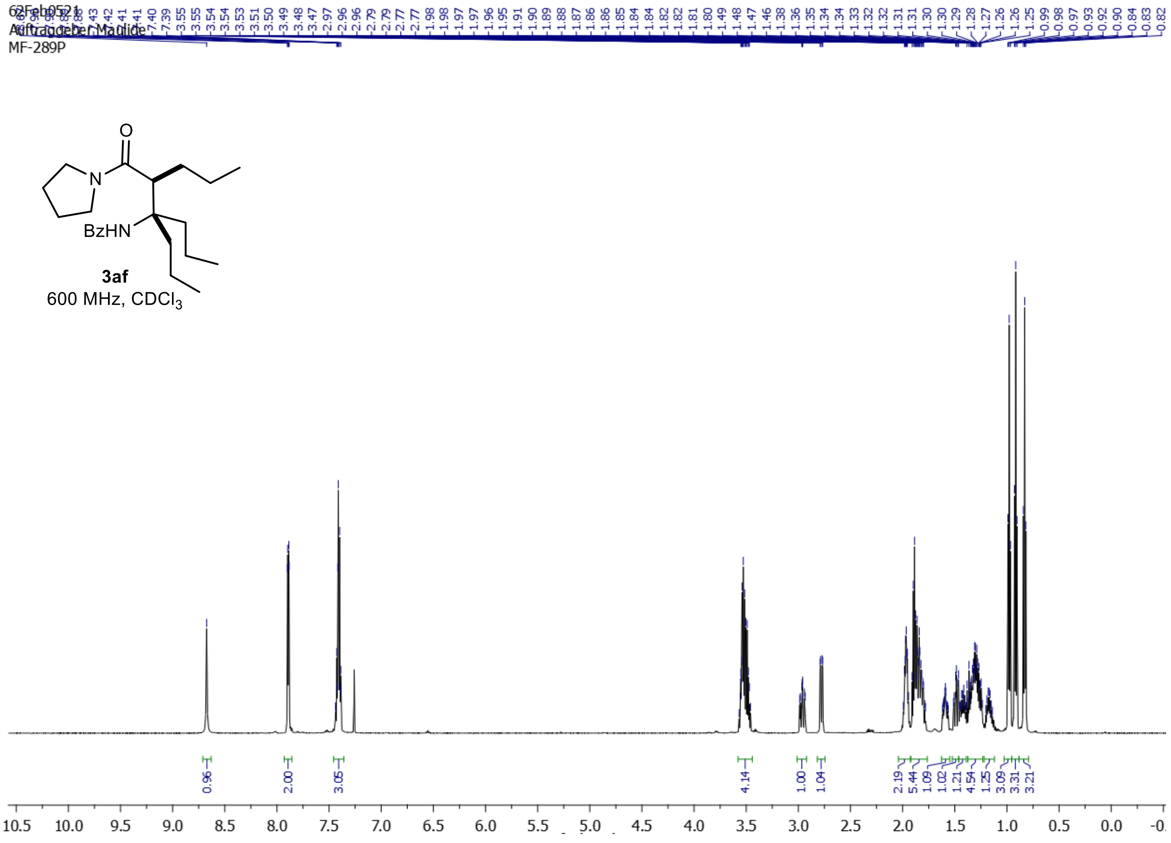
62Feb0521
Auftraggeber Maulide
MF-289P
BzHN
3af
600 MHz, CDCl$_3$
0.96
2.00
3.05
4.14
1.00
1.04
2.19
5.44
1.09
1.02
1.21
4.54
1.25
3.09
3.31
3.21

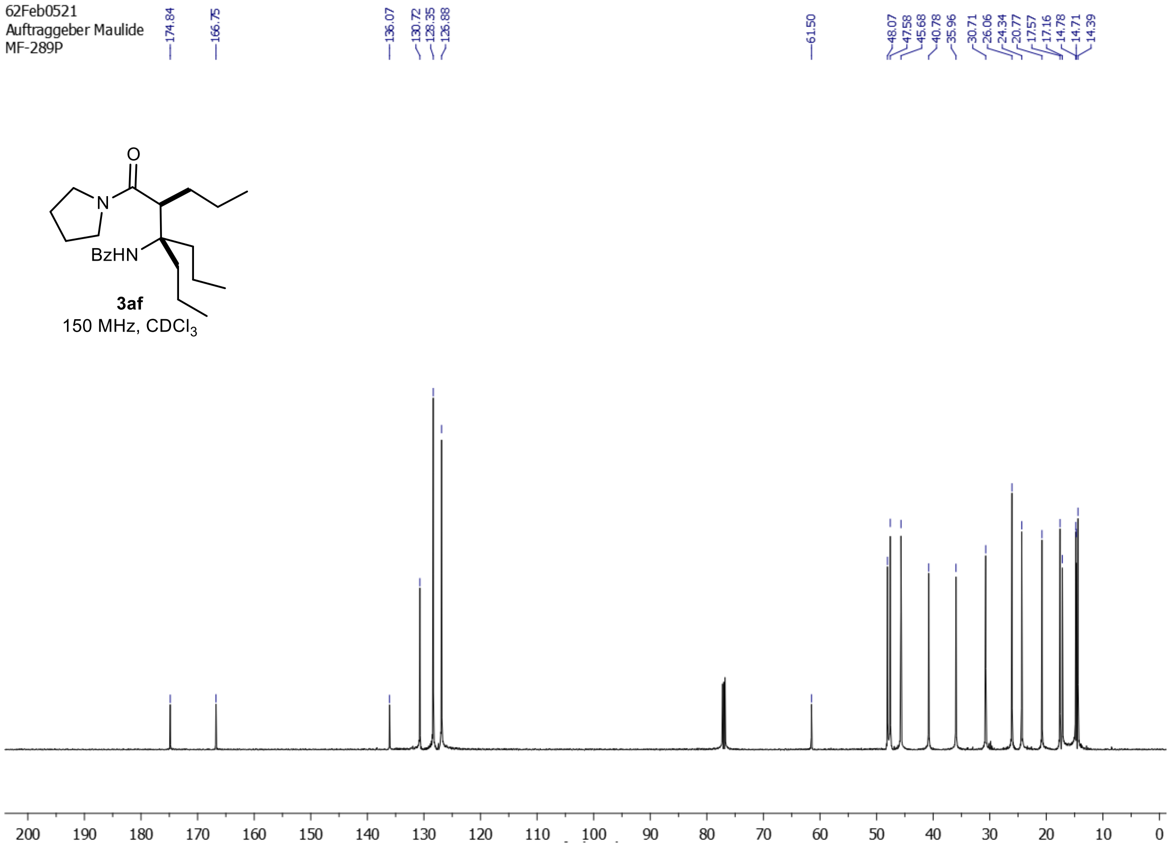
62Feb0521
Auftraggeber Maulide
MF-289P
174.84
166.75
136.07
130.72
128.35
126.88
61.50
48.07
47.58
45.68
40.78
35.96
30.71
26.06
24.34
20.77
17.57
17.16
14.78
14.71
14.39
BzHN
3af
150 MHz, CDCl$_3$

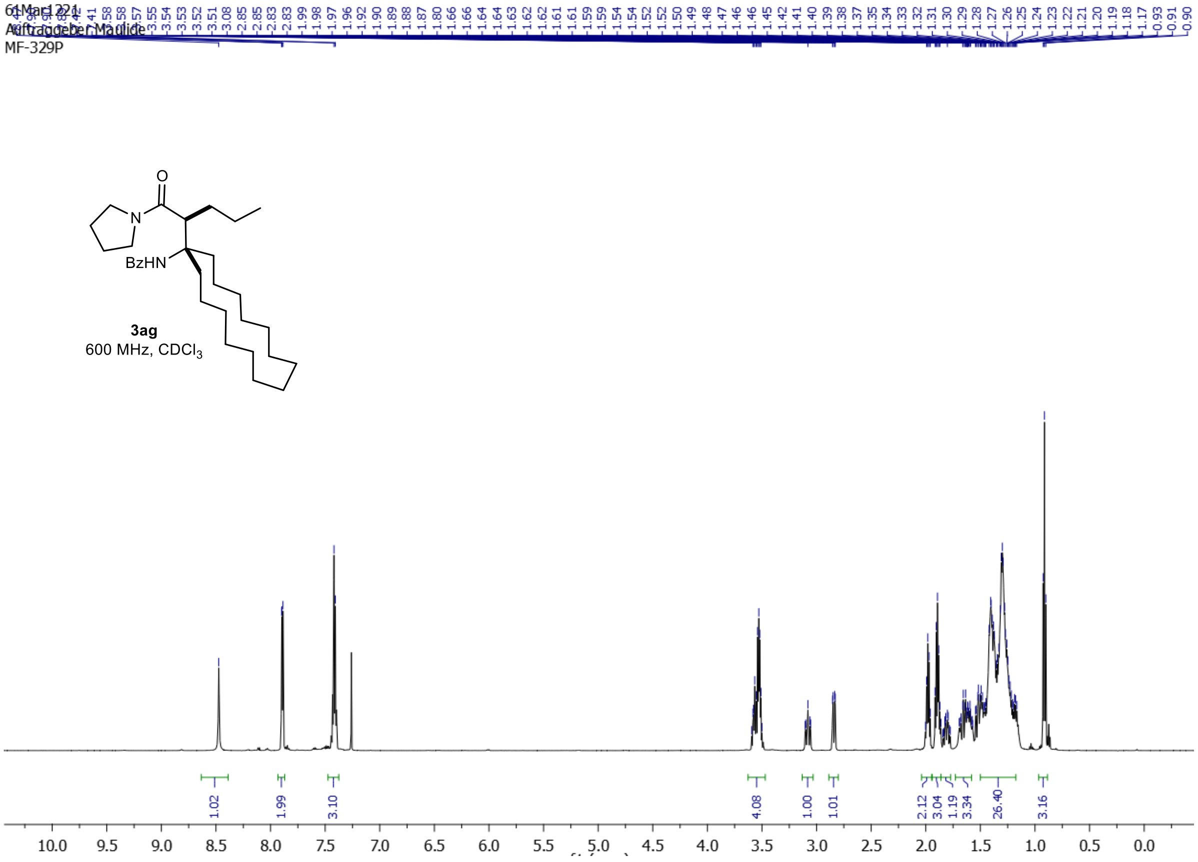
61Mar1221
Auftraggeber Maulide
MF-329P
3ag
600 MHz, CDCl3

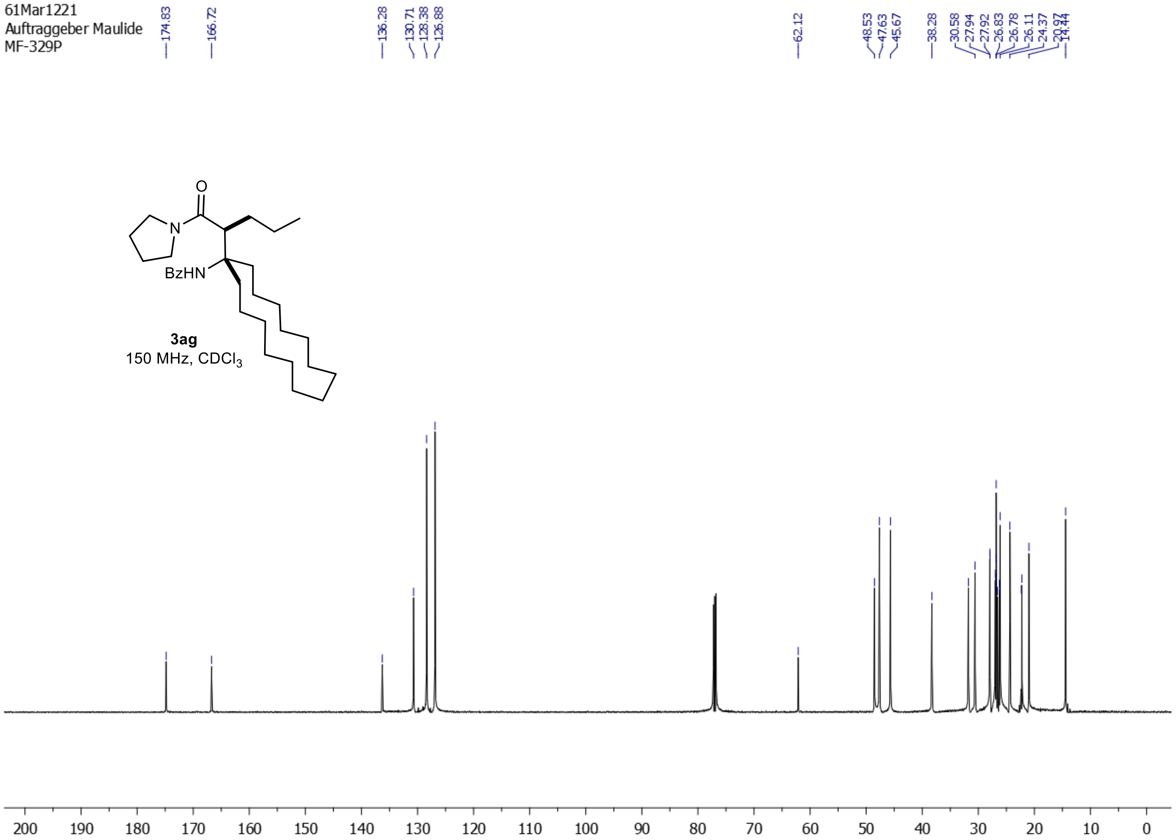
61Mar1221
Auftraggeber Maulide
MF-329P
3ag
150 MHz, CDCl3

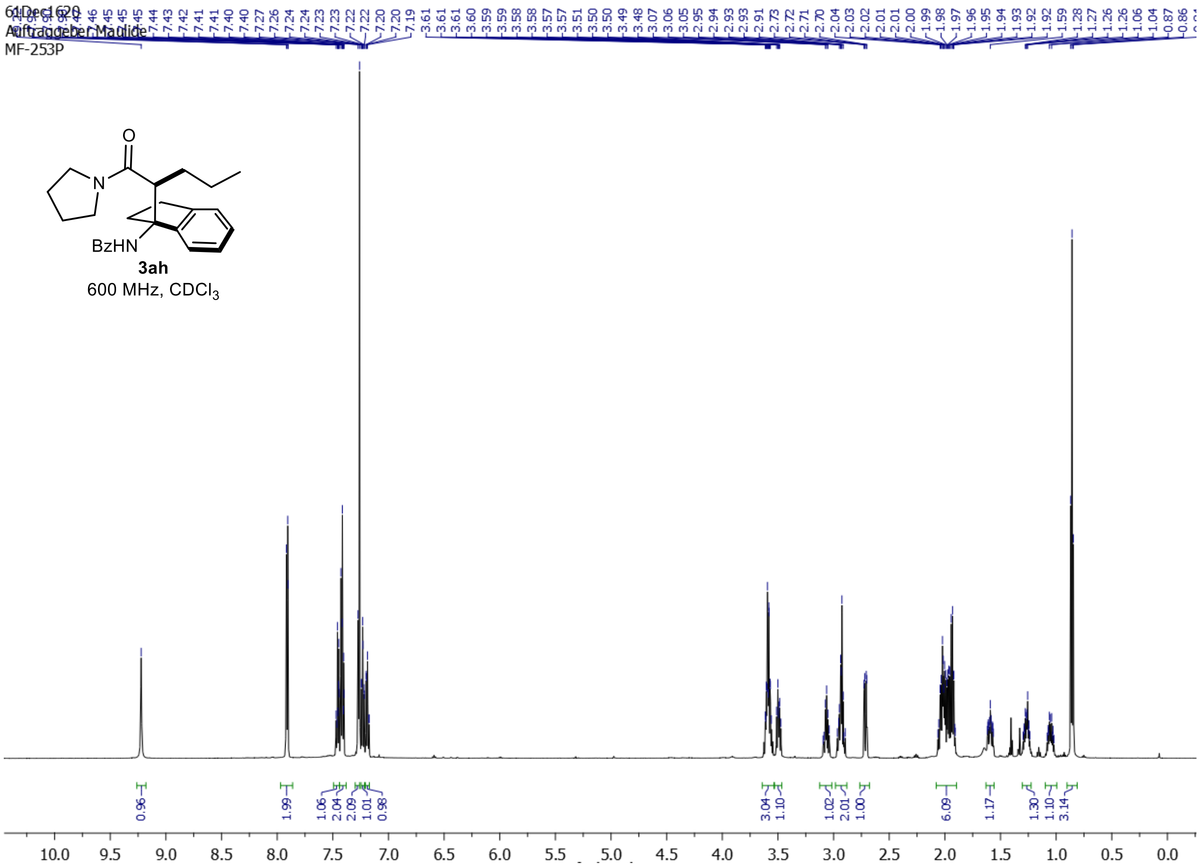

61Dec1620
Auftraggeber Maulide
MF-253P
7.44 7.43 7.42 7.41 7.41 7.40 7.40 7.27 7.26 7.24 7.24 7.23 7.23 7.22 7.22 7.20 7.20 7.19
3.61 3.61 3.60 3.59 3.59 3.58 3.58 3.57 3.57 3.51 3.50 3.50 3.49 3.48 3.07 3.06 3.05 2.95 2.94 2.93 2.93 2.91 2.73 2.72 2.71 2.70 2.04 2.03 2.02 2.01 2.01 2.00 1.99 1.98 1.97 1.96 1.95 1.94 1.93 1.92 1.92 1.59 1.28 1.27 1.26 1.26 1.06 1.04 0.87 0.86
O
N
BzHN
3ah
600 MHz, $CDCl_3$
0.96 1.99 1.06 2.04 2.09 1.01 0.98 3.04 1.10 1.02 2.01 1.00 6.09 1.17 1.30 1.10 3.14
10.0 9.5 9.0 8.5 8.0 7.5 7.0 6.5 6.0 5.5 5.0 4.5 4.0 3.5 3.0 2.5 2.0 1.5 1.0 0.5 0.0

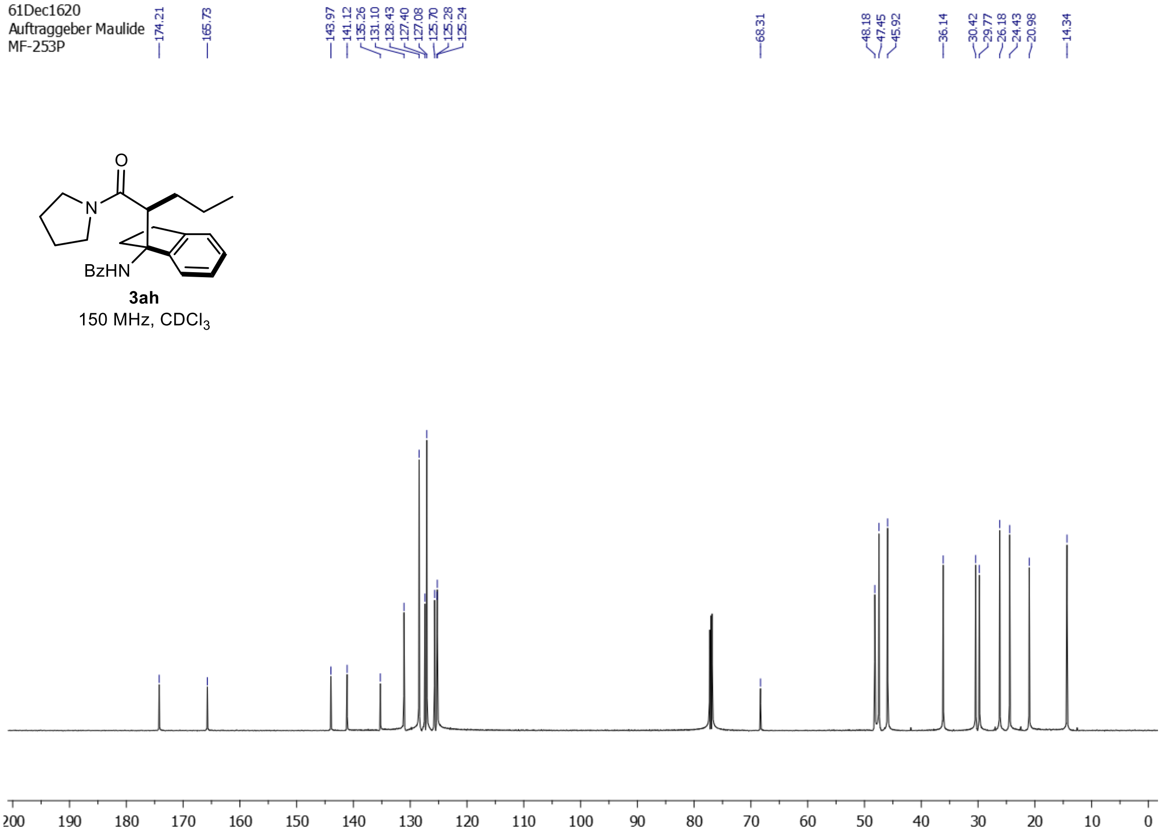

61Dec1620
Auftraggeber Maulide
MF-253P
174.21
165.73
143.97 141.12 135.26 131.10 128.43 127.40 127.08 125.70 125.28 125.24
68.31
48.18 47.45 45.92
36.14
30.42 29.77 26.18 24.43 20.98
14.34
O
N
BzHN
3ah
150 MHz, $CDCl_3$
200 190 180 170 160 150 140 130 120 110 100 90 80 70 60 50 40 30 20 10 0

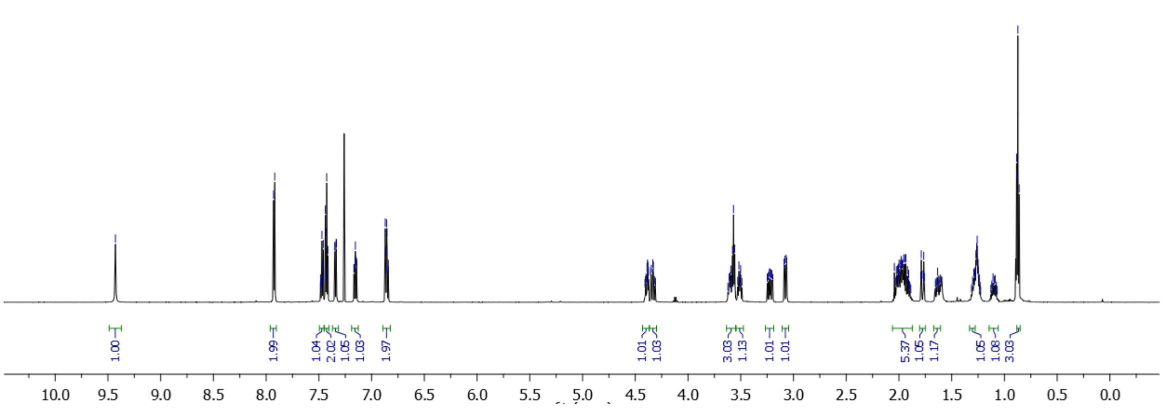

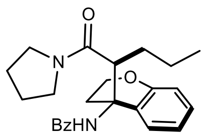

3ai
600 MHz, $CDCl_3$

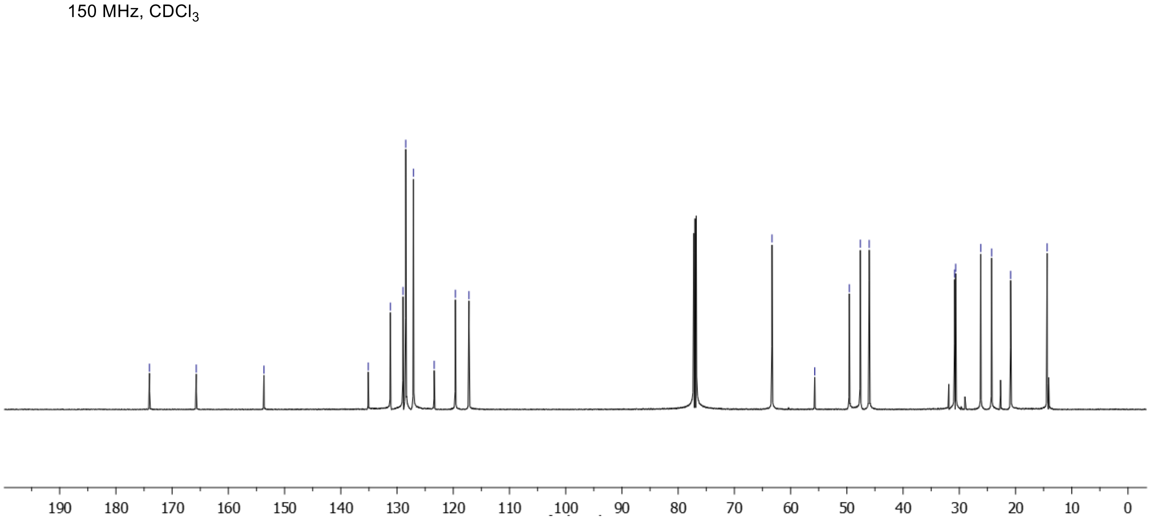

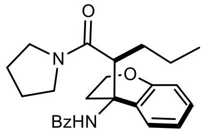

3ai
150 MHz, $CDCl_3$

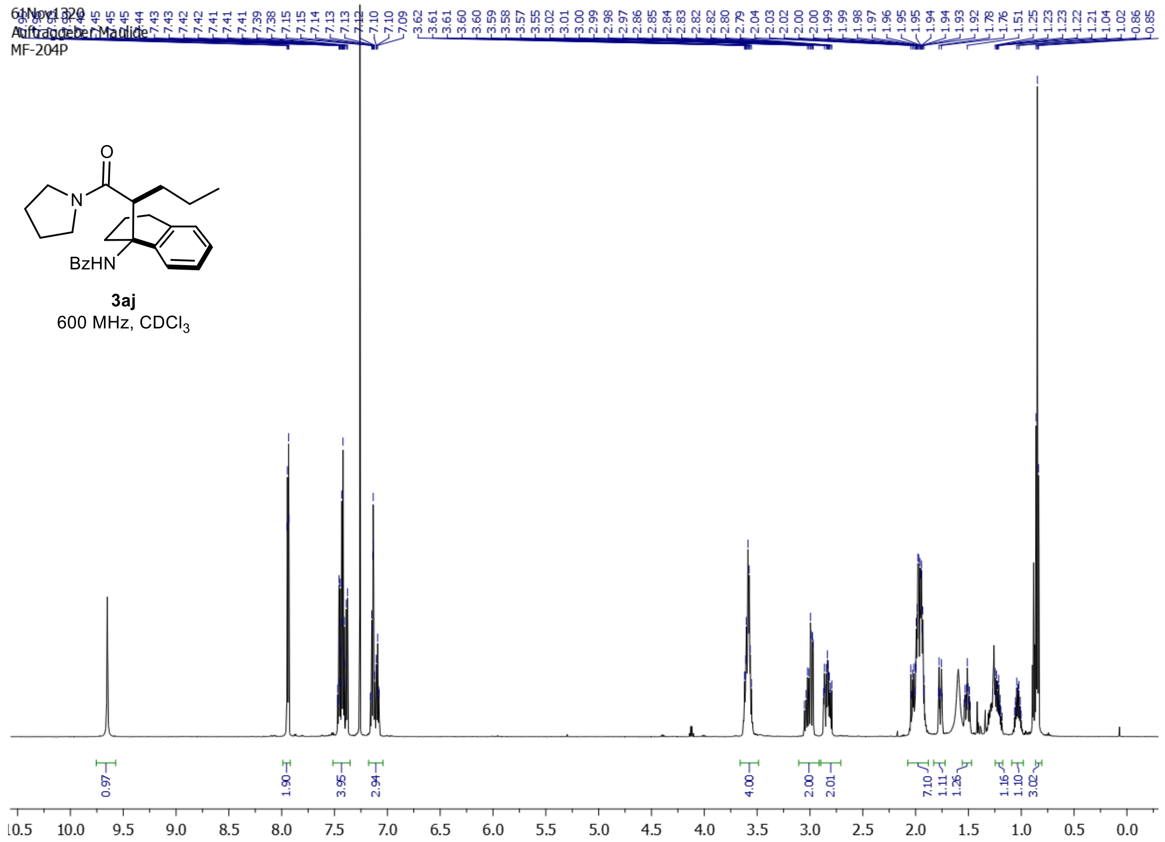
61Nov1320
Auftraggeber Maulide
MF-204P
O
N
BzHN
3aj
600 MHz, CDCl$_3$
0.97
1.90
3.95
2.94
4.00
2.00
2.01
7.10
1.11
1.26
1.16
1.10
3.02
10.5 10.0 9.5 9.0 8.5 8.0 7.5 7.0 6.5 6.0 5.5 5.0 4.5 4.0 3.5 3.0 2.5 2.0 1.5 1.0 0.5 0.0

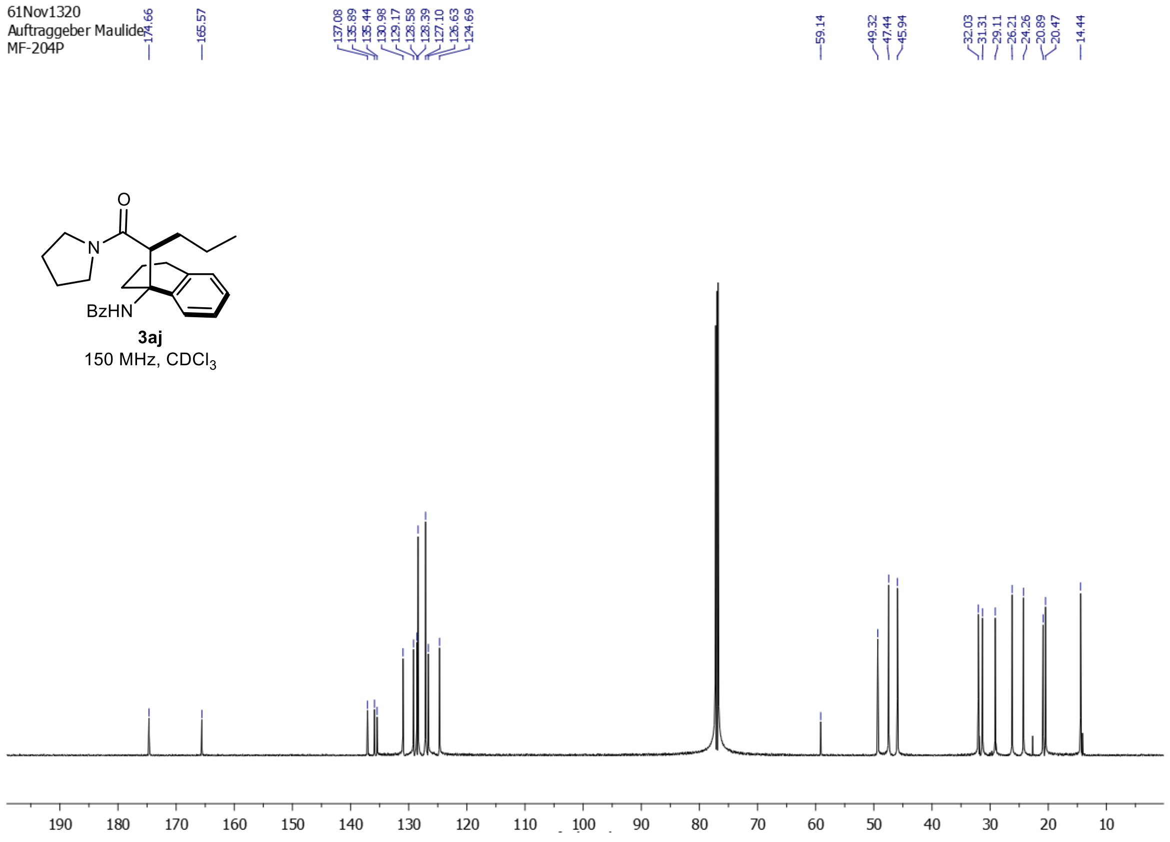
61Nov1320
Auftraggeber Maulide
MF-204P
174.66
165.57
137.08
135.89
135.44
130.98
129.17
128.58
128.39
127.10
126.63
124.69
59.14
49.32
47.44
45.94
32.03
31.31
29.11
26.21
24.26
20.89
20.47
14.44
O
N
BzHN
3aj
150 MHz, CDCl$_3$
190 180 170 160 150 140 130 120 110 100 90 80 70 60 50 40 30 20 10

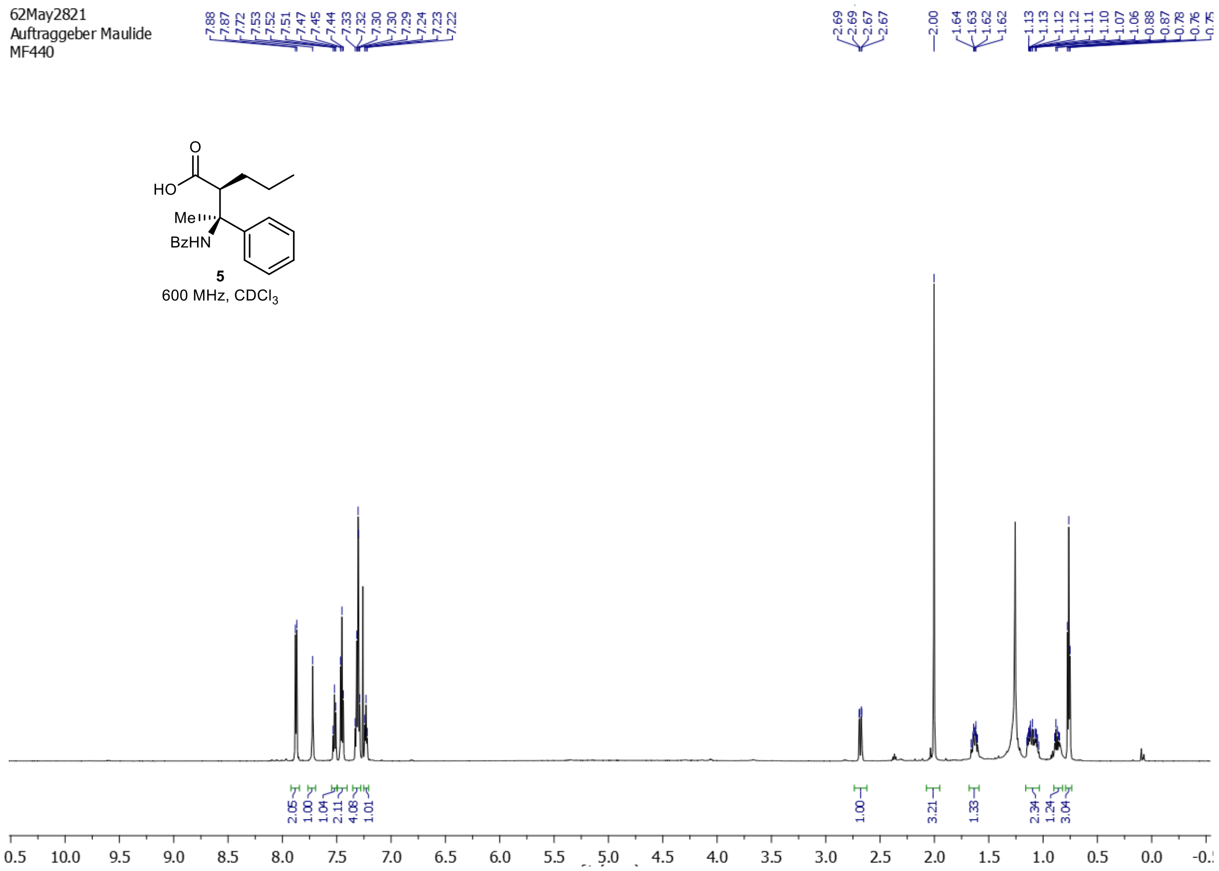
62May2821
Auftraggeber Maulide
MF440
7.88 7.87 7.72 7.53 7.52 7.51 7.47 7.45 7.44 7.33 7.32 7.30 7.29 7.24 7.23 7.22
2.69 2.69 2.67 2.67
2.00
1.64 1.63 1.62 1.62
1.13 1.13 1.12 1.12 1.11 1.10 1.07 1.06 0.88 0.87 0.78
HO
O
Me
BzHN
5
600 MHz, CDCl3
2.05 1.00 1.04 2.11 4.08 1.01
1.00
3.21
1.33
2.34 1.24 3.04
10.0 9.5 9.0 8.5 8.0 7.5 7.0 6.5 6.0 5.5 5.0 4.5 4.0 3.5 3.0 2.5 2.0 1.5 1.0 0.5 0.0

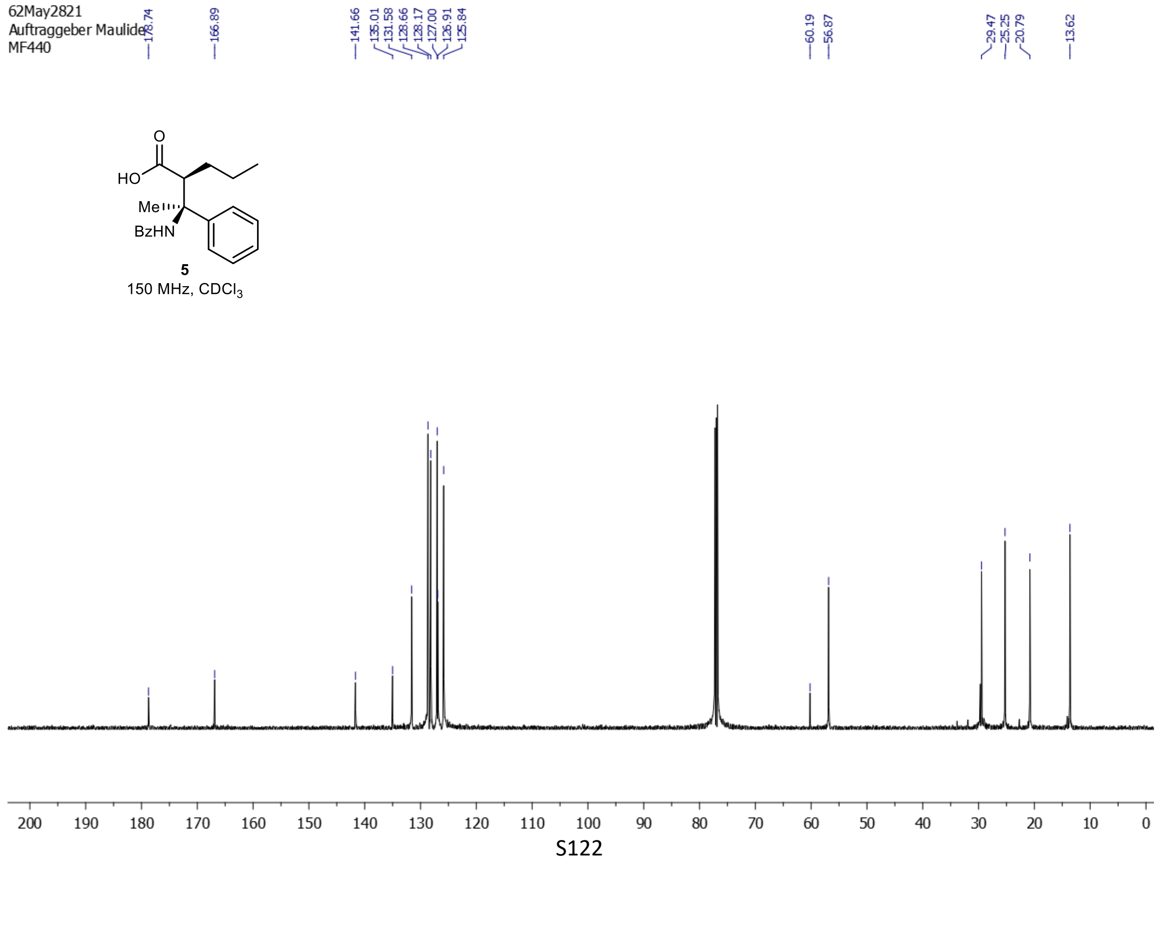
62May2821
Auftraggeber Maulide
MF440
166.89
141.66 135.01 131.58 128.66 128.17 127.00 126.91 125.84
60.19 56.87
29.47 25.25 20.79
13.62
HO
O
Me
BzHN
5
150 MHz, CDCl3
200 190 180 170 160 150 140 130 120 110 100 90 80 70 60 50 40 30 20 10 0

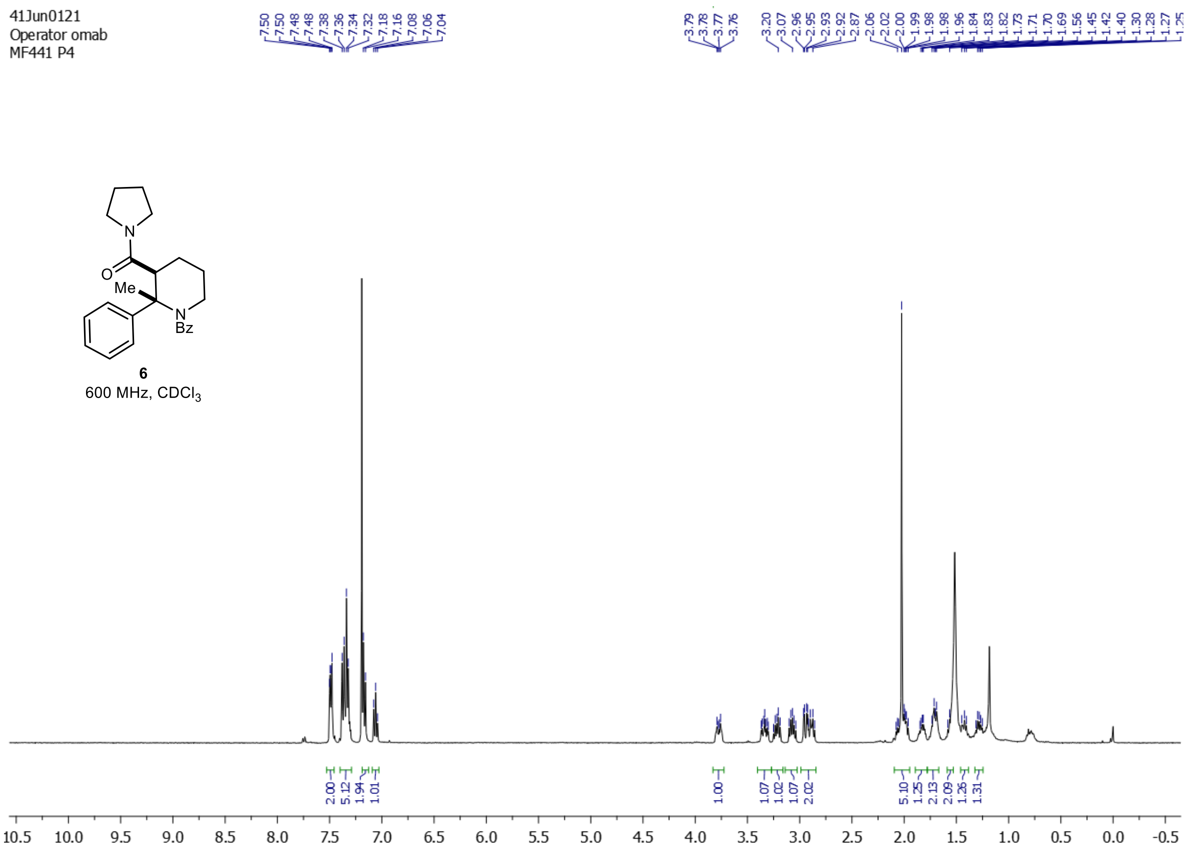

6
600 MHz, $CDCl_3$

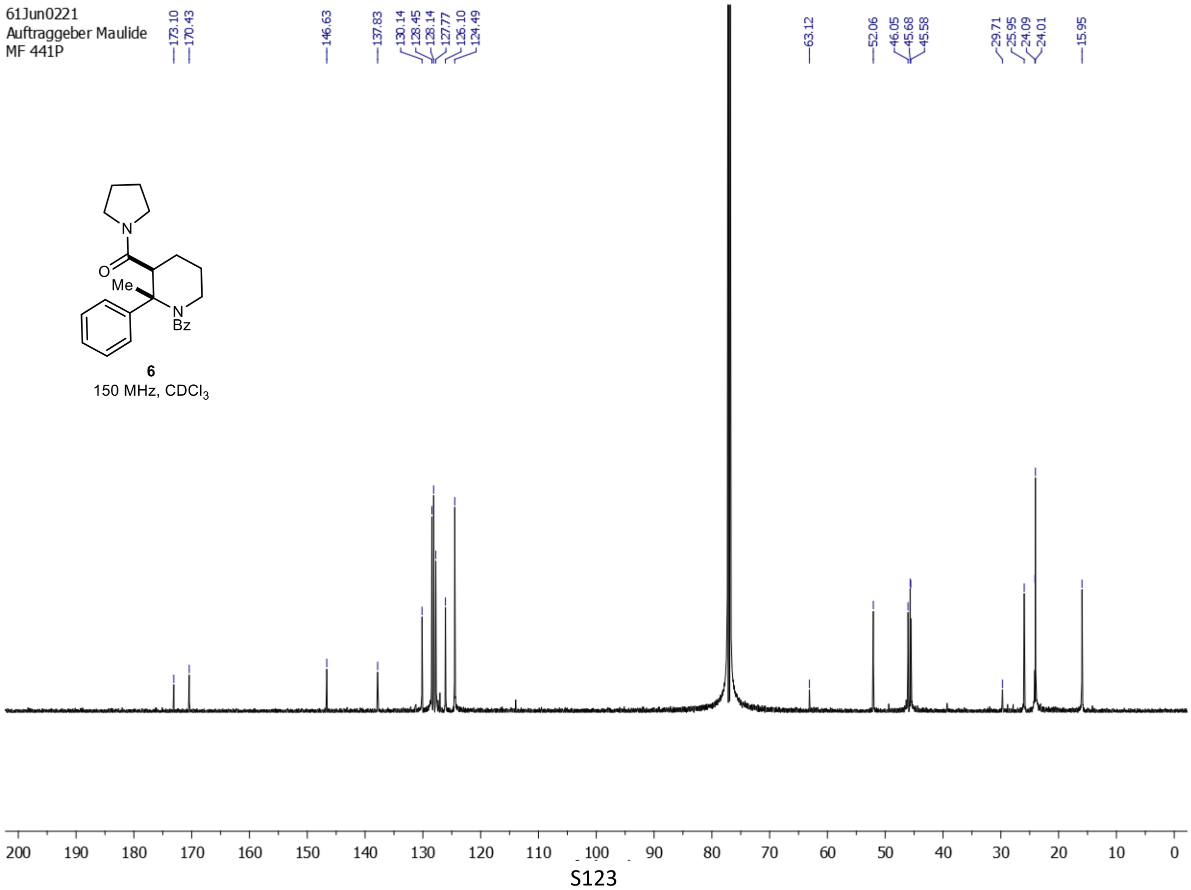

6
150 MHz, $CDCl_3$

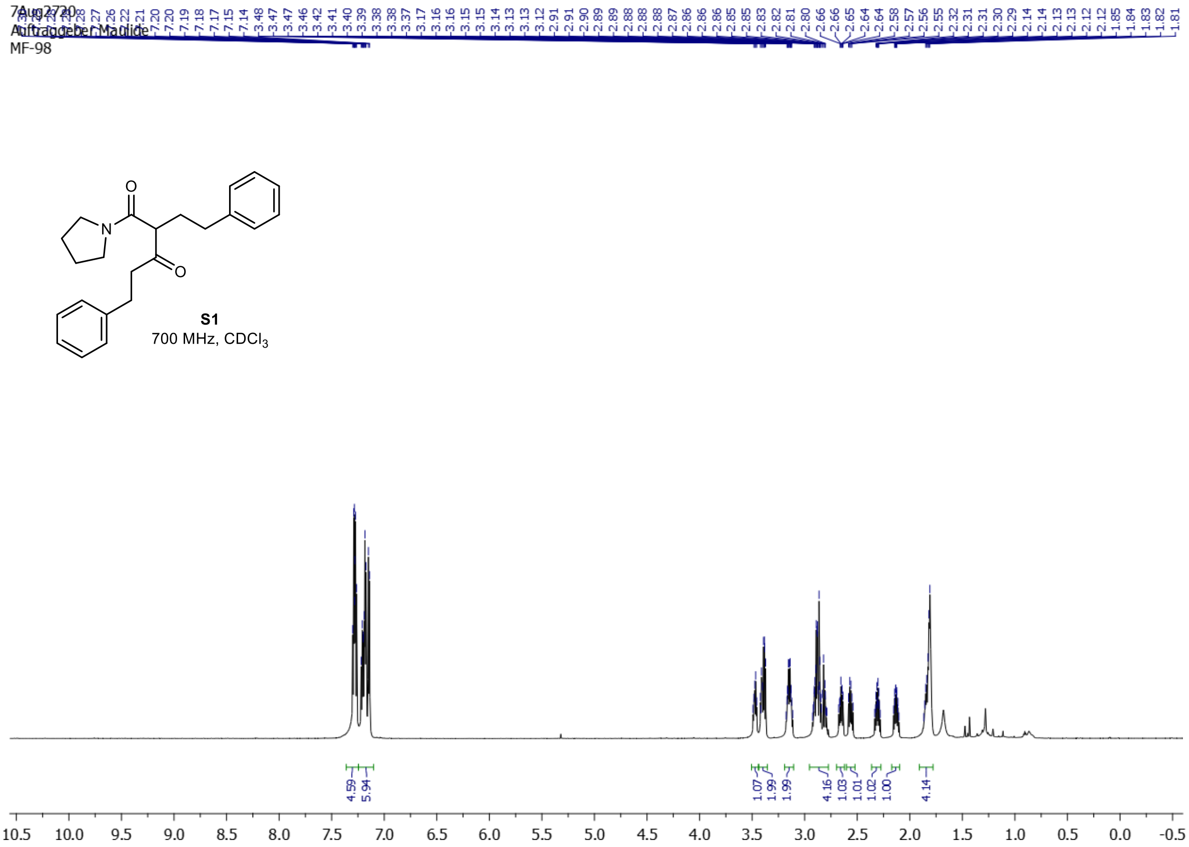
7Aug2720
Auftraggeber Maulide
MF-98
S1
700 MHz, CDCl3
4.59
5.94
1.07
1.99
1.99
4.16
1.03
1.01
1.02
1.00
4.14

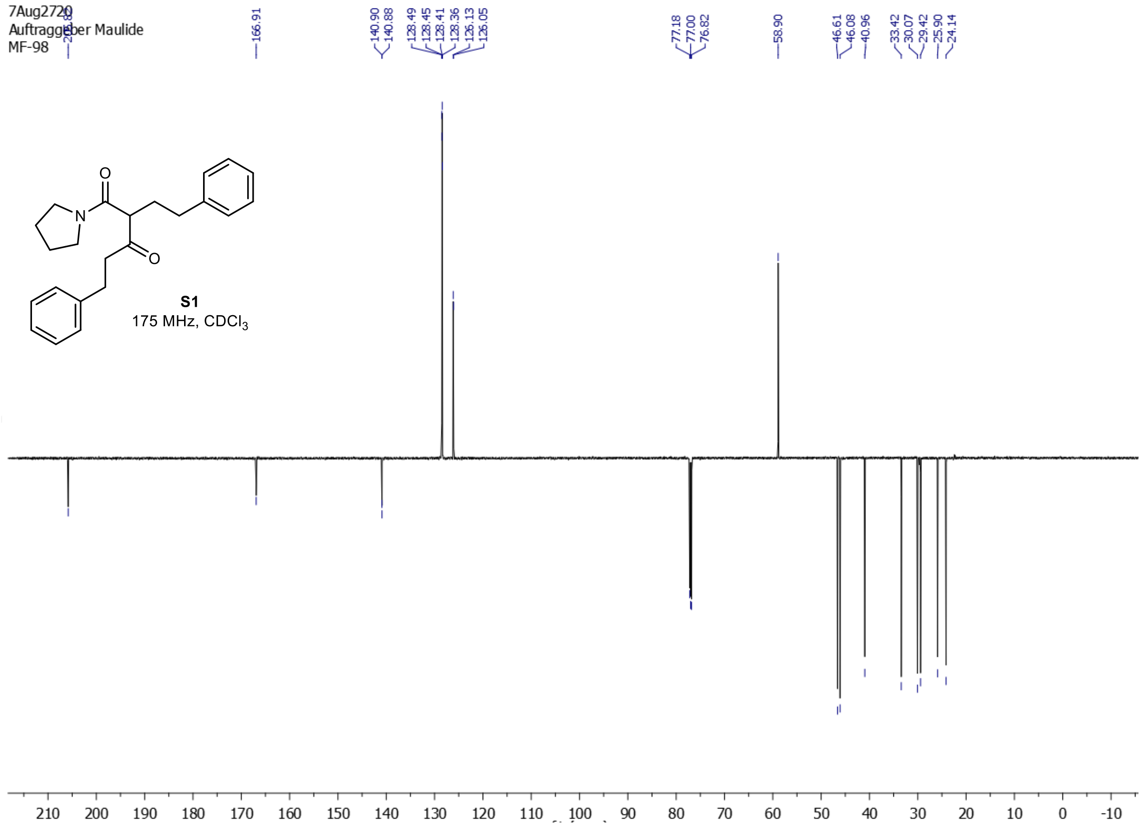
7Aug2720
Auftraggeber Maulide
MF-98
166.91
140.90
140.88
128.49
128.45
128.41
128.36
126.13
126.05
77.18
77.00
76.82
58.90
46.61
46.08
40.96
33.42
30.07
29.42
25.90
24.14
S1
175 MHz, CDCl3

14. References

1. Lam, K. & Markó, I. E. Using toluates as simple and versatile radical precursors. *Org. Lett.* **10**, 2773–2776 (2008).

2. Oare, D. A., Henderson, M. A., Sanner M. A., Heathcock, C. H. Stereochemistry of the Michael addition of N, N-disubstituted amide and thioamide enolates to α, β-unsaturated ketones. *J. Org. Chem.* **55**, 132–157 (1990).

3. Peng, B., Geerdink, D., Farès, C. & Maulide, N. Chemoselective intermolecular α-arylation of amides. *Angew. Chem., Int. Ed.* **53**, 5462–5466 (2014).

4. Tona, V. *et al.* Chemo- and stereoselective transition-metal-free amination of amides with azides. *J. Am. Chem. Soc.* **138**, 8348–8351 (2016).

5. de la Torre, A., Kaiser, D. & Maulide, N. Flexible and chemoselective oxidation of amides to α-keto amides and α-hydroxy Amides. *J. Am. Chem. Soc.* **139**, 6578–6581 (2017).

6. Komeyama, K., Michiyuki, T. & Osaka, I. Nickel/Cobalt-catalyzed C(sp^3)-C(sp^3) cross-coupling of alkyl halides with alkyl tosylates. *ACS Catal.* **9**, 9285–9291 (2019).

7. Zhang, F. *et al.* Cu-Catalyzed cascades to carbocycles: Union of diaryliodonium salts with alkenes or alkynes exploiting remote carbocations. *J. Am. Chem. Soc.* **136**, 8851–8854 (2014).

8. Madelaine, C., Valerio, V. & Maulide, N. Unexpected electrophilic rearrangements of amides: A stereoselective entry to challenging substituted lactones. *Angew. Chem., Int. Ed.* **49**, 1583–1586 (2010).

9. Gnanaprakasam, B. & Milstein, D. Synthesis of amides from esters and amines with liberation of H_2 under neutral conditions. *J. Am. Chem. Soc.* **133**, 1682–1685 (2011).

10. Adler, P. *et al.* α-Fluorination of carbonyls with nucleophilic fluorine. *Nat. Chem.* **11**, 329–334 (2019).

11. Hama, T., Culkin, D. A. & Hartwig, J. F. Palladium-catalyzed intermolecular α-arylation of zinc amide enolates under mild conditions. *J. Am. Chem. Soc.* **128**, 4976–4985 (2006).

12. Teskey, C. J., Adler, P., Gonçalves, C. R. & Maulide, N. Chemoselective α,β-dehydrogenation of saturated amides. *Angew. Chem., Int. Ed.* **58**, 447–451 (2018).

13. Guo, T. *et al.* Highly efficient asymmetric construction of quaternary carbon-containing homoallylic and homopropargylic amines. *Chem. Commun.* **49**, 5402–5404 (2013).

14. Metternich, J. B., Reiterer, M. & Jacobsen, E. N. Asymmetric Nazarov cyclizations of unactivated dienones by hydrogen-bond-donor/Lewis acid Co–catalyzed, enantioselective proton-transfer. *Adv. Synth. Catal.* **362**, 4092–4097 (2020).

15. Yoshinaga, Y., Yamamoto, T. & Suginome, M. Stereoinvertive C–C bond formation at the boron-bound stereogenic centers through copper-bipyridine-catalyzed intramolecular coupling of α-aminobenzylboronic esters. *Angew. Chem. Int. Ed.* **59**, 7251–7255 (2020).

16. Dawood, R. S., Georgiou, I., Wilkie, R. P., Lewis, W. & Stockman, R. A. Asymmetric synthesis of pyrrolidine-containing chemical scaffolds via Tsuji–Trost allylation of N-tert-butanesulfinyl imines. *Chem. Eur. J.* **23**, 11153–11158 (2017).

17. Datta, G. K. & Ellman, J. A. Racemization free protocol for the synthesis of N-tert-butanesulfinyl ketimines. *J. Org. Chem.* **75**, 6283–6285 (2010).

18. Foley, V. M., McSweeney, C. M., Eccles, K. S., Lawrence, S. E. & McGlacken, G. P. Asymmetric Aldol-Tishchenko reaction of sulfinimines. *Org. Lett.* **17**, 5642–5645 (2015).

19. Chen, Q. & Yuan, C. A facile synthesis of chiral 4-(tert-butylsulfinylamino)-2-oxophosphonates and their conversion into 5,5-disubstituted 2-benzylidene-3-oxopyrrolidines. *Synthesis* 1085–1093 (2008).

20. Xiao, M. *et al.* Transition-metal-free hydrogen autotransfer: diastereoselective N-alkylation of amines with racemic alcohols. *Angew. Chem. Int. Ed.* **131**, 10528–10536 (2019).

21. Pablo, Ó. *et al.* A versatile Ru catalyst for the asymmetric transfer hydrogenation of both aromatic and aliphatic sulfinylimines. *Chem. Eur. J.* **18**, 1969–1983 (2012).

22. Mendes, J. A. *et al.* Enantioselective synthesis, DFT calculations, and preliminary antineoplastic activity of dibenzo 1-azaspiro[4.5]decanes on drug-resistant leukemias. *J. Org. Chem.* **84**, 2219–2233 (2019).

23. Tzitzoglaki, C. *et al.* Approaches to primary tert-alkyl amines as building blocks. *Tetrahedron* **75**, 130408–130425 (2019).

24. Tang, T. P. & Ellman, J. A. Asymmetric synthesis of β-amino acid derivatives incorporating a broad range of substitution patterns by enolate additions to tert-butanesulfinyl imines. *J. Org. Chem.* **67**, 7819–7832 (2002).

25. Lundin, P. M. & Fu, G. C. Asymmetric Suzuki cross-couplings of activated secondary alkyl electrophiles: Arylations of racemic α-chloroamides. *J. Am. Chem. Soc.* **132**, 11027–11029 (2010).

26. Pracht, P., Bohle, F. & Grimme, S. Automated exploration of the low-energy chemical space with fast quantum chemical methods. *Phys. Chem. Chem. Phys.* **22**, 7169–7192 (2020).

27. Grimme, S. Exploration of Chemical Compound, Conformer, and Reaction Space with Meta-Dynamics Simulations Based on Tight-Binding Quantum Chemical Calculations. *J. Chem. Theory Comput.* **15**, 2847–2862 (2019).

28. Becke, A. Density Functional Thermochemistry III The Role of Exact Exchange. *J. Chem. Phys.* **98**, 5648–5652 (1993).

29. Lee, C., Yang, W. & Parr, R. G. Development of the Colle-Salvetti correlation-energy formula into a functional of the electron density. *Phys. Rev. B* **37**, 785–789 (1988).

30. Vosko, S. H., Wilk, L. & Nusair, M. Accurate spin-dependent electron liquid correlation energies for local spin density calculations: a critical analysis. *Can. J. Phys.* **58**, 1200–1211 (1980).

31. Stephens, P. J., Devlin, F. J., Chabalowski, C. F. & Frisch, M. J. Ab Initio Calculation of Vibrational Absorption and Circular Dichroism Spectra Using Density Functional Force Fields. *J. Phys. Chem.* **98**, 11623–11627 (1994).

32. Grimme, S., Antony, J., Ehrlich, S. & Krieg, H. A consistent and accurate ab initio parametrization of density functional dispersion correction (DFT-D) for the 94 elements H-Pu. *J. Chem. Phys.* **132**, 154104 (2010).

33. Weigend, F. & Ahlrichs, R. Balanced basis sets of split valence, triple zeta valence and quadruple zeta valence quality for H to Rn: Design and assessment of accuracy. *Phys. Chem. Chem. Phys.* **7**, 3297–305 (2005).

34. Grimme, S., Ehrlich, S. & Goerigk, L. Effect of the damping function in dispersion corrected density functional theory. *J. Comput. Chem.* **32**, 1456–1465 (2011).

35. Riplinger, C. & Neese, F. An efficient and near linear scaling pair natural orbital based local coupled cluster method. *J. Chem. Phys.* **138**, 034106 (2013).

36. Riplinger, C., Sandhoefer, B., Hansen, A. & Neese, F. Natural triple excitations in local coupled cluster calculations with pair natural orbitals. *J. Chem. Phys.* **139**, 134101 (2013).

37. Cancès, E., Mennucci, B. & Tomasi, J. A new integral equation formalism for the polarizable continuum model: Theoretical background and applications to Isotropic and anisotropic dielectrics. *J. Chem. Phys.* **107**, 3032–3041 (1997).

38. Marenich, A. V., Cramer, C. J. & Truhlar, D. G. Universal solvation model based on solute electron density and on a continuum model of the solvent defined by the bulk dielectric constant and atomic surface tensions. *J. Phys. Chem. B* **113**, 6378–6396 (2009).

39. Barone, V. & Cossi, M. Quantum calculation of molecular energies and energy gradients in solution by a conductor solvent model. *J. Phys. Chem. A* **102**, 1995–2001 (1998).

40. Frisch, M. J. *et al.* Gaussian 16, Revision A.03.

41. Neese, F. The ORCA program system. **2**, 73–78 (2012).

42. Smith, D. G. A. *et al.* PSI4 1.4: Open-source software for high-throughput quantum chemistry. *J. Chem. Phys.* **152**, 184108 (2020).

43. Hohenstein, E. G., Parrish, R. M., Sherrill, C. D., Turney, J. M. & Schaefer, H. F. Large-scale symmetry-adapted perturbation theory computations via density fitting and Laplace transformation techniques: Investigating the fundamental forces of DNA-intercalator interactions. *Journal of Chemical Physics* **135**, (2011).

44. Hohenstein, E. G. & Sherrill, C. D. Density fitting and Cholesky decomposition approximations in symmetry-adapted perturbation theory: Implementation and application to probe the nature of π - π. *Journal of Chemical Physics* **132**, (2010).
